# Integrative Modeling of Read Depth and B-Allele Frequency Improves Single-Cell Copy Number Calling from Targeted DNA Sequencing Panels

**DOI:** 10.34133/csbj.0203

**Published:** 2026-08-21

**Authors:** Dong Pei, Rachel Griffard-Smith, Brahian Cano Urrego, Emily Schueddig

**Affiliations:** ^1^ Department of Biostatistics & Data Science, University of Kansas Medical Center, Kansas City, KS, USA.; ^2^ The University of Kansas Cancer Center, Kansas City, KS, USA.

## Abstract

Copy number variations (CNVs) drive cancer initiation and progression, but resolving them at single-cell resolution from targeted DNA sequencing panels remains challenging. The Mission Bio Tapestri platform generates 2 complementary signals for CNV inference: sequencing depth and B-allele frequency (BAF) from heterozygous variants; however, existing methods such as karyotapR rely primarily on read depth, leaving allele-specific events unused. Here, we introduce scPloidyR, a hidden Markov model (HMM) that jointly models read depth and BAF at amplicon resolution for single-cell copy number calling from Tapestri data. scPloidyR fits per-chromosome Markov chains with copy number as the hidden state, factorizes emissions into depth and BAF likelihoods, and learns parameters by Baum–Welch expectation-maximization with Viterbi decoding. We compared scPloidyR with the established karyotapR Gaussian mixture model (GMM) in 2 simulation studies spanning BAF noise, variant density, amplicon density, sample size, and heterozygosity rate, and on a public Tapestri 5-cell-line mixture dataset. In simulations, scPloidyR substantially outperformed karyotapR on class-balanced metrics (macro-F1: 0.477 versus 0.273; alteration F1: 0.903 versus 0.381 in simulation study 1) when allelic information was available. Adding just one heterozygous variant per amplicon increased scPloidyR accuracy from 0.556 to 0.897 for gains. However, when BAF information was absent, karyotapR outperformed scPloidyR, and high BAF noise sharply degraded joint-model performance. On real data, scPloidyR produced more spatially coherent and biologically plausible copy number profiles. These results show that joint depth-BAF modeling benefits single-cell CNV calling when allelic information is available, while depth-only methods remain preferable when it is absent.

## Introduction

Copy number variations (CNVs), which include genomic gains or losses ranging from kilobases to entire chromosomes, are frequent drivers of cancer initiation and progression [1]. Genome-wide aneuploidy, gains or losses of whole chromosomes, is found in roughly 90% of solid tumors and about 75% of hematopoietic malignancies, highlighting its pervasive role in tumorigenesis [2]. Somatic CNVs activate oncogenes through amplification and inactivate tumor suppressors through deletion, and recurrent patterns of copy number alteration have been cataloged across dozens of cancer types [1,3]. Copy number signatures derived from recurrent patterns of genomic gain and loss can help characterize tumors and stratify them by biological processes and clinical outcomes [4]. Beyond these linear copy number signatures, the 3-dimensional organization of chromosomes offers a complementary structural view, and network-based representations of chromatin architecture can link chromosome topology to linear genome annotations [5]. Despite their clinical importance, bulk sequencing averages CNV signals across millions of cells, masking the intra-tumor heterogeneity that drives treatment resistance and clonal evolution [6]. Single-cell DNA sequencing overcomes this limitation by resolving copy number profiles at the level of individual cells, enabling reconstruction of clonal architectures and identification of rare subpopulations [6,7]. Single-cell and multi-omics approaches have also been used to dissect cellular dynamics and intra-tumor heterogeneity across solid tumors and to characterize malignant subpopulations and their therapeutic vulnerabilities [8,9].

The Mission Bio Tapestri platform is a droplet-based microfluidic system that performs targeted single-cell DNA sequencing of thousands of cells per run [10]. By amplifying a defined panel of genomic loci, Tapestri generates 2 complementary signals for CNV inference: sequencing depth (amplicon read counts), which reports total DNA content, and B-allele frequency (BAF) from heterozygous single-nucleotide polymorphisms (SNPs), which provides allelic information that can distinguish copy number states with similar total counts [10]. The platform has been widely adopted for profiling hematologic malignancies and solid tumors at single-cell resolution [10–14], and targeted single-cell DNA sequencing continues to be used to dissect clonal heterogeneity and evolution in malignancies such as acute myeloid leukemia [15]. Rapid multiplex amplicon nanopore sequencing has also been demonstrated as a feasible approach for profiling central nervous system tumor markers within an intraoperative timeframe [16].

Several computational tools have been developed for analyzing Tapestri data. The optima R package provides an integrated toolkit for processing Tapestri single-cell multi-omics data, including data import, normalization, quality filtering, and visualization [17]. karyotapR extends this ecosystem with a Gaussian mixture model (GMM) approach to copy number calling: It normalizes amplicon read counts against a set of reference cells, simulates synthetic cells at each copy number state using Weibull distributions fitted to reference data, and classifies cells by posterior probability under Gaussian components [18,19]. karyotapR has been shown to detect chromosome- and arm-scale aneuploidy across thousands of single cells [18]. However, karyotapR classifies each amplicon independently without exploiting the spatial ordering of loci along chromosomes, and it relies primarily on read depth, leaving BAF information underused and potentially missing allele-specific events such as copy-neutral loss of heterozygosity.

Hidden Markov models (HMMs) provide an alternative framework for copy number inference because they exploit the spatial ordering of genomic loci along chromosomes [20]. HMM-based methods model copy number states as hidden variables that transition rarely between adjacent loci, with observed signals (depth and/or BAF) emitted according to state-dependent distributions. PennCNV demonstrated this approach on SNP arrays by jointly modeling log *R* ratio and BAF within a 6-state HMM [21], and HoneyBADGER extended HMM-based CNV detection to single-cell RNA sequencing by integrating allelic and expression signals through a Bayesian hierarchical framework [22]. More recently, Numbat combined population-based haplotype phasing with expression and allele signals to infer copy number from single-cell transcriptomes [23]. These methods have been successfully applied to array comparative genomic hybridization (CGH) [24], SNP genotyping arrays [21], and single-cell transcriptomic data [22,23], but the transcriptomic methods infer copy number indirectly from gene expression, and HMM-based approaches have not been systematically adapted for targeted single-cell DNA platforms like Tapestri, where amplicon panels are sparse and unevenly distributed across the genome, as is typical of many classes of genomic features, including noncoding loci [25].

Integrating BAF alongside read depth can improve CNV detection by resolving allele-specific events, such as copy-neutral loss of heterozygosity and unbalanced gains, that are invisible to depth-only methods [21,26]. However, the value of BAF information depends on the availability of heterozygous variants within the targeted panel, which varies across loci and individuals.

Here, we introduce scPloidyR, an R package that implements an HMM that jointly models read depth and BAF at amplicon resolution for single-cell CNV inference from Tapestri data. We compare scPloidyR with the established karyotapR GMM method through systematic simulation studies that evaluate performance across varying levels of simulation parameters (BAF noise, variant density, amplicon density, sample size, and heterozygosity rate) that govern the information content available to each method and through application to a public Tapestri cell mixture dataset. Our results characterize the conditions under which joint depth-BAF modeling improves copy number calling and identify regimes where depth-only inference remains preferable.

## Results

### Simulation study 1

**Fig. 1.**
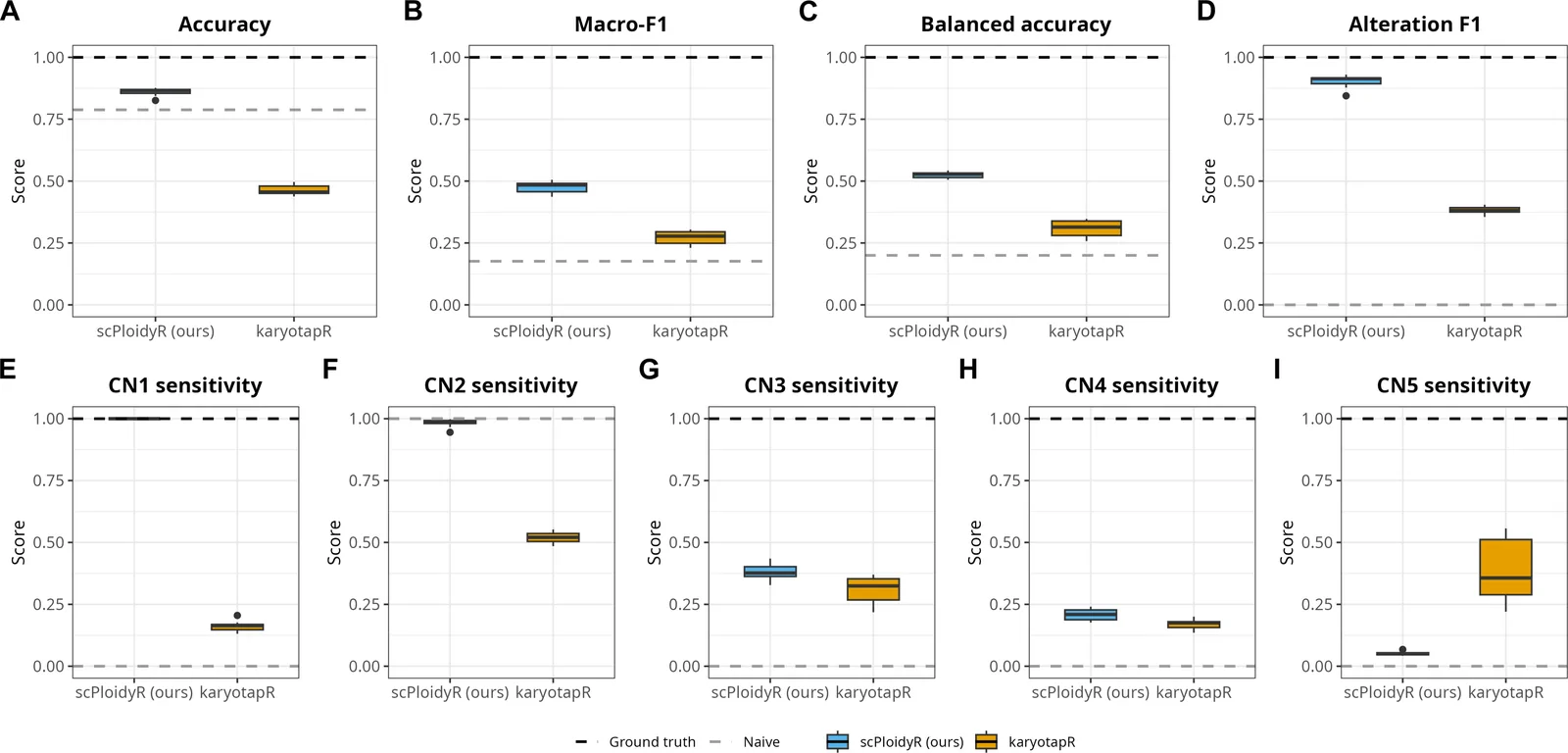
Simulation study 1: Comparison of scPloidyR and karyotapR (GMM) across copy number states 1 to 5 over 10 independent replicates, with dashed lines for the ground truth (upper bound) and naive diploid predictor (always CN = 2). (A) Accuracy, (B) macro-F1, (C) balanced accuracy, (D) alteration F1, and (E to I) per-class sensitivity for CN states 1 to 5.

Simulation study 1 mixed multiple cell populations across copy number states to approximate real datasets with heterogeneous karyotypes (see Methods). Across 10 replicates, scPloidyR consistently outperformed karyotapR on all discrimination-focused metrics. Mean accuracy for scPloidyR was 0.860, compared with 0.464 for karyotapR and 0.788 for the naive diploid predictor (Fig. 1A); however, naive accuracy reflects the dominance of diploid segments and does not indicate true CNV detection ability. scPloidyR achieved substantially higher macro-F1 (0.477 versus 0.273; Fig. 1B) and balanced accuracy (0.525 versus 0.310; Fig. 1C), demonstrating improved performance across all copy number classes rather than only the dominant class. scPloidyR also maintained high alteration F1 (0.903 versus 0.381 for karyotapR; Fig. 1D), whereas the naive baseline had zero alteration F1. The ground truth, as expected, achieved perfect scores across all metrics.

Per-class sensitivity analysis reveals how each method performs across individual copy number states. For single-copy deletions (CN 1), scPloidyR achieved perfect sensitivity (1.000) compared with 0.162 for karyotapR (Fig. 1E). For the diploid state (CN 2), scPloidyR achieved 0.983 sensitivity compared with 0.520 for karyotapR (Fig. 1F), indicating that scPloidyR correctly identifies the majority class far more reliably. For single-copy gains (CN 3), scPloidyR modestly outperformed karyotapR (0.382 versus 0.310; Fig. 1G). At higher ploidy states, sensitivity dropped for both methods: CN 4 sensitivity was 0.208 for scPloidyR versus 0.171 for karyotapR (Fig. 1H), while for CN 5, karyotapR showed higher sensitivity than scPloidyR (0.387 versus 0.052; Fig. 1I), suggesting that the GMM approach may better distinguish extreme gains. The naive diploid predictor, by construction, has zero sensitivity for all nondiploid states. These per-class results show that aggregate metrics such as accuracy can obscure important differences in method behavior across copy number states. Full numerical results for all metrics are provided in File S5.

To isolate the source of scPloidyR’s advantage, we ablated its emission and transition components on this benchmark (File S9). Removing the allelic channel (a depth-only HMM) collapsed macro-F1 to 0.101, because read depth alone cannot separate 5 states under the realistic overdispersion of the simulation, whereas removing the HMM transition structure (a joint non-HMM decode that classifies each amplicon independently) reduced macro-F1 to 0.320 and lowered alteration F1 from 0.903 to 0.541 by introducing false alterations in diploid regions. Both BAF integration and HMM smoothing therefore contribute strongly to the full model. A BAF-only HMM matched the full model on this BAF-rich benchmark (macro-F1 0.479 versus 0.477), with the depth channel’s contribution concentrated in the low-information regime characterized in simulation study 2, where allelic signal is sparse or absent.

### Simulation study 2

Simulation study 2 varied 5 parameters one at a time—BAF noise, variant density, amplicon density, sample size, and heterozygosity rate—with 3 replicates per condition (see Methods). Each condition was evaluated separately for gains (CN 3) and losses (CN 1). Across most conditions, scPloidyR outperformed karyotapR, but the magnitude of the gap varied substantially by parameter, and karyotapR achieved higher accuracy when BAF information was absent, i.e., zero variants or zero heterozygosity. The subsections below describe each tested variable in detail.

#### BAF noise

**Fig. 2.**
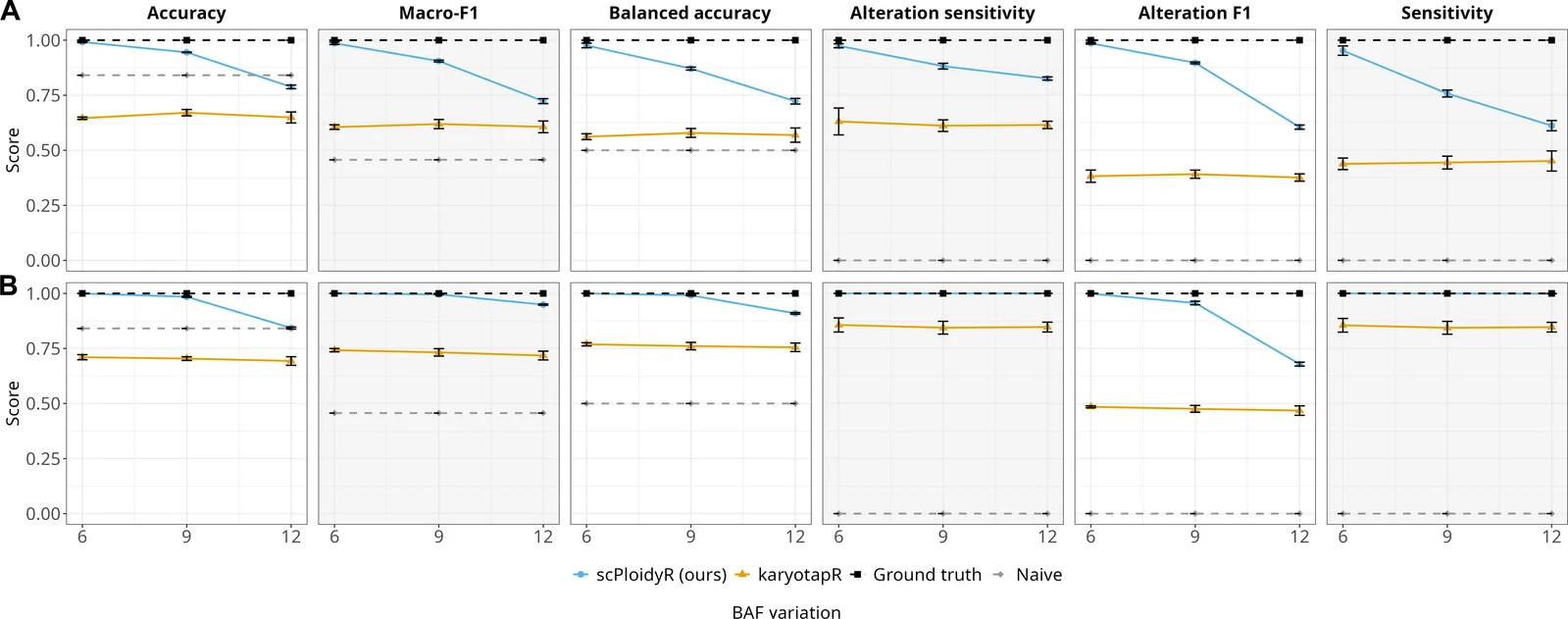
Simulation study 2: BAF noise univariate analysis. (A) Copy number gains (CN = 3). (B) Copy number losses (CN = 1). Each panel plots a performance metric as a function of BAF noise (SD of allele frequency perturbation) with 3 replicates per condition. Lines represent scPloidyR (blue), karyotapR (GMM, orange), the ground truth (black), and the naive diploid predictor (gray).

Increasing BAF noise strongly degraded scPloidyR performance while leaving karyotapR essentially unchanged. For gains, scPloidyR accuracy fell from 0.992 (SD = 6) to 0.945 (SD = 9) to 0.788 (SD = 12), with macro-F1 declining in parallel from 0.986 to 0.905 to 0.723 and alteration F1 from 0.986 to 0.897 to 0.605. karyotapR accuracy remained flat at approximately 0.66 across all noise levels (0.646, 0.671, 0.649), and its alteration F1 remained near 0.38 to 0.39. A sharp inflection occurred between SD = 9 and SD = 12, where scPloidyR’s balanced accuracy dropped from 0.871 to 0.723. For losses, the pattern was similar but less severe: scPloidyR accuracy declined from 0.999 to 0.985 to 0.843, while karyotapR remained near 0.69 to 0.71. These results indicate that scPloidyR’s advantage depends heavily on BAF signal quality, with high noise levels reducing its ability to discriminate copy number states (Fig. 2).

#### Variant density

**Fig. 3.**
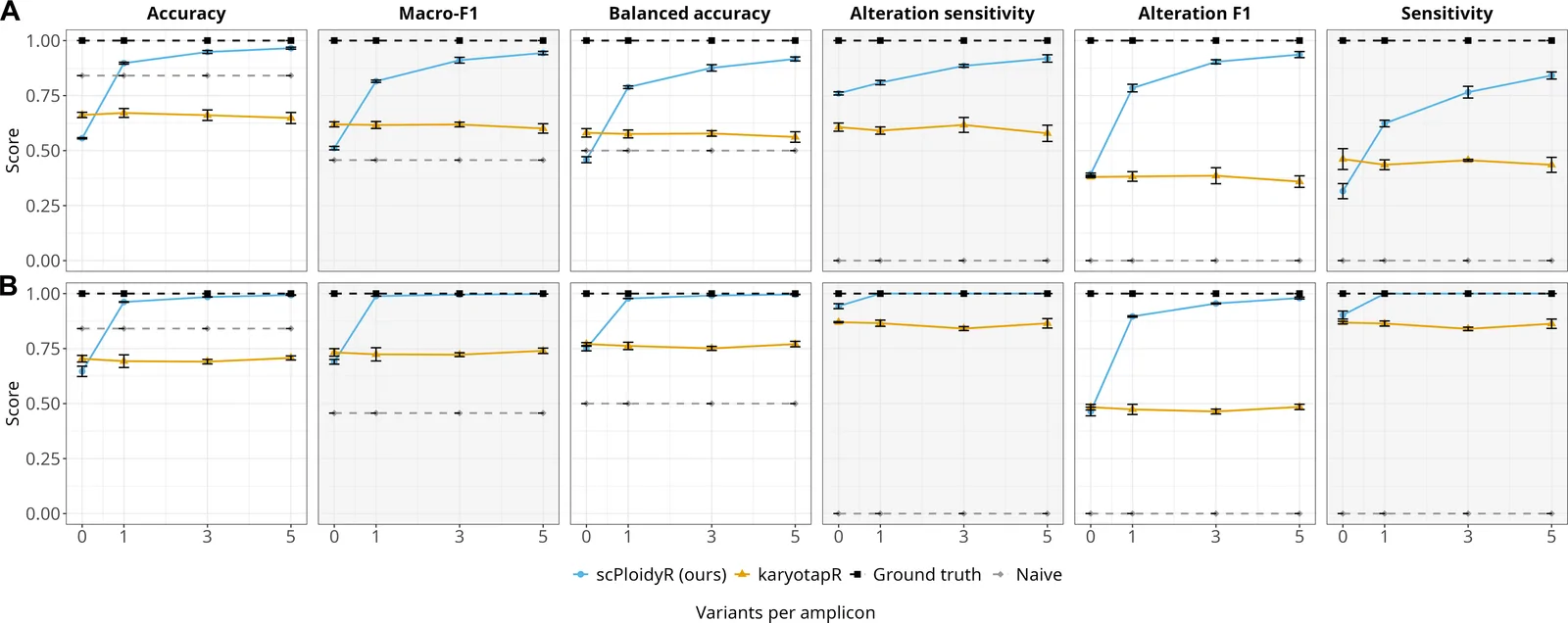
Simulation study 2: Variant density univariate analysis. (A) Copy number gains (CN = 3). (B) Copy number losses (CN = 1). Performance metrics plotted as a function of heterozygous SNPs per amplicon (0, 1, 3, 5). Layout and conventions as in Fig. 2.

Variant density, heterozygous SNPs per amplicon, produced the largest change in scPloidyR performance. For gains, scPloidyR accuracy jumped from 0.556 with zero variants to 0.897 with one variant per amplicon. Performance continued to improve with additional variants (0.948 at 3, 0.965 at 5) but with diminishing returns. Alteration F1 showed the same pattern: 0.393 (0 variants), 0.784 (1 variant), 0.904 (3 variants), and 0.936 (5 variants). karyotapR was unaffected by variant count, with accuracy between 0.648 and 0.671 across all levels. With zero variants, i.e., depth-only mode, scPloidyR lost its BAF advantage entirely and performed below the naive baseline in accuracy (0.556 versus 0.841). For losses, the zero-variant deficit was smaller but karyotapR again outperformed scPloidyR (0.704 versus 0.646), and adding even one variant restored near-perfect scPloidyR performance (0.962) (Fig. 3). To anchor this range, we tabulated variants per amplicon in the 5-cell-line panel (319 amplicons, 584 variants). Amplicons carried a median of one mapped variant (mean 1.8), while informative heterozygous variants (minor allele frequency above 0.3) averaged about 0.4 per amplicon, with 41% (131 of 319) carrying none across all 5 lines.

#### Amplicon density

**Fig. 4.**
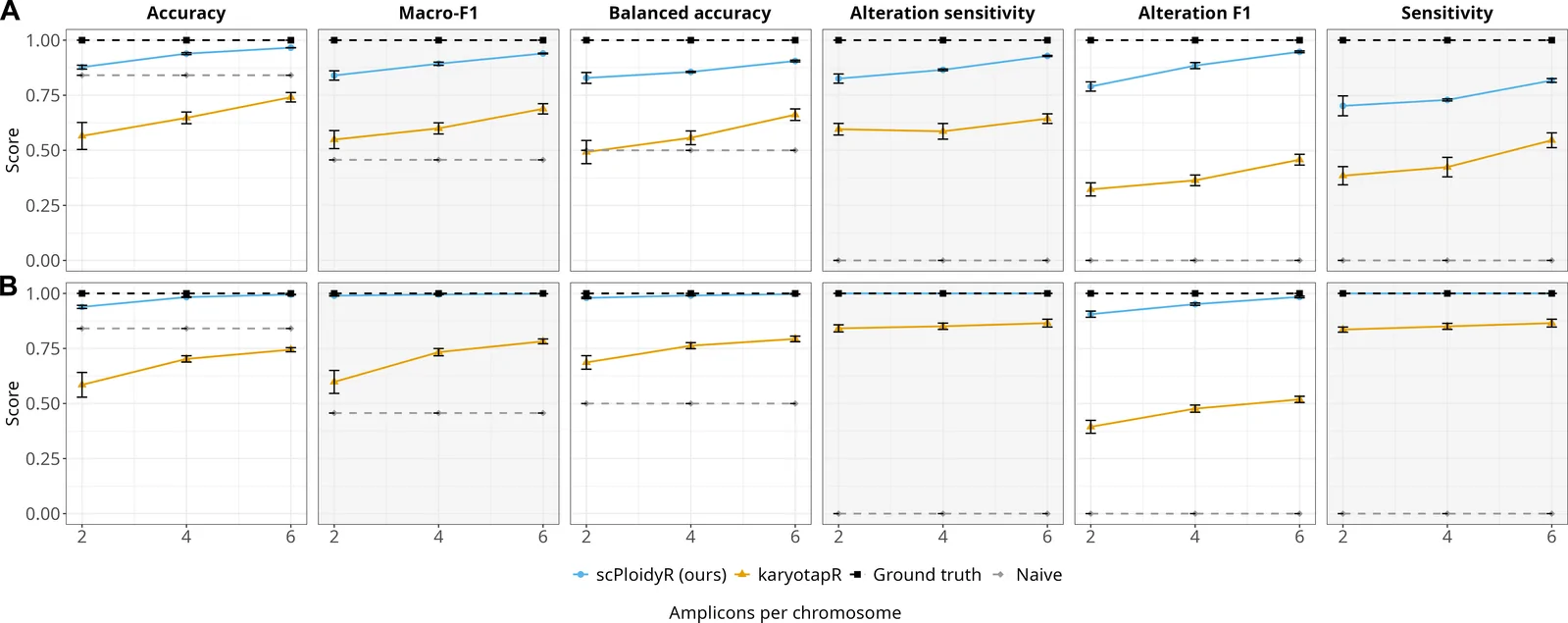
Simulation study 2: Amplicon density univariate analysis. (A) Copy number gains (CN = 3). (B) Copy number losses (CN = 1). Performance metrics plotted as a function of amplicons per chromosome (2, 4, 6). Layout and conventions as in Fig. 2.

Both methods improved with more amplicons per chromosome, but the pattern differed. karyotapR showed the larger relative improvement for gains, with accuracy rising from 0.565 (2 amplicons) to 0.647 (4 amplicons) to 0.741 (6 amplicons), a 31% relative improvement. scPloidyR improved more modestly in absolute terms (0.878 to 0.939 to 0.966) since it was already performing well at low amplicon counts. Alteration F1 for scPloidyR rose from 0.790 to 0.885 to 0.947, while karyotapR improved from 0.323 to 0.364 to 0.457. The improvement was monotonic for both methods with no inflection point, suggesting that additional spatial resolution continues to benefit copy number inference. For losses, scPloidyR was already near-perfect at 2 amplicons (accuracy 0.939) and reached 0.995 at 6 amplicons (Fig. 4).

#### Sample size

**Fig. 5.**
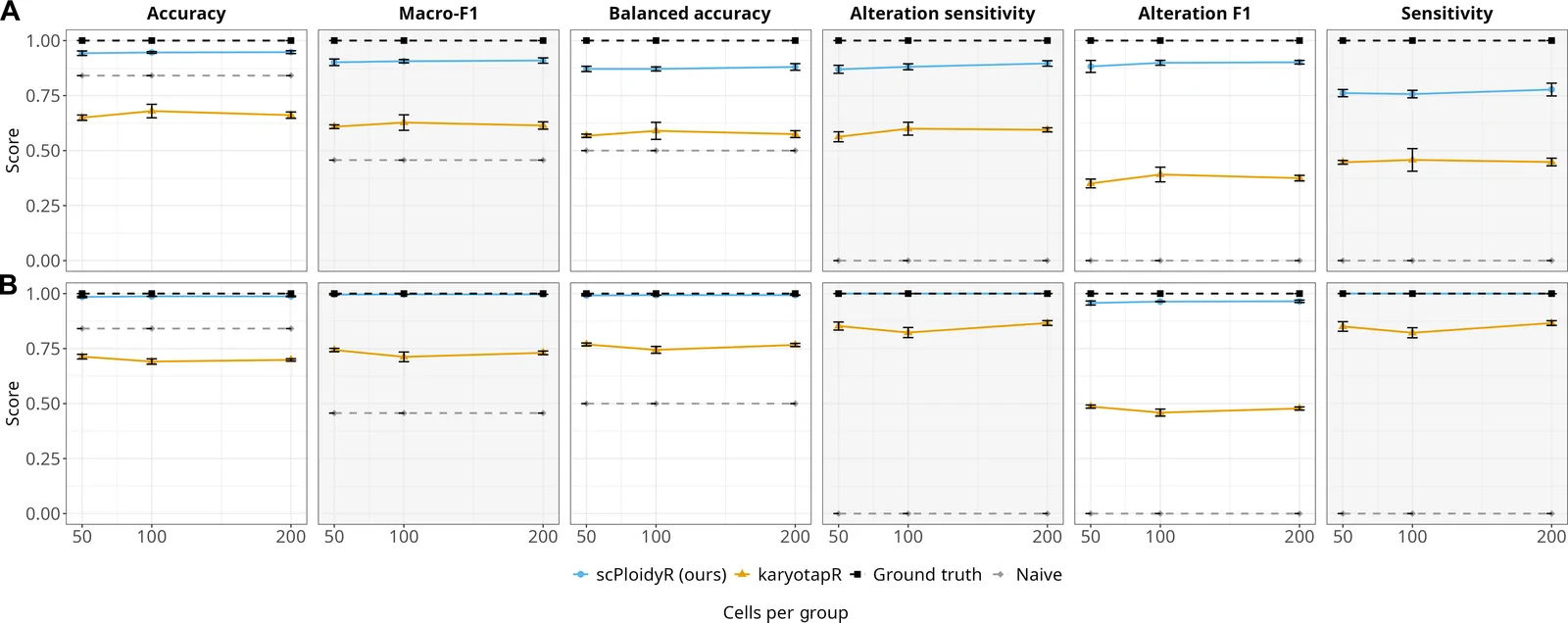
Simulation study 2: Sample size univariate analysis. (A) Copy number gains (CN = 3). (B) Copy number losses (CN = 1). Performance metrics plotted as a function of cells per group (50, 100, 200). Layout and conventions as in Fig. 2.

Sample size, cells per group, had minimal impact on either method. For gains, scPloidyR accuracy was 0.943 at 50 cells, 0.946 at 100 cells, and 0.948 at 200 cells; macro-F1 varied by less than 0.01 across conditions (0.901 to 0.909). karyotapR showed similarly flat performance (accuracy 0.650 to 0.680). For losses, the same stability held: scPloidyR accuracy ranged from 0.985 to 0.988 and karyotapR from 0.691 to 0.713. These results indicate that reference panel estimation is stable across different number of cells within a group (Fig. 5).

#### Heterozygosity rate

**Fig. 6.**
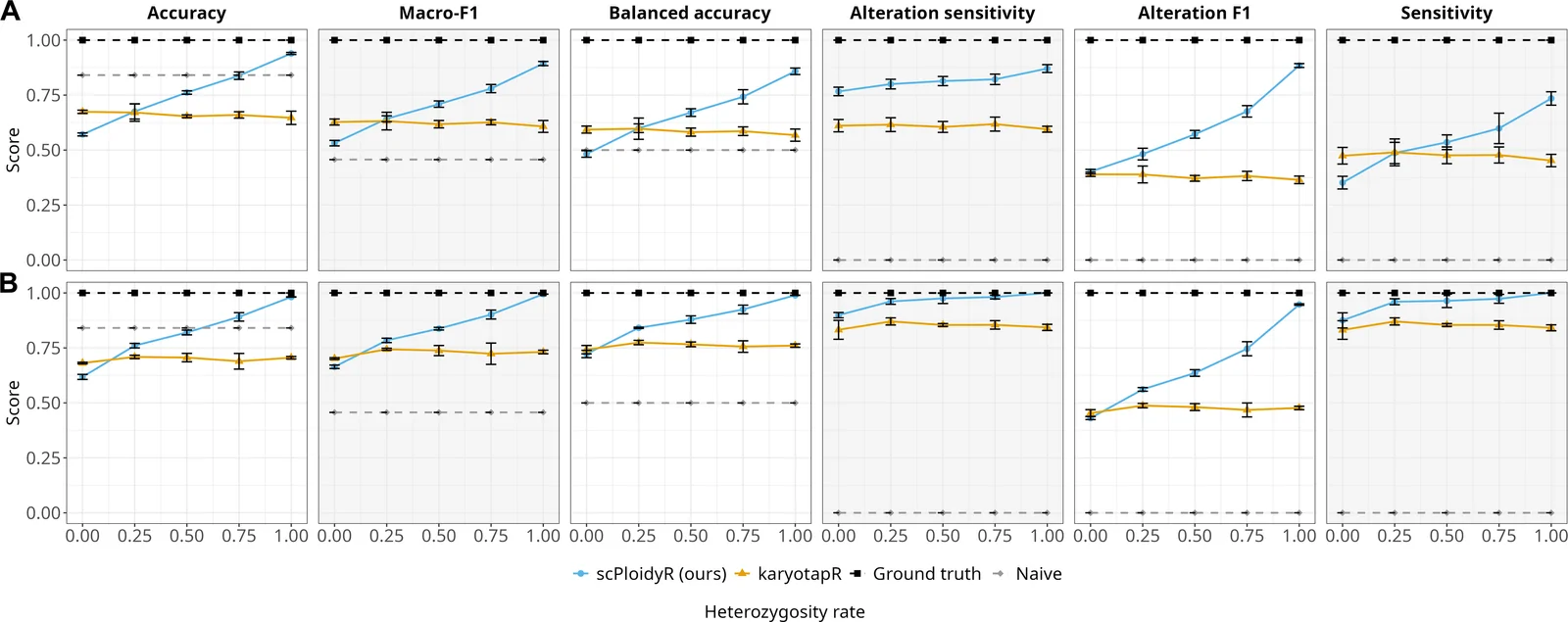
Simulation study 2: Heterozygosity rate univariate analysis. (A) Copy number gains (CN = 3). (B) Copy number losses (CN = 1). Performance metrics plotted as a function of heterozygosity rate (0%, 25%, 50%, 75%, 100%). Layout and conventions as in Fig. 2.

Heterozygosity rate produced one of the 2 largest performance swings for scPloidyR, alongside variant density. For gains, accuracy rose from 0.571 at 0% heterozygosity to 0.675 (25%), 0.762 (50%), 0.838 (75%), and 0.939 (100%). Alteration F1 rose across the same levels: 0.402, 0.482, 0.572, 0.676, and 0.885. The alteration F1 curve was nonlinear, with the steepest increase between 75% and 100% heterozygosity. karyotapR remained stable across all heterozygosity levels (accuracy 0.647 to 0.674, alteration F1 0.365 to 0.390). At 0% heterozygosity, karyotapR outperformed scPloidyR (0.674 versus 0.571), demonstrating that depth-only inference surpasses the HMM when no allelic information is available. By 25% heterozygosity, the 2 methods were essentially tied (scPloidyR 0.675 versus karyotapR 0.671), so even a quarter of sites carrying heterozygous variants was enough to close the gap. For losses, heterozygosity had a qualitatively similar but less extreme effect: scPloidyR accuracy rose from 0.618 (0%) to 0.982 (100%). At 0% heterozygosity for losses, karyotapR again outperformed scPloidyR (0.681 versus 0.618) (Fig. 6).

#### Summary of simulation study 2 trends

Across the 5 tested variables, scPloidyR performance was primarily driven by BAF-related parameters, namely, heterozygosity rate, variant density, and BAF noise, which jointly determine the information content of allele frequency signals. When BAF information was absent, i.e., zero variants per amplicon or 0% heterozygosity, karyotapR outperformed scPloidyR for both gains and losses. karyotapR was mainly sensitive to amplicon density, which governs spatial resolution for depth-based inference. Sample size had minimal impact on either method. Losses were consistently easier to detect than gains for both methods, likely because monosomy produces a larger departure from the diploid baseline than trisomy. Detailed metric values for all conditions are provided in Files S6 (gains) and S7 (losses).

#### CNV event size and detection resolution

The preceding simulations alter whole chromosomes and therefore do not isolate how detection depends on the physical size of a copy number event. To characterize the effective resolution and detection limits of scPloidyR, we simulated alterations that span an increasing number of consecutive amplicons within a chromosome (see Methods). Using a higher-density panel of 20 amplicons per chromosome so that a chromosome arm corresponds to 10 amplicons, we placed contiguous alterations of 5 sizes—single-amplicon (1 amplicon), small focal (3 amplicons), medium focal (8 amplicons), chromosome-arm (10 amplicons, half the chromosome), and whole-chromosome (20 amplicons)—and scored detection at amplicon resolution for both gains (CN 3) and losses (CN 1), with 5 replicates per condition. Because scPloidyR calls copy number per amplicon, whereas karyotapR aggregates to whole chromosomes, the chromosome-level karyotapR call was broadcast to its constituent amplicons for a like-for-like comparison; detection sensitivity is the fraction of truly altered amplicons recovered in the correct direction.

**Fig. 7.**
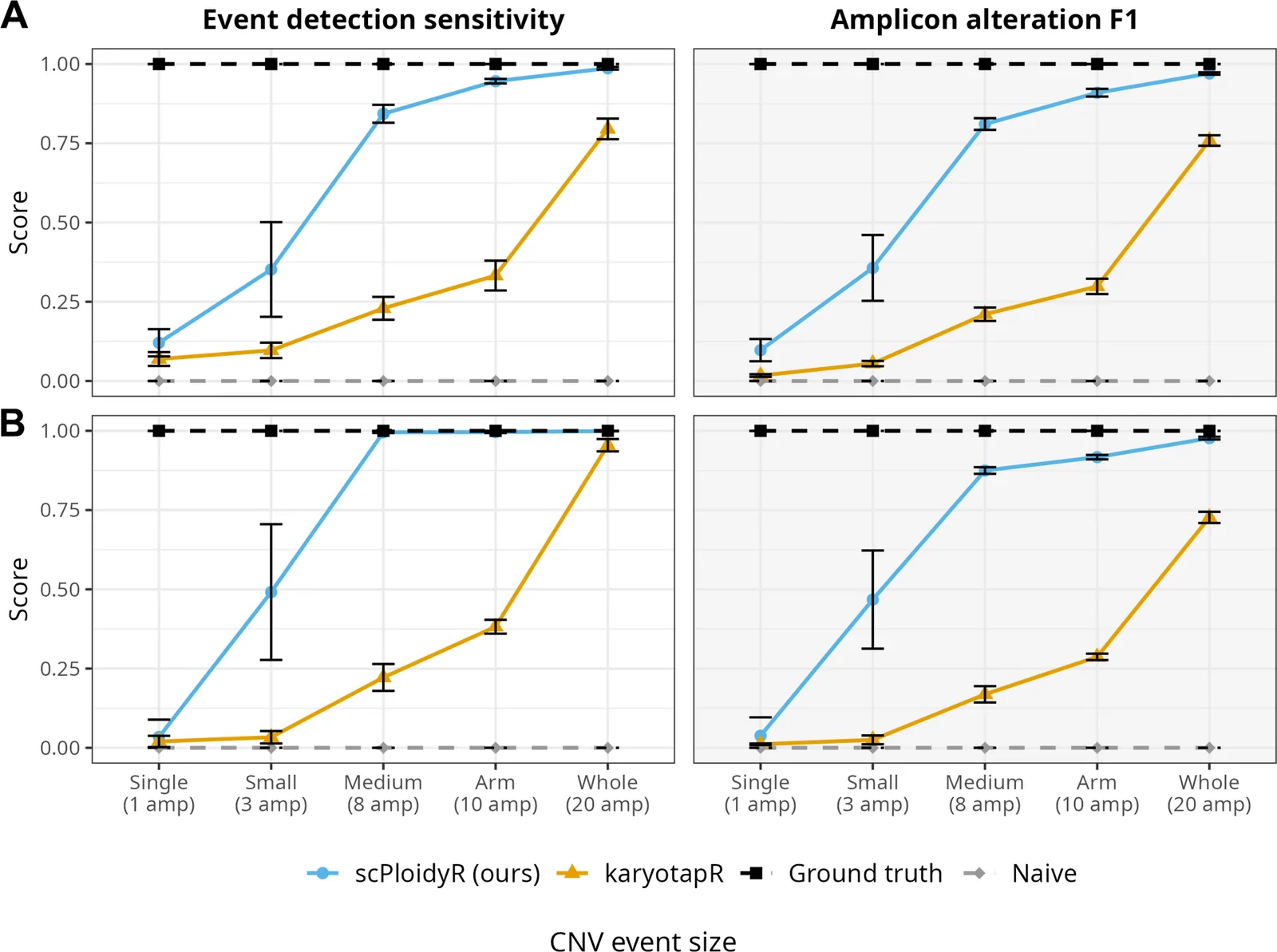
Simulation study 2: CNV event size and detection resolution. Copy number gains (A, top row) and losses (B, bottom row) are each scored by event-detection sensitivity (left column) and amplicon-level alteration F1 (right column) as a function of event size. Event size increases from single-amplicon to whole-chromosome along the *x* axis (single, 1 amplicon; small, 3 amplicons; medium, 8 amplicons; arm, 10 amplicons; whole chromosome, 20 amplicons). Lines represent scPloidyR (blue), karyotapR (GMM, orange) with its chromosome-level call broadcast to amplicons, the ground truth (black), and the naive diploid predictor (gray); error bars show SD across 5 replicates.

Detection improved monotonically with event size for both methods, but scPloidyR became reliable at a much smaller event size (Fig. 7). Single-amplicon events were near the detection floor for both methods (scPloidyR detection sensitivity 0.121 for gains and 0.034 for losses; karyotapR 0.069 and 0.020), defining the practical lower limit of resolution: A lone amplicon carries too little depth and allelic signal to separate from background noise. scPloidyR detection then rose steeply with size, to 0.352 (gain) and 0.491 (loss) at small focal events, 0.843 and 0.995 at medium focal events, 0.946 and 0.996 at arm-level events, and 0.987 and 0.999 at whole-chromosome events, so focal events of 8 or more amplicons and all larger events were recovered reliably. karyotapR, by contrast, became reliable only at the whole-chromosome scale (detection 0.795 for gains and 0.955 for losses) and remained poor through arm-level events (0.333 and 0.382), confirming that chromosome-level smoothing cannot localize subchromosome alterations. Throughout, scPloidyR retained high specificity in the surrounding diploid regions (at least 0.98 across all sizes), so its higher focal-event sensitivity did not come at the cost of false positives, whereas broadcasting the karyotapR call necessarily spread any chromosome-level alteration across the whole chromosome (specificity approximately 0.92). The amplicon-level alteration F1 followed the same ordering (Fig. 7, right column): scPloidyR rose from near zero for single-amplicon events (0.097 gain, 0.038 loss) to 0.811 and 0.875 at medium focal events and 0.970 and 0.977 at whole-chromosome events, whereas karyotapR stayed below 0.30 for every subchromosome event and reached only 0.759 (gain) and 0.727 (loss) even at whole-chromosome scale, because broadcasting a chromosome-level call inflates false positives across the unaltered amplicons of any focal event. These results characterize scPloidyR’s effective resolution: It resolves focal events down to a few amplicons and reliably detects multi-amplicon focal, arm-level, and whole-chromosome alterations, while single-amplicon events fall below its detection limit (Fig. 7). Complete per-size metric values for all methods, including the diploid-region specificity summarized here, are provided in File S10.

### Application to real data

**Fig. 8.**
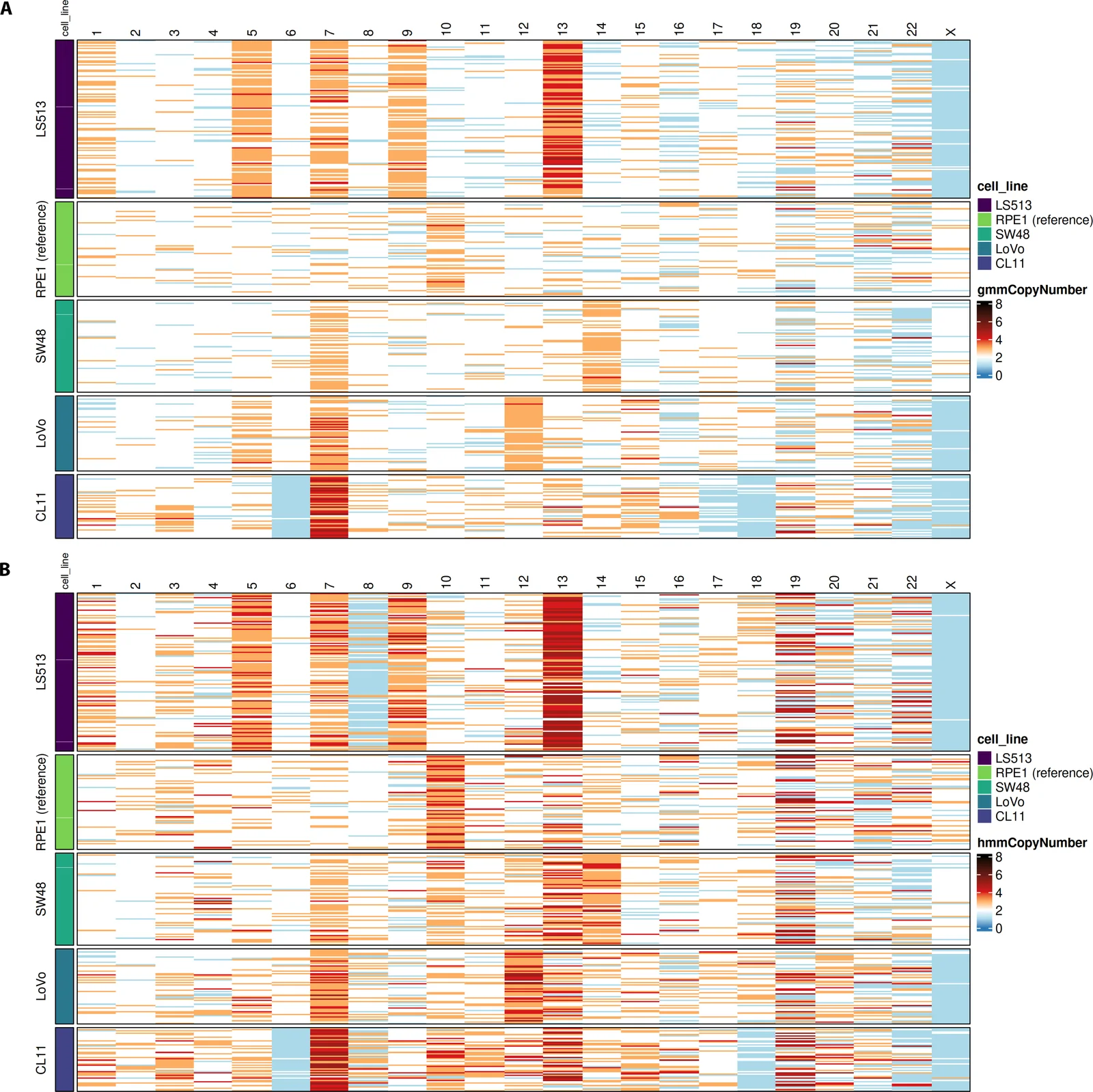
Single-cell copy number heatmaps from the Tapestri 5-cell-line mixture dataset. (A) karyotapR GMM copy number calls. (B) scPloidyR HMM copy number calls. Rows represent individual cells grouped and labeled by cell line (LS513, RPE1, SW48, LoVo, CL11; color bar, left); columns represent chromosomes 1 to 22 and X. Copy number states are encoded by a diverging color scale (blue: loss, white: diploid, orange/red: gain). Cell line 2 (RPE1) served as the near-diploid reference with known trisomy 10q; because it defines the reference baseline, it is shown for baseline context rather than as an independent result. The expected karyotype of each cell line is summarized in Table 1, against which the per-chromosome calls in each heatmap can be read directly.

We applied both methods to the public Tapestri cell mixture dataset (see Methods). Matched single-cell ground truth is unavailable; instead, the comparison focuses on concordance between methods, biological plausibility, and agreement with the published line-level karyotypes (Table 1), which were established by orthogonal assays and serve as an external reference. The karyotapR (GMM) and scPloidyR copy number heatmaps (Fig. 8A and B) provide direct qualitative comparisons across the 5 cell-line populations, which correspond to the 5 lines pooled in the source study: LS513 (cell line 1), RPE1 (cell line 2), SW48 (cell line 3), LoVo (cell line 4), and CL11 (cell line 5). We matched each cluster to its line by comparing its recovered copy number profile with the published karyotypes (Table 1) and label the heatmaps accordingly so that the per-chromosome calls of each line can be read directly against its expected gains and losses. RPE1 (cell line 2) is the near-diploid reference: It defines the diploid baseline, so its profile is shown for baseline context rather than as an independent result.

**Table 1.** Expected karyotypes of the 5 cell lines in the Tapestri mixture dataset [18]. Gains are denoted by + and losses by −; p/q denote chromosome arms. Whole-chromosome modal copy number calls reflect whole-chromosome gains and losses but do not resolve arm-restricted events such as +1q, +5p, or +10q.

Cell line	Published karyotype
RPE1 (cell line 2, reference)	+10q, XX
LS513 (cell line 1)	+1q, +5, +7, +9, +13, +13, XY
SW48 (cell line 3)	+7, +14, XX
LoVo (cell line 4)	+5p, +7, +12, XY
CL11 (cell line 5)	−6, +7, +7, −17, −18, −22, XY

Both methods recover concordant large-scale karyotypic structures across all 5 cell lines. LS513 (cell line 1) displays extensive gains on chromosomes 1, 5, 7, 9, and 13; the RPE1 population (cell line 2) is the reference, so its near-diploid profile and trisomy 10q are expected by construction (the baseline is learned from these cells and the trisomy is recorded in the reference copy number specification) rather than independently detected, and are shown only as an internal consistency check [18]; and SW48, LoVo, and CL11 (cell lines 3 to 5) each exhibit distinct but consistent alteration patterns in both heatmaps. The spatial distribution of gains (orange/red) and losses (blue) is broadly similar, confirming that both approaches detect the major CNV events in this dataset. Overall, the gains and losses recovered across the 4 nonreference cancer lines are consistent with their published karyotypes (Table 1): The chromosome 7 gain shared across these 4 lines, high-level (copy number 4 to 5) amplification reflecting the duplicated +7 and +13 genotypes, and chromosome 6, 17, and 18 losses are all recapitulated; because RPE1 defines the reference baseline, its profile is not counted as independent evidence here. As expected, whole-chromosome modal calls do not resolve arm-restricted events (+1q, +5p), which appear as partial rather than whole-chromosome signals in the heatmaps.

Despite this overall concordance, several qualitative differences are apparent. The clearest is on chromosome 19, which stands out as one of the most divergent regions between the 2 methods: scPloidyR makes scattered gain calls there, whereas karyotapR calls chromosome 19 as a gain far less often. We examined these calls directly and interpret them as low-confidence over-calls rather than true events. The strongest evidence is in the near-diploid reference line (RPE1), which is diploid on every chromosome except its known chromosome 10 trisomy, so any chromosome 19 gain in these cells is a false positive by construction: scPloidyR calls chromosome 19 as a gain in 45% of RPE1 cells, against 22% for karyotapR. We therefore treat chromosome 19 as low-confidence for scPloidyR in this panel and note that such false positives could be mitigated at the amplicon-design stage in future panels. Second, for the X chromosome in cell line 1, karyotapR assigns noticeably more heterogeneous copy number states across cells, while scPloidyR yields more uniform calls within this population. This difference may reflect a consistent copy number call driven by the BAF signal.

#### Runtime and memory

We benchmarked the computational cost of both methods on the application dataset (3,020 cells, 319 amplicons; see Methods), scaling the number of cells from 1,000 to 8,000 by subsampling below the dataset size and oversampling with replacement above it while holding the reference set fixed. karyotapR’s GMM is single-threaded but fast: Copy number calling took 9.8 s of wall-clock time at 1,000 cells and 19.1 s at 8,000 cells [14.2 s and 23.4 s total CPU (central processing unit), respectively]. scPloidyR, which fits a per-chromosome HMM with joint depth-BAF emissions and per-cell expectation-maximization (EM), was much more compute-intensive: Using 8 cores, it required 122.1 s of wall-clock time at 1,000 cells and 894.6 s at 8,000 cells (957.9 s and 7,058 s total CPU), corresponding to a roughly constant 0.11 s per cell. At 8,000 cells, scPloidyR was therefore about 47-fold slower in wall-clock time and about 300-fold more CPU-intensive than karyotapR, the cost of jointly modeling allelic information and enforcing spatial coherence. Because scPloidyR fits each cell independently, its workload is fully parallel and its wall-clock runtime scaled approximately linearly with the number of cells (122.1 s at 1,000, 219.5 s at 2,000, 456.7 s at 4,000, and 894.6 s at 8,000; Table 2). Peak resident memory was modest in absolute terms for both methods: scPloidyR remained near 1.0 GB across the entire range (956 to 1,020 MB), while karyotapR’s footprint grew with cell number (from approximately 1.1 GB at 1,000 cells to 1.6 GB at 8,000 cells) so that at large cell counts scPloidyR used somewhat less memory than karyotapR. In practice, the per-cell HMM cost is small and constant, so larger datasets are handled by adding cores, while memory grows only slowly; the main consideration for scaling is the total CPU budget rather than memory.

**Table 2.** Runtime and peak-memory scaling of karyotapR (GMM, single core) and scPloidyR (HMM, 8 cores) on the Tapestri 5-cell-line dataset, measured at 1,000, 2,000, 4,000, and 8,000 cells (subsampled below and oversampled with replacement above the 3,020-cell dataset, with the reference set held fixed), for the core copy number calling step. Total CPU is user + system time aggregated across all worker processes.

Cells	Method	Cores	Wall (s)	Total CPU (s)	Peak memory (MB)
1,000	karyotapR	1	9.8	14.2	1,141
1,000	scPloidyR	8	122.1	957.9	956
2,000	karyotapR	1	11.1	15.3	978
2,000	scPloidyR	8	219.5	1,735.0	958
4,000	karyotapR	1	14.5	18.6	1,349
4,000	scPloidyR	8	456.7	3,609.2	970
8,000	karyotapR	1	19.1	23.4	1,599
8,000	scPloidyR	8	894.6	7,057.8	1,020

### Summary of comparative performance

Across both simulation study 1 and simulation study 2, scPloidyR generally improves class-balanced performance and alteration detection relative to karyotapR (macro-F1 0.477 versus 0.273; alteration F1 0.903 versus 0.381 in simulation study 1; Fig. 1B and D), while the naive diploid predictor achieves misleadingly elevated accuracy but fails to detect CNVs. However, when BAF information is unavailable, either due to zero heterozygous variants per amplicon or 0% heterozygosity, karyotapR outperforms scPloidyR. Overall, the results indicate that joint modeling of depth and BAF is advantageous for single-cell CNV calling in heterogeneous populations but that depth-only methods remain preferable when allelic information is absent.

## Discussion

This study demonstrates that jointly modeling read depth and BAF within an HMM framework markedly improves single-cell copy number calling from targeted DNA sequencing panels. Across both simulation studies, scPloidyR outperformed the depth-only karyotapR GMM on macro-F1, balanced accuracy, and alteration F1 whenever allelic information was available; only in the 2 conditions that remove that information entirely, zero variants per amplicon and 0% heterozygosity, did karyotapR lead on macro-F1 and balanced accuracy, and even there scPloidyR retained the higher alteration F1. Application to a real Tapestri cell mixture dataset confirmed that the joint model produces more spatially coherent copy number profiles.

The primary advantage of scPloidyR lies in exploiting allelic information invisible to depth-only methods. BAF distinguishes copy number states that produce similar total read counts, such as different trisomy genotypes that share the same depth but yield distinct BAF values [21,26]. The variant density experiment directly demonstrates this: Adding just one heterozygous variant per amplicon increased scPloidyR accuracy from 0.556 to 0.897 for gains, showing that even sparse allelic information carries strong discriminative power. The per-chromosome HMM structure further contributes by enforcing spatial coherence, smoothing isolated noisy observations toward the local consensus state and reducing false positive calls in regions without true alterations.

However, scPloidyR is heavily dependent on BAF availability and quality. When heterozygous variants are absent, scPloidyR loses its allelic signal and underperforms karyotapR. The BAF noise experiment revealed a second, equally important vulnerability: scPloidyR’s advantage collapses once allelic measurements become noisy. As noise increased from SD = 9 to SD = 12, scPloidyR accuracy for gains fell sharply from 0.945 to 0.788 and its balanced accuracy from 0.871 to 0.723, while karyotapR remained stable; past this inflection, the joint model’s advantage over depth alone narrows sharply, and depth-only calling becomes the safer default. The quality, not merely the presence, of allelic information therefore governs whether joint modeling helps, so users should estimate BAF noise before applying scPloidyR. This estimate is direct: BAF noise is the dispersion of heterozygous-site minor allele frequency (MAF) around its expected value of 0.5, which is the BAF emission standard deviation (SD) $\sigma_{b}$ that scPloidyR estimates from the reference population and refines by EM [Eq. (18b)]. Reading $\sigma_{b}$ from a fitted model, or equivalently measuring the spread of MAF at known-heterozygous reference sites, places a dataset directly on the degradation curve of Fig. 2; the simulated noise SD is the same quantity on a 0 to 100 scale, so SD = 9 and SD = 12 correspond to $\sigma_{b}=0.09$ and $\sigma_{b}=0.12$. Values up to about $\sigma_{b}=0.09$ lie in scPloidyR’s favorable regime, whereas values approaching $\sigma_{b}=0.12$ fall past the inflection where it degrades. As a practical guideline, scPloidyR should be preferred when at least one heterozygous variant per amplicon is available and the estimated BAF noise is moderate ($\sigma_{b}$ below roughly 0.09); karyotapR is the safer choice when allelic information is sparse or this estimated noise is high. Like karyotapR and reference-based copy number callers generally, scPloidyR calls copy number relative to a reference population of known ploidy, from which the per-amplicon baseline, depth variance, and heterozygosity rates are estimated [Eqs. (10), (11), and (13); see the Reference parameter learning and EM section in Methods); the reference need not be diploid, since nondiploid ploidy can be entered in the reference copy number specification (used here for the RPE1 trisomy 10q).

A consistent pattern across simulation study 1 is that performance is strongest for low-order copy number changes and degrades for high-level amplifications, indicating that the framework is primarily optimized for distinguishing low-order alterations (CN 1 to CN 3). scPloidyR resolved single-copy deletions (CN 1, sensitivity 1.000), the diploid state (CN 2, 0.983), and single-copy gains (CN 3, 0.382) far more reliably than higher-ploidy states, where sensitivity fell to 0.208 at CN 4 and 0.052 at CN 5 (Fig. 1E to I). At CN 4, both methods perform poorly and scPloidyR remains marginally ahead of karyotapR (0.208 versus 0.171), whereas at CN 5 karyotapR retains the higher sensitivity (0.387 versus 0.052). Two assay-level factors make high-level amplifications intrinsically difficult to call from targeted panels. First, the depth signal compresses at high ploidy: The relative depth step between adjacent states shrinks from a 2-fold change between CN 1 and CN 2 to a 1.25-fold change between CN 4 and CN 5, so additive measurement noise increasingly overwhelms the true difference. Second, the allelic signal becomes less distinctive, because balanced high-ploidy genotypes such as AABB at CN 4 produce BAF values near 0.5 that resemble the diploid state, and the BAF separations between successive high-ploidy genotypes narrow. Examining the misclassification structure directly shows how these 2 factors play out for scPloidyR: Rather than being graded one state downward, high-ploidy gains collapse predominantly to the single-copy deletion state, with 49% of true CN 5 cells and 44% of true CN 4 cells called CN 1 (the same depth-anchoring collapse is already visible at CN 3). Once the compressed depth signal and the near-diploid balanced BAF make a high gain hard to separate from baseline, the per-chromosome model anchors the cell to the lowest-depth state. The depth-only karyotapR GMM, which lacks this allelic anchoring, instead degrades one step downward (true CN 5 distributed mainly across CN 3, CN 4, and CN 5) and so preserves the gain direction, which is why it retains the higher CN 5 sensitivity (0.387 versus 0.052). We therefore recommend interpreting calls of CN 4 or higher categorically, as evidence of an alteration rather than as an exact integer copy number, and at the highest ploidy even the direction of the change should be treated with caution. This limitation is further tempered by how copy number alterations are typically distributed in cancer, where the large majority are low-order single-copy gains and losses and arm- or chromosome-level aneuploidies (gains or losses of a single copy) rather than high-level focal amplifications [1,3,27], so scPloidyR’s strongest regime (CN 1 to CN 3) coincides with the most common class of alteration. High-level amplifications, although comparatively infrequent, remain biologically and clinically important and are precisely the events for which we recommend depth-based calibration or orthogonal validation. Altered-versus-diploid detection nonetheless remains reliable in this regime (alteration F1 0.903), because these collapsed high-ploidy calls are still correctly flagged as nondiploid even when their exact state or direction is uncertain; when precise high-level amplification counts are the primary objective, depth-based calibration or orthogonal validation is advisable.

Several further limitations should be noted. Although our event-size analysis now spans alterations from single amplicons to whole chromosomes and shows that scPloidyR resolves focal events down to a few amplicons, while single-amplicon events fall below its detection limit (Fig. 7), the simulation framework otherwise employed simplified ground truth designs with uniform copy number states within each event; real tumors exhibit more complex patterns including subclonal alterations and intermixed focal events [1,6] that may affect performance differently. Additionally, the application study lacked matched single-cell ground truth copy number profiles; the published line-level karyotypes provide an external reference for qualitative concordance but not the per-cell resolution needed for quantitative accuracy. Sex chromosomes are handled only through the depth-based reference_cn template: Because a hemizygous chromosome admits no heterozygous genotypes, the allelic channel is uninformative and such calls rest on depth alone, and because a single reference ploidy cannot describe a mixed-sex cohort, sex-aware modeling with per-cell sex inference is left to future work. The performance of CNV callers also depends strongly on the underlying data type and detection strategy, highlighting the importance of systematic, platform-specific benchmarking when selecting a method [28].

scPloidyR also occupies a distinct niche within the broader single-cell CNV-calling landscape, which differs from it at the level of the underlying assay. A family of methods infers allele-specific or high-resolution copy number from single-cell or low-coverage whole-genome DNA sequencing, including CHISEL [26], SCOPE [29], SEACON [30], and HiScanner [31]. These tools rely on near-contiguous, genome-wide coverage to bin the genome into many consecutive windows, segment across breakpoints, and, in the allele-specific methods, phase haplotypes across the genome. Targeted Tapestri panels instead sample only sparse, noncontiguous amplicons, so genome-wide binning, cross-bin segmentation, and genome-wide haplotype phasing are unavailable, and these methods are not directly applicable to amplicon data. A second family infers copy number from single-cell RNA sequencing, including Numbat [23] and CaSpER [32]; these methods likewise exploit allelic information, but they infer copy number indirectly from gene expression and derive allele frequencies only from heterozygous variants in expressed transcripts, making their inference sensitive to expression variation, transcriptional bursting, and dropout. scPloidyR instead measures DNA dosage and BAF directly from targeted DNA amplicons at panel-defined resolution. Because these approaches operate on fundamentally different data types, they cannot be benchmarked head-to-head on a shared dataset and are best regarded as complementary, with scPloidyR addressing the targeted single-cell DNA setting that whole-genome and RNA-based callers do not cover.

Despite these limitations, our results show that joint depth-BAF modeling via scPloidyR improves on depth-only approaches for single-cell CNV calling when allelic information is available. By combining the discriminative power of BAF with the spatial smoothing of a per-chromosome HMM, scPloidyR provides a practical framework for copy number inference from targeted single-cell DNA sequencing panels. Building on this framework, a natural next step is to integrate its copy number profiles with complementary modalities such as DNA methylation, consistent with broader integrative and machine-learning approaches to single-cell analysis [33–35].

## Methods

### Data normalization and reference specification

Normalized read counts are computed using Mission Bio’s mb scheme. Let $d_{i,c}$ denote the raw read count for amplicon i in cell c (c=1,…,C, where C is the total number of cells), and A the number of amplicons. First, “good barcodes” are defined as cells with total counts exceeding 10% of $S_{\left(11\right)}$, the 11th-largest total count across all cells:

$$B=\left\{c:\sum_{i=1}^{A}d_{i,c}>\frac{1}{10}\cdot S_{\left(11\right)}\right\}$$ (1)

Second, counts are adjusted for cell depth by dividing by the cell mean (plus a stabilizing constant):

$${\tilde{d}}_{i,c}=\frac{d_{i,c}}{{\bar{d}}_{c}+1}$$ (2)

where ${\bar{d}}_{c}=\frac{1}{A}\sum_{j=1}^{A}d_{j,c}$. Third, amplicon-level normalization uses medians across good barcodes and scales to the diploid baseline:

$$y_{i,c}=\frac{{\tilde{d}}_{i,c}}{m_{i}+0.05}\times 2$$ (3)

where $m_{i}=\text{median}\left\{{\tilde{d}}_{i,c\prime }:c\prime \in B\right\}$. Reference cells are cell clusters defined by users with known ploidy (typically diploid) that define the baseline copy number profile. karyotapR accepts a control.copy.number template; scPloidyR uses reference_cn. Nondiploid regions in reference cells (for example, trisomy) are explicitly encoded in these templates. Sex chromosomes are accommodated through the same reference_cn template rather than a dedicated model: A user specifies the expected sex-chromosome ploidy of the reference (for example, one copy of X and Y for a male reference), and changes are then called against that baseline.

### Copy number inference models

#### karyotapR (GMM)

For each probe i, a Weibull distribution is fitted to normalized counts in reference cells. The Weibull distribution was selected because it provides a good fit for the observed distribution of normalized amplicon counts [18]. Synthetic cells are generated for each copy number state k by scaling the Weibull scale parameter:

$$d_{i}^{\left(k\right)}∼\text{Weibull}\left(\alpha_{i},\lambda_{i}\cdot \frac{k}{{\mathrm{CN}}_{\mathrm{ref}}}\right)$$ (4)

with shape $\alpha_{i}$ and scale $\lambda_{i}$ estimated per probe and ${\mathrm{CN}}_{\mathrm{ref}}$ typically 2. Because each draw from Eq. 4 represents one synthetic cell rather than an observed cell, the cell index c is implicit. Probe-level values are summarized to segments (chromosome arms or whole chromosomes) using the median. Gaussian components $N\left(\mu_{k},\sigma_{k}^{2}\right)$ are fitted to each copy number class, and real cells are classified by posterior probability under equal priors:

$$\hat{k}=\arg\max_{k}\frac{N\left(y|\mu_{k},\sigma_{k}^{2}\right)}{\sum_{j=1}^{K}N\left(y|\mu_{j},\sigma_{j}^{2}\right)}$$ (5)

where y is the segment-level copy number value.

#### scPloidyR

scPloidyR models the ordered amplicons along each chromosome as emissions from a Markov chain with hidden copy number states $z=\left(z_{1},\ldots ,z_{n}\right)$, where $z_{i}\in \left\{1,2,\ldots ,K\right\}$ with K=5. Transitions are governed by a persistence matrix with rare breakpoints:

$$T_{\mathrm{jk}}=P\left(z_{i}=k|z_{i-1}=j\right)=\left\{\begin{array}{ll} 1-\epsilon & \text{if}\;j=k\\ \frac{\epsilon }{K-1} & \text{if}\;j\neq k \end{array}\right.$$ (6)

with ϵ initialized at $10^{-3}$. The emission probability factorizes into depth and BAF likelihoods, where $y_{i}=\left(d_{i}^{\mathrm{norm}},b_{i}\right)$ is the observation vector at amplicon i, consisting of the normalized read depth $d_{i}^{\mathrm{norm}}$ and the vector of MAFs $b_{i}$:

$$P\left(y_{i}|z_{i}=k\right)=P\left(d_{i}^{\mathrm{norm}}|z_{i}=k\right)\cdot P\left(b_{i}|z_{i}=k\right)$$ (7)

Depth is modeled as Gaussian on the normalized scale with state-dependent means:

$$P\left(d_{i}^{\mathrm{norm}}|z_{i}=k\right)=N\left(d_{i}^{\mathrm{norm}}|\mu_{k},\sigma_{d}^{2}\right),\;\mu_{k}=\frac{k}{2}$$ (8–9)

where $\sigma_{d}^{2}$ is the variance of normalized depth across amplicons (subscript d for depth). Input depths $d_{i}^{\mathrm{raw}}$ (Mission Bio’s mb scheme-normalized counts from Eq. 3) are normalized by per-amplicon baselines learned from reference cells:

$$d_{i}^{\mathrm{norm}}=\frac{d_{i}^{\mathrm{raw}}}{\beta_{i}},\;\beta_{i}={\text{median}}_{c\in R}\left(d_{i,c}^{\mathrm{raw}}\right)\times \frac{2}{{\mathrm{CN}}_{\mathrm{ref},i}}$$ (10–11)

where R are reference cells and ${\mathrm{CN}}_{\mathrm{ref},i}$ permits nondiploid reference regions.

For allele frequency, each amplicon i provides $V_{i}$ variants. Raw BAFs are converted to MAFs via $b=\min\left(\mathrm{BAF},1-\mathrm{BAF}\right)$, yielding observed values $b_{i}=\left(b_{i,1},\ldots ,b_{i,V_{i}}\right)$ on $\left[0,0.5\right]$. The likelihood marginalizes over genotypes compatible with copy number state k, where $G_{k}$ is the set of possible genotypes for copy number state k:

$$P\left(b_{i}|z_{i}=k\right)=\sum_{g\in G_{k}}P\left(g|k,i\right)\prod_{v=1}^{V_{i}}P\left(b_{i,v}|g\right)$$ (12)

Genotypes are all combinations of k alleles (A or B). The expected MAF for a genotype with m minor allele copies is $\min\left(m,k-m\right)/k$, yielding representative values of 0 (homozygous), 1/3 for k=3 heterozygotes, 1/4 and 1/2 for k=4 heterozygotes, and analogous values for general k. Genotype priors are anchored by the per-amplicon heterozygosity rate $h_{i}$ estimated from reference cells; for diploid loci:

$$P\left(\mathrm{AB}\right)=h_{i},\;P\left(\mathrm{AA}\right)=P\left(\mathrm{BB}\right)=\frac{1-h_{i}}{2}$$ (13)

with higher copy number priors interpolated between homozygous and heterozygous templates. Each MAF observation is modeled by a truncated normal on $\left[0,0.5\right]$:

$$P\left(b|g\right)=\frac{\frac{1}{\sigma_{b}}\phi \left(\frac{b-\mu_{g}}{\sigma_{b}}\right)}{\Phi \left(\frac{0.5-\mu_{g}}{\sigma_{b}}\right)-\Phi \left(\frac{0-\mu_{g}}{\sigma_{b}}\right)}$$ (14)

where $\mu_{g}$ is the expected MAF for genotype g.

#### Reference parameter learning and EM

scPloidyR learns per-amplicon depth baselines $\beta_{i}$, depth variance $\sigma_{d}^{2}$, BAF SD $\sigma_{b}$, and heterozygosity rates $h_{i}$ from reference cells. Model parameters $\theta =\left\{\mu ,\sigma_{d}^{2},\sigma_{b},\epsilon \right\}$ are then estimated using Baum–Welch with forward–backward posteriors

$$\gamma_{i}\left(k\right)=P\left(z_{i}=k|Y,\theta \right),\;\xi_{i}\left(j,k\right)=P\left(z_{i-1}=j,z_{i}=k|Y,\theta \right)$$ (15–16)

where Y denotes the complete set of observations across all amplicons, and standard M-step updates for $\mu_{k}$, $\sigma_{d}^{2}$, $\sigma_{b}$, and ϵ:

$$\mu_{k}^{\mathrm{new}}=\frac{\sum_{i=1}^{n}\gamma_{i}\left(k\right)d_{i}^{\mathrm{norm}}}{\sum_{i=1}^{n}\gamma_{i}\left(k\right)}$$ (17)

$${\left(\sigma_{d}^{2}\right)}^{\mathrm{new}}=\frac{\sum_{i=1}^{n}\sum_{k=1}^{K}\gamma_{i}\left(k\right){\left(d_{i}^{\mathrm{norm}}-\mu_{k}\right)}^{2}}{n_{d}}$$ (18a)

where $n_{d}$ is the number of amplicons with observed depth, and

$${\left(\sigma_{b}\right)}^{\mathrm{new}}=\sqrt{\frac{\sum_{i=1}^{n}\sum_{k:s_{k}\geq 1}\gamma_{i}\left(k\right)\sum_{v=1}^{V_{i}}{\left(b_{i,v}-{\bar{\mu }}_{k,i}\right)}^{2}}{\sum_{i=1}^{n}\sum_{k:s_{k}\geq 1}\gamma_{i}\left(k\right)\;V_{i}}}$$ (18b)

where ${\bar{\mu }}_{k,i}=\sum_{g\in G_{k}}P\left(g|k,i\right)\;\mu_{g}$ is the prior-weighted expected MAF for state k at amplicon i, and the sums are restricted to informative states ($s_{k}\geq 1$) with observed variants ($V_{i}>0$).

$$\epsilon^{\mathrm{new}}=\frac{\sum_{i=2}^{n}\sum_{j\neq k}\xi_{i}\left(j,k\right)}{n-1}$$ (19)

When heterozygosity is low (mean heterozygosity $\bar{h}=\frac{1}{n}\sum_{i}h_{i}<0.25$) , depth means can collapse. We therefore regularize toward prior means $\mu_{k}^{\text{prior}}=k/2$ using a weight w that increases as h decreases:

$$\mu_{k}^{\mathrm{new}}=\frac{\sum_{i}\gamma_{i}\left(k\right)d_{i}^{\mathrm{norm}}+w\mu_{k}^{\text{prior}}}{\sum_{i}\gamma_{i}\left(k\right)+w}$$ (20)

EM iterates until parameter change is $<10^{-5}$ or 50 iterations. Final copy number paths are obtained by Viterbi decoding:

$$\hat{z}=\arg\max_{z}P\left(z|Y,\hat{\theta }\right)$$ (21)

To enforce chromosome boundaries, scPloidyR fits independent chains per chromosome and concatenates per-chromosome Viterbi paths. Amplicon-level calls are aggregated to segments using the mode (scPloidyR) or median (karyotapR):

$${\mathrm{CN}}_{\mathrm{seg}}=\text{mode}\left(\left\{z_{i}:i\in \text{segment}\right\}\right)$$ (22)

scPloidyR does not consume the raw Mission Bio Tapestri HDF5 output directly; it operates on preprocessed per-cell matrices (mb-normalized amplicon depths, per-amplicon BAF values, amplicon-to-chromosome labels, reference-cell indices, and a reference_cn template) produced by the standard karyotapR preprocessing pipeline. It returns copy number calls at per-amplicon and per-cell resolution, which are aggregated to per-segment (chromosome or arm) and per-clone (cell population) profiles. A complete specification of required inputs, output format, and the full table of default parameters (copy number states, transition probability, depth- and BAF-variance initialization, heterozygosity and filtering thresholds, and EM convergence criteria) is provided in File S8.

#### Model assumptions and robustness

The Gaussian depth and truncated-normal BAF emissions are working approximations chosen for stability under EM rather than exact descriptions of the count process. Because the depth emission operates on normalized depth (Eq. 10, the ratio to the per-amplicon reference baseline), it divides out the dominant amplicon-specific bias such as amplification efficiency and GC- and length-dependent effects, leaving an approximately symmetric residual for which a Gaussian is a reasonable central approximation. The model absorbs residual overdispersion and heavier tails through the EM-learned depth variance (Eq. 18a), per-chromosome HMM smoothing (Eq. 6), and mode aggregation across amplicons (Eq. 22); the existing simulation studies, which draw depth from a skewed, overdispersed Weibull rather than a Gaussian, retain high accuracy (Fig. 1), confirming robustness to this misspecification. The truncated normal on $\left[0,0.5\right]$ (Eq. 14) suits the bounded MAF, and the per-genotype mixture (Eq. 12) accommodates allelic noise and partial dropout without an explicit dropout term. Amplicons with no recovered variants reduce to the depth-only emission (Eq. 7); the same fallback, together with depth-anchoring under low heterozygosity (Eq. 20) and removal of probes whose reference baseline is at or below 0.05, handles missing BAF, low-depth variants, and low-coverage probes.

#### Ablation analysis

To quantify how much each component of the model contributes, we evaluated 3 ablated variants against the full model and the depth-only GMM on the simulation study 1 benchmark: a depth-only HMM (BAF emission removed), a BAF-only HMM (depth emission removed), and a joint non-HMM decode (the transition matrix held uniform, so decoding reduces to independent per-amplicon classification). The variants are produced by toggling emission and transition terms on the single production code path, isolating the model from preprocessing and aggregation differences; full results are reported in File S9.

### Simulation study

We evaluated methods on synthetic data with known ground truth (i.e., known copy number assignments) in 2 simulation studies. Simulation study 1 used a balanced multi-state design with 6 cell populations of equal size: copy number (CN) = 1 (monosomy), CN = 2 (disomy/diploid, reference), CN = 2 (disomy, mixed genotypes), CN = 3 (trisomy), CN = 4 (tetrasomy), and CN = 5 (pentasomy). Thirty percent of chromosomes (7 of 22) were randomly altered in nonreference groups across 22 chromosomes with 4 amplicons per chromosome, 3 variants per amplicon, BAF noise (the SD of Gaussian noise added to simulated allele frequencies) of 9, and 100% heterozygosity. Ten independent replicates were run with parameter jitter (cells ± 10%, BAF noise ± 15%). Simulation study 2 used a multi-cell line design with 2 populations (a diploid reference and one altered cell line) and 30% of chromosomes altered. Five parameters were varied one at a time (BAF noise, variants per amplicon, amplicons per chromosome, cells per group, and heterozygosity rate), each tested for both gains (CN=3) and losses (CN = 1), with 3 independent replicates per condition. Baseline values were BAF SD = 9, 3 variants per amplicon, 4 amplicons per chromosome, 100 cells per group, and 100% heterozygosity. Table 3 summarizes the ground truth design for both simulation studies.

**Table 3.** (A) Simulation study 1: Synthetic cell populations. Ten replicates with parameter jitter (cells ± 10%, BAF noise ± 15%). (B) Simulation study 2 (copy number gain): Synthetic cell populations. (C) Simulation study 2 (copy number loss): Synthetic cell populations. CN = 2 (reference) cells are diploid across all chromosomes and serve as the reference for normalization. CN = 2 (mixed) cells are also diploid but carry heterogeneous genotype configurations to test genotype-driven BAF variation without copy number changes.

Population	CN	Cells	Chr. altered	Replicates
(A)
CN=1	1	80	7/22	10
CN=2 (reference)	2	80	0/22	10
CN=2 (mixed)	2	80	0/22	10
CN=3	3	80	7/22	10
CN=4	4	80	7/22	10
CN=5	5	80	7/22	10
(B)
Diploid reference	2	100	0/22	3
Altered (gain)	3	100	7/22	3
(C)
Diploid reference	2	100	0/22	3
Altered (loss)	1	100	7/22	3

Study 2 varied parameters one at a time: BAF SD (6, 9, 12), variants/amplicon (0, 1, 3, 5), amplicons/chr (2, 4, 6), cells/group (50, 100, 200), heterozygosity rate (0%, 25%, 50%, 75%, 100%). Baseline values: 4 amplicons/chr, 3 variants/amplicon, BAF SD = 9, 100 cells/group, 100% heterozygosity. Table 3B and C shows baseline values; each varied parameter replaced one baseline value at a time (3 replicates per condition). A complete summary of simulation parameters and conditions is provided in File S1.

#### Read count simulation

For amplicon i and cell c, counts were sampled from a Weibull distribution with mean proportional to copy number:

$$d_{i,c}∼\text{Weibull}\left(k_{i},\lambda_{i}\cdot \frac{{\mathrm{CN}}_{c,\mathrm{chr}\left(i\right)}}{2}\right)$$ (23)

with $k_{i}∼\left|N\left(2,0.8^{2}\right)\right|+0.3$. The scale parameter $\lambda_{i}$ was constructed to be inversely related to $k_{i}$ and normalized to $\left[0.5,2.5\right]$, reproducing heterogeneous amplicon capture efficiencies observed in Tapestri data. Parameters $\left(k_{i},\lambda_{i}\right)$ were shared across cell lines to preserve realistic amplicon-specific biases.

#### BAF simulation

For each variant, a genotype was assigned according to the chromosome genotype and heterozygosity rate, the expected MAF was computed, and Gaussian noise with SD equal to the BAF noise parameter (SD) was added, with truncation to $\left[0,1\right]$. The number of variants per amplicon was sampled from user-defined options (with a default of 3 variants per amplicon in the baseline condition).

#### Univariate analyses

Simulation study 2 experiments used varied BAF noise (SD 6, 9, 12), variants per amplicon (0, 1, 3, 5), amplicons per chromosome (2, 4, 6), cells per group (50, 100, 200), and heterozygosity rate (0%, 25%, 50%, 75%, 100%). Each combination was tested for both gains (CN = 3) and losses (CN = 1), with 3 independent replicates.

#### Event-size simulation

To evaluate detection as a function of copy number event size, we extended the simulator to alter a contiguous run of amplicons within a chromosome rather than the whole chromosome. This required generating ground truth, read depth, and BAF at amplicon resolution: Each amplicon receives its own copy number multiplier ${\mathrm{CN}}_{i}/2$ in Eq. (23), and the genotype driving its BAF is assigned per amplicon, so an alteration shifts depth and allele frequency only within the event, while the remainder of the chromosome stays diploid. We used a 2-population design (diploid reference and one altered line, 100 cells each) on a higher-density panel of 10 chromosomes with 20 amplicons per chromosome (so one arm spans 10 amplicons), with baseline BAF noise (SD = 9), 3 variants per amplicon, and 100% heterozygosity. Five event sizes were simulated, single-amplicon (1), small focal (3), medium focal (8), chromosome-arm (10 amplicons aligned to a p/q boundary), and whole-chromosome (20), each for gains (CN = 3) and losses (CN = 1) with 5 independent replicates. Detection was scored at amplicon resolution against the amplicon-level ground truth; for the chromosome-level karyotapR (GMM) caller, the per-chromosome call was broadcast to its amplicons for a like-for-like comparison. Detection within the event was quantified by directional event detection sensitivity [Eq. (31)], and control of false positives in the surrounding diploid amplicons by amplicon-level alteration F1 and specificity [Eqs. (29) and (30)]; we use these amplicon-resolution metrics rather than the chromosome-level accuracy and macro-F1 of Simulation study 1 for the reasons given under Evaluation metrics.

#### Evaluation metrics

Performance was assessed using accuracy

$$\text{Accuracy}=\frac{1}{C\cdot N}\sum_{c,i}1\left[{\hat{z}}_{c,i}=g_{c,i}\right]$$ (24)

where N is the number of segments and $1\left[\cdot \right]$ denotes the indicator function, macro-F1

$$\text{Macro}-F1=\frac{1}{K}\sum_{k=1}^{K}F1_{k}$$ (25)

balanced accuracy

$$\text{Balanced}\;\mathrm{Acc}=\frac{1}{K}\sum_{k=1}^{K}\frac{TP_{k}}{TP_{k}+FN_{k}}$$ (26)

and per-class sensitivity

$${\text{Sensitivity}}_{k}=\frac{TP_{k}}{TP_{k}+FN_{k}}$$ (27)

We additionally evaluated binary detection of any CNV (CN ≠2), using a naive diploid predictor (always CN = 2) as a performance floor. Letting TP and FN denote true positives and false negatives under the binary classification where “positive” is any alteration (CN ≠2) and “negative” is diploid (CN = 2). Because this is a binary classification (altered versus diploid), class-specific subscripts are not needed. Alteration sensitivity is

$$\text{Alteration}\;s\text{ensitivity}=\frac{\mathrm{TP}}{\mathrm{TP}+\mathrm{FN}}$$ (28)

and alteration F1 combines sensitivity with alteration precision ${\text{Precision}}_{\mathrm{alt}}=\mathrm{TP}/\left(\mathrm{TP}+\mathrm{FP}\right)$:

$$\text{Alteration}\;F1=\frac{2\cdot {\text{Precision}}_{\mathrm{alt}}\cdot {\text{Sensitivity}}_{\mathrm{alt}}}{{\text{Precision}}_{\mathrm{alt}}+{\text{Sensitivity}}_{\mathrm{alt}}}$$ (29)

For the event-size analysis described below, which scores focal alterations at amplicon resolution, we use 2 further amplicon-level metrics. Alteration specificity measures correct rejection of the diploid background, where TN and FP are true negatives and false positives under the same altered-versus-diploid classification,

$$\text{Alteration}\;s\text{pecificity}=\frac{\mathrm{TN}}{\mathrm{TN}+\mathrm{FP}}$$ (30)

and event detection sensitivity measures localization within the event itself. Restricting to the set of truly altered amplicon–cell entries $E=\left\{\left(i,c\right):g_{i,c}\neq 2\right\}$, it is the fraction called in the correct direction (${\hat{z}}_{i,c}>2$ for gains, ${\hat{z}}_{i,c}<2$ for losses),

$$\begin{array}{c} \text{Detection}\;s\text{ensitivity}=\\ \frac{1}{\left|E\right|}\sum_{\left(i,c\right)\in E}1\;\left[{\hat{z}}_{i,c}\;\text{altered in the true direction}\right] \end{array}$$ (31)

Each metric captures a distinct facet of copy number calling performance. Overall accuracy provides a straightforward measure of correctness but can be misleading when diploid segments dominate the profile, inflating scores for methods that default to CN = 2. Macro-F1 addresses this class imbalance by averaging F1 scores equally across all CN states, ensuring that rare but clinically important states such as deletions and high-level gains contribute as much as the diploid majority. Balanced accuracy shares this motivation, averaging per-class recall so that majority-class dominance does not inflate aggregate scores. Per-class sensitivity reveals which specific CN states a method can and cannot detect, exposing failure modes that summary statistics may obscure. Alteration sensitivity measures the ability to detect any nondiploid event, the core clinical task of CNV screening, while alteration F1 balances detection of true alterations against false positives; this matters for clinical decision-making where both missed calls and spurious calls carry consequences. For the event-size analysis specifically, we report event detection sensitivity together with amplicon-level alteration F1 and specificity rather than overall accuracy or per-state macro-F1. A focal alteration occupies only a small fraction of the amplicons on its chromosome, so overall accuracy and macro-F1 are dominated by the surrounding diploid amplicons and reflect that background more than a method’s ability to localize the event; detection sensitivity scores recovery within the event, and amplicon-level alteration F1 and specificity capture the false positives such localization could introduce in the diploid flanks.

#### Confusion matrices

For each simulation replicate and method, we computed a multi-class confusion matrix $M\in {\mathbb{Z}}^{K\times K}$ over copy number states $\left\{1,\ldots ,K\right\}$. Predicted and ground truth copy number matrices were flattened to vectors; each element was tabulated into entry $M_{\mathrm{jk}}$, the count of observations with true state j and predicted state k. Per-class precision, recall (sensitivity), and F1 scores were derived from the confusion matrix as ${\text{Precision}}_{k}=M_{\mathrm{kk}}/\sum_{j}M_{\mathrm{jk}}$, ${\text{Recall}}_{k}=M_{\mathrm{kk}}/\sum_{j}M_{\mathrm{kj}}$, and $F1_{k}=2\cdot {\text{Precision}}_{k}\cdot {\text{Recall}}_{k}/\left({\text{Precision}}_{k}+{\text{Recall}}_{k}\right)$, respectively. Full confusion matrices for all simulation conditions are provided in Files S2 to S4.

### Application study

We applied both methods to the public karyotapR tutorial dataset [18,36], a mixture of 5 human cell lines sequenced with the Mission Bio Tapestri CO261 panel (330 designed probes spanning chromosomes 1 to 22 and X; 319 amplicons retained after quality filtering in the tutorial dataset). Cell populations were identified by allele frequency-based clustering using PCA (principal components analysis, UMAP (uniform manifold approximation and projection), and DBSCAN (density-based spatial clustering of applications with noise); identifying discrete cell populations from single-cell sequencing data is itself an active area of method development, with recent graph-partitioning approaches such as topology entropy reported to improve clustering accuracy [37]. Cell line 2 (RPE1) served as the near-diploid reference; its known trisomy of chromosome 10q was encoded as reference_cn = c(“10” = 3) in both methods.

The expected karyotypic profiles of the 5 lines, as determined in the source study by bulk whole-genome sequencing (and by G-banding for the RPE1 reference), are summarized in Table 1; these provide a reference against which the single-cell calls can be interpreted [18].

Preprocessing removed amplicons with near-zero median counts in reference cells (9 to 11 probes, analysis-dependent), normalized counts with the mb scheme, and extracted BAF values from the alleleFrequency assay with variants mapped to amplicons by genomic coordinates. karyotapR inferred continuous copy number values per amplicon, smoothed to segments, and fit a GMM with components 1 to 5. scPloidyR learned reference parameters, fit a per-chromosome HMM with states 1 to 5, and aggregated amplicon calls by segment mode. Matched single-cell ground truth is unavailable; instead, we evaluated concordance between methods and consistency with the published line-level karyotypes (Table 1), which serve as an external reference from orthogonal assays.

### Runtime and memory benchmarking

We benchmarked the computational cost of the core copy number calling step of each method on the Tapestri 5-cell-line dataset. Because karyotapR is single-threaded, while scPloidyR parallelizes its per-cell HMM across CPU cores, we report karyotapR on one core and scPloidyR on 8 cores (its default), and for each method, we report both wall-clock runtime and total CPU time (user + system, aggregated across all worker processes) so that the per-core cost of parallelism is visible. Each configuration was executed as an isolated subprocess wrapped by GNU time, which aggregates CPU time over the full process tree (including forked workers) and records peak resident memory as the maximum resident set size; with copy-on-write forking, this peak approximates the true memory footprint. To characterize scaling with the number of cells, we varied the total cell count over 1,000, 2,000, 4,000, and 8,000 while holding the reference set fixed, subsampling the query (nonreference) cells below the dataset size and oversampling them with replacement above it (the dataset contains 3,020 cells), and re-measured runtime and peak memory for both methods.

### Software application

All analyses were implemented in R and are available in Github. karyotapR (version 1.0.1) was used as distributed through CRAN. The scPloidyR package is available at https://github.com/dpei/scPloidyR, and the simulation study code is available at https://github.com/dpei/scPloidyR_simulation.

## Data Availability

All data needed to evaluate the conclusions of the study are available in the paper and its Supplementary Materials.
