# Supplementary material for: Integrative Modeling of Read Depth and B-Allele Frequency Improves Single-Cell Copy Number Calling from Targeted DNA Sequencing Panels
Source: Comput Struct Biotechnol J. 2026 Aug 21;35(1):0203. doi: 10.34133/csbj.0203 (PMC13494159; doi:10.34133/csbj.0203)
Supplement: Supplementary file 1 [file csbj.0203.f1.zip › revision_S2_confusion_comprehensive.pdf]

Confusion Matrices: Comprehensive Mode (Repetition 1)

Repetition 1 | All CN States 1–5 | BAF SD: 9.0 | Var/Amp: 3 | Amp/Chr: 4 | Cells: 100

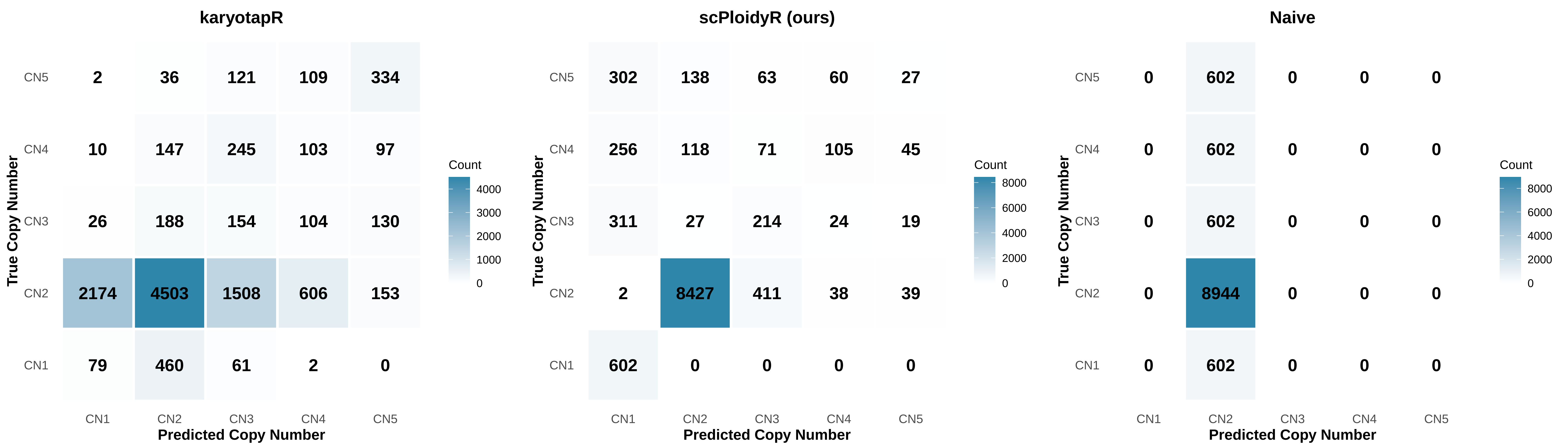

Confusion Matrices: Comprehensive Mode (Repetition 2)

Repetition 2 | All CN States 1–5 | BAF SD: 9.0 | Var/Amp: 3 | Amp/Chr: 4 | Cells: 100

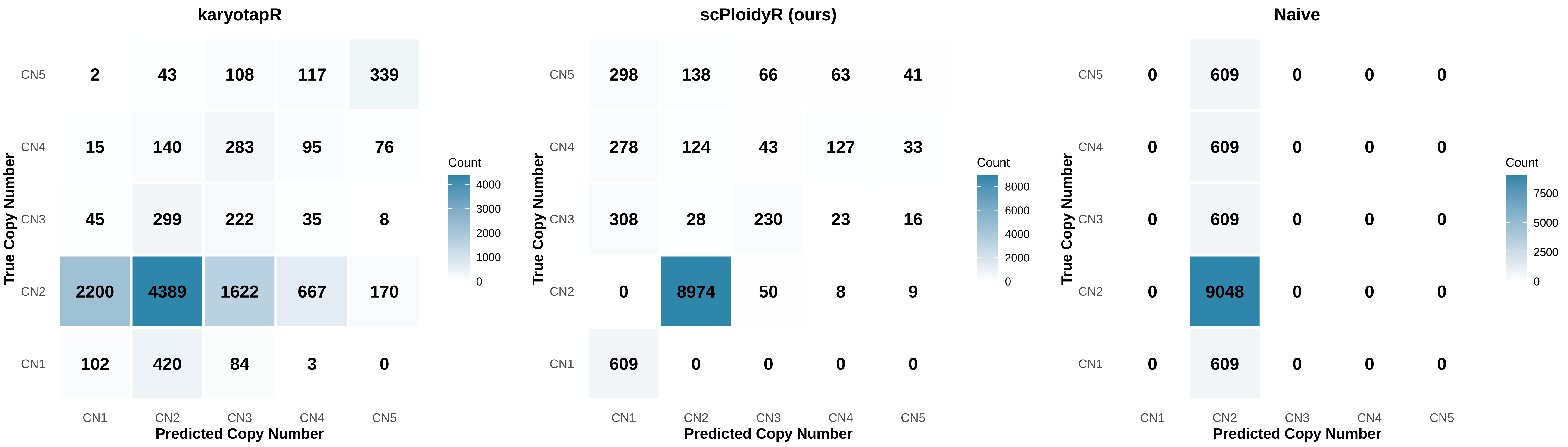

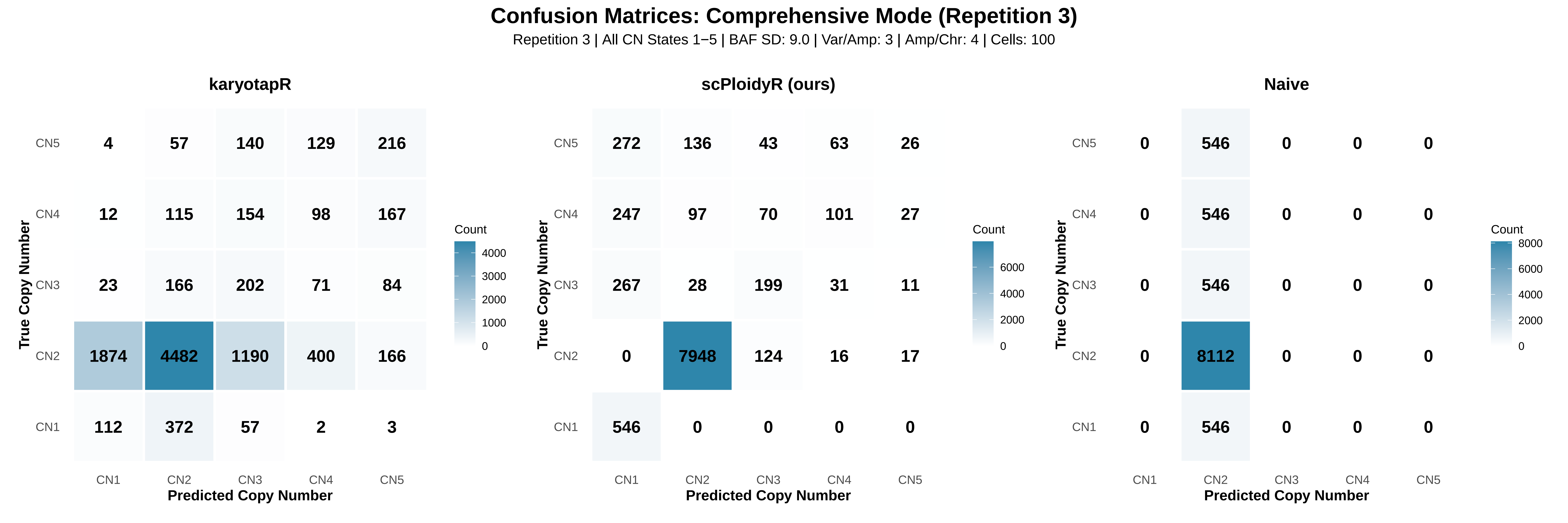
Confusion Matrices: Comprehensive Mode (Repetition 3)
Repetition 3 | All CN States 1–5 | BAF SD: 9.0 | Var/Amp: 3 | Amp/Chr: 4 | Cells: 100
karyotapR
CN5 4 57 140 129 216
CN4 12 115 154 98 167
CN3 23 166 202 71 84
CN2 1874 4482 1190 400 166
CN1 112 372 57 2 3
CN1 CN2 CN3 CN4 CN5
Predicted Copy Number
True Copy Number
Count
4000
3000
2000
1000
0
scPloidyR (ours)
CN5 272 136 43 63 26
CN4 247 97 70 101 27
CN3 267 28 199 31 11
CN2 0 7948 124 16 17
CN1 546 0 0 0 0
CN1 CN2 CN3 CN4 CN5
Predicted Copy Number
True Copy Number
Count
6000
4000
2000
0
Naive
CN5 0 546 0 0 0
CN4 0 546 0 0 0
CN3 0 546 0 0 0
CN2 0 8112 0 0 0
CN1 0 546 0 0 0
CN1 CN2 CN3 CN4 CN5
Predicted Copy Number
True Copy Number
Count
8000
6000
4000
2000
0

Confusion Matrices: Comprehensive Mode (Repetition 4)

Repetition 4 | All CN States 1–5 | BAF SD: 9.0 | Var/Amp: 3 | Amp/Chr: 4 | Cells: 100

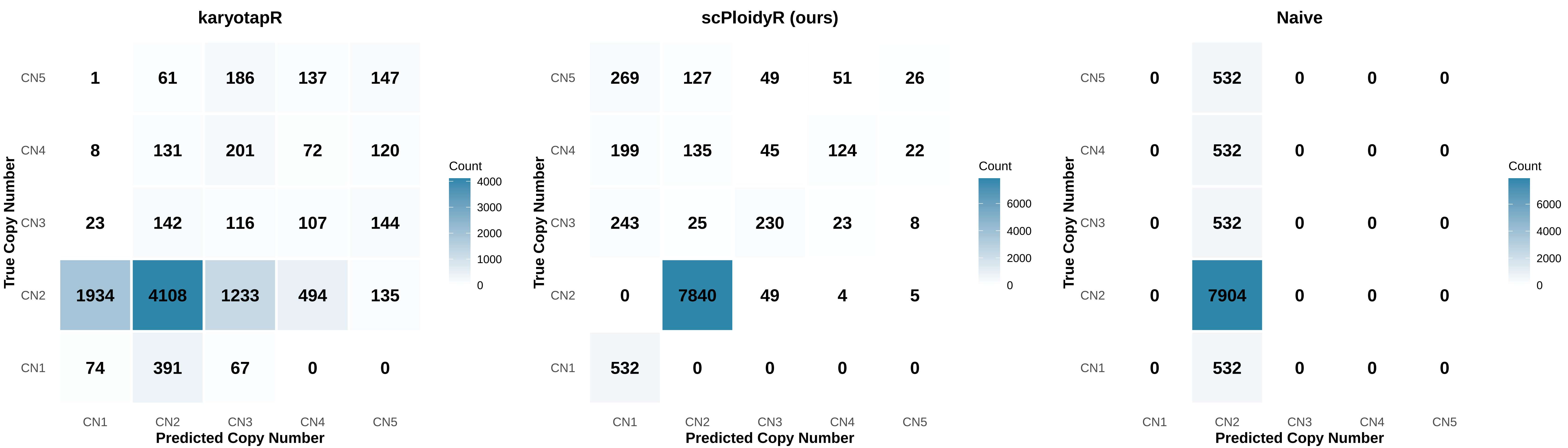

Confusion Matrices: Comprehensive Mode (Repetition 5)

Repetition 5 | All CN States 1–5 | BAF SD: 9.0 | Var/Amp: 3 | Amp/Chr: 4 | Cells: 100

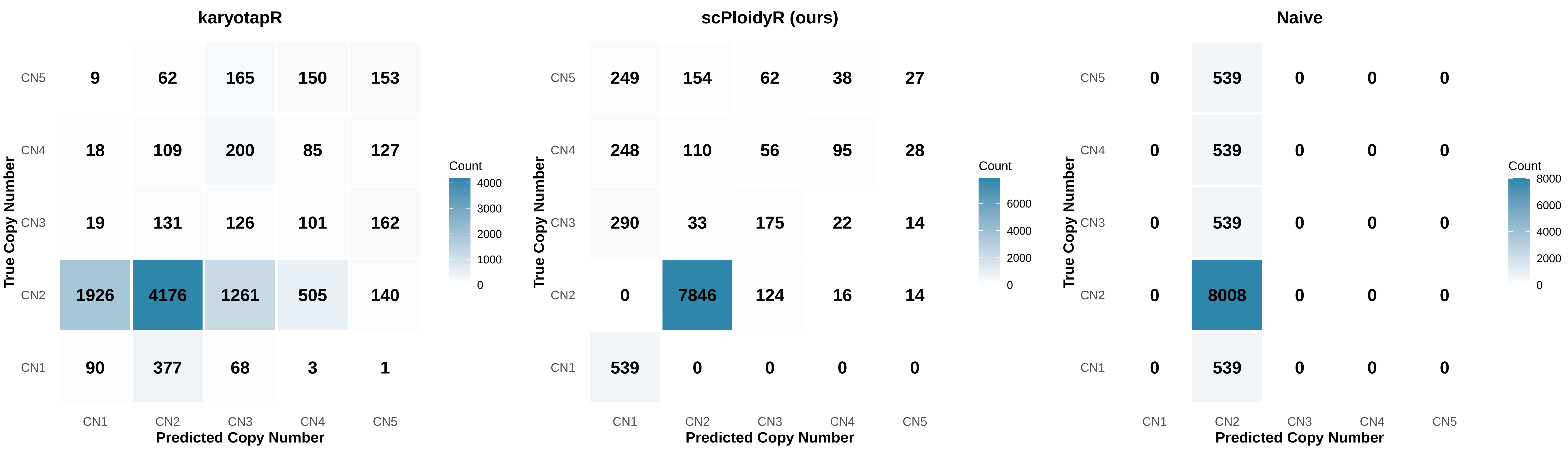

Confusion Matrices: Comprehensive Mode (Repetition 6)

Repetition 6 | All CN States 1–5 | BAF SD: 9.0 | Var/Amp: 3 | Amp/Chr: 4 | Cells: 100

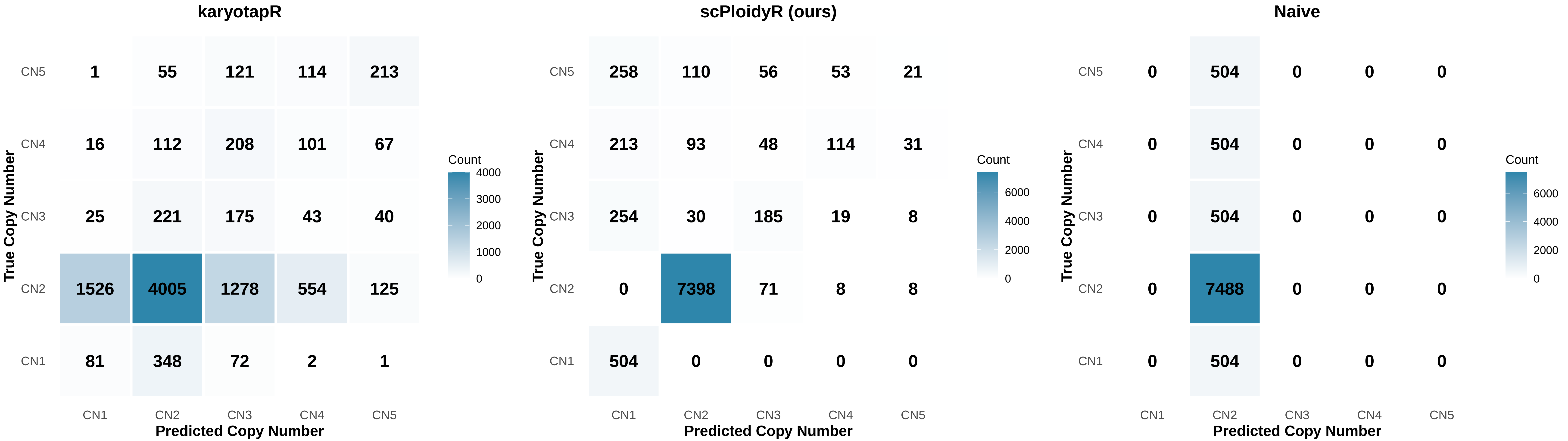

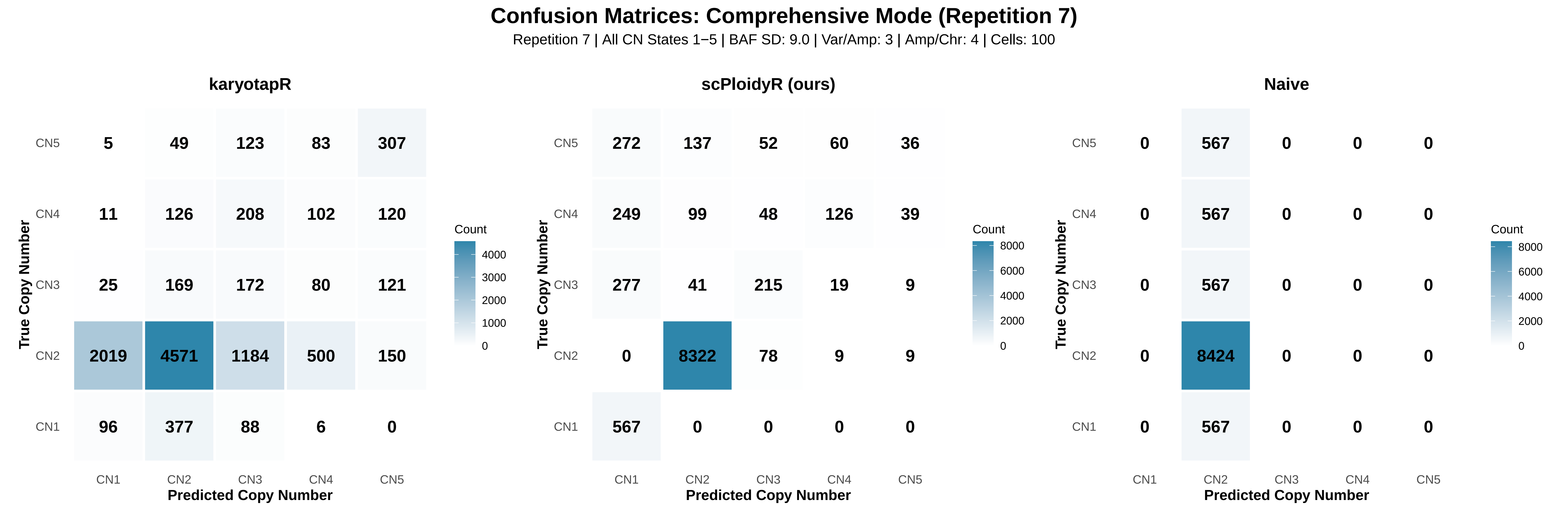
Confusion Matrices: Comprehensive Mode (Repetition 7)
Repetition 7 | All CN States 1–5 | BAF SD: 9.0 | Var/Amp: 3 | Amp/Chr: 4 | Cells: 100
karyotapR
CN5 5 49 123 83 307
CN4 11 126 208 102 120
CN3 25 169 172 80 121
CN2 2019 4571 1184 500 150
CN1 96 377 88 6 0
CN1 CN2 CN3 CN4 CN5
True Copy Number
Predicted Copy Number
Count
4000
3000
2000
1000
0
scPloidyR (ours)
CN5 272 137 52 60 36
CN4 249 99 48 126 39
CN3 277 41 215 19 9
CN2 0 8322 78 9 9
CN1 567 0 0 0 0
CN1 CN2 CN3 CN4 CN5
True Copy Number
Predicted Copy Number
Count
8000
6000
4000
2000
0
Naive
CN5 0 567 0 0 0
CN4 0 567 0 0 0
CN3 0 567 0 0 0
CN2 0 8424 0 0 0
CN1 0 567 0 0 0
CN1 CN2 CN3 CN4 CN5
True Copy Number
Predicted Copy Number
Count
8000
6000
4000
2000
0

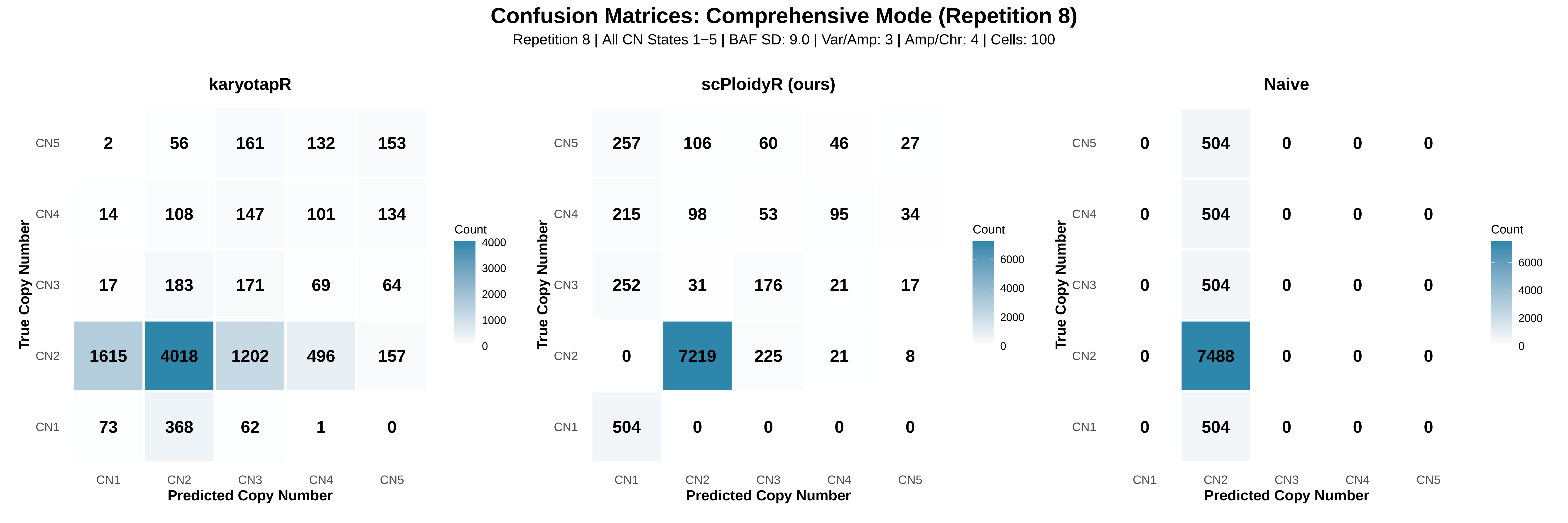
Confusion Matrices: Comprehensive Mode (Repetition 8)
Repetition 8 | All CN States 1–5 | BAF SD: 9.0 | Var/Amp: 3 | Amp/Chr: 4 | Cells: 100
karyotapR
scPloidyR (ours)
Naive
True Copy Number
Predicted Copy Number
Count
CN1
CN2
CN3
CN4
CN5

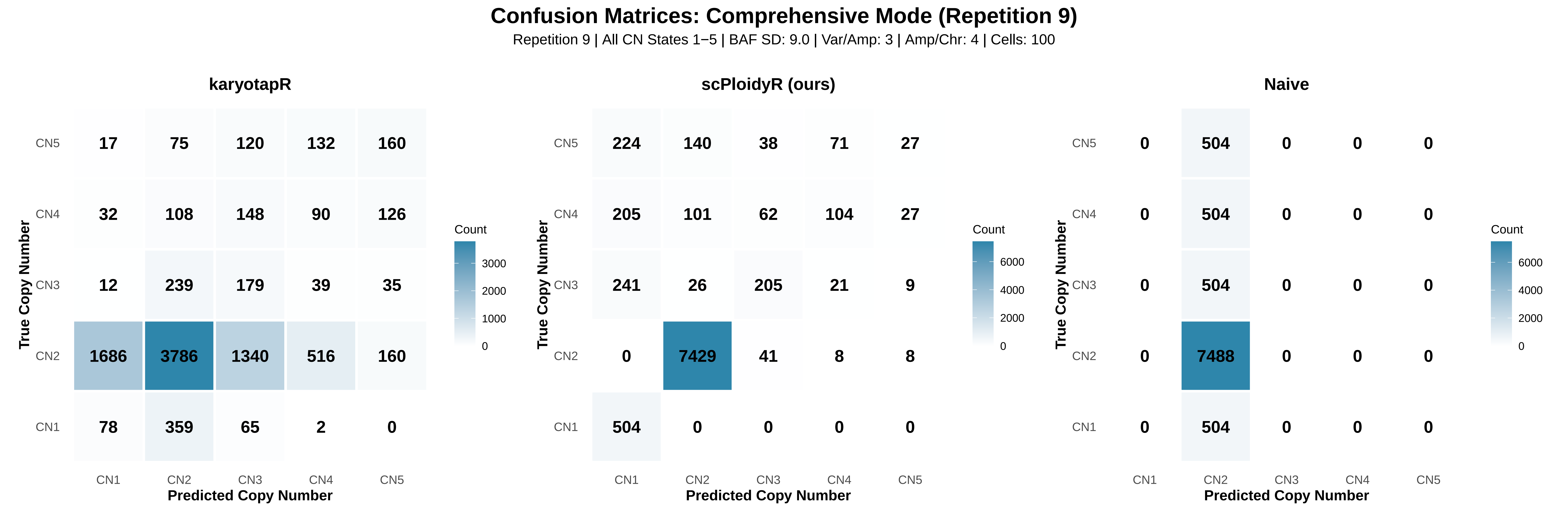
Confusion Matrices: Comprehensive Mode (Repetition 9)
Repetition 9 | All CN States 1–5 | BAF SD: 9.0 | Var/Amp: 3 | Amp/Chr: 4 | Cells: 100
karyotapR
CN5 17 75 120 132 160
CN4 32 108 148 90 126
CN3 12 239 179 39 35
CN2 1686 3786 1340 516 160
CN1 78 359 65 2 0
CN1 CN2 CN3 CN4 CN5
Predicted Copy Number
True Copy Number
Count
3000
2000
1000
0
scPloidyR (ours)
CN5 224 140 38 71 27
CN4 205 101 62 104 27
CN3 241 26 205 21 9
CN2 0 7429 41 8 8
CN1 504 0 0 0 0
CN1 CN2 CN3 CN4 CN5
Predicted Copy Number
True Copy Number
Count
6000
4000
2000
0
Naive
CN5 0 504 0 0 0
CN4 0 504 0 0 0
CN3 0 504 0 0 0
CN2 0 7488 0 0 0
CN1 0 504 0 0 0
CN1 CN2 CN3 CN4 CN5
Predicted Copy Number
True Copy Number
Count
6000
4000
2000
0

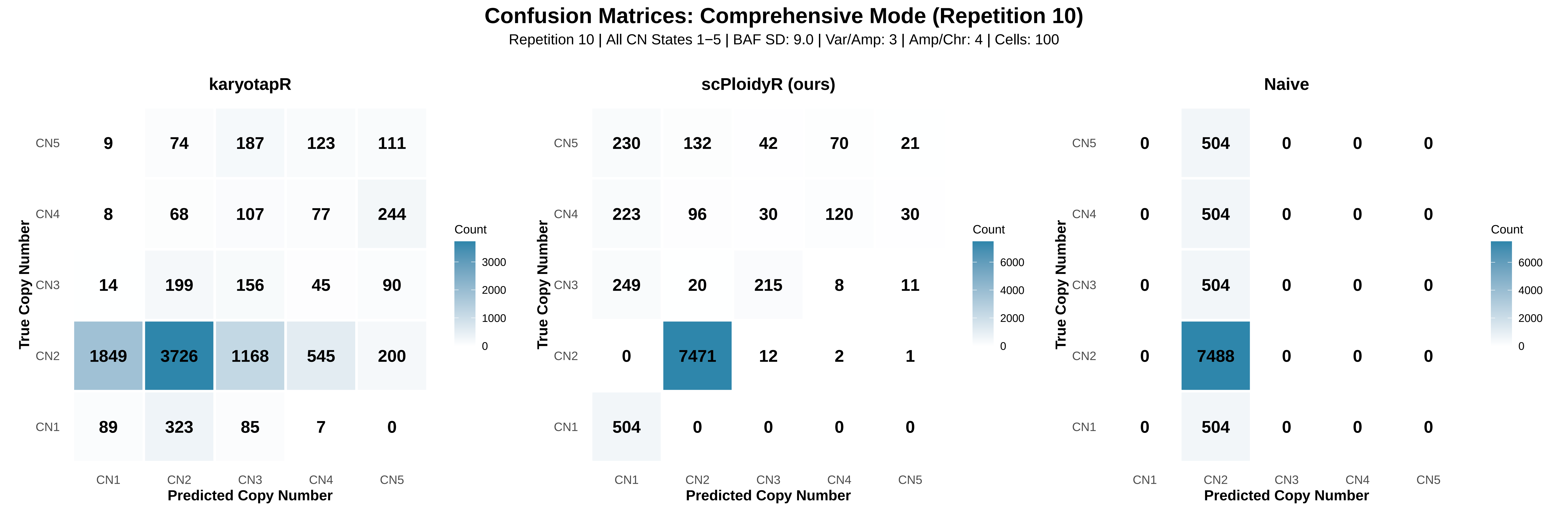
Confusion Matrices: Comprehensive Mode (Repetition 10)
Repetition 10 | All CN States 1−5 | BAF SD: 9.0 | Var/Amp: 3 | Amp/Chr: 4 | Cells: 100
karyotapR
CN5 9 74 187 123 111
CN4 8 68 107 77 244
CN3 14 199 156 45 90
CN2 1849 3726 1168 545 200
CN1 89 323 85 7 0
CN1 CN2 CN3 CN4 CN5
True Copy Number
Predicted Copy Number
Count
3000
2000
1000
0
scPloidyR (ours)
CN5 230 132 42 70 21
CN4 223 96 30 120 30
CN3 249 20 215 8 11
CN2 0 7471 12 2 1
CN1 504 0 0 0 0
CN1 CN2 CN3 CN4 CN5
True Copy Number
Predicted Copy Number
Count
6000
4000
2000
0
Naive
CN5 0 504 0 0 0
CN4 0 504 0 0 0
CN3 0 504 0 0 0
CN2 0 7488 0 0 0
CN1 0 504 0 0 0
CN1 CN2 CN3 CN4 CN5
True Copy Number
Predicted Copy Number
Count
6000
4000
2000
0
