# Supplementary material for: Integrative Modeling of Read Depth and B-Allele Frequency Improves Single-Cell Copy Number Calling from Targeted DNA Sequencing Panels
Source: Comput Struct Biotechnol J. 2026 Aug 21;35(1):0203. doi: 10.34133/csbj.0203 (PMC13494159; doi:10.34133/csbj.0203)

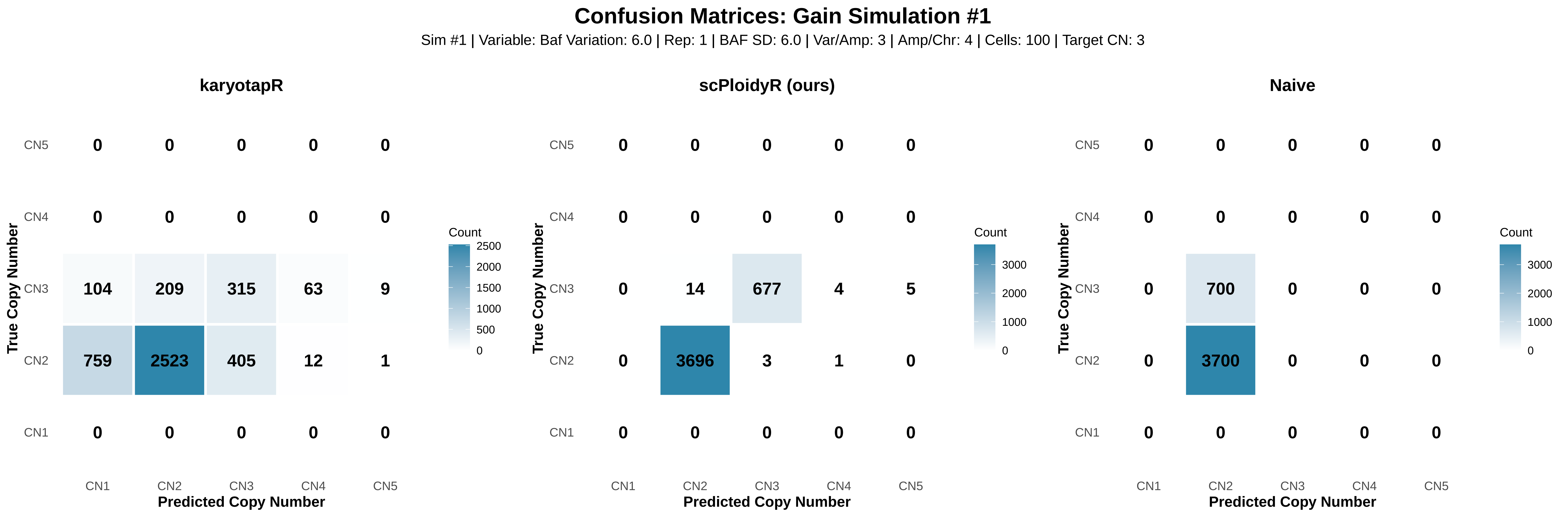
Confusion Matrices: Gain Simulation #1
Sim #1 | Variable: Baf Variation: 6.0 | Rep: 1 | BAF SD: 6.0 | Var/Amp: 3 | Amp/Chr: 4 | Cells: 100 | Target CN: 3
karyotapR
scPloidyR (ours)
Naive
True Copy Number
Predicted Copy Number
CN1
CN2
CN3
CN4
CN5
Count
2500
2000
1500
1000
500
0
3000
karyotapR: CN5: 0 0 0 0 0
CN4: 0 0 0 0 0
CN3: 104 209 315 63 9
CN2: 759 2523 405 12 1
CN1: 0 0 0 0 0
scPloidyR (ours): CN5: 0 0 0 0 0
CN4: 0 0 0 0 0
CN3: 0 14 677 4 5
CN2: 0 3696 3 1 0
CN1: 0 0 0 0 0
Naive: CN5: 0 0 0 0 0
CN4: 0 0 0 0 0
CN3: 0 700 0 0 0
CN2: 0 3700 0 0 0
CN1: 0 0 0 0 0

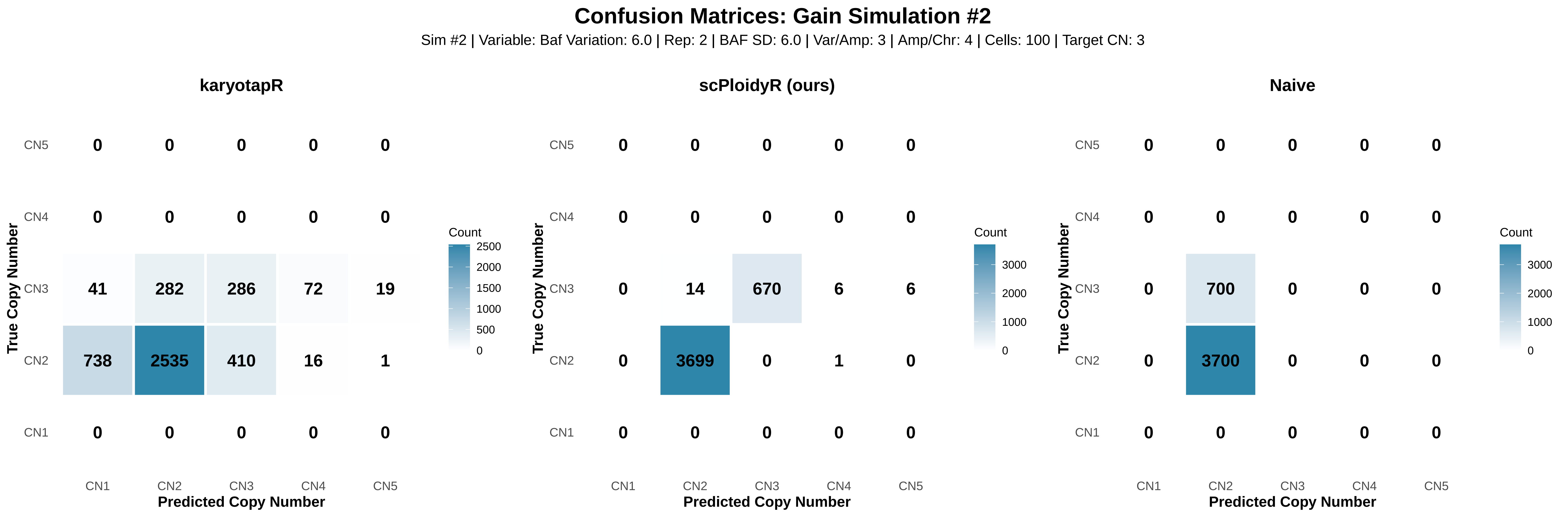
Confusion Matrices: Gain Simulation #2
Sim #2 | Variable: Baf Variation: 6.0 | Rep: 2 | BAF SD: 6.0 | Var/Amp: 3 | Amp/Chr: 4 | Cells: 100 | Target CN: 3
karyotapR
CN5 0 0 0 0 0
CN4 0 0 0 0 0
CN3 41 282 286 72 19
CN2 738 2535 410 16 1
CN1 0 0 0 0 0
CN1 CN2 CN3 CN4 CN5
Predicted Copy Number
True Copy Number
Count
2500
2000
1500
1000
500
0
scPloidyR (ours)
CN5 0 0 0 0 0
CN4 0 0 0 0 0
CN3 0 14 670 6 6
CN2 0 3699 0 1 0
CN1 0 0 0 0 0
CN1 CN2 CN3 CN4 CN5
Predicted Copy Number
True Copy Number
Count
3000
2000
1000
0
Naive
CN5 0 0 0 0 0
CN4 0 0 0 0 0
CN3 0 700 0 0 0
CN2 0 3700 0 0 0
CN1 0 0 0 0 0
CN1 CN2 CN3 CN4 CN5
Predicted Copy Number
True Copy Number
Count
3000
2000
1000
0

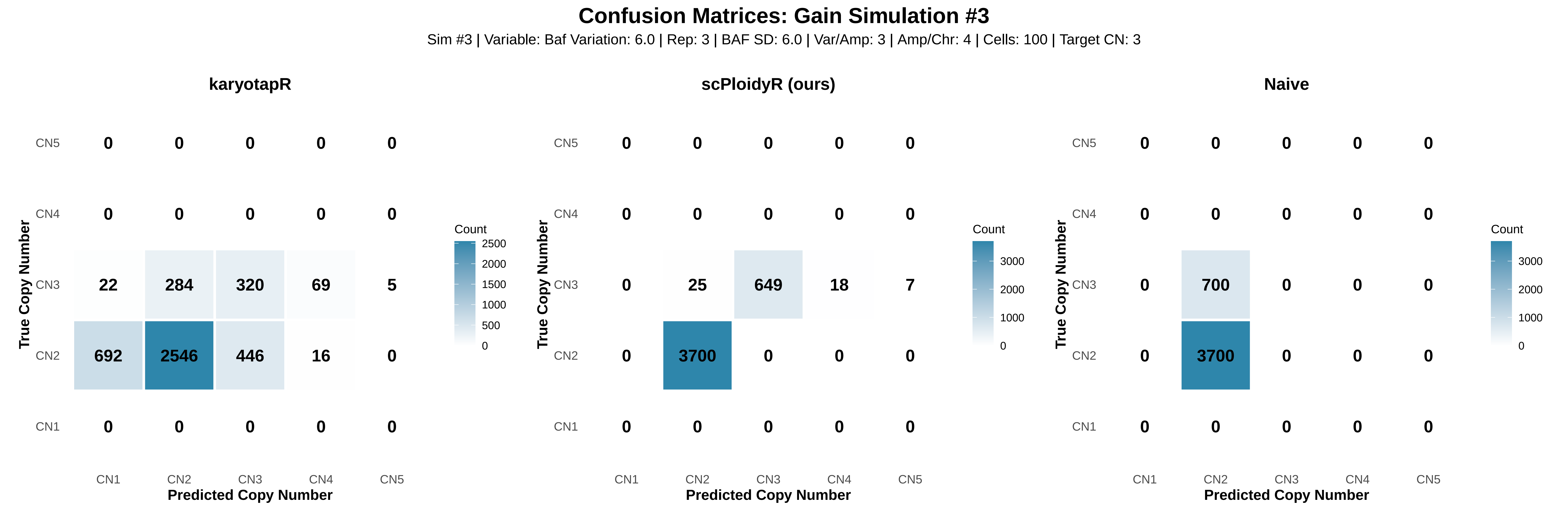
Confusion Matrices: Gain Simulation #3
Sim #3 | Variable: Baf Variation: 6.0 | Rep: 3 | BAF SD: 6.0 | Var/Amp: 3 | Amp/Chr: 4 | Cells: 100 | Target CN: 3
karyotapR
scPloidyR (ours)
Naive
True Copy Number
Predicted Copy Number
Count
CN1
CN2
CN3
CN4
CN5
22
284
320
69
5
692
2546
446
16
25
649
18
7
3700
700

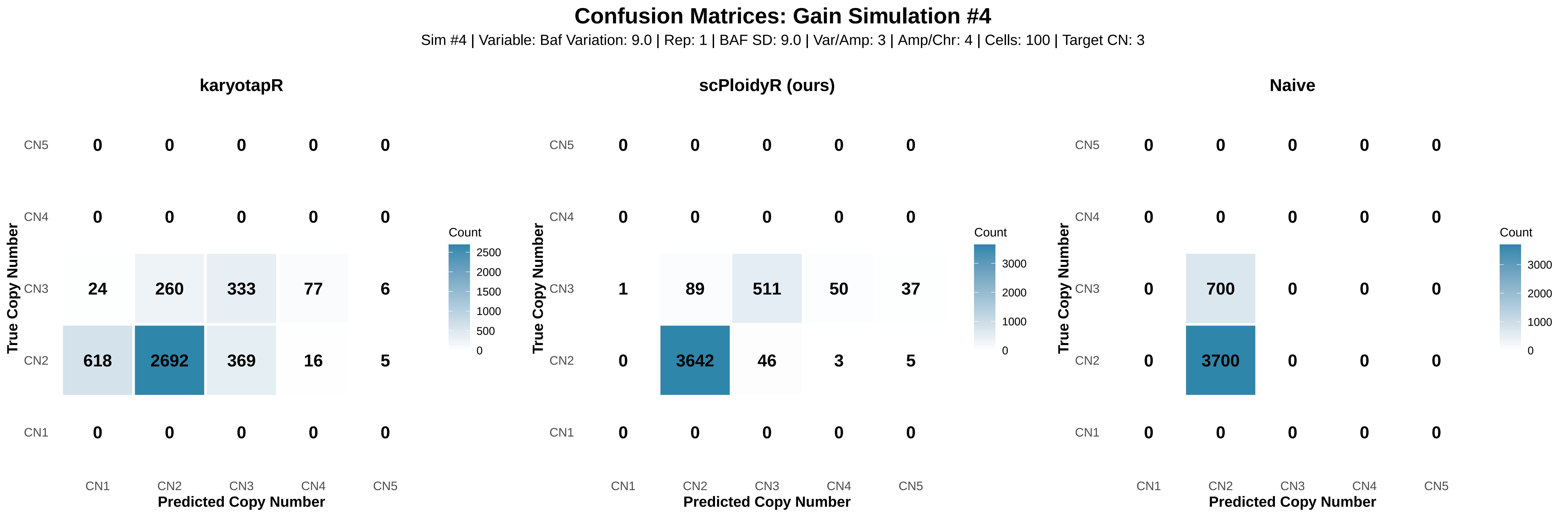
Confusion Matrices: Gain Simulation #4
Sim #4 | Variable: Baf Variation: 9.0 | Rep: 1 | BAF SD: 9.0 | Var/Amp: 3 | Amp/Chr: 4 | Cells: 100 | Target CN: 3
karyotapR
scPloidyR (ours)
Naive
True Copy Number
Predicted Copy Number
CN1
CN2
CN3
CN4
CN5
Count
0
500
1000
1500
2000
2500
3000
24
260
333
77
6
618
2692
369
16
5
1
89
511
50
37
3642
46
3
700
3700

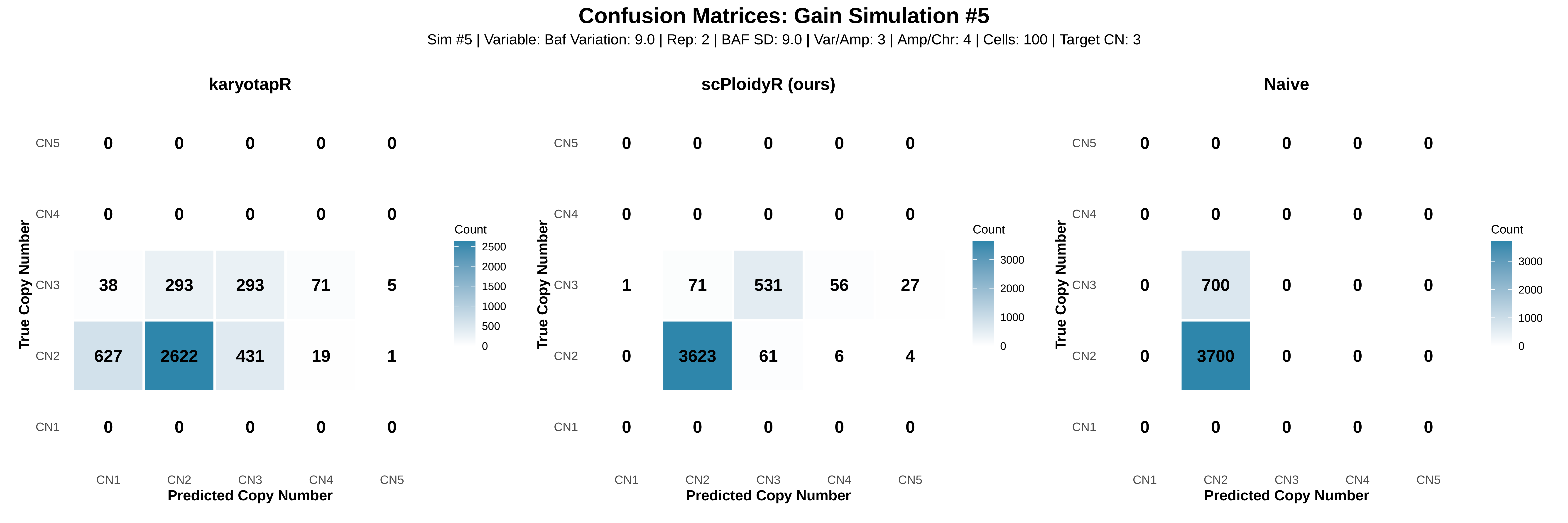
Confusion Matrices: Gain Simulation #5
Sim #5 | Variable: Baf Variation: 9.0 | Rep: 2 | BAF SD: 9.0 | Var/Amp: 3 | Amp/Chr: 4 | Cells: 100 | Target CN: 3
karyotapR
scPloidyR (ours)
Naive
True Copy Number
Predicted Copy Number
CN1
CN2
CN3
CN4
CN5
Count
0
500
1000
1500
2000
2500
3000
38
293
293
71
5
627
2622
431
19
1
71
531
56
27
3623
61
6
4
700
3700

Confusion Matrices: Gain Simulation #6

Sim #6 | Variable: Baf Variation: 9.0 | Rep: 3 | BAF SD: 9.0 | Var/Amp: 3 | Amp/Chr: 4 | Cells: 100 | Target CN: 3

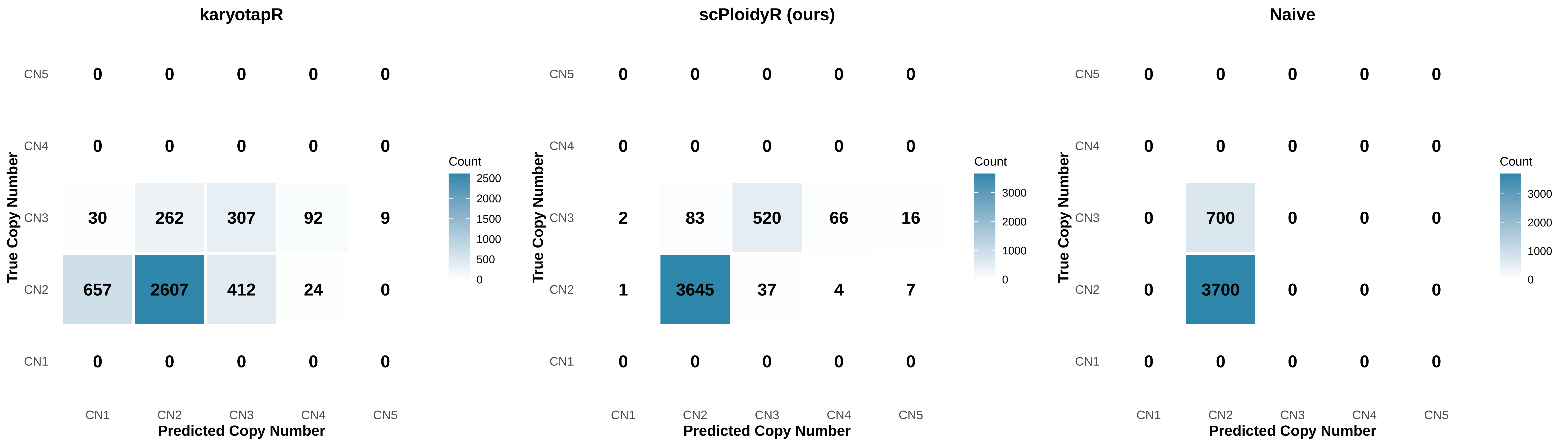

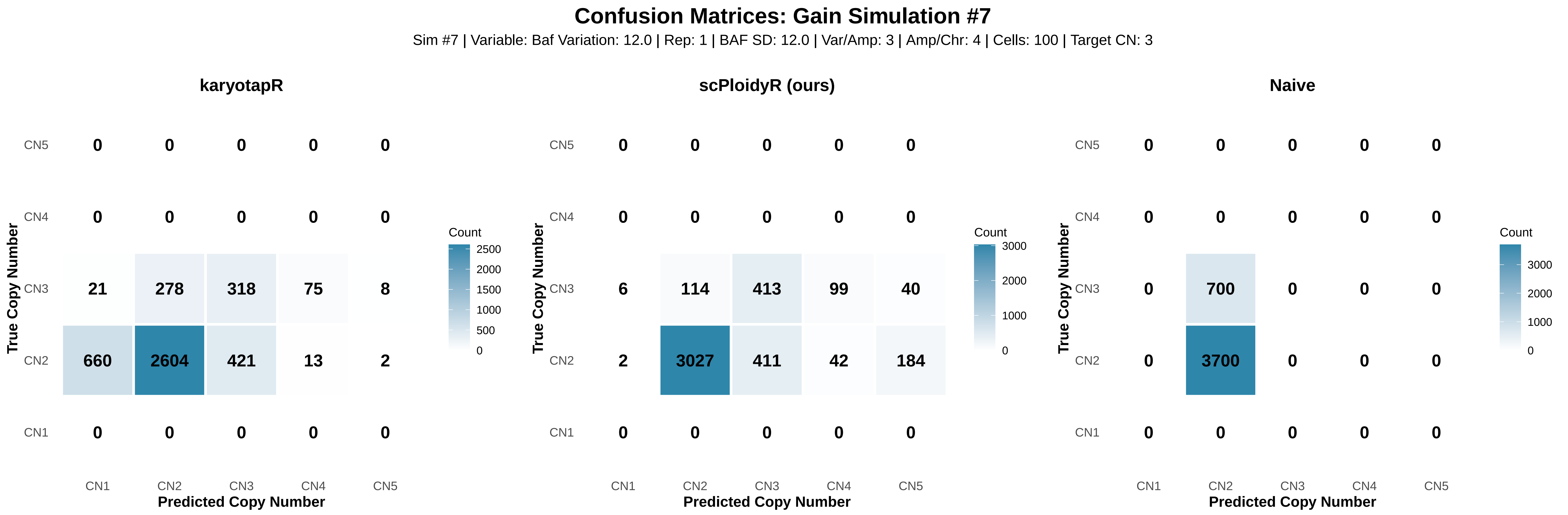
Confusion Matrices: Gain Simulation #7
Sim #7 | Variable: Baf Variation: 12.0 | Rep: 1 | BAF SD: 12.0 | Var/Amp: 3 | Amp/Chr: 4 | Cells: 100 | Target CN: 3
karyotapR
scPloidyR (ours)
Naive
True Copy Number
Predicted Copy Number
CN1
CN2
CN3
CN4
CN5
Count
karyotapR: CN3: 21 278 318 75 8
karyotapR: CN2: 660 2604 421 13 2
scPloidyR: CN3: 6 114 413 99 40
scPloidyR: CN2: 2 3027 411 42 184
Naive: CN3: 0 700 0 0 0
Naive: CN2: 0 3700 0 0 0
0
500
1000
1500
2000
2500
3000

Confusion Matrices: Gain Simulation #8

Sim #8 | Variable: Baf Variation: 12.0 | Rep: 2 | BAF SD: 12.0 | Var/Amp: 3 | Amp/Chr: 4 | Cells: 100 | Target CN: 3

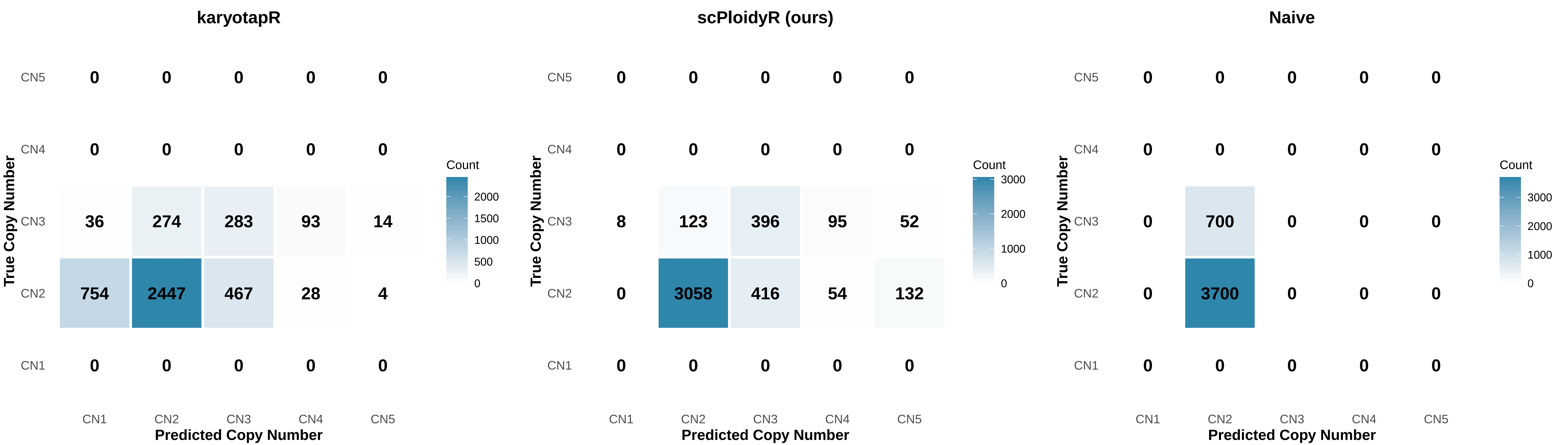

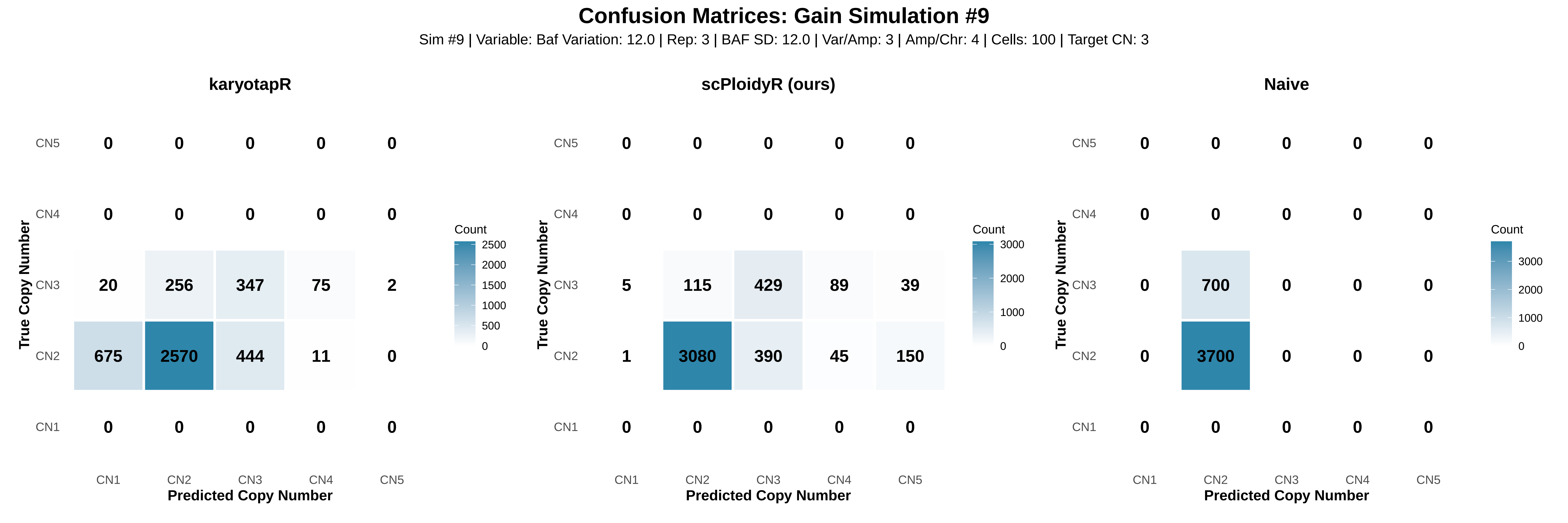
Confusion Matrices: Gain Simulation #9
Sim #9 | Variable: Baf Variation: 12.0 | Rep: 3 | BAF SD: 12.0 | Var/Amp: 3 | Amp/Chr: 4 | Cells: 100 | Target CN: 3
karyotapR
True Copy Number
CN5 0 0 0 0 0
CN4 0 0 0 0 0
CN3 20 256 347 75 2
CN2 675 2570 444 11 0
CN1 0 0 0 0 0
CN1 CN2 CN3 CN4 CN5
Predicted Copy Number
Count
2500
2000
1500
1000
500
0
scPloidyR (ours)
True Copy Number
CN5 0 0 0 0 0
CN4 0 0 0 0 0
CN3 5 115 429 89 39
CN2 1 3080 390 45 150
CN1 0 0 0 0 0
CN1 CN2 CN3 CN4 CN5
Predicted Copy Number
Count
3000
2000
1000
0
Naive
True Copy Number
CN5 0 0 0 0 0
CN4 0 0 0 0 0
CN3 0 700 0 0 0
CN2 0 3700 0 0 0
CN1 0 0 0 0 0
CN1 CN2 CN3 CN4 CN5
Predicted Copy Number
Count
3000
2000
1000
0

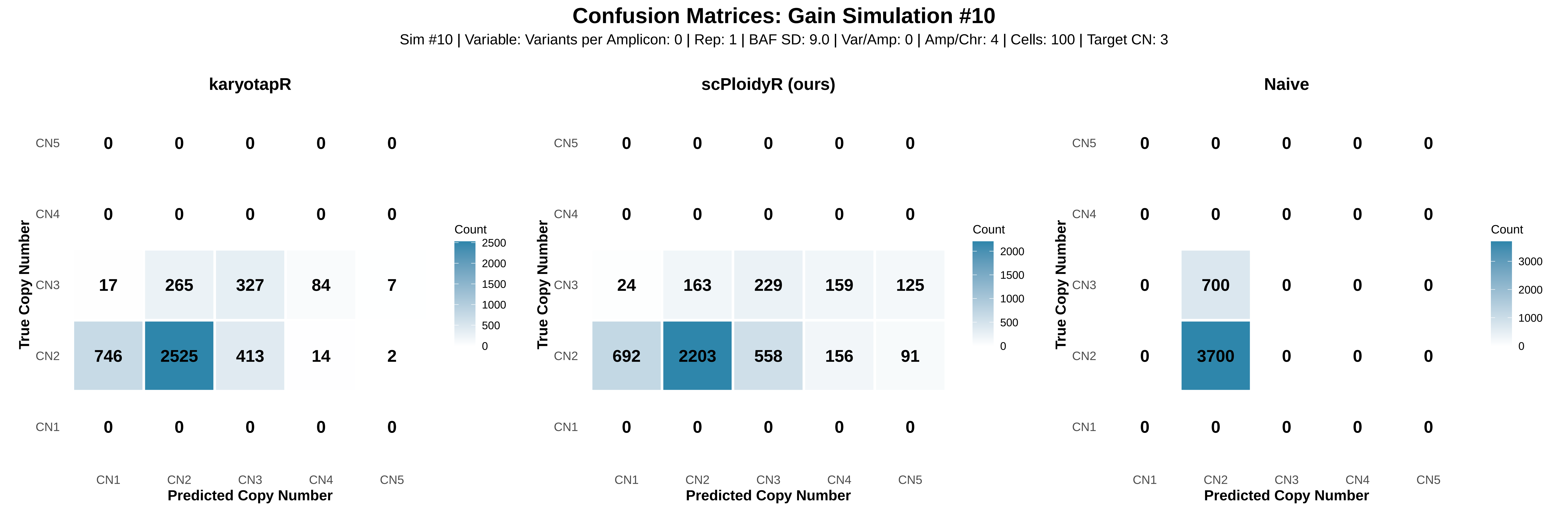

Confusion Matrices: Gain Simulation #10
Sim #10 | Variable: Variants per Amplicon: 0 | Rep: 1 | BAF SD: 9.0 | Var/Amp: 0 | Amp/Chr: 4 | Cells: 100 | Target CN: 3
karyotapR
scPloidyR (ours)
Naive
True Copy Number
Predicted Copy Number
Count
CN1
CN2
CN3
CN4
CN5

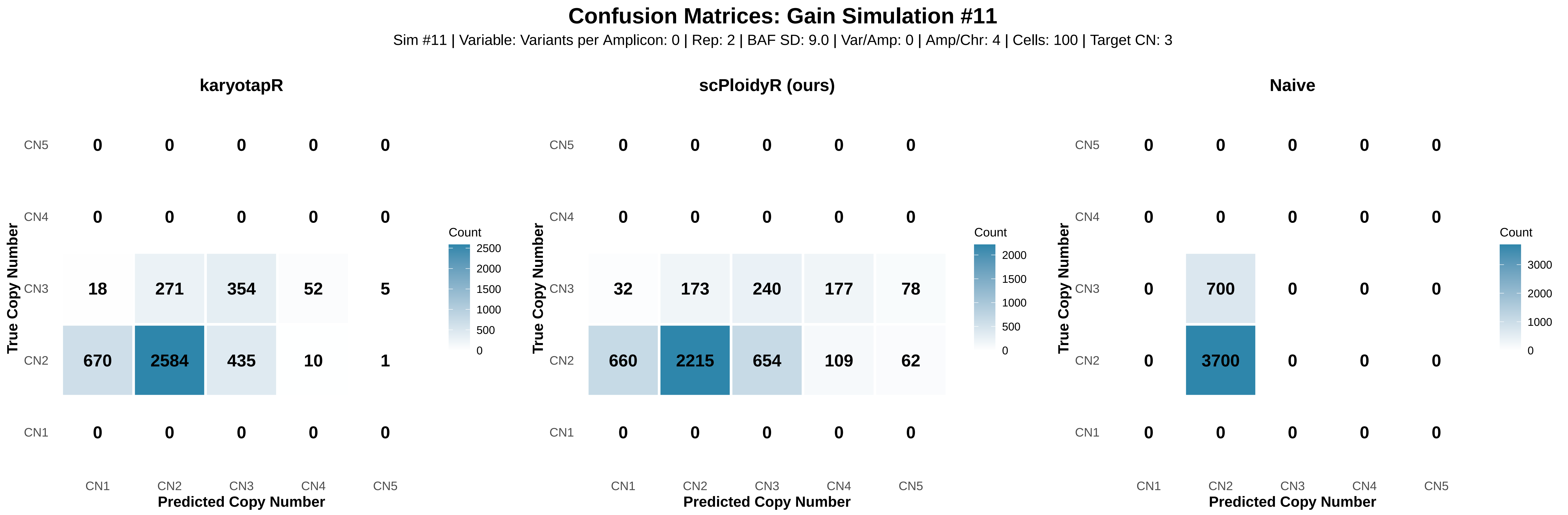
Confusion Matrices: Gain Simulation #11
Sim #11 | Variable: Variants per Amplicon: 0 | Rep: 2 | BAF SD: 9.0 | Var/Amp: 0 | Amp/Chr: 4 | Cells: 100 | Target CN: 3
karyotapR
CN5 0 0 0 0 0
CN4 0 0 0 0 0
CN3 18 271 354 52 5
CN2 670 2584 435 10 1
CN1 0 0 0 0 0
CN1 CN2 CN3 CN4 CN5
True Copy Number
Predicted Copy Number
Count
2500
2000
1500
1000
500
0
scPloidyR (ours)
CN5 0 0 0 0 0
CN4 0 0 0 0 0
CN3 32 173 240 177 78
CN2 660 2215 654 109 62
CN1 0 0 0 0 0
CN1 CN2 CN3 CN4 CN5
True Copy Number
Predicted Copy Number
Count
2000
1500
1000
500
0
Naive
CN5 0 0 0 0 0
CN4 0 0 0 0 0
CN3 0 700 0 0 0
CN2 0 3700 0 0 0
CN1 0 0 0 0 0
CN1 CN2 CN3 CN4 CN5
True Copy Number
Predicted Copy Number
Count
3000
2000
1000
0

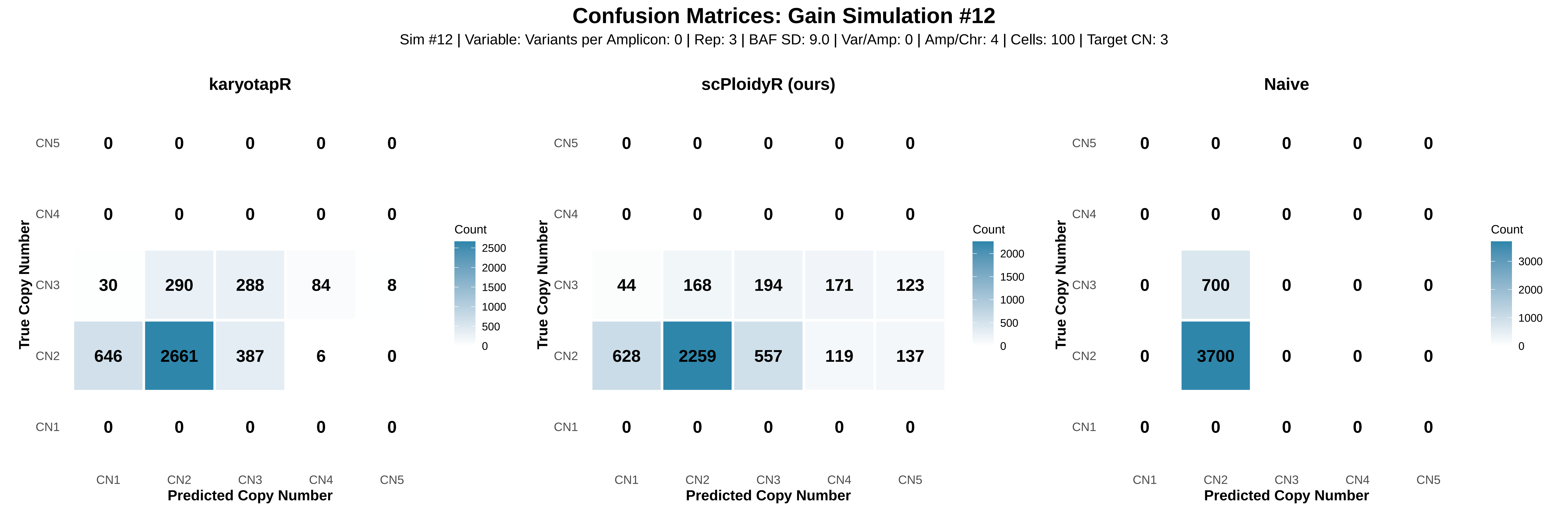
Confusion Matrices: Gain Simulation #12
Sim #12 | Variable: Variants per Amplicon: 0 | Rep: 3 | BAF SD: 9.0 | Var/Amp: 0 | Amp/Chr: 4 | Cells: 100 | Target CN: 3
karyotapR
scPloidyR (ours)
Naive
True Copy Number
Predicted Copy Number
CN1
CN2
CN3
CN4
CN5
Count
2500
2000
1500
1000
500
0
3000
karyotapR CN3: 30 290 288 84 8
karyotapR CN2: 646 2661 387 6 0
scPloidyR CN3: 44 168 194 171 123
scPloidyR CN2: 628 2259 557 119 137
Naive CN3: 0 700 0 0 0
Naive CN2: 0 3700 0 0 0

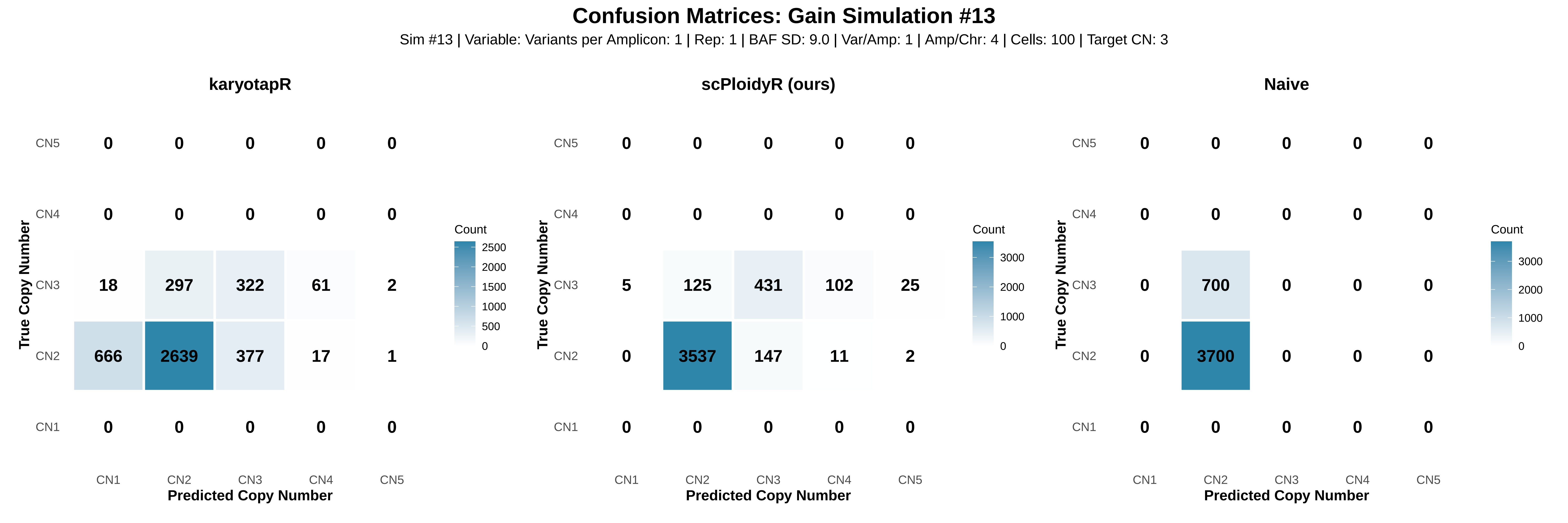
Confusion Matrices: Gain Simulation #13
Sim #13 | Variable: Variants per Amplicon: 1 | Rep: 1 | BAF SD: 9.0 | Var/Amp: 1 | Amp/Chr: 4 | Cells: 100 | Target CN: 3
karyotapR
scPloidyR (ours)
Naive
True Copy Number
Predicted Copy Number
CN1
CN2
CN3
CN4
CN5
Count
0
18
297
322
61
2
666
2639
377
17
1
5
125
431
102
25
3537
147
11
700
3700
500
1000
1500
2000
2500
3000

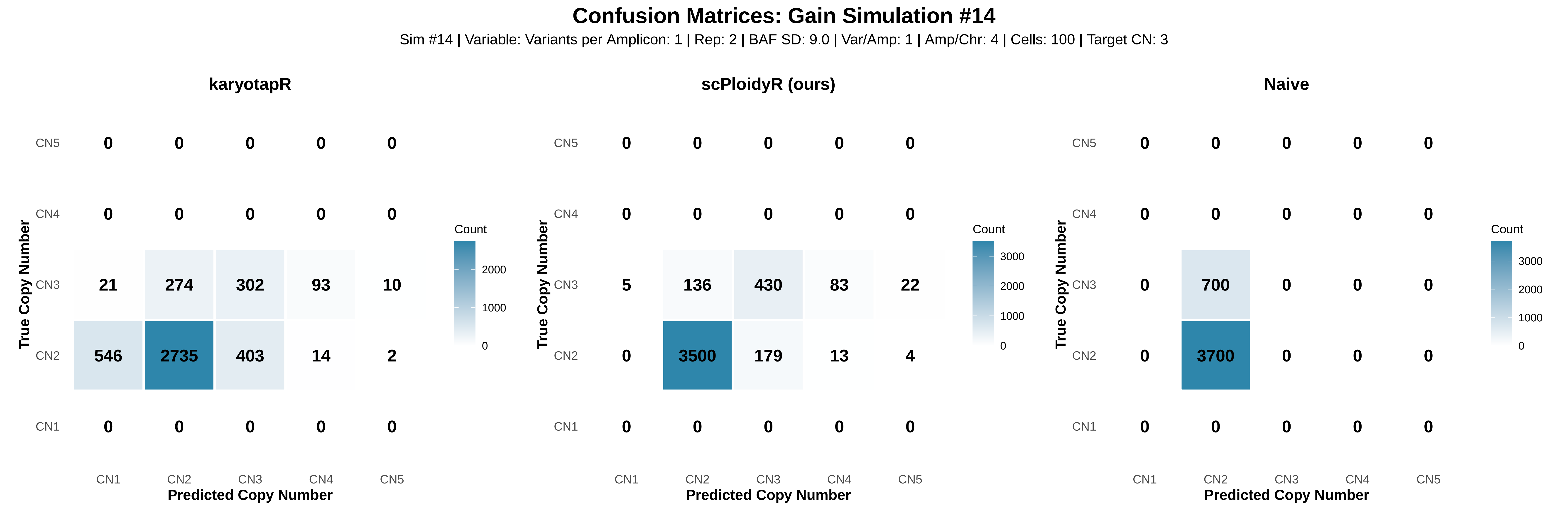

Confusion Matrices: Gain Simulation #14
Sim #14 | Variable: Variants per Amplicon: 1 | Rep: 2 | BAF SD: 9.0 | Var/Amp: 1 | Amp/Chr: 4 | Cells: 100 | Target CN: 3
karyotapR
scPloidyR (ours)
Naive
True Copy Number
Predicted Copy Number
CN1
CN2
CN3
CN4
CN5
Count
0
1000
2000
3000
21
274
302
93
10
546
2735
403
14
2
5
136
430
83
22
3500
179
13
4
700
3700

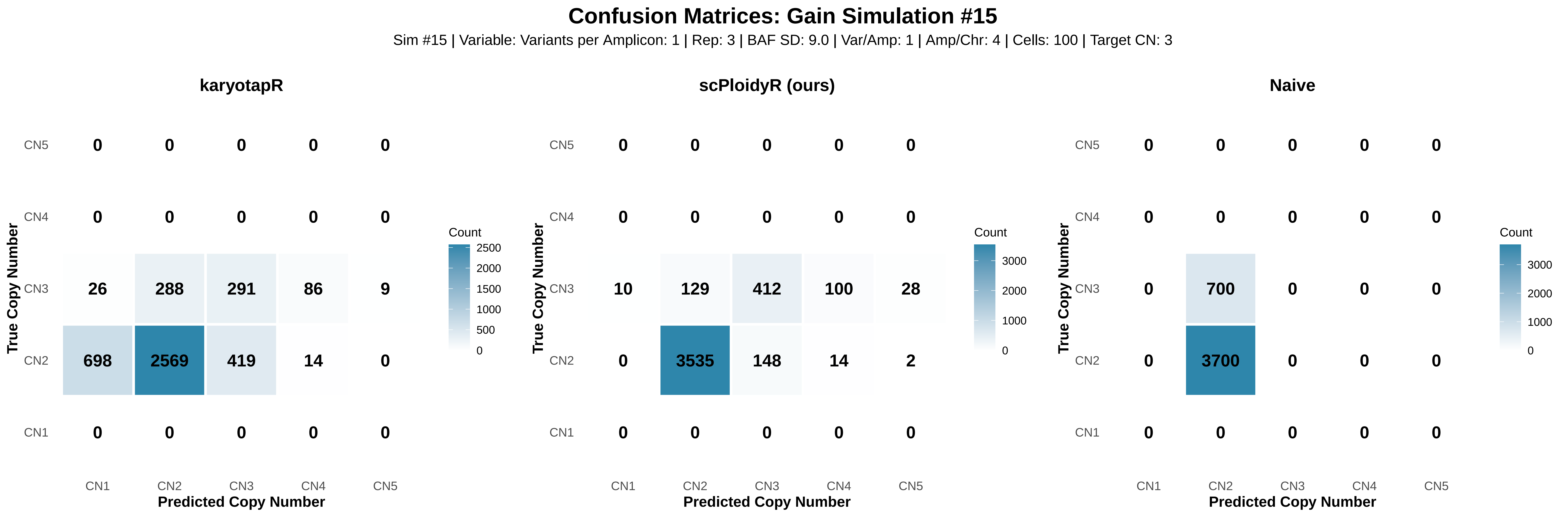
Confusion Matrices: Gain Simulation #15
Sim #15 | Variable: Variants per Amplicon: 1 | Rep: 3 | BAF SD: 9.0 | Var/Amp: 1 | Amp/Chr: 4 | Cells: 100 | Target CN: 3
karyotapR
scPloidyR (ours)
Naive
True Copy Number
Predicted Copy Number
CN1
CN2
CN3
CN4
CN5
Count
karyotapR: CN3 row: 26, 288, 291, 86, 9; CN2 row: 698, 2569, 419, 14, 0; CN5, CN4, CN1 rows: 0, 0, 0, 0, 0
scPloidyR (ours): CN3 row: 10, 129, 412, 100, 28; CN2 row: 0, 3535, 148, 14, 2; CN5, CN4, CN1 rows: 0, 0, 0, 0, 0
Naive: CN3 row: 0, 700, 0, 0, 0; CN2 row: 0, 3700, 0, 0, 0; CN5, CN4, CN1 rows: 0, 0, 0, 0, 0
0
500
1000
1500
2000
2500
3000

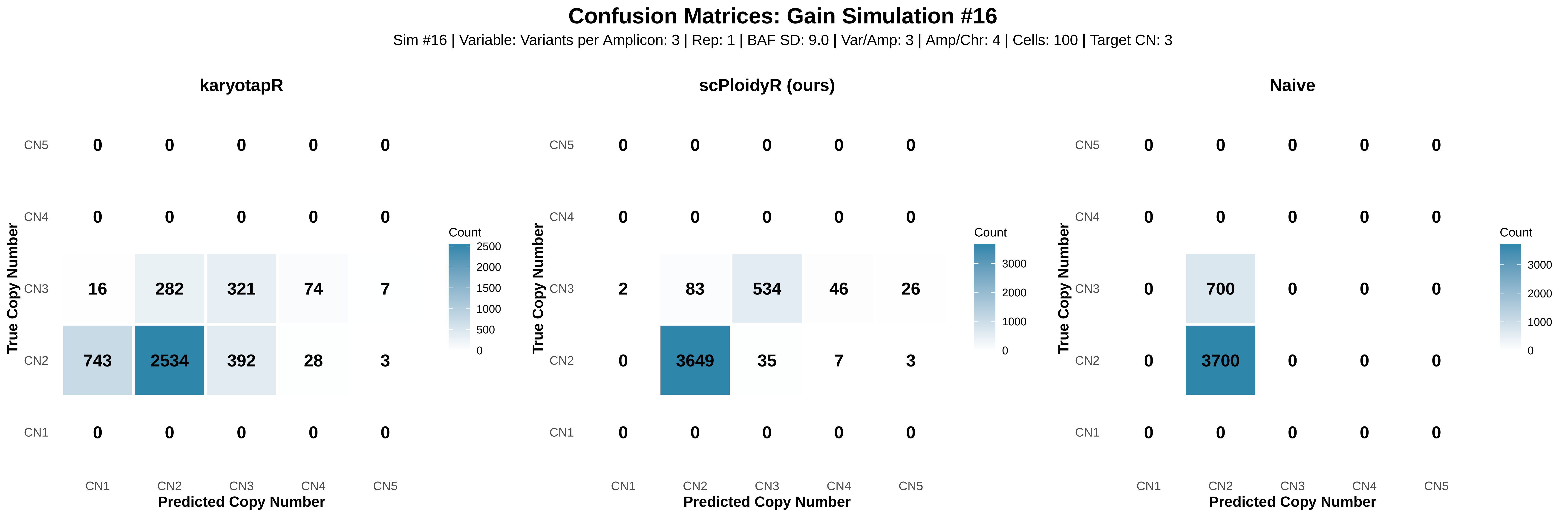
Confusion Matrices: Gain Simulation #16
Sim #16 | Variable: Variants per Amplicon: 3 | Rep: 1 | BAF SD: 9.0 | Var/Amp: 3 | Amp/Chr: 4 | Cells: 100 | Target CN: 3
karyotapR
scPloidyR (ours)
Naive
True Copy Number
Predicted Copy Number
CN1
CN2
CN3
CN4
CN5
Count
0
16
282
321
74
7
743
2534
392
28
3
2
83
534
46
26
3649
35
700
3700
500
1000
1500
2000
2500
3000

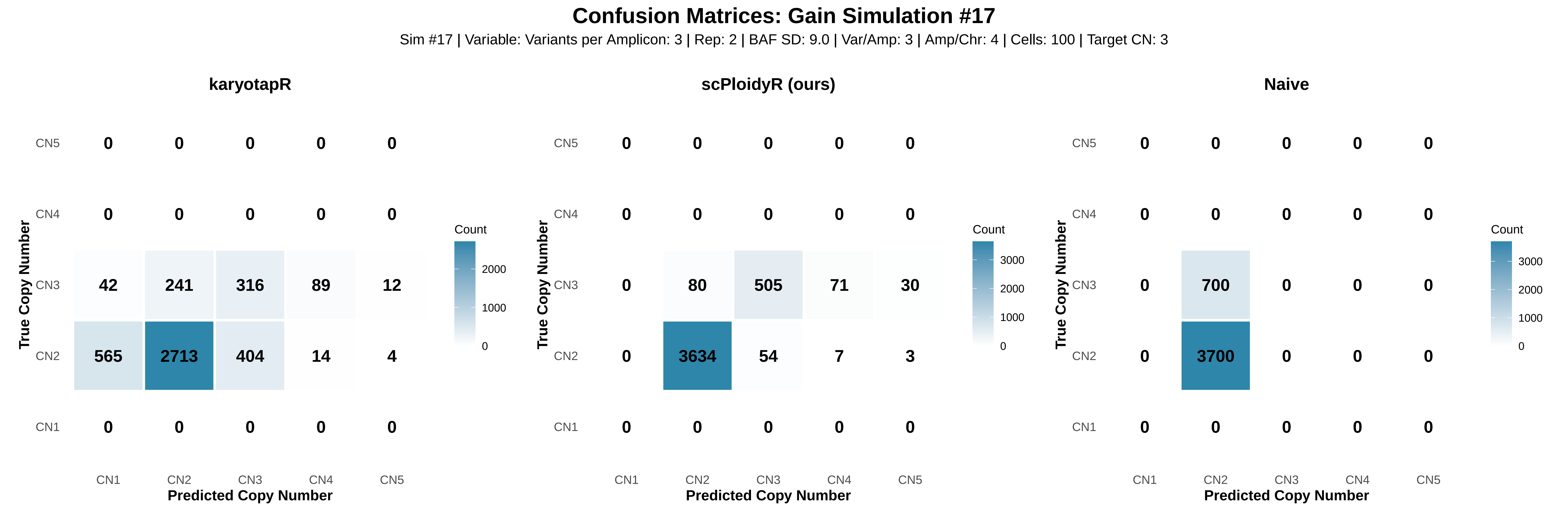
Confusion Matrices: Gain Simulation #17
Sim #17 | Variable: Variants per Amplicon: 3 | Rep: 2 | BAF SD: 9.0 | Var/Amp: 3 | Amp/Chr: 4 | Cells: 100 | Target CN: 3
karyotapR
scPloidyR (ours)
Naive
True Copy Number
Predicted Copy Number
Count
CN1
CN2
CN3
CN4
CN5
0
42
241
316
89
12
565
2713
404
14
4
80
505
71
30
3634
54
7
3
700
3700
1000
2000
3000

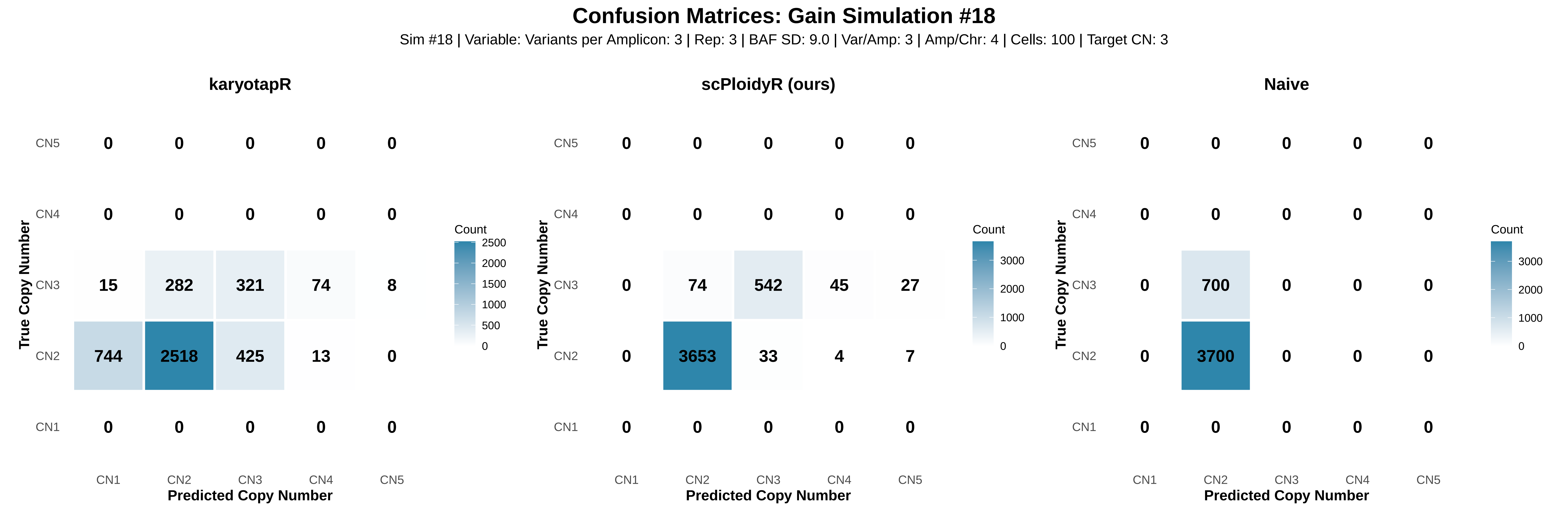
Confusion Matrices: Gain Simulation #18
Sim #18 | Variable: Variants per Amplicon: 3 | Rep: 3 | BAF SD: 9.0 | Var/Amp: 3 | Amp/Chr: 4 | Cells: 100 | Target CN: 3
karyotapR
scPloidyR (ours)
Naive
True Copy Number
Predicted Copy Number
CN1
CN2
CN3
CN4
CN5
Count
karyotapR: CN5: 0 0 0 0 0
CN4: 0 0 0 0 0
CN3: 15 282 321 74 8
CN2: 744 2518 425 13 0
CN1: 0 0 0 0 0
2500
2000
1500
1000
500
0
scPloidyR (ours): CN5: 0 0 0 0 0
CN4: 0 0 0 0 0
CN3: 0 74 542 45 27
CN2: 0 3653 33 4 7
CN1: 0 0 0 0 0
3000
2000
1000
0
Naive: CN5: 0 0 0 0 0
CN4: 0 0 0 0 0
CN3: 0 700 0 0 0
CN2: 0 3700 0 0 0
CN1: 0 0 0 0 0
3000
2000
1000
0

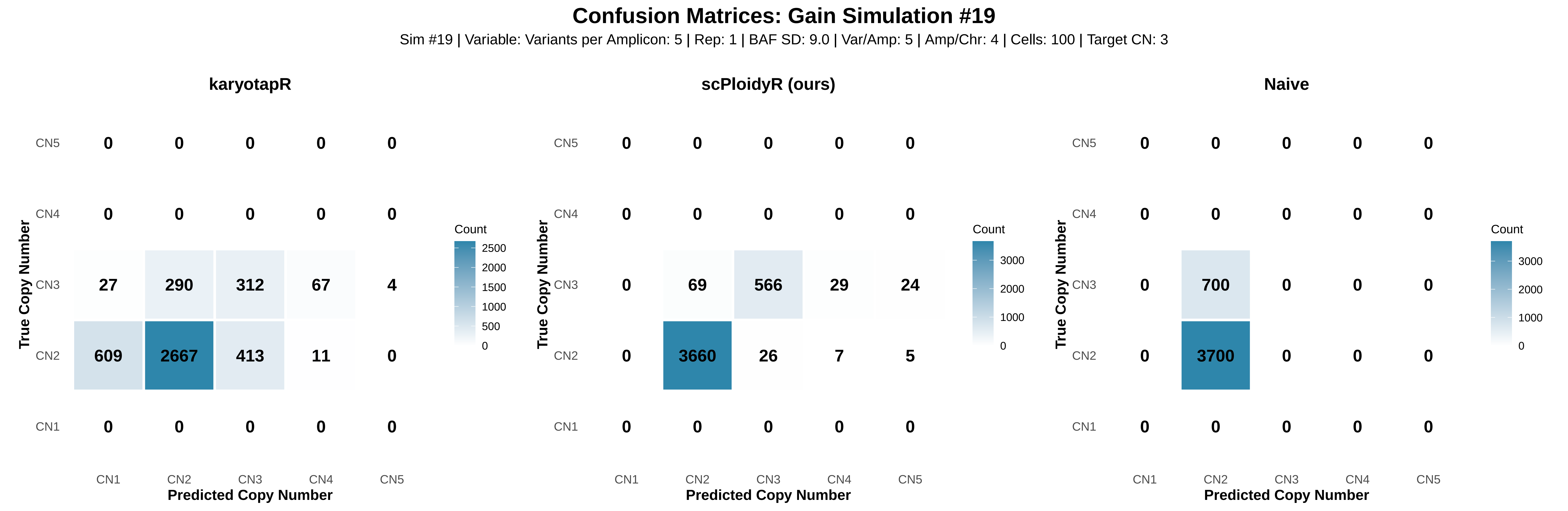

Confusion Matrices: Gain Simulation #19
Sim #19 | Variable: Variants per Amplicon: 5 | Rep: 1 | BAF SD: 9.0 | Var/Amp: 5 | Amp/Chr: 4 | Cells: 100 | Target CN: 3
karyotapR
scPloidyR (ours)
Naive
True Copy Number
Predicted Copy Number
CN1
CN2
CN3
CN4
CN5
Count
karyotapR: CN5: 0 0 0 0 0; CN4: 0 0 0 0 0; CN3: 27 290 312 67 4; CN2: 609 2667 413 11 0; CN1: 0 0 0 0 0
scPloidyR (ours): CN5: 0 0 0 0 0; CN4: 0 0 0 0 0; CN3: 0 69 566 29 24; CN2: 0 3660 26 7 5; CN1: 0 0 0 0 0
Naive: CN5: 0 0 0 0 0; CN4: 0 0 0 0 0; CN3: 0 700 0 0 0; CN2: 0 3700 0 0 0; CN1: 0 0 0 0 0
2500
2000
1500
1000
500
0
3000
2000
1000
0

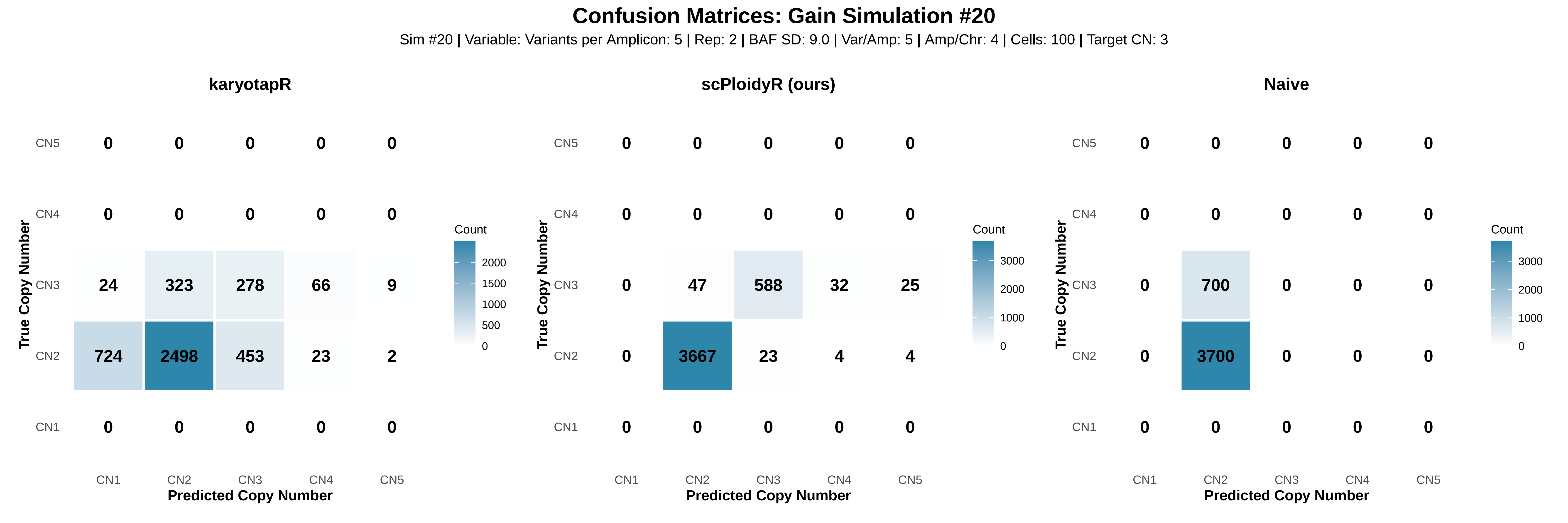
Confusion Matrices: Gain Simulation #20
Sim #20 | Variable: Variants per Amplicon: 5 | Rep: 2 | BAF SD: 9.0 | Var/Amp: 5 | Amp/Chr: 4 | Cells: 100 | Target CN: 3
karyotapR
CN5 0 0 0 0 0
CN4 0 0 0 0 0
CN3 24 323 278 66 9
CN2 724 2498 453 23 2
CN1 0 0 0 0 0
CN1 CN2 CN3 CN4 CN5
True Copy Number
Predicted Copy Number
Count
2000
1500
1000
500
0
scPloidyR (ours)
CN5 0 0 0 0 0
CN4 0 0 0 0 0
CN3 0 47 588 32 25
CN2 0 3667 23 4 4
CN1 0 0 0 0 0
CN1 CN2 CN3 CN4 CN5
True Copy Number
Predicted Copy Number
Count
3000
2000
1000
0
Naive
CN5 0 0 0 0 0
CN4 0 0 0 0 0
CN3 0 700 0 0 0
CN2 0 3700 0 0 0
CN1 0 0 0 0 0
CN1 CN2 CN3 CN4 CN5
True Copy Number
Predicted Copy Number
Count
3000
2000
1000
0

Confusion Matrices: Gain Simulation #21

Sim #21 | Variable: Variants per Amplicon: 5 | Rep: 3 | BAF SD: 9.0 | Var/Amp: 5 | Amp/Chr: 4 | Cells: 100 | Target CN: 3

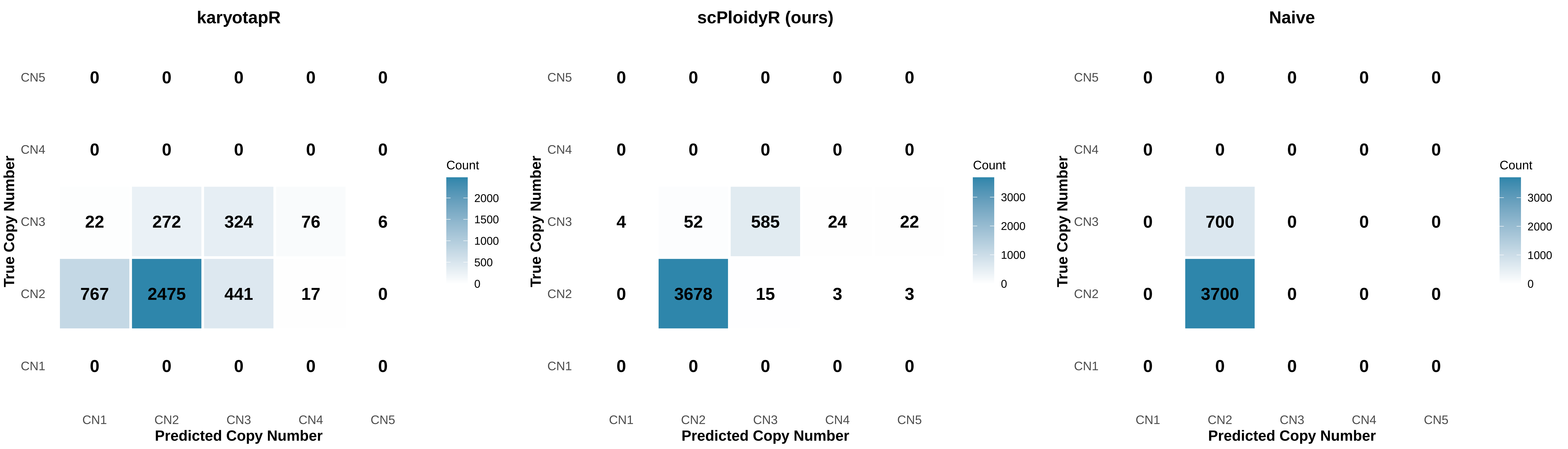

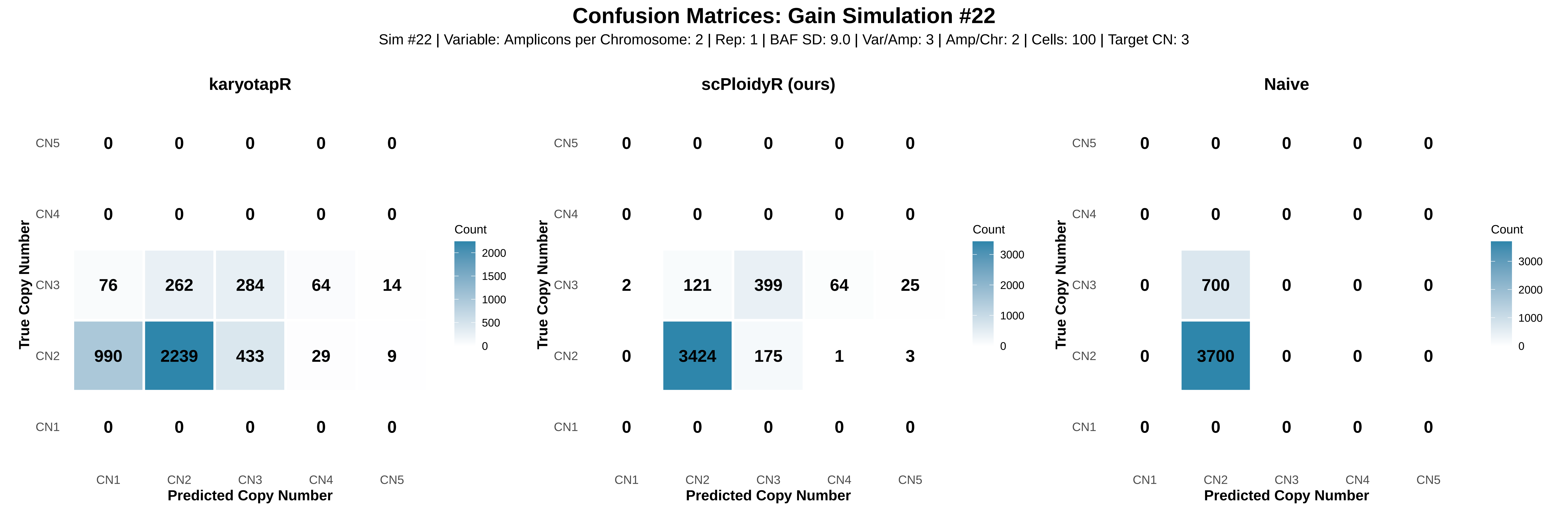

Confusion Matrices: Gain Simulation #22
Sim #22 | Variable: Amplicons per Chromosome: 2 | Rep: 1 | BAF SD: 9.0 | Var/Amp: 3 | Amp/Chr: 2 | Cells: 100 | Target CN: 3
karyotapR
scPloidyR (ours)
Naive
True Copy Number
Predicted Copy Number
Count
CN1
CN2
CN3
CN4
CN5
76
262
284
64
14
990
2239
433
29
9
2
121
399
64
25
0
3424
175
1
3
700
3700
0
500
1000
1500
2000
3000

Confusion Matrices: Gain Simulation #23

Sim #23 | Variable: Amplicons per Chromosome: 2 | Rep: 2 | BAF SD: 9.0 | Var/Amp: 3 | Amp/Chr: 2 | Cells: 100 | Target CN: 3

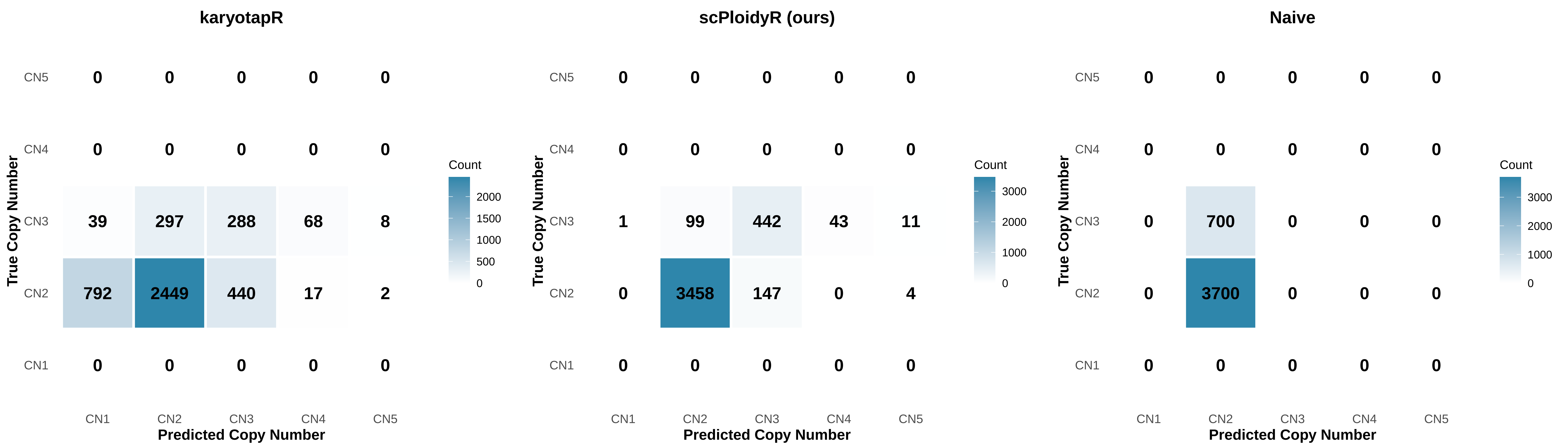

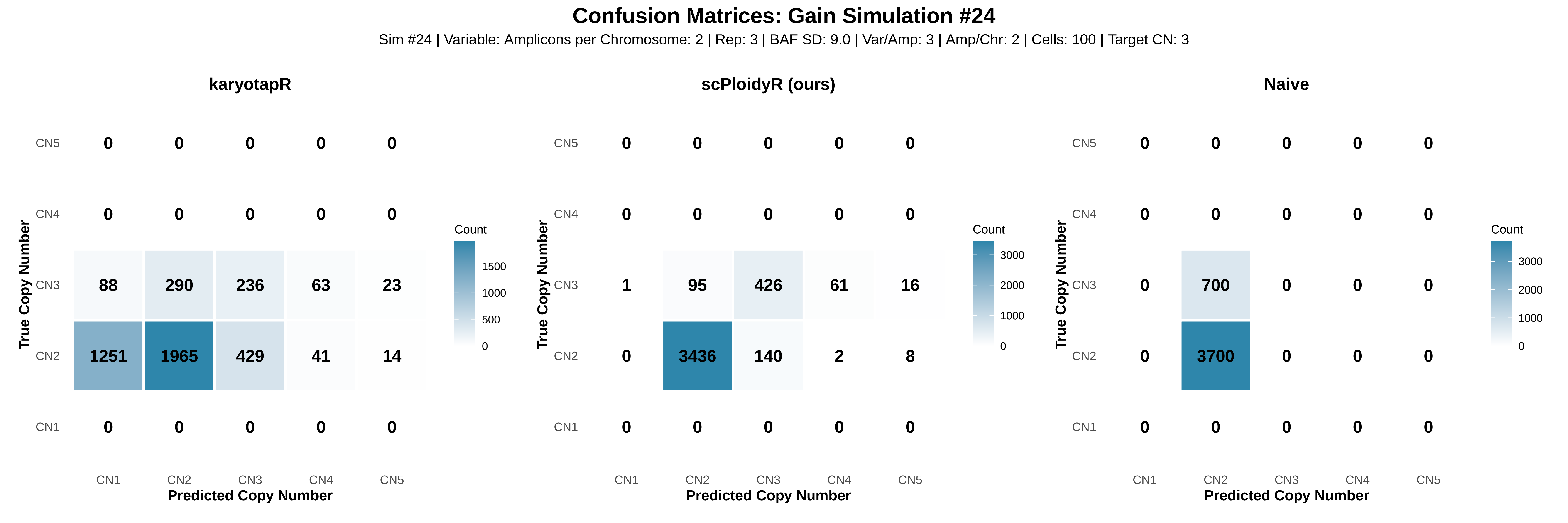

Confusion Matrices: Gain Simulation #24
Sim #24 | Variable: Amplicons per Chromosome: 2 | Rep: 3 | BAF SD: 9.0 | Var/Amp: 3 | Amp/Chr: 2 | Cells: 100 | Target CN: 3
karyotapR
scPloidyR (ours)
Naive
True Copy Number
Predicted Copy Number
CN1
CN2
CN3
CN4
CN5
Count
88
290
236
63
23
1251
1965
429
41
14
1
95
426
61
16
3436
140
2
8
700
3700

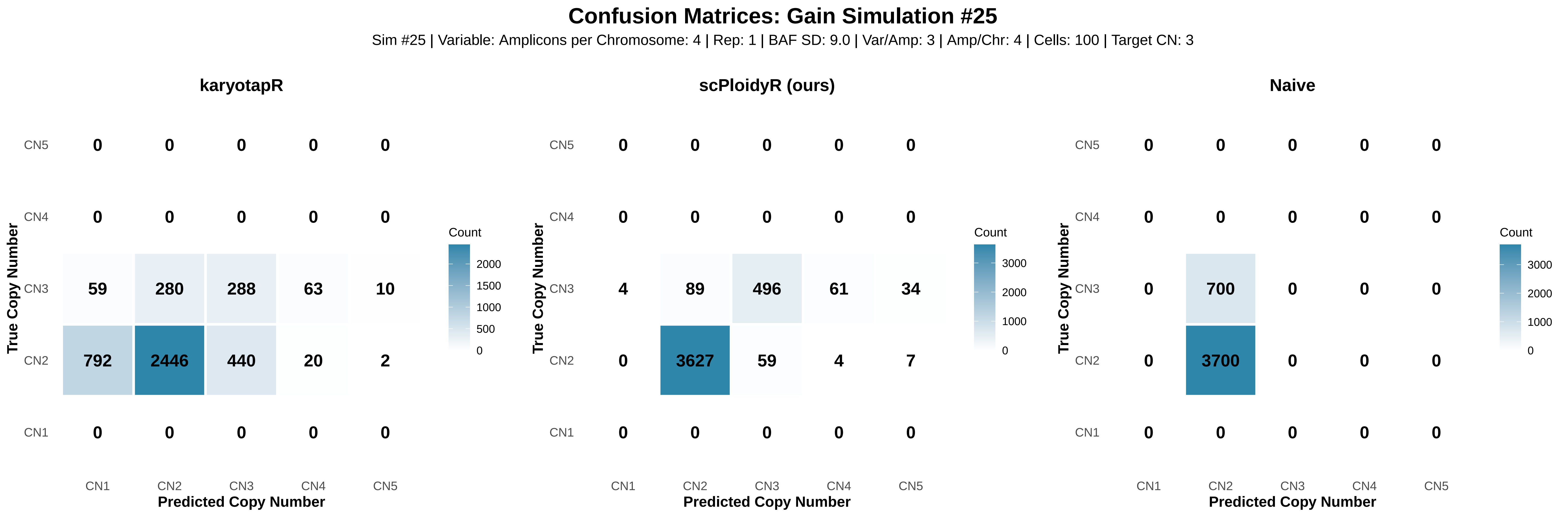
Confusion Matrices: Gain Simulation #25
Sim #25 | Variable: Amplicons per Chromosome: 4 | Rep: 1 | BAF SD: 9.0 | Var/Amp: 3 | Amp/Chr: 4 | Cells: 100 | Target CN: 3
karyotapR
scPloidyR (ours)
Naive
True Copy Number
Predicted Copy Number
Count
CN1
CN2
CN3
CN4
CN5
0
0
0
0
0
0
0
0
0
0
59
280
288
63
10
792
2446
440
20
2
0
0
0
0
0
0
0
0
0
0
0
0
0
0
0
4
89
496
61
34
0
3627
59
4
7
0
0
0
0
0
0
0
0
0
0
0
0
0
0
0
0
700
0
0
0
0
3700
0
0
0
0
0
0
0
0

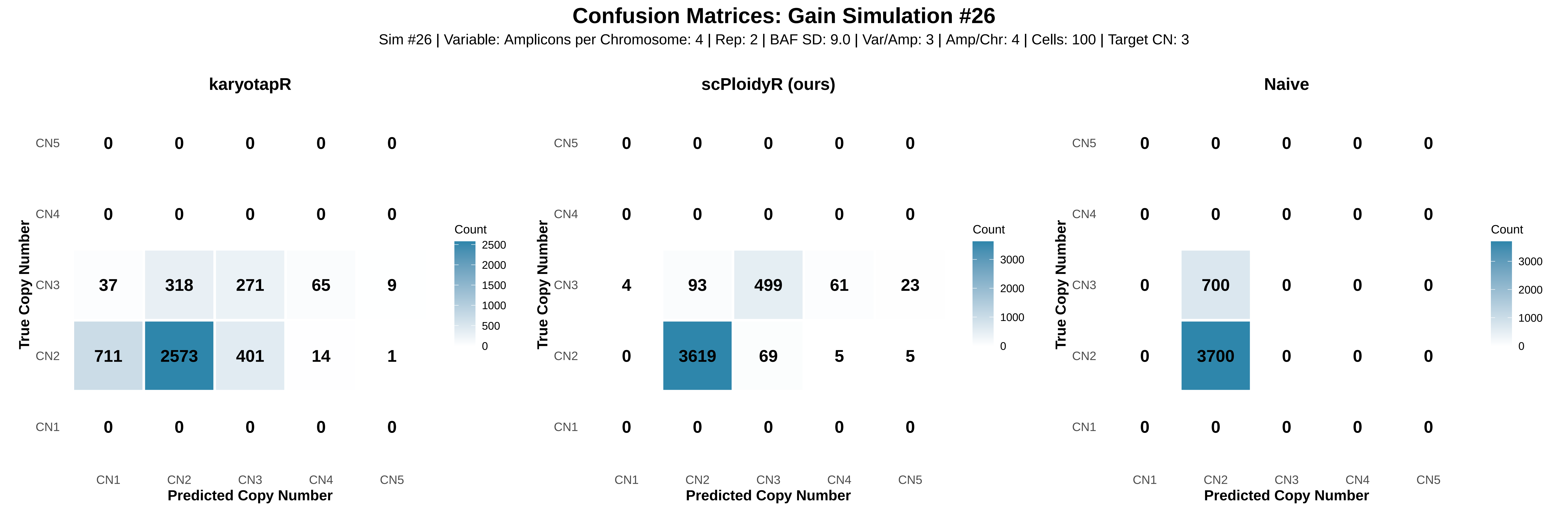
Confusion Matrices: Gain Simulation #26
Sim #26 | Variable: Amplicons per Chromosome: 4 | Rep: 2 | BAF SD: 9.0 | Var/Amp: 3 | Amp/Chr: 4 | Cells: 100 | Target CN: 3
karyotapR
scPloidyR (ours)
Naive
True Copy Number
Predicted Copy Number
CN1
CN2
CN3
CN4
CN5
Count
0
0
0
0
0
0
0
0
0
0
37
318
271
65
9
711
2573
401
14
1
0
0
0
0
0
4
93
499
61
23
0
3619
69
5
5
0
700
0
0
0
0
3700
0
0
0
2500
2000
1500
1000
500
3000

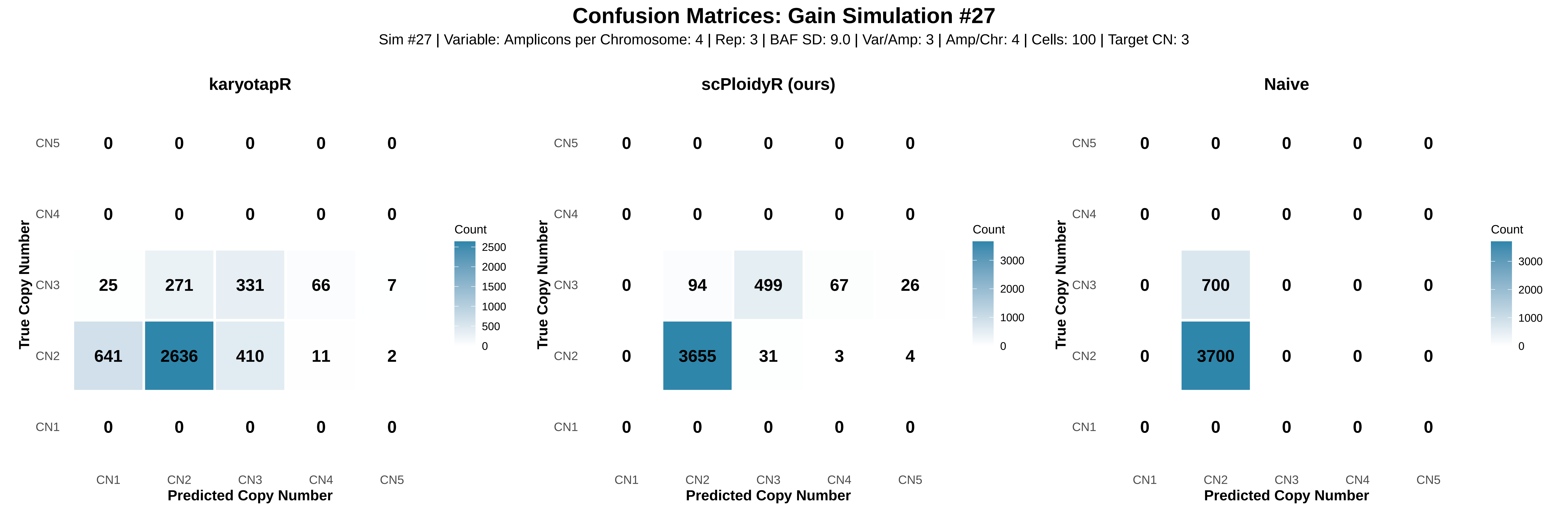
Confusion Matrices: Gain Simulation #27
Sim #27 | Variable: Amplicons per Chromosome: 4 | Rep: 3 | BAF SD: 9.0 | Var/Amp: 3 | Amp/Chr: 4 | Cells: 100 | Target CN: 3
karyotapR
scPloidyR (ours)
Naive
True Copy Number
Predicted Copy Number
CN1
CN2
CN3
CN4
CN5
Count
karyotapR CN3: 25 271 331 66 7
karyotapR CN2: 641 2636 410 11 2
scPloidyR CN3: 0 94 499 67 26
scPloidyR CN2: 0 3655 31 3 4
Naive CN3: 0 700 0 0 0
Naive CN2: 0 3700 0 0 0

Confusion Matrices: Gain Simulation #28

Sim #28 | Variable: Amplicons per Chromosome: 6 | Rep: 1 | BAF SD: 9.0 | Var/Amp: 3 | Amp/Chr: 6 | Cells: 100 | Target CN: 3

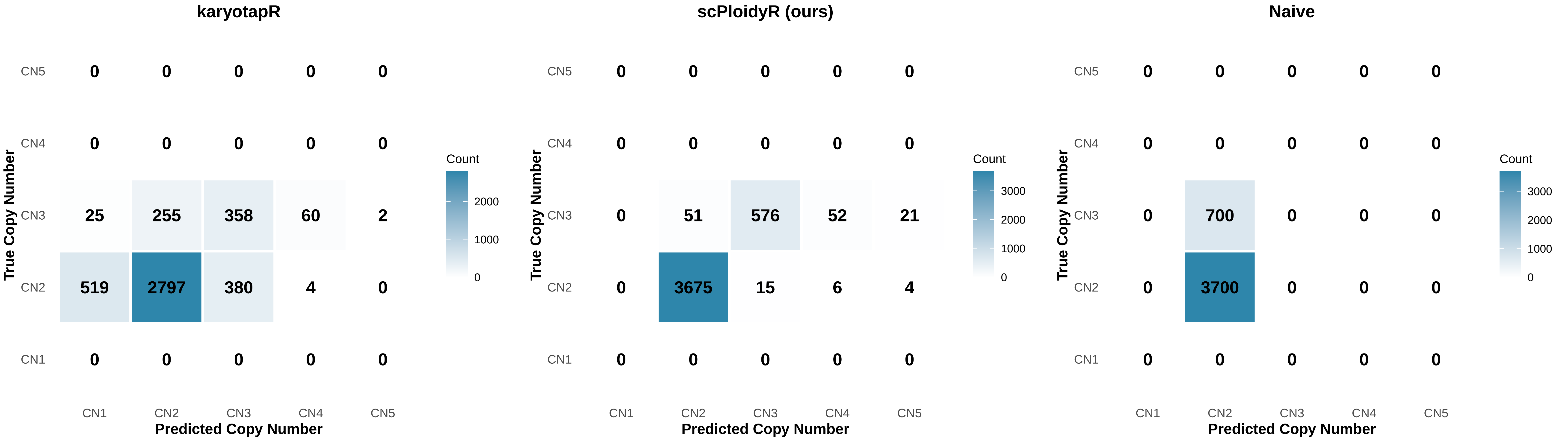

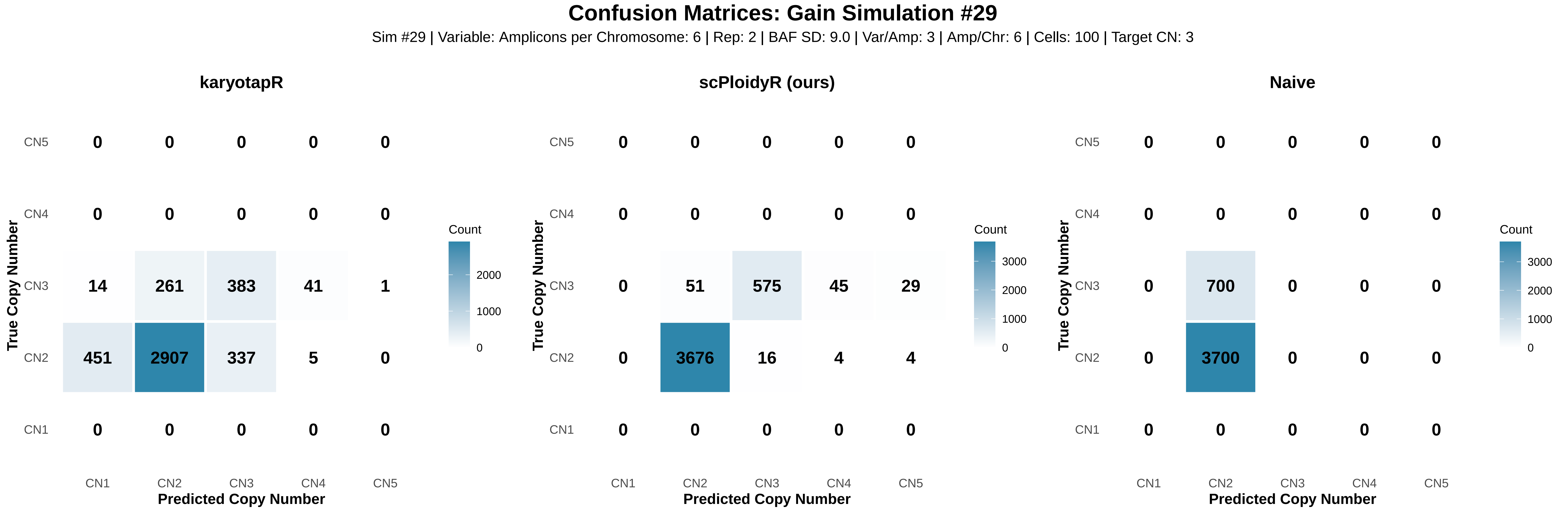
Confusion Matrices: Gain Simulation #29
Sim #29 | Variable: Amplicons per Chromosome: 6 | Rep: 2 | BAF SD: 9.0 | Var/Amp: 3 | Amp/Chr: 6 | Cells: 100 | Target CN: 3
karyotapR
CN5 0 0 0 0 0
CN4 0 0 0 0 0
CN3 14 261 383 41 1
CN2 451 2907 337 5 0
CN1 0 0 0 0 0
CN1 CN2 CN3 CN4 CN5
True Copy Number
Predicted Copy Number
Count
2000
1000
0
scPloidyR (ours)
CN5 0 0 0 0 0
CN4 0 0 0 0 0
CN3 0 51 575 45 29
CN2 0 3676 16 4 4
CN1 0 0 0 0 0
CN1 CN2 CN3 CN4 CN5
True Copy Number
Predicted Copy Number
Count
3000
2000
1000
0
Naive
CN5 0 0 0 0 0
CN4 0 0 0 0 0
CN3 0 700 0 0 0
CN2 0 3700 0 0 0
CN1 0 0 0 0 0
CN1 CN2 CN3 CN4 CN5
True Copy Number
Predicted Copy Number
Count
3000
2000
1000
0

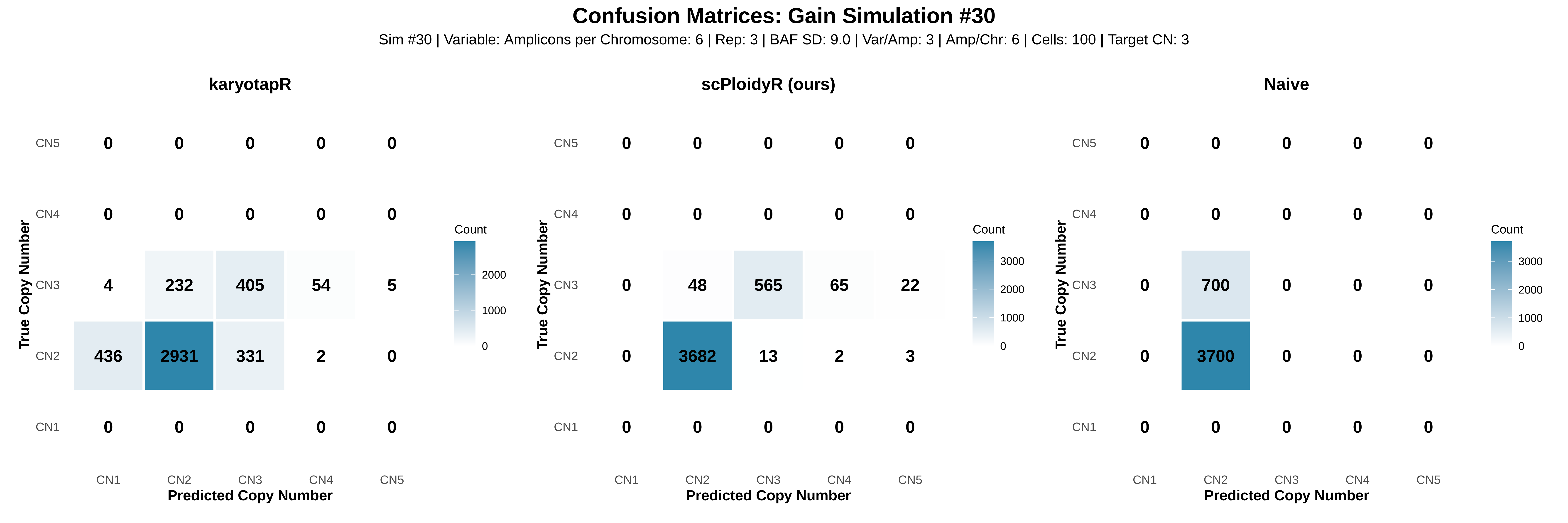
Confusion Matrices: Gain Simulation #30
Sim #30 | Variable: Amplicons per Chromosome: 6 | Rep: 3 | BAF SD: 9.0 | Var/Amp: 3 | Amp/Chr: 6 | Cells: 100 | Target CN: 3
karyotapR
scPloidyR (ours)
Naive
True Copy Number
Predicted Copy Number
CN1
CN2
CN3
CN4
CN5
Count
0
1000
2000
3000
karyotapR CN5: 0 0 0 0 0
karyotapR CN4: 0 0 0 0 0
karyotapR CN3: 4 232 405 54 5
karyotapR CN2: 436 2931 331 2 0
karyotapR CN1: 0 0 0 0 0
scPloidyR CN5: 0 0 0 0 0
scPloidyR CN4: 0 0 0 0 0
scPloidyR CN3: 0 48 565 65 22
scPloidyR CN2: 0 3682 13 2 3
scPloidyR CN1: 0 0 0 0 0
Naive CN5: 0 0 0 0 0
Naive CN4: 0 0 0 0 0
Naive CN3: 0 700 0 0 0
Naive CN2: 0 3700 0 0 0
Naive CN1: 0 0 0 0 0

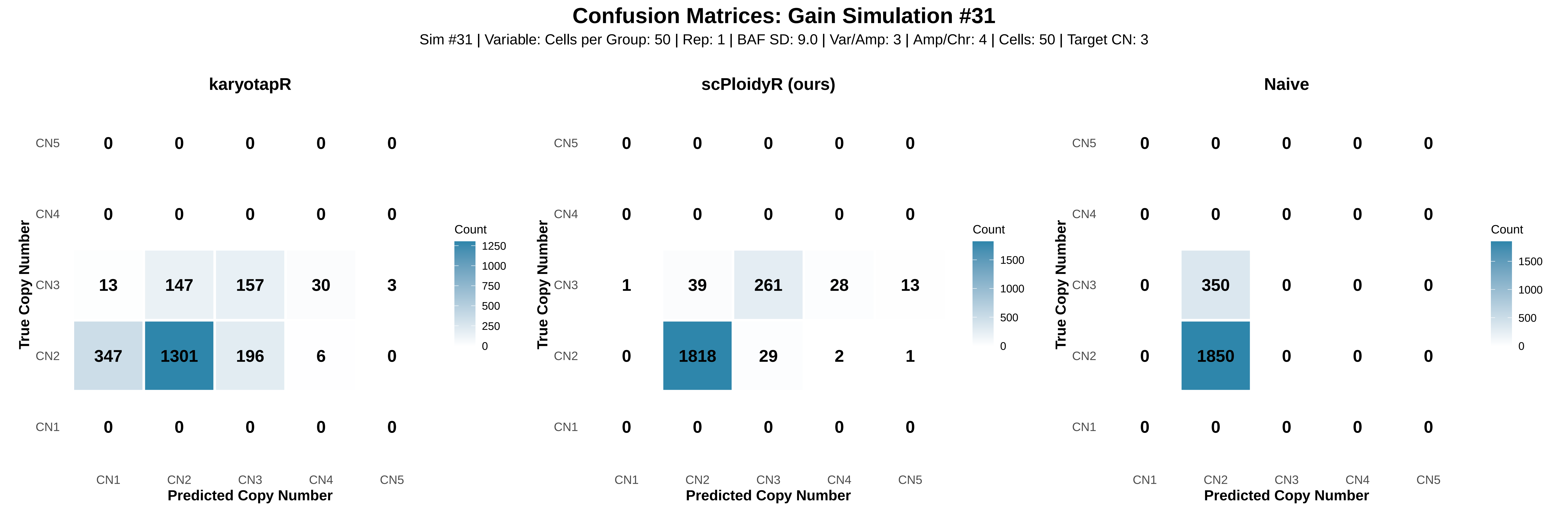

Confusion Matrices: Gain Simulation #31
Sim #31 | Variable: Cells per Group: 50 | Rep: 1 | BAF SD: 9.0 | Var/Amp: 3 | Amp/Chr: 4 | Cells: 50 | Target CN: 3
karyotapR
scPloidyR (ours)
Naive
True Copy Number
Predicted Copy Number
CN1
CN2
CN3
CN4
CN5
Count
1250
1000
750
500
250
0
1500
1000
500
0
karyotapR: CN5: 0 0 0 0 0; CN4: 0 0 0 0 0; CN3: 13 147 157 30 3; CN2: 347 1301 196 6 0; CN1: 0 0 0 0 0
scPloidyR (ours): CN5: 0 0 0 0 0; CN4: 0 0 0 0 0; CN3: 1 39 261 28 13; CN2: 0 1818 29 2 1; CN1: 0 0 0 0 0
Naive: CN5: 0 0 0 0 0; CN4: 0 0 0 0 0; CN3: 0 350 0 0 0; CN2: 0 1850 0 0 0; CN1: 0 0 0 0 0

Confusion Matrices: Gain Simulation #32

Sim #32 | Variable: Cells per Group: 50 | Rep: 2 | BAF SD: 9.0 | Var/Amp: 3 | Amp/Chr: 4 | Cells: 50 | Target CN: 3

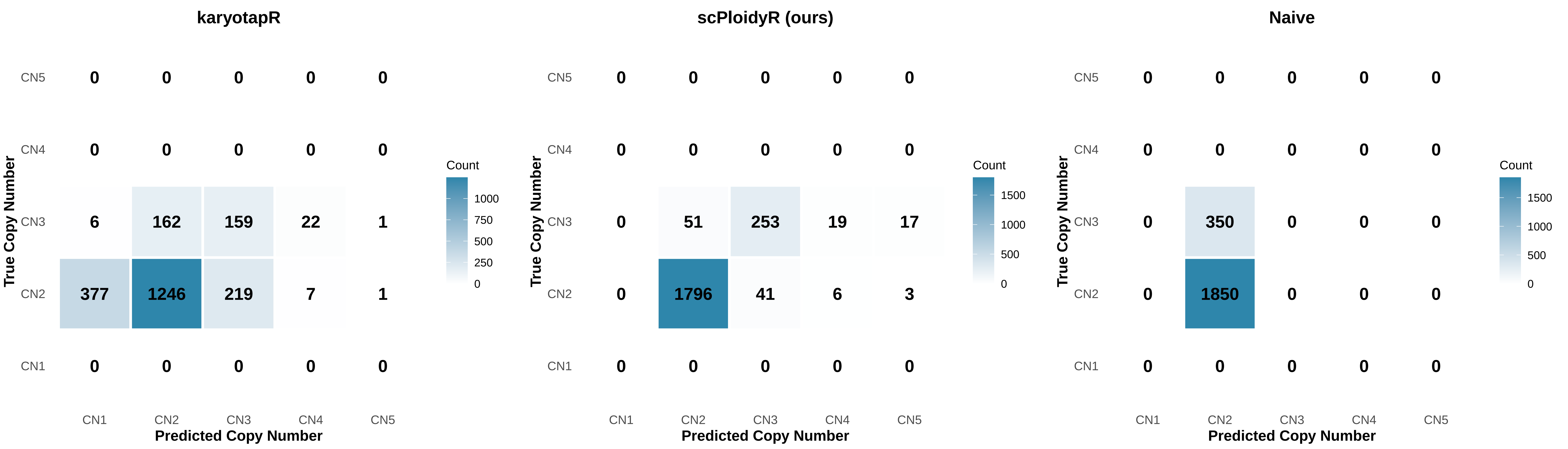

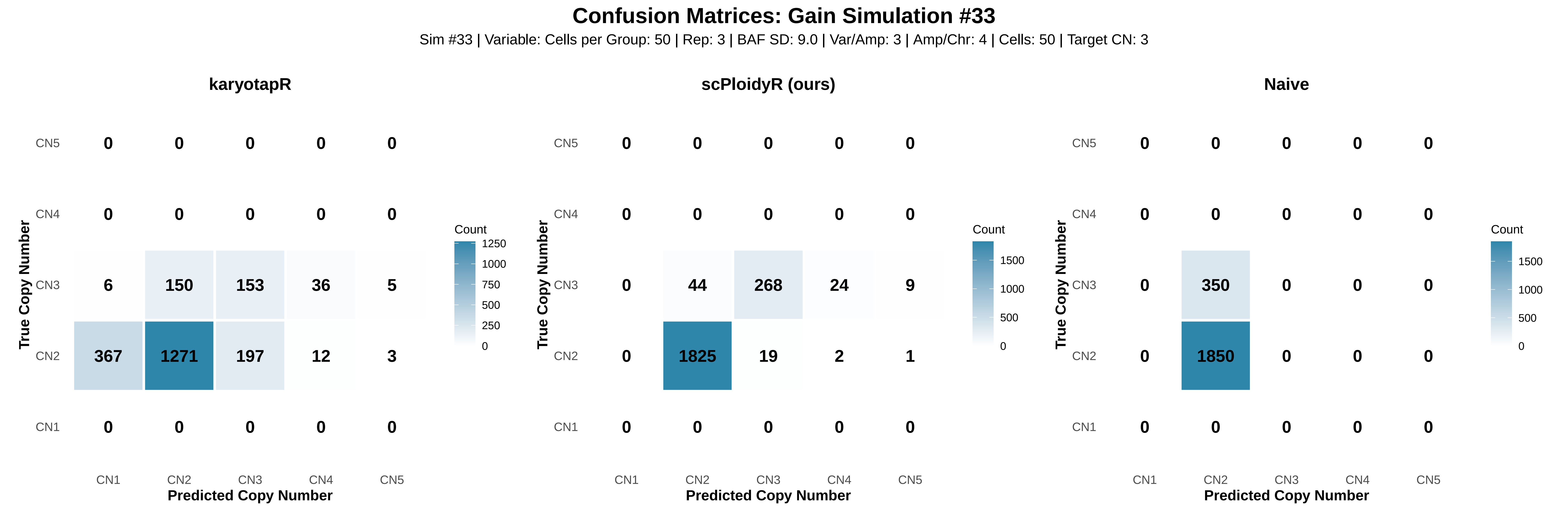
Confusion Matrices: Gain Simulation #33
Sim #33 | Variable: Cells per Group: 50 | Rep: 3 | BAF SD: 9.0 | Var/Amp: 3 | Amp/Chr: 4 | Cells: 50 | Target CN: 3
karyotapR
CN5 0 0 0 0 0
CN4 0 0 0 0 0
CN3 6 150 153 36 5
CN2 367 1271 197 12 3
CN1 0 0 0 0 0
CN1 CN2 CN3 CN4 CN5
Predicted Copy Number
True Copy Number
Count
1250
1000
750
500
250
0
scPloidyR (ours)
CN5 0 0 0 0 0
CN4 0 0 0 0 0
CN3 0 44 268 24 9
CN2 0 1825 19 2 1
CN1 0 0 0 0 0
CN1 CN2 CN3 CN4 CN5
Predicted Copy Number
True Copy Number
Count
1500
1000
500
0
Naive
CN5 0 0 0 0 0
CN4 0 0 0 0 0
CN3 0 350 0 0 0
CN2 0 1850 0 0 0
CN1 0 0 0 0 0
CN1 CN2 CN3 CN4 CN5
Predicted Copy Number
True Copy Number
Count
1500
1000
500
0

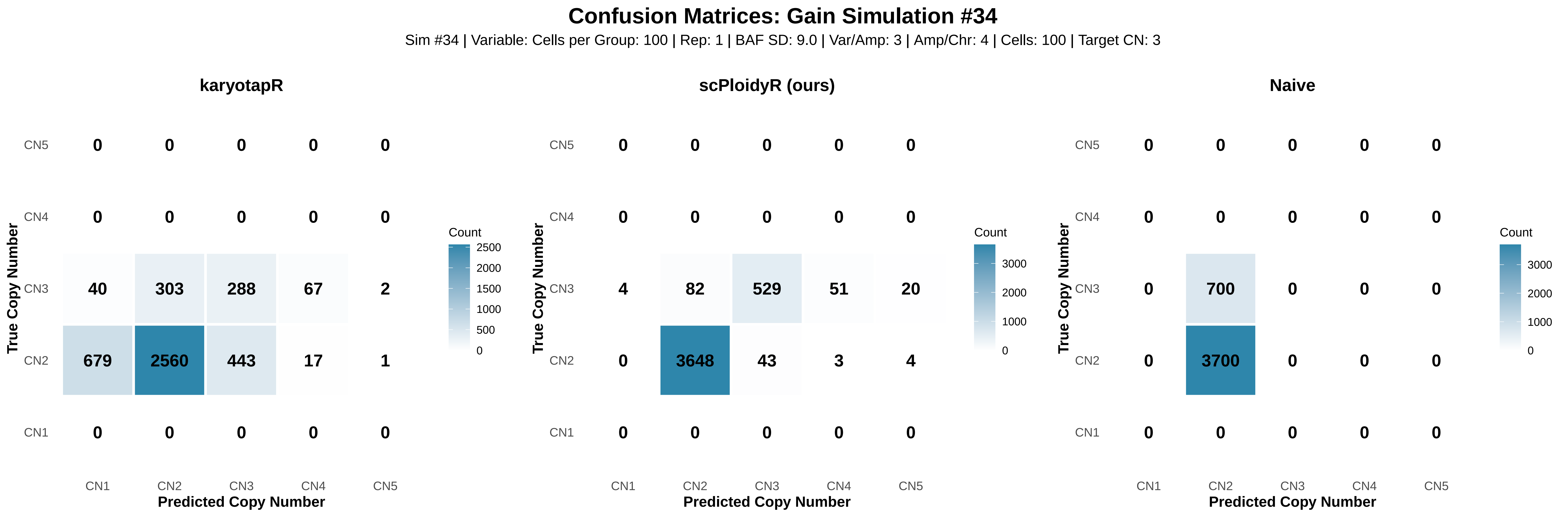

Confusion Matrices: Gain Simulation #34
Sim #34 | Variable: Cells per Group: 100 | Rep: 1 | BAF SD: 9.0 | Var/Amp: 3 | Amp/Chr: 4 | Cells: 100 | Target CN: 3
karyotapR
CN5 0 0 0 0 0
CN4 0 0 0 0 0
CN3 40 303 288 67 2
CN2 679 2560 443 17 1
CN1 0 0 0 0 0
CN1 CN2 CN3 CN4 CN5
Predicted Copy Number
True Copy Number
Count
2500
2000
1500
1000
500
0
scPloidyR (ours)
CN5 0 0 0 0 0
CN4 0 0 0 0 0
CN3 4 82 529 51 20
CN2 0 3648 43 3 4
CN1 0 0 0 0 0
CN1 CN2 CN3 CN4 CN5
Predicted Copy Number
True Copy Number
Count
3000
2000
1000
0
Naive
CN5 0 0 0 0 0
CN4 0 0 0 0 0
CN3 0 700 0 0 0
CN2 0 3700 0 0 0
CN1 0 0 0 0 0
CN1 CN2 CN3 CN4 CN5
Predicted Copy Number
True Copy Number
Count
3000
2000
1000
0

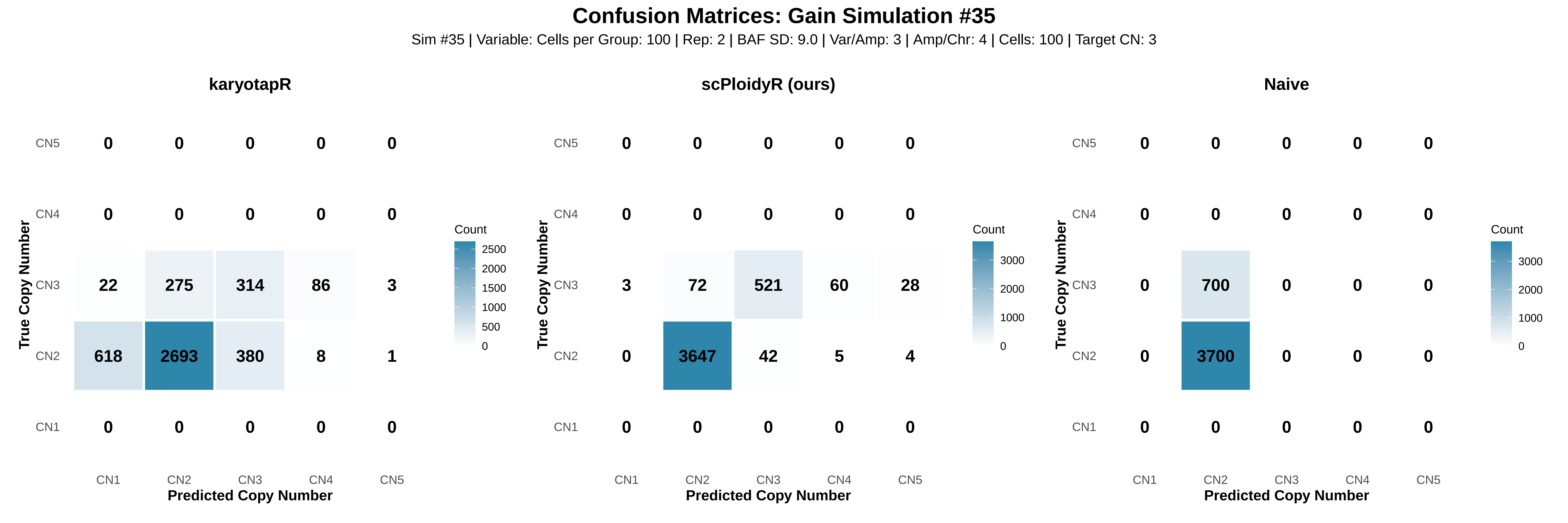
Confusion Matrices: Gain Simulation #35
Sim #35 | Variable: Cells per Group: 100 | Rep: 2 | BAF SD: 9.0 | Var/Amp: 3 | Amp/Chr: 4 | Cells: 100 | Target CN: 3
karyotapR
scPloidyR (ours)
Naive
True Copy Number
Predicted Copy Number
CN1
CN2
CN3
CN4
CN5
Count
CN5: 0 0 0 0 0
CN4: 0 0 0 0 0
CN3: 22 275 314 86 3
CN2: 618 2693 380 8 1
CN1: 0 0 0 0 0
CN5: 0 0 0 0 0
CN4: 0 0 0 0 0
CN3: 3 72 521 60 28
CN2: 0 3647 42 5 4
CN1: 0 0 0 0 0
CN5: 0 0 0 0 0
CN4: 0 0 0 0 0
CN3: 0 700 0 0 0
CN2: 0 3700 0 0 0
CN1: 0 0 0 0 0
2500
2000
1500
1000
500
0
3000
2000
1000
0

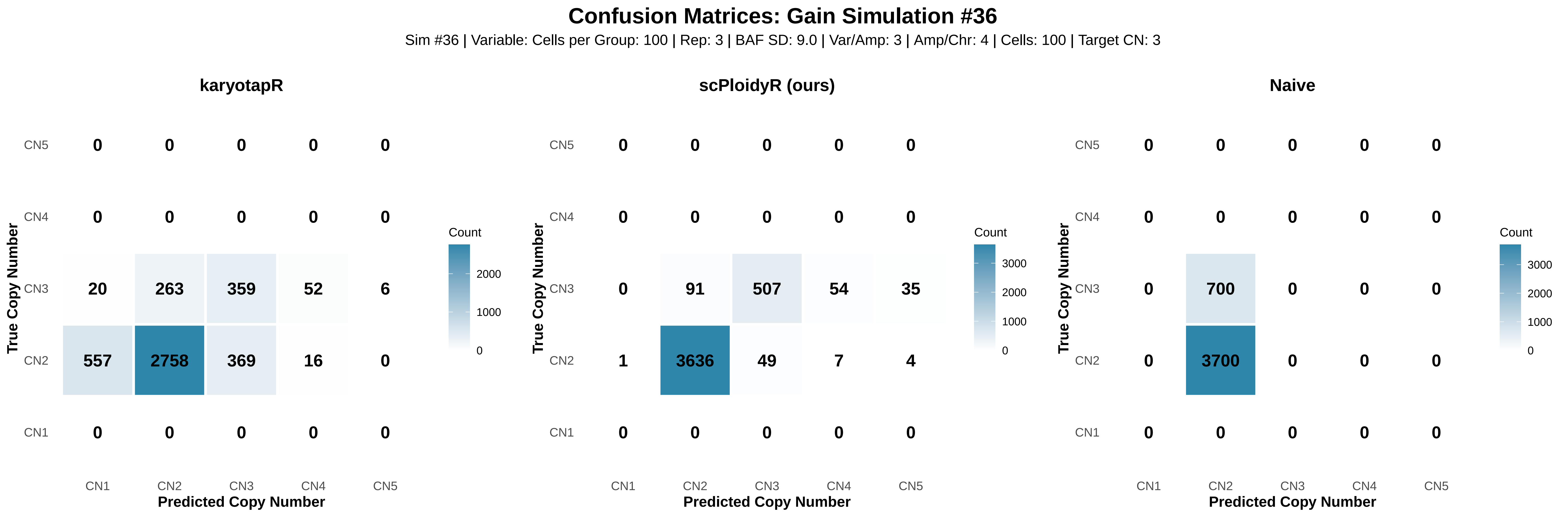
Confusion Matrices: Gain Simulation #36
Sim #36 | Variable: Cells per Group: 100 | Rep: 3 | BAF SD: 9.0 | Var/Amp: 3 | Amp/Chr: 4 | Cells: 100 | Target CN: 3
karyotapR
scPloidyR (ours)
Naive
True Copy Number
Predicted Copy Number
CN1
CN2
CN3
CN4
CN5
Count
0
1000
2000
3000
20
263
359
52
6
557
2758
369
16
91
507
54
35
1
3636
49
7
4
700
3700

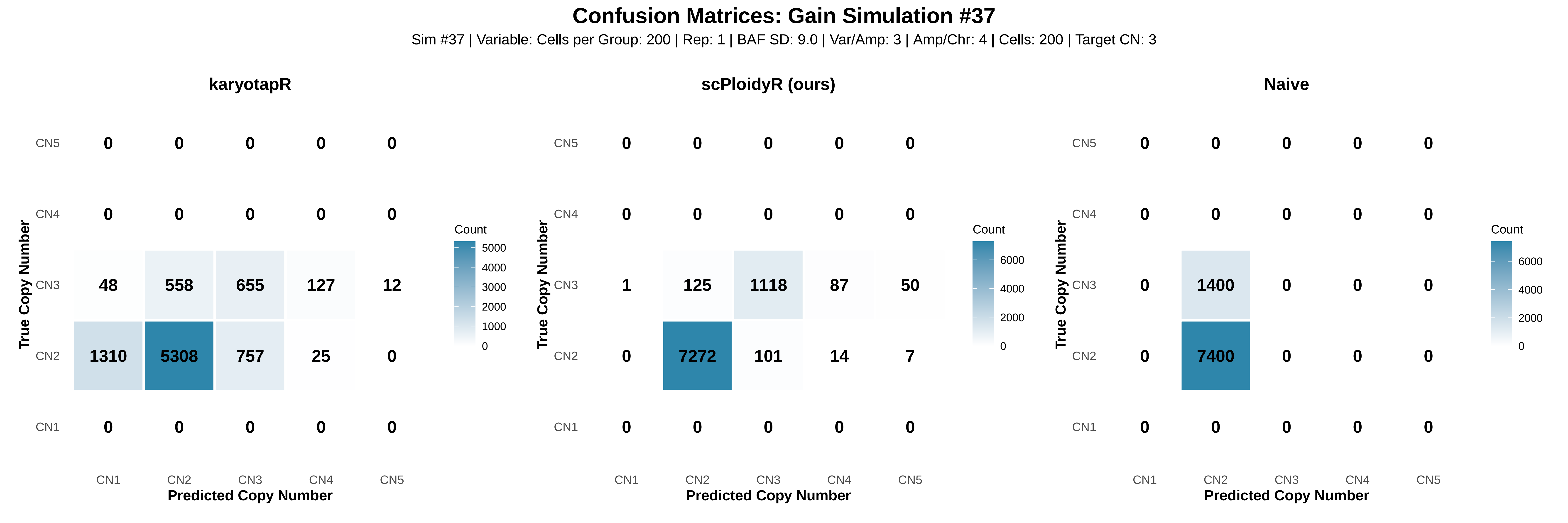
Confusion Matrices: Gain Simulation #37
Sim #37 | Variable: Cells per Group: 200 | Rep: 1 | BAF SD: 9.0 | Var/Amp: 3 | Amp/Chr: 4 | Cells: 200 | Target CN: 3
karyotapR
scPloidyR (ours)
Naive
True Copy Number
Predicted Copy Number
CN1
CN2
CN3
CN4
CN5
Count
0 0 0 0 0
0 0 0 0 0
48 558 655 127 12
1310 5308 757 25 0
0 0 0 0 0
1 125 1118 87 50
0 7272 101 14 7
0 1400 0 0 0
0 7400 0 0 0

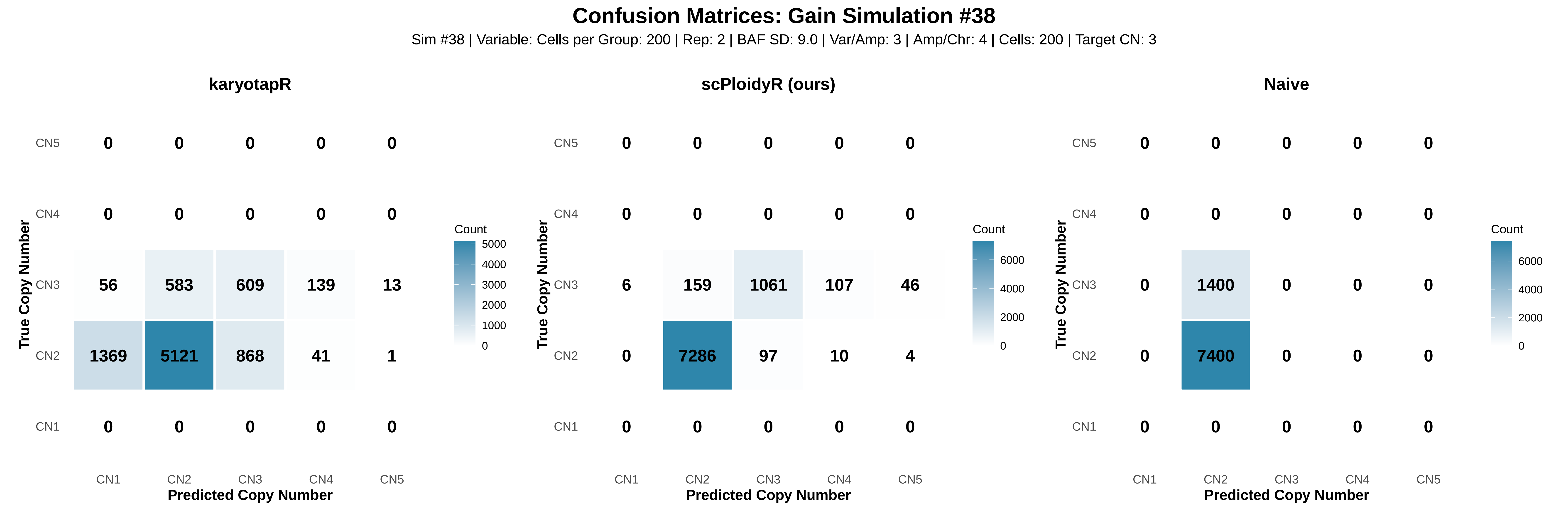
Confusion Matrices: Gain Simulation #38
Sim #38 | Variable: Cells per Group: 200 | Rep: 2 | BAF SD: 9.0 | Var/Amp: 3 | Amp/Chr: 4 | Cells: 200 | Target CN: 3
karyotapR
scPloidyR (ours)
Naive
True Copy Number
Predicted Copy Number
CN1
CN2
CN3
CN4
CN5
Count
0
1000
2000
3000
4000
5000
6000
0
0
0
0
0
56
583
609
139
13
1369
5121
868
41
1
6
159
1061
107
46
7286
97
10
4
1400
7400

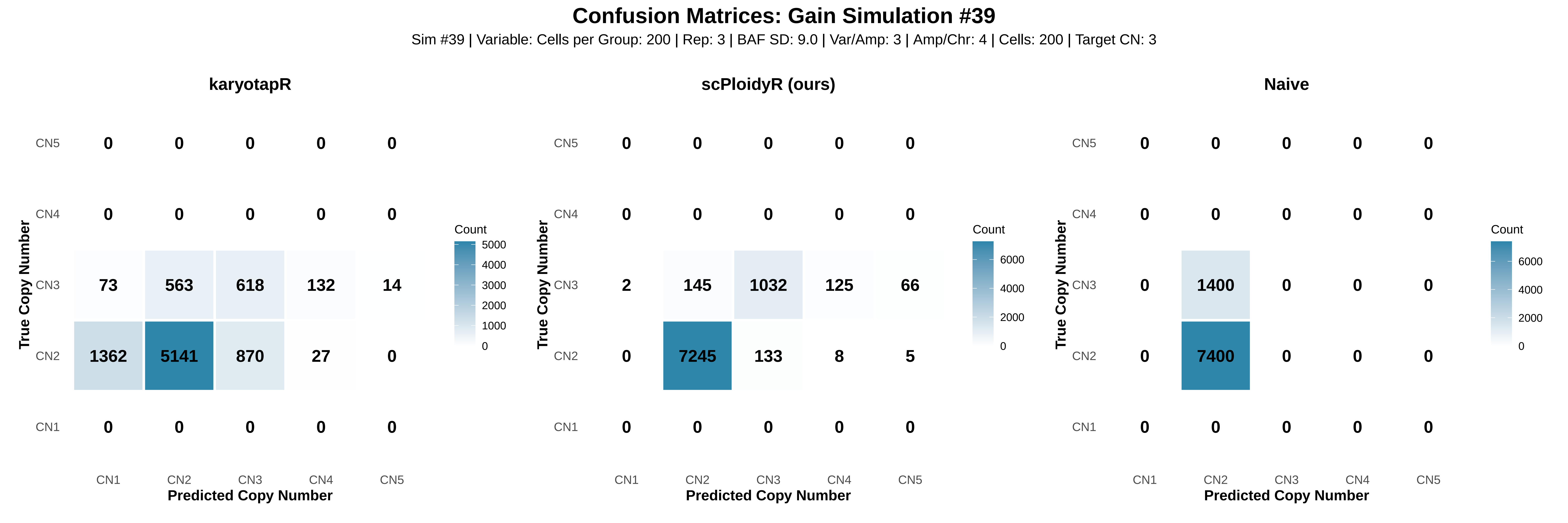
Confusion Matrices: Gain Simulation #39
Sim #39 | Variable: Cells per Group: 200 | Rep: 3 | BAF SD: 9.0 | Var/Amp: 3 | Amp/Chr: 4 | Cells: 200 | Target CN: 3
karyotapR
scPloidyR (ours)
Naive
True Copy Number
Predicted Copy Number
CN1
CN2
CN3
CN4
CN5
Count
karyotapR: CN3 row: 73 563 618 132 14; CN2 row: 1362 5141 870 27 0; CN5, CN4, CN1 rows: 0 0 0 0 0
scPloidyR (ours): CN3 row: 2 145 1032 125 66; CN2 row: 0 7245 133 8 5; CN5, CN4, CN1 rows: 0 0 0 0 0
Naive: CN3 row: 0 1400 0 0 0; CN2 row: 0 7400 0 0 0; CN5, CN4, CN1 rows: 0 0 0 0 0
0
1000
2000
3000
4000
5000
6000

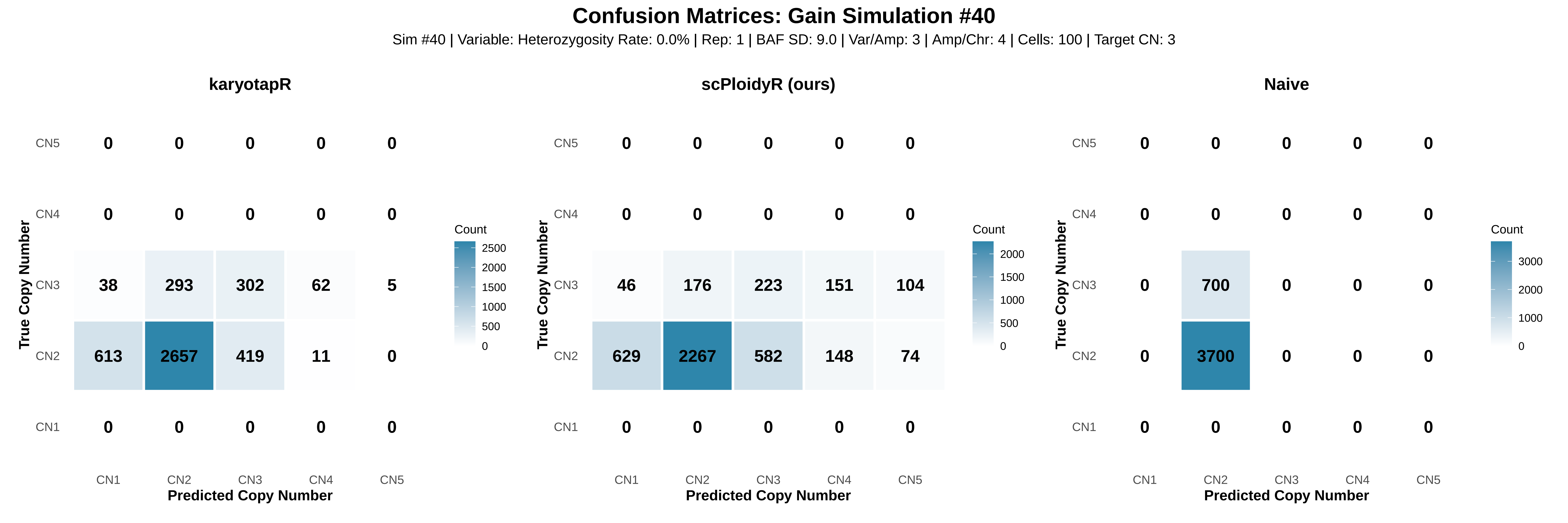
Confusion Matrices: Gain Simulation #40
Sim #40 | Variable: Heterozygosity Rate: 0.0% | Rep: 1 | BAF SD: 9.0 | Var/Amp: 3 | Amp/Chr: 4 | Cells: 100 | Target CN: 3
karyotapR
CN5 0 0 0 0 0
CN4 0 0 0 0 0
CN3 38 293 302 62 5
CN2 613 2657 419 11 0
CN1 0 0 0 0 0
CN1 CN2 CN3 CN4 CN5
Predicted Copy Number
True Copy Number
Count
2500
2000
1500
1000
500
0
scPloidyR (ours)
CN5 0 0 0 0 0
CN4 0 0 0 0 0
CN3 46 176 223 151 104
CN2 629 2267 582 148 74
CN1 0 0 0 0 0
CN1 CN2 CN3 CN4 CN5
Predicted Copy Number
True Copy Number
Count
2000
1500
1000
500
0
Naive
CN5 0 0 0 0 0
CN4 0 0 0 0 0
CN3 0 700 0 0 0
CN2 0 3700 0 0 0
CN1 0 0 0 0 0
CN1 CN2 CN3 CN4 CN5
Predicted Copy Number
True Copy Number
Count
3000
2000
1000
0

Confusion Matrices: Gain Simulation #41

Sim #41 | Variable: Heterozygosity Rate: 0.0% | Rep: 2 | BAF SD: 9.0 | Var/Amp: 3 | Amp/Chr: 4 | Cells: 100 | Target CN: 3

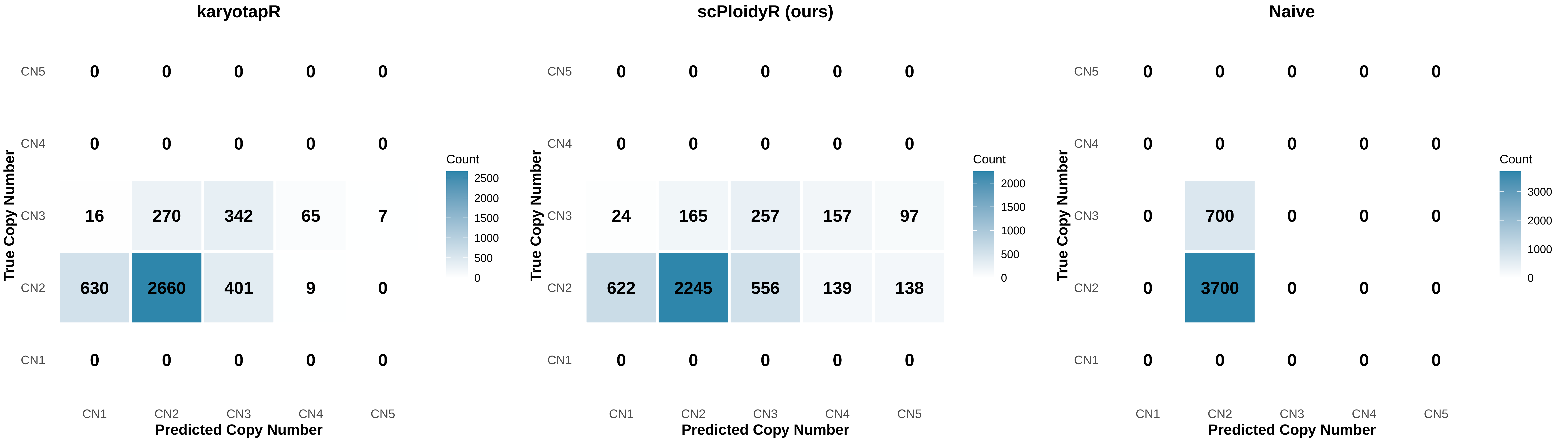

Confusion Matrices: Gain Simulation #42

Sim #42 | Variable: Heterozygosity Rate: 0.0% | Rep: 3 | BAF SD: 9.0 | Var/Amp: 3 | Amp/Chr: 4 | Cells: 100 | Target CN: 3

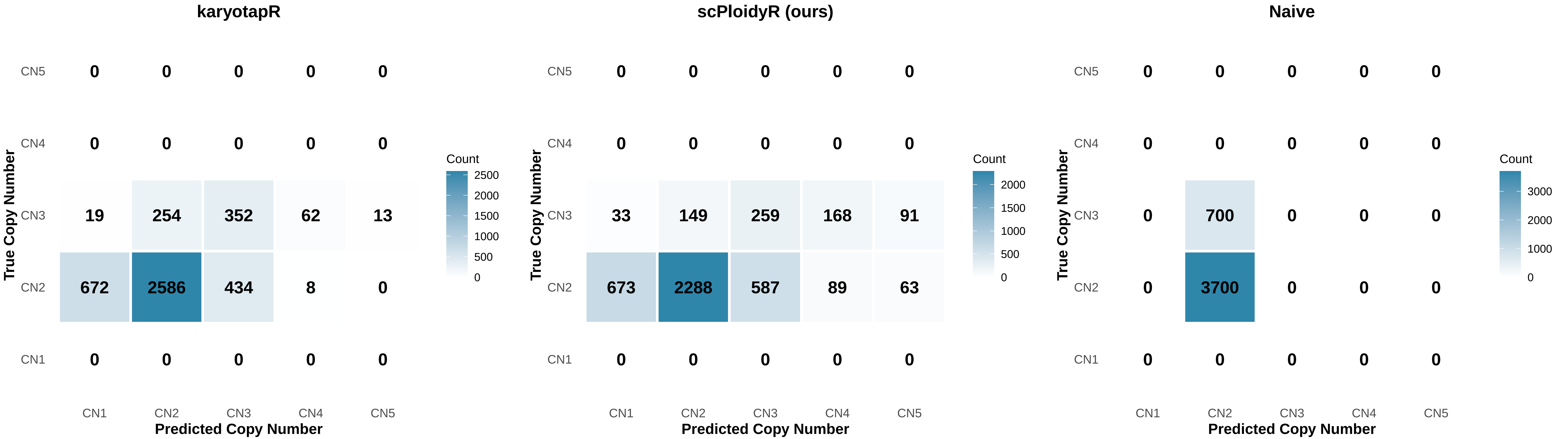

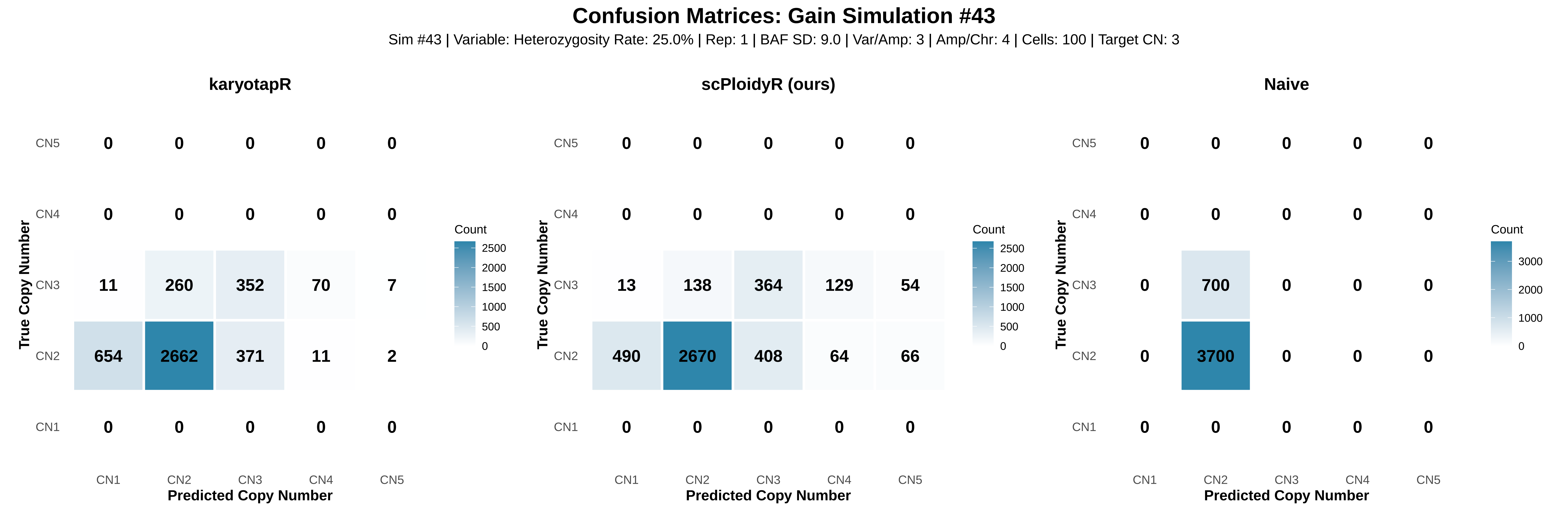
Confusion Matrices: Gain Simulation #43
Sim #43 | Variable: Heterozygosity Rate: 25.0% | Rep: 1 | BAF SD: 9.0 | Var/Amp: 3 | Amp/Chr: 4 | Cells: 100 | Target CN: 3
karyotapR
scPloidyR (ours)
Naive
True Copy Number
Predicted Copy Number
CN1
CN2
CN3
CN4
CN5
Count
karyotapR CN5: 0 0 0 0 0
karyotapR CN4: 0 0 0 0 0
karyotapR CN3: 11 260 352 70 7
karyotapR CN2: 654 2662 371 11 2
karyotapR CN1: 0 0 0 0 0
scPloidyR CN5: 0 0 0 0 0
scPloidyR CN4: 0 0 0 0 0
scPloidyR CN3: 13 138 364 129 54
scPloidyR CN2: 490 2670 408 64 66
scPloidyR CN1: 0 0 0 0 0
Naive CN5: 0 0 0 0 0
Naive CN4: 0 0 0 0 0
Naive CN3: 0 700 0 0 0
Naive CN2: 0 3700 0 0 0
Naive CN1: 0 0 0 0 0
2500
2000
1500
1000
500
0
3000

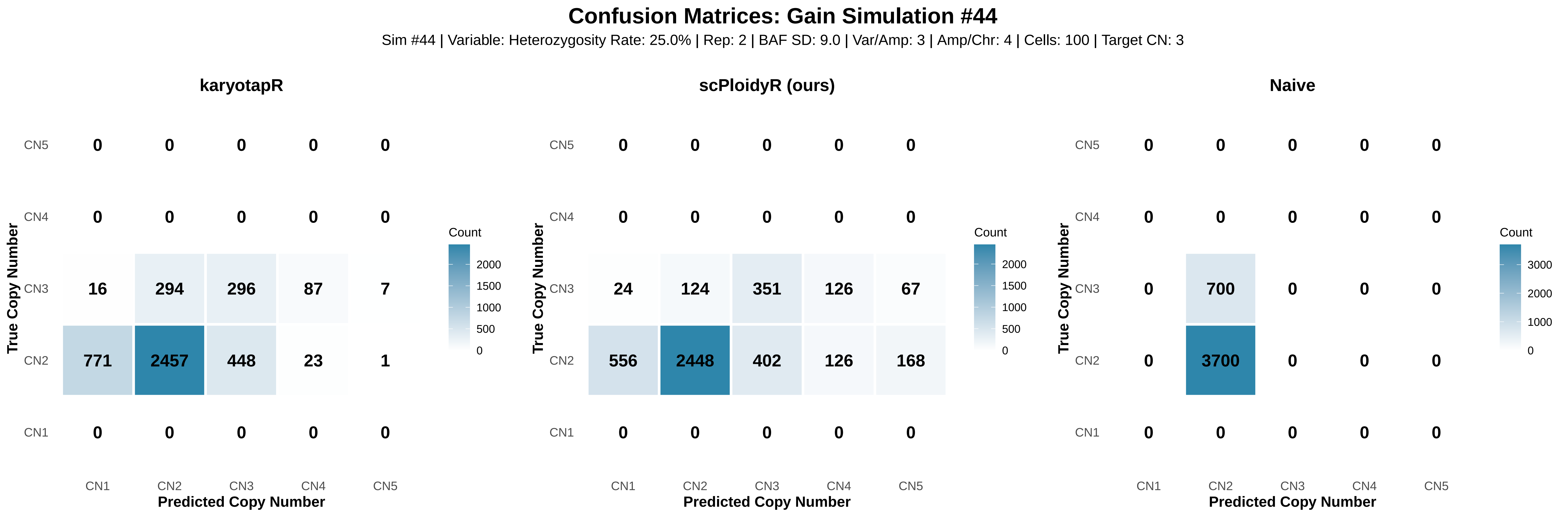
Confusion Matrices: Gain Simulation #44
Sim #44 | Variable: Heterozygosity Rate: 25.0% | Rep: 2 | BAF SD: 9.0 | Var/Amp: 3 | Amp/Chr: 4 | Cells: 100 | Target CN: 3
karyotapR
CN5 0 0 0 0 0
CN4 0 0 0 0 0
CN3 16 294 296 87 7
CN2 771 2457 448 23 1
CN1 0 0 0 0 0
CN1 CN2 CN3 CN4 CN5
True Copy Number
Predicted Copy Number
Count
2000
1500
1000
500
0
scPloidyR (ours)
CN5 0 0 0 0 0
CN4 0 0 0 0 0
CN3 24 124 351 126 67
CN2 556 2448 402 126 168
CN1 0 0 0 0 0
CN1 CN2 CN3 CN4 CN5
True Copy Number
Predicted Copy Number
Count
2000
1500
1000
500
0
Naive
CN5 0 0 0 0 0
CN4 0 0 0 0 0
CN3 0 700 0 0 0
CN2 0 3700 0 0 0
CN1 0 0 0 0 0
CN1 CN2 CN3 CN4 CN5
True Copy Number
Predicted Copy Number
Count
3000
2000
1000
0

Confusion Matrices: Gain Simulation #45

Sim #45 | Variable: Heterozygosity Rate: 25.0% | Rep: 3 | BAF SD: 9.0 | Var/Amp: 3 | Amp/Chr: 4 | Cells: 100 | Target CN: 3

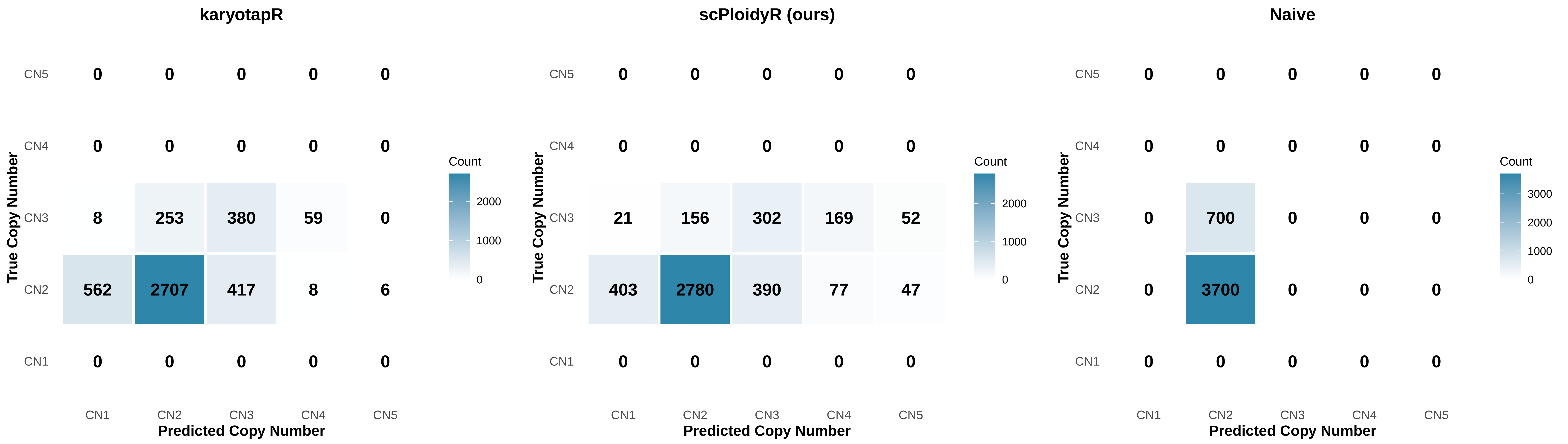

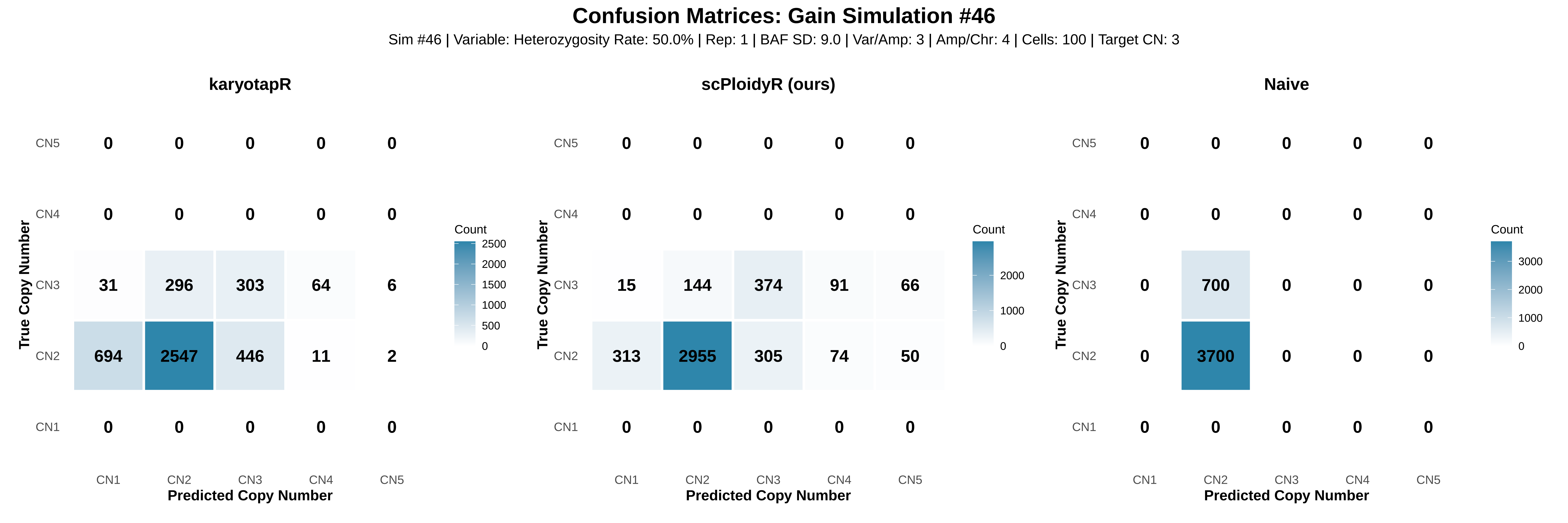
Confusion Matrices: Gain Simulation #46
Sim #46 | Variable: Heterozygosity Rate: 50.0% | Rep: 1 | BAF SD: 9.0 | Var/Amp: 3 | Amp/Chr: 4 | Cells: 100 | Target CN: 3
karyotapR
CN5 0 0 0 0 0
CN4 0 0 0 0 0
CN3 31 296 303 64 6
CN2 694 2547 446 11 2
CN1 0 0 0 0 0
CN1 CN2 CN3 CN4 CN5
Predicted Copy Number
True Copy Number
Count
2500
2000
1500
1000
500
0
scPloidyR (ours)
CN5 0 0 0 0 0
CN4 0 0 0 0 0
CN3 15 144 374 91 66
CN2 313 2955 305 74 50
CN1 0 0 0 0 0
CN1 CN2 CN3 CN4 CN5
Predicted Copy Number
True Copy Number
Count
2000
1000
0
Naive
CN5 0 0 0 0 0
CN4 0 0 0 0 0
CN3 0 700 0 0 0
CN2 0 3700 0 0 0
CN1 0 0 0 0 0
CN1 CN2 CN3 CN4 CN5
Predicted Copy Number
True Copy Number
Count
3000
2000
1000
0

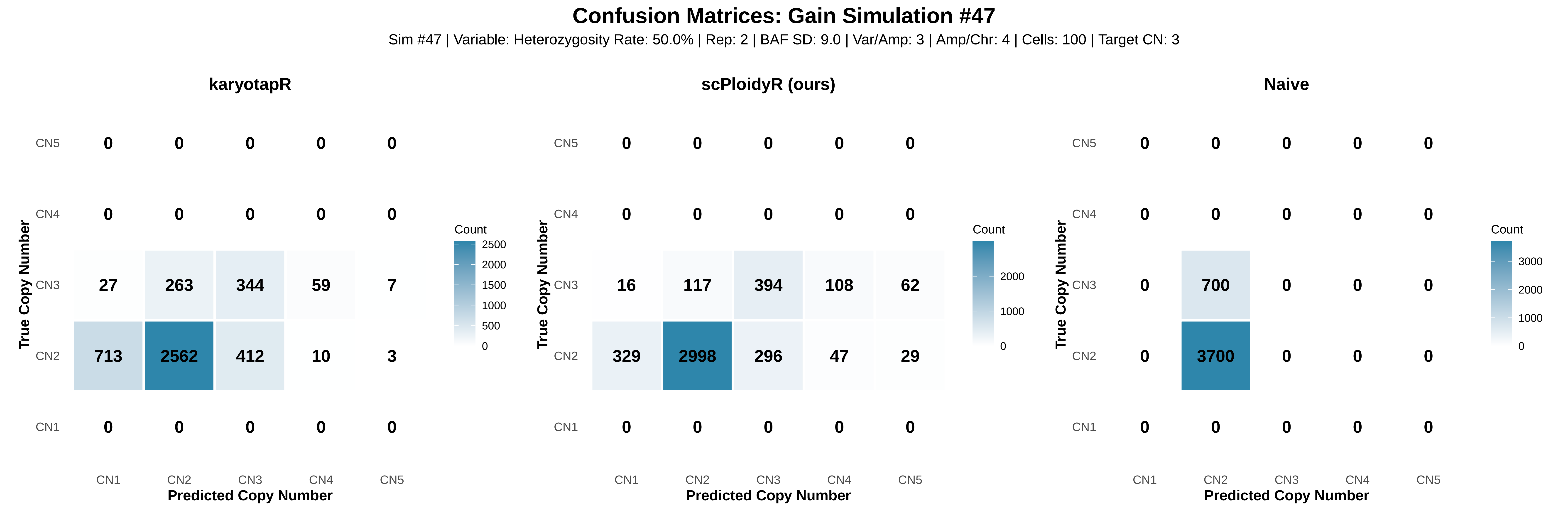
Confusion Matrices: Gain Simulation #47
Sim #47 | Variable: Heterozygosity Rate: 50.0% | Rep: 2 | BAF SD: 9.0 | Var/Amp: 3 | Amp/Chr: 4 | Cells: 100 | Target CN: 3
karyotapR
scPloidyR (ours)
Naive
True Copy Number
Predicted Copy Number
CN1
CN2
CN3
CN4
CN5
Count
karyotapR: CN3 row: 27 263 344 59 7; CN2 row: 713 2562 412 10 3
scPloidyR: CN3 row: 16 117 394 108 62; CN2 row: 329 2998 296 47 29
Naive: CN3 row: 0 700 0 0 0; CN2 row: 0 3700 0 0 0

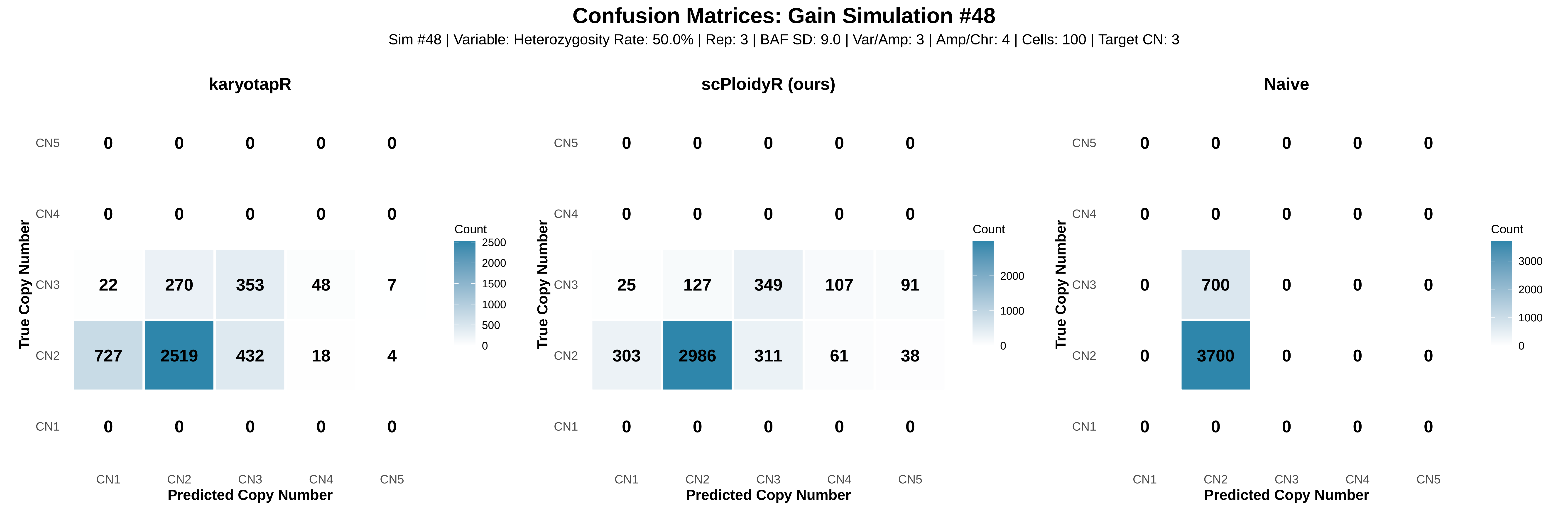

Confusion Matrices: Gain Simulation #48
Sim #48 | Variable: Heterozygosity Rate: 50.0% | Rep: 3 | BAF SD: 9.0 | Var/Amp: 3 | Amp/Chr: 4 | Cells: 100 | Target CN: 3
karyotapR
scPloidyR (ours)
Naive
True Copy Number
Predicted Copy Number
CN1
CN2
CN3
CN4
CN5
Count
0
0
0
0
0
0
0
0
0
0
22
270
353
48
7
727
2519
432
18
4
0
0
0
0
0
0
0
0
0
0
0
0
0
0
0
25
127
349
107
91
303
2986
311
61
38
0
0
0
0
0
0
0
0
0
0
0
0
0
0
0
0
700
0
0
0
0
3700
0
0
0
0
0
0
0
0

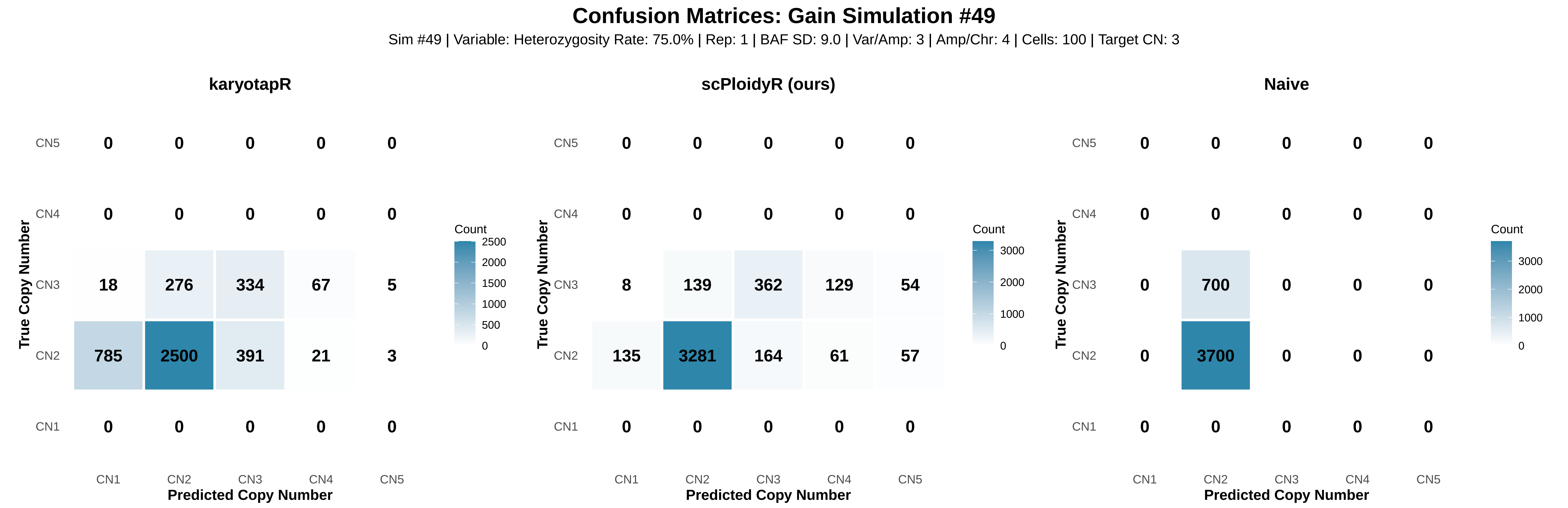

Confusion Matrices: Gain Simulation #49
Sim #49 | Variable: Heterozygosity Rate: 75.0% | Rep: 1 | BAF SD: 9.0 | Var/Amp: 3 | Amp/Chr: 4 | Cells: 100 | Target CN: 3
karyotapR
scPloidyR (ours)
Naive
True Copy Number
Predicted Copy Number
CN1
CN2
CN3
CN4
CN5
Count
0
500
1000
1500
2000
2500
3000
18
276
334
67
5
785
2500
391
21
3
8
139
362
129
54
135
3281
164
61
57
700
3700

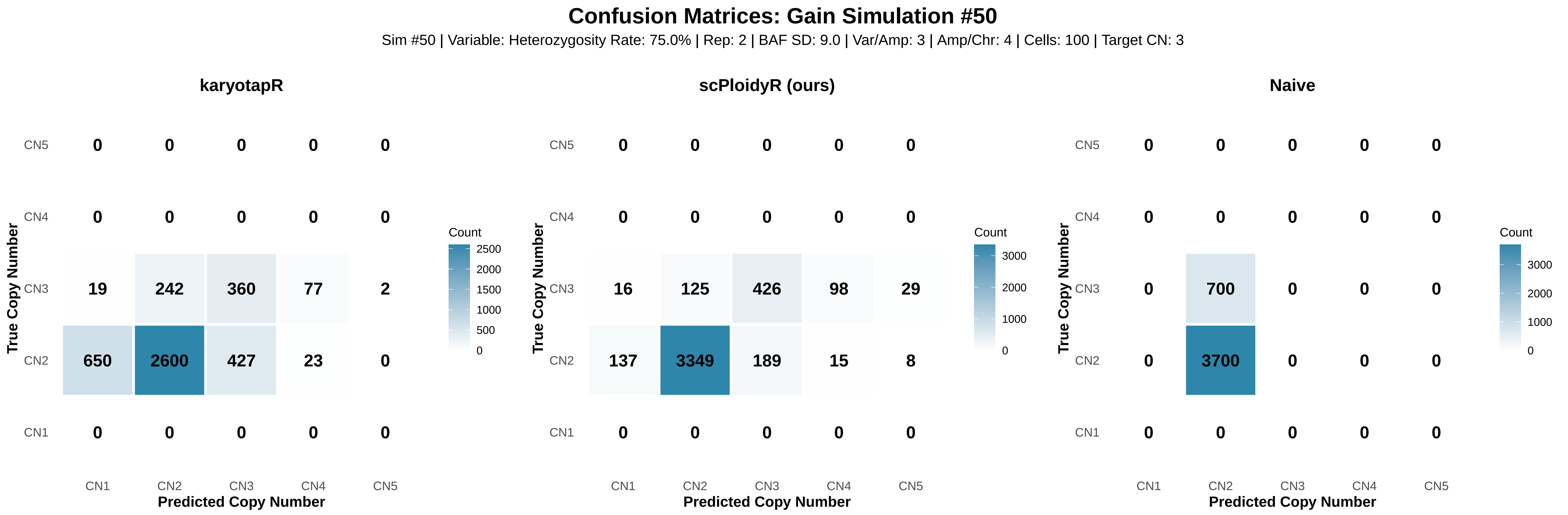
Confusion Matrices: Gain Simulation #50
Sim #50 | Variable: Heterozygosity Rate: 75.0% | Rep: 2 | BAF SD: 9.0 | Var/Amp: 3 | Amp/Chr: 4 | Cells: 100 | Target CN: 3
karyotapR
scPloidyR (ours)
Naive
True Copy Number
Predicted Copy Number
CN1
CN2
CN3
CN4
CN5
Count
karyotapR: CN3 row: 19, 242, 360, 77, 2; CN2 row: 650, 2600, 427, 23, 0
scPloidyR (ours): CN3 row: 16, 125, 426, 98, 29; CN2 row: 137, 3349, 189, 15, 8
Naive: CN3 row: 0, 700, 0, 0, 0; CN2 row: 0, 3700, 0, 0, 0

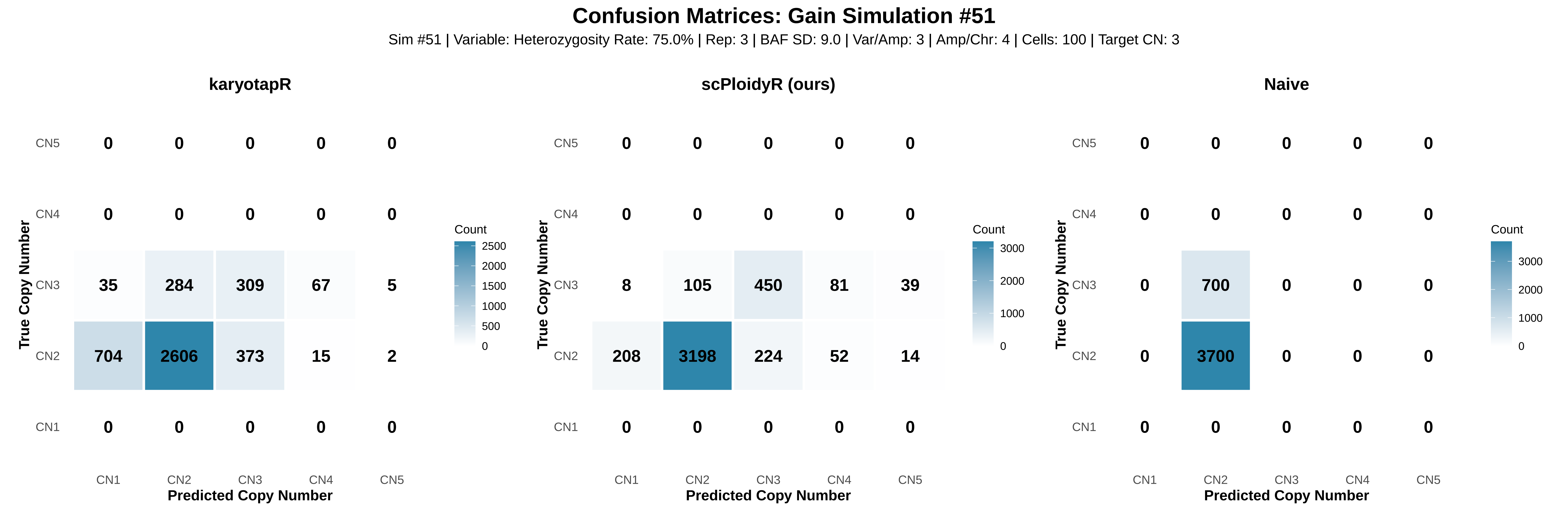
Confusion Matrices: Gain Simulation #51
Sim #51 | Variable: Heterozygosity Rate: 75.0% | Rep: 3 | BAF SD: 9.0 | Var/Amp: 3 | Amp/Chr: 4 | Cells: 100 | Target CN: 3
karyotapR
scPloidyR (ours)
Naive
True Copy Number
Predicted Copy Number
CN1
CN2
CN3
CN4
CN5
Count
karyotapR: CN5: 0 0 0 0 0; CN4: 0 0 0 0 0; CN3: 35 284 309 67 5; CN2: 704 2606 373 15 2; CN1: 0 0 0 0 0
scPloidyR (ours): CN5: 0 0 0 0 0; CN4: 0 0 0 0 0; CN3: 8 105 450 81 39; CN2: 208 3198 224 52 14; CN1: 0 0 0 0 0
Naive: CN5: 0 0 0 0 0; CN4: 0 0 0 0 0; CN3: 0 700 0 0 0; CN2: 0 3700 0 0 0; CN1: 0 0 0 0 0
2500
2000
1500
1000
500
0
3000

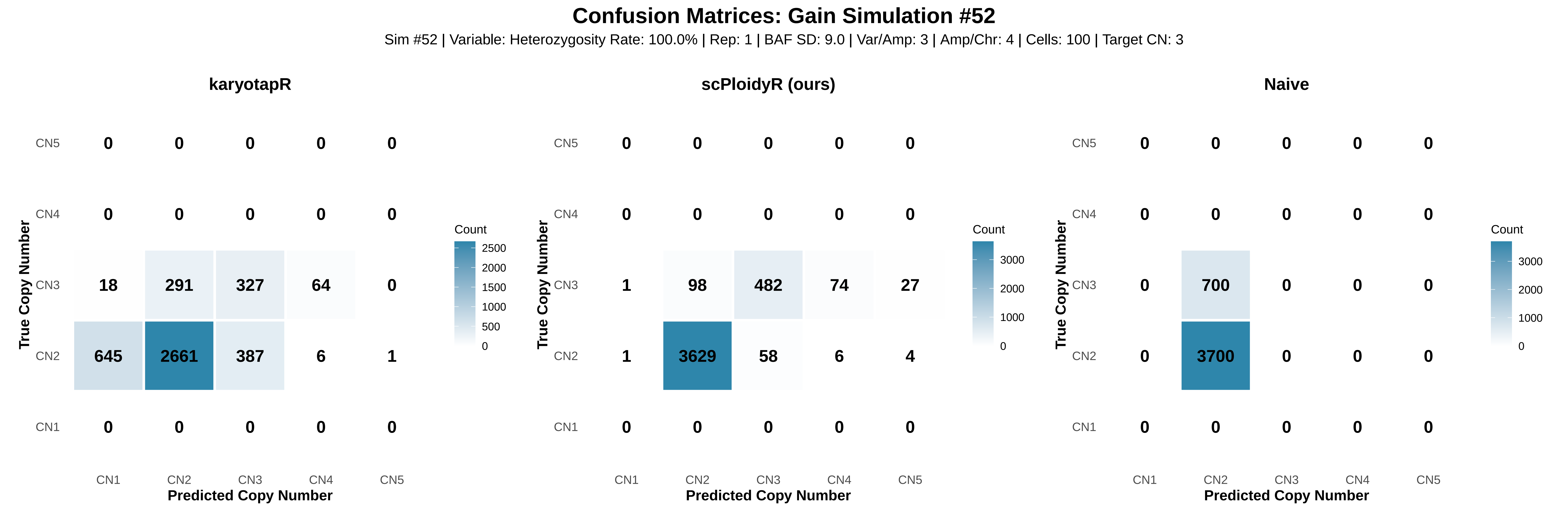

Confusion Matrices: Gain Simulation #52
Sim #52 | Variable: Heterozygosity Rate: 100.0% | Rep: 1 | BAF SD: 9.0 | Var/Amp: 3 | Amp/Chr: 4 | Cells: 100 | Target CN: 3
karyotapR
scPloidyR (ours)
Naive
True Copy Number
Predicted Copy Number
Count
CN1
CN2
CN3
CN4
CN5

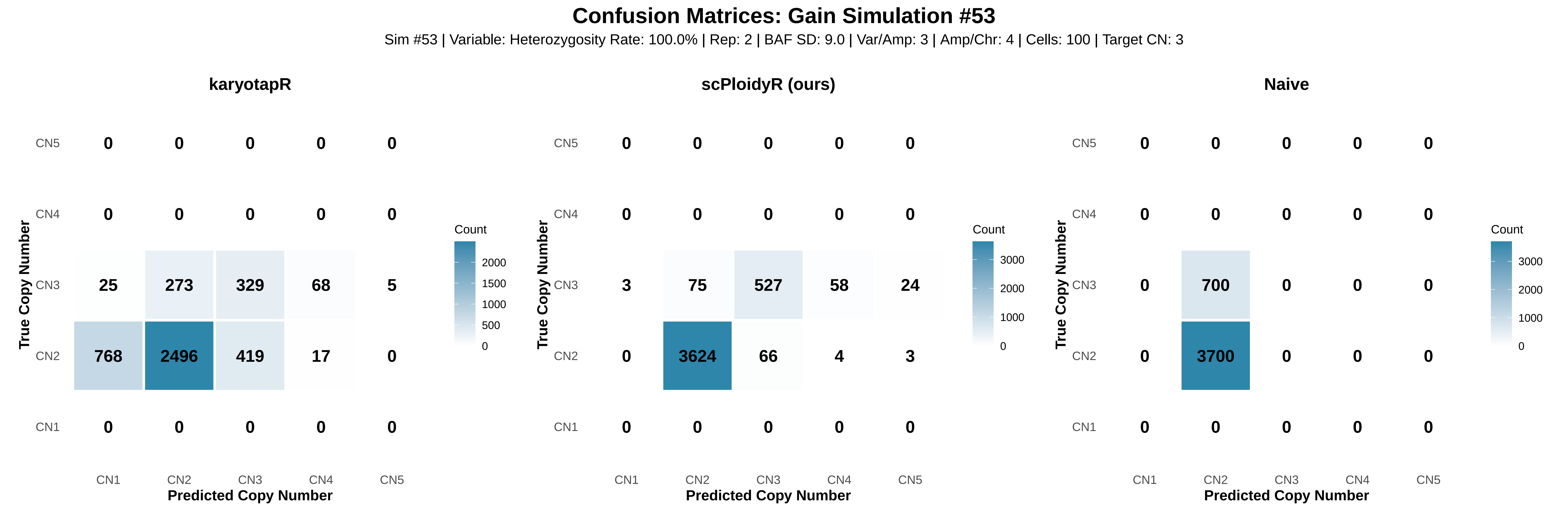
Confusion Matrices: Gain Simulation #53
Sim #53 | Variable: Heterozygosity Rate: 100.0% | Rep: 2 | BAF SD: 9.0 | Var/Amp: 3 | Amp/Chr: 4 | Cells: 100 | Target CN: 3
karyotapR
scPloidyR (ours)
Naive
True Copy Number
Predicted Copy Number
CN1
CN2
CN3
CN4
CN5
Count
karyotapR CN3: 25 273 329 68 5
karyotapR CN2: 768 2496 419 17 0
scPloidyR CN3: 3 75 527 58 24
scPloidyR CN2: 0 3624 66 4 3
Naive CN3: 0 700 0 0 0
Naive CN2: 0 3700 0 0 0

Confusion Matrices: Gain Simulation #54

Sim #54 | Variable: Heterozygosity Rate: 100.0% | Rep: 3 | BAF SD: 9.0 | Var/Amp: 3 | Amp/Chr: 4 | Cells: 100 | Target CN: 3

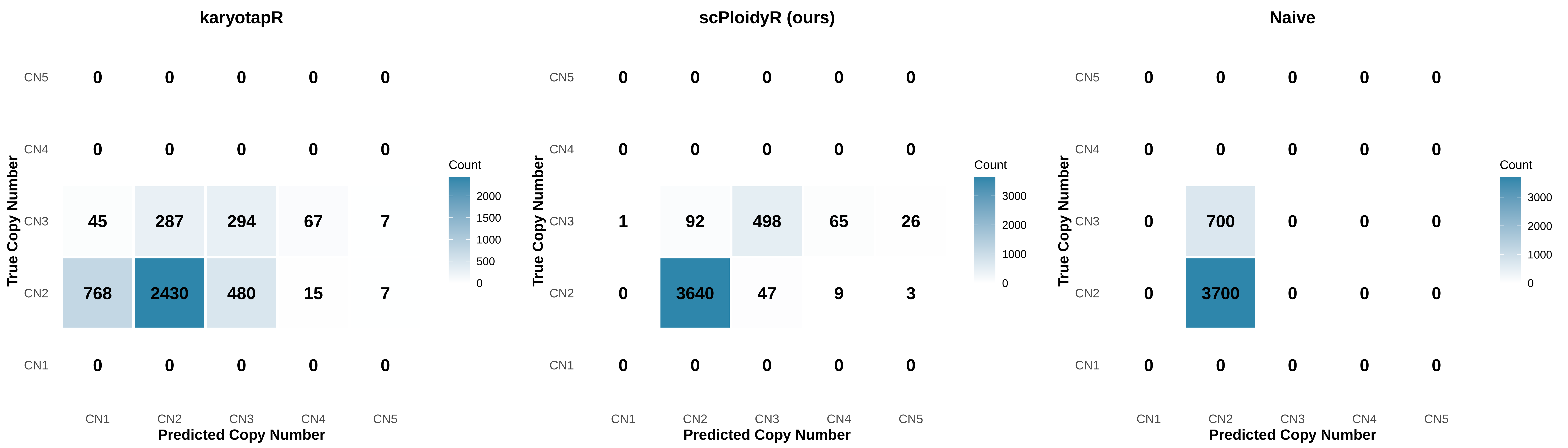

Supplement: Supplementary file 1 [file csbj.0203.f1.zip › revision_S3_confusion_gain.pdf]
