# Supplementary material for: Integrative Modeling of Read Depth and B-Allele Frequency Improves Single-Cell Copy Number Calling from Targeted DNA Sequencing Panels
Source: Comput Struct Biotechnol J. 2026 Aug 21;35(1):0203. doi: 10.34133/csbj.0203 (PMC13494159; doi:10.34133/csbj.0203)
Supplement: Supplementary file 1 [file csbj.0203.f1.zip › revision_S4_confusion_loss.pdf]

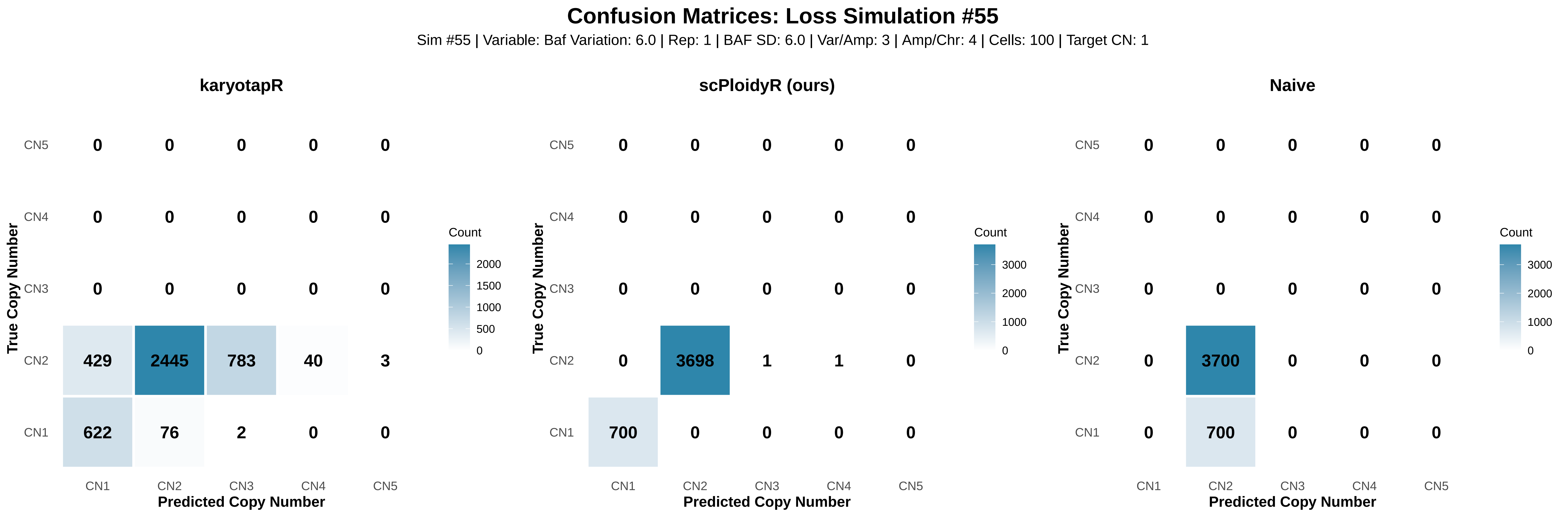
Confusion Matrices: Loss Simulation #55
Sim #55 | Variable: Baf Variation: 6.0 | Rep: 1 | BAF SD: 6.0 | Var/Amp: 3 | Amp/Chr: 4 | Cells: 100 | Target CN: 1
karyotapR
scPloidyR (ours)
Naive
True Copy Number
Predicted Copy Number
CN1
CN2
CN3
CN4
CN5
Count
2000
1500
1000
500
0
3000
2000
1000
0
429
2445
783
40
3
622
76
2
3698
1
700
3700

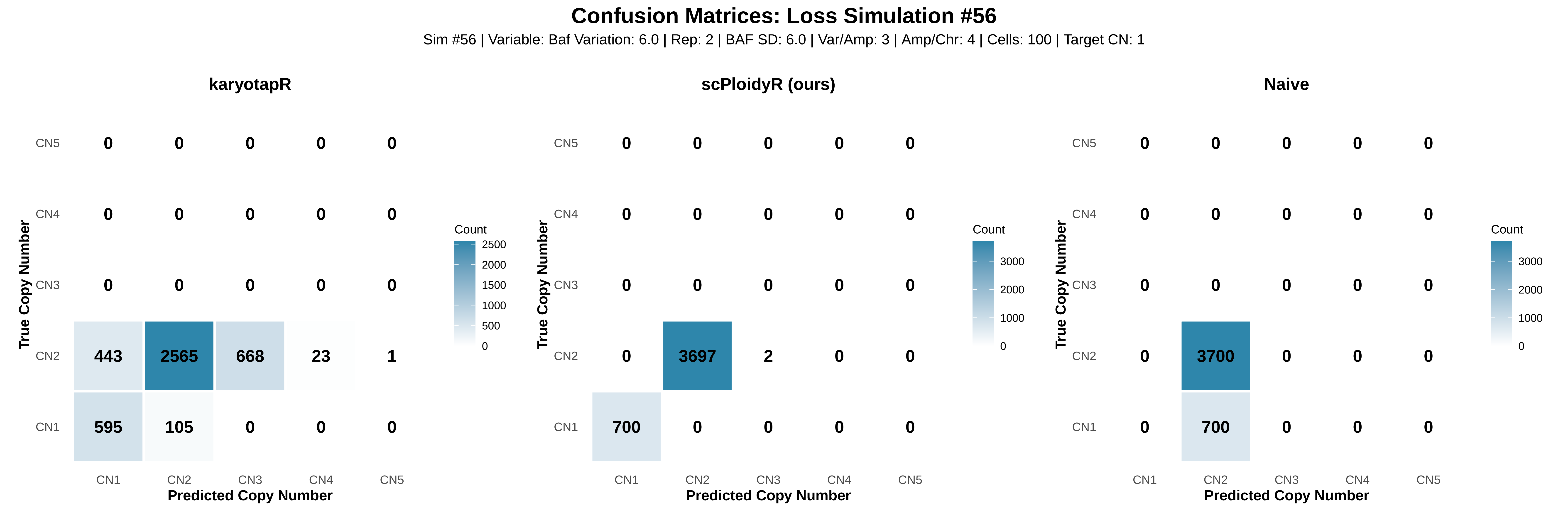
Confusion Matrices: Loss Simulation #56
Sim #56 | Variable: Baf Variation: 6.0 | Rep: 2 | BAF SD: 6.0 | Var/Amp: 3 | Amp/Chr: 4 | Cells: 100 | Target CN: 1
karyotapR
scPloidyR (ours)
Naive
True Copy Number
Predicted Copy Number
CN1
CN2
CN3
CN4
CN5
Count
443
2565
668
23
1
595
105
3697
2
700
3700
2500
2000
1500
1000
500
0
3000

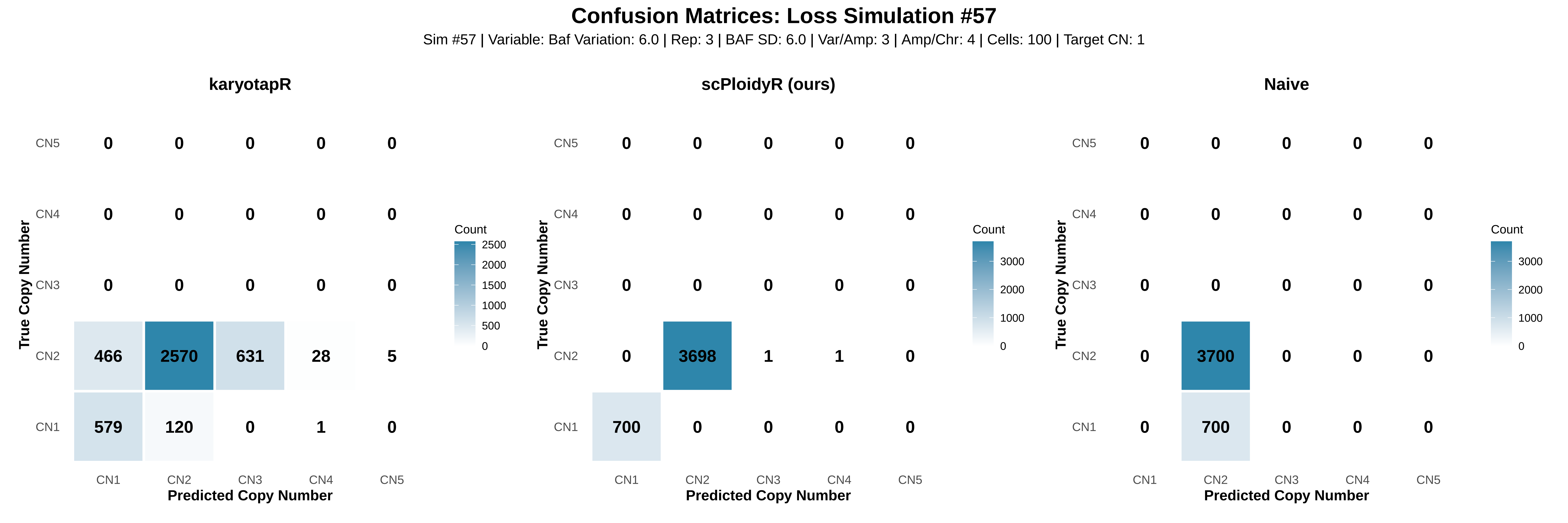
Confusion Matrices: Loss Simulation #57
Sim #57 | Variable: Baf Variation: 6.0 | Rep: 3 | BAF SD: 6.0 | Var/Amp: 3 | Amp/Chr: 4 | Cells: 100 | Target CN: 1
karyotapR
scPloidyR (ours)
Naive
True Copy Number
Predicted Copy Number
CN1
CN2
CN3
CN4
CN5
Count
2500
2000
1500
1000
500
0
3000
466
2570
631
28
5
579
120
1
3698
700
3700

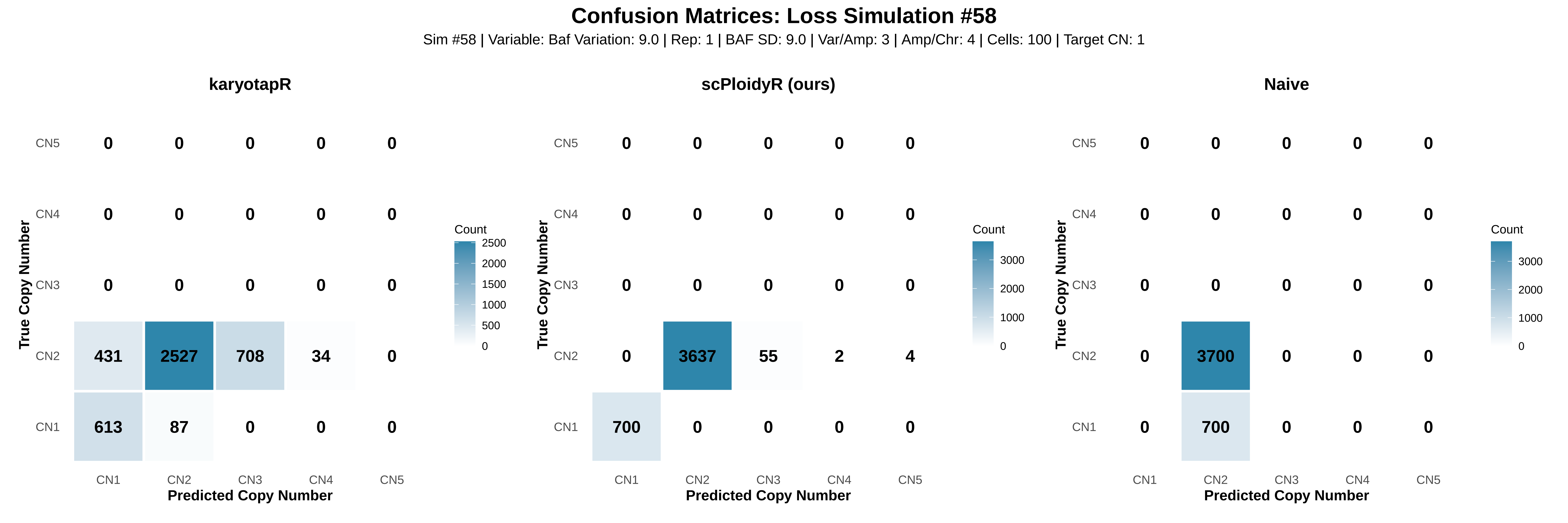
Confusion Matrices: Loss Simulation #58
Sim #58 | Variable: Baf Variation: 9.0 | Rep: 1 | BAF SD: 9.0 | Var/Amp: 3 | Amp/Chr: 4 | Cells: 100 | Target CN: 1
karyotapR
scPloidyR (ours)
Naive
True Copy Number
Predicted Copy Number
CN1
CN2
CN3
CN4
CN5
Count
431
2527
708
34
613
87
3637
55
2
4
700
3700
700
2500
2000
1500
1000
500
3000
0

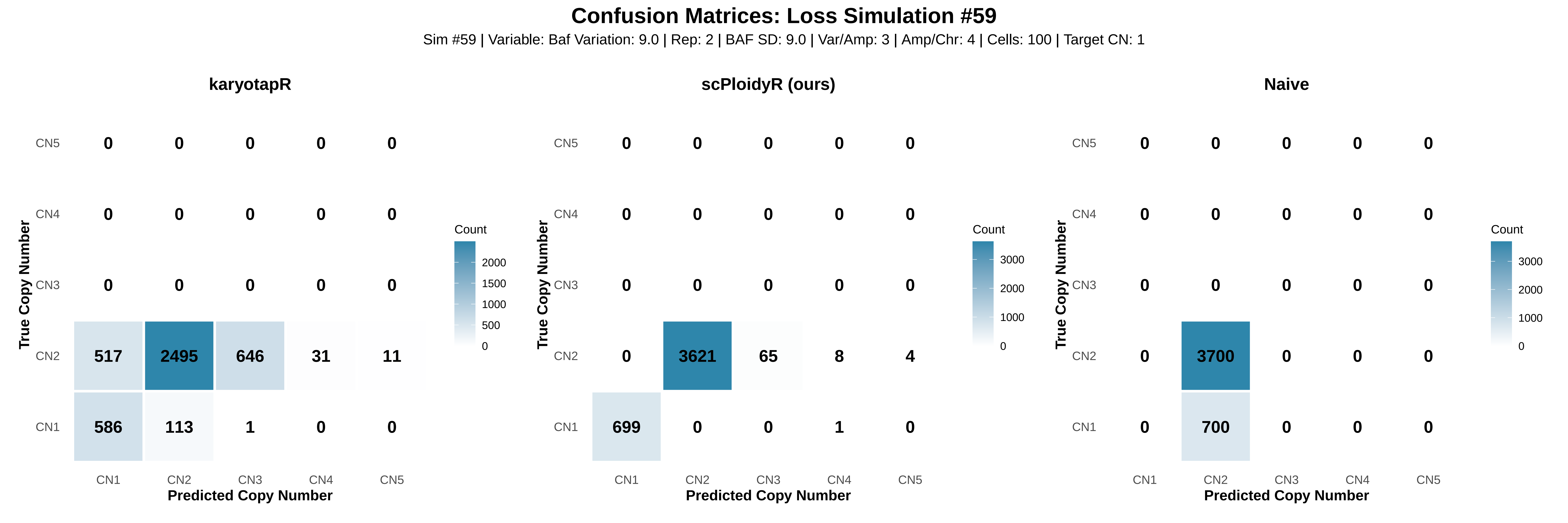

Confusion Matrices: Loss Simulation #59
Sim #59 | Variable: Baf Variation: 9.0 | Rep: 2 | BAF SD: 9.0 | Var/Amp: 3 | Amp/Chr: 4 | Cells: 100 | Target CN: 1
karyotapR
scPloidyR (ours)
Naive
True Copy Number
Predicted Copy Number
Count
CN1 CN2 CN3 CN4 CN5
karyotapR: CN5: 0 0 0 0 0; CN4: 0 0 0 0 0; CN3: 0 0 0 0 0; CN2: 517 2495 646 31 11; CN1: 586 113 1 0 0
scPloidyR (ours): CN5: 0 0 0 0 0; CN4: 0 0 0 0 0; CN3: 0 0 0 0 0; CN2: 0 3621 65 8 4; CN1: 699 0 0 1 0
Naive: CN5: 0 0 0 0 0; CN4: 0 0 0 0 0; CN3: 0 0 0 0 0; CN2: 0 3700 0 0 0; CN1: 0 700 0 0 0

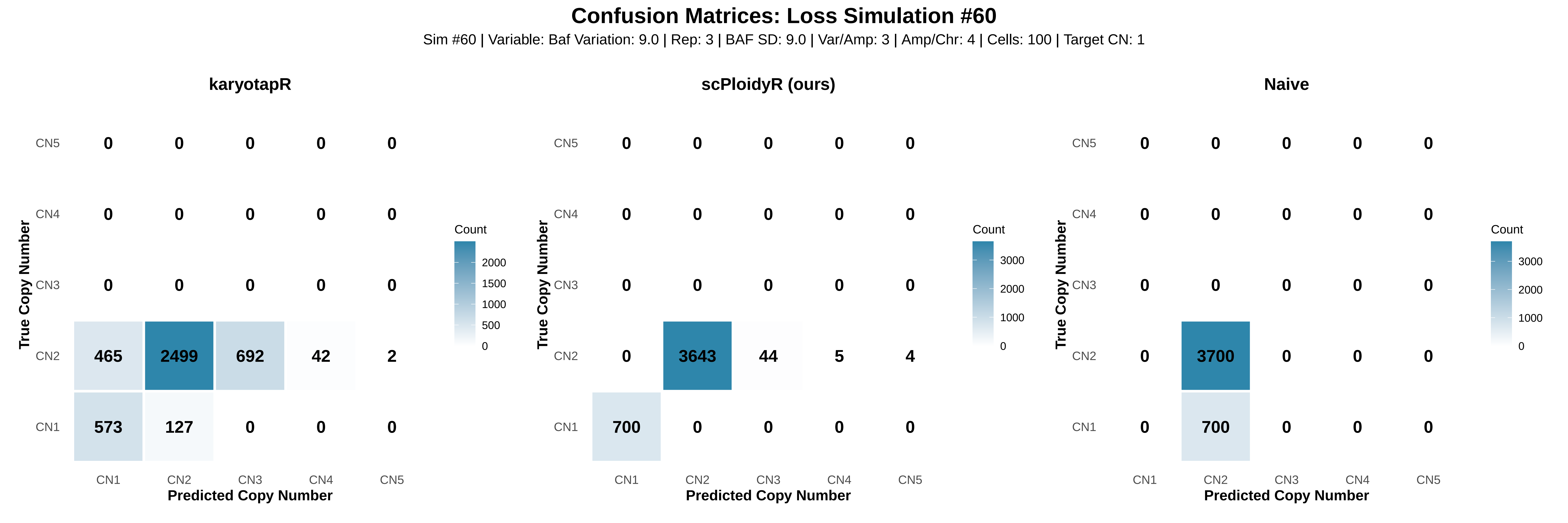

Confusion Matrices: Loss Simulation #60
Sim #60 | Variable: Baf Variation: 9.0 | Rep: 3 | BAF SD: 9.0 | Var/Amp: 3 | Amp/Chr: 4 | Cells: 100 | Target CN: 1
karyotapR
scPloidyR (ours)
Naive
True Copy Number
Predicted Copy Number
CN1
CN2
CN3
CN4
CN5
Count
465
2499
692
42
2
573
127
3643
44
5
4
700
3700

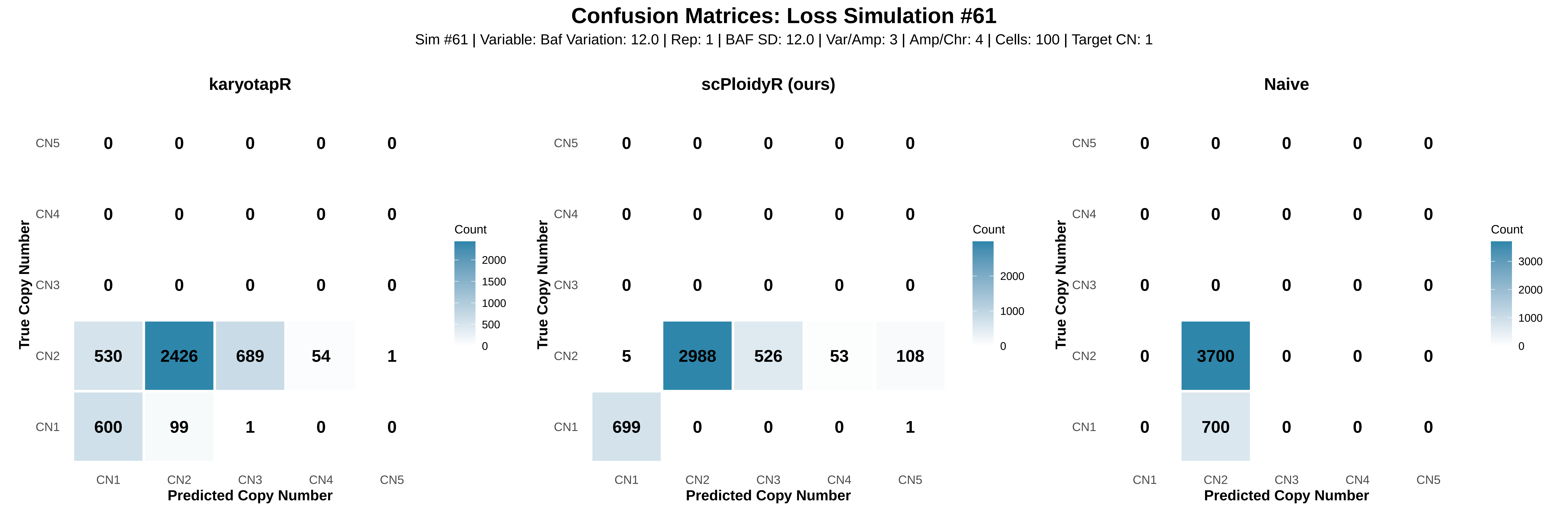
Confusion Matrices: Loss Simulation #61
Sim #61 | Variable: Baf Variation: 12.0 | Rep: 1 | BAF SD: 12.0 | Var/Amp: 3 | Amp/Chr: 4 | Cells: 100 | Target CN: 1
karyotapR
scPloidyR (ours)
Naive
True Copy Number
Predicted Copy Number
CN1
CN2
CN3
CN4
CN5
Count
karyotapR: CN5: 0 0 0 0 0
CN4: 0 0 0 0 0
CN3: 0 0 0 0 0
CN2: 530 2426 689 54 1
CN1: 600 99 1 0 0
0
500
1000
1500
2000
scPloidyR (ours): CN5: 0 0 0 0 0
CN4: 0 0 0 0 0
CN3: 0 0 0 0 0
CN2: 5 2988 526 53 108
CN1: 699 0 0 0 1
0
1000
2000
Naive: CN5: 0 0 0 0 0
CN4: 0 0 0 0 0
CN3: 0 0 0 0 0
CN2: 0 3700 0 0 0
CN1: 0 700 0 0 0
0
1000
2000
3000

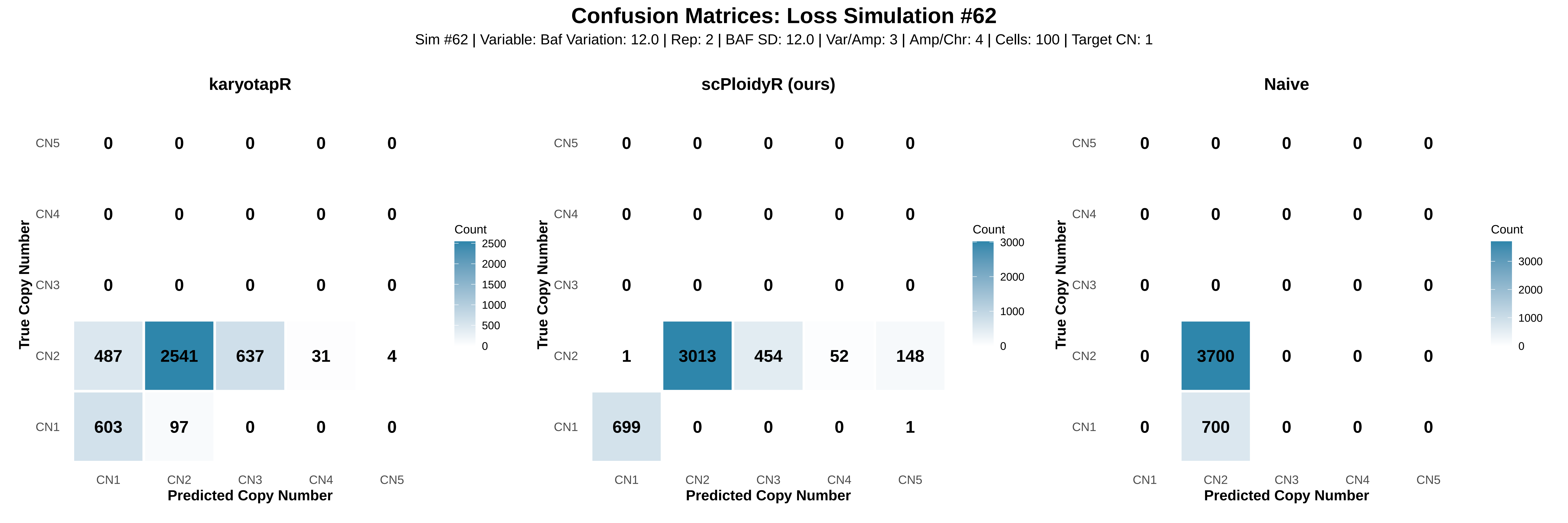
Confusion Matrices: Loss Simulation #62
Sim #62 | Variable: Baf Variation: 12.0 | Rep: 2 | BAF SD: 12.0 | Var/Amp: 3 | Amp/Chr: 4 | Cells: 100 | Target CN: 1
karyotapR
scPloidyR (ours)
Naive
True Copy Number
Predicted Copy Number
Count
CN1
CN2
CN3
CN4
CN5
487
2541
637
31
4
603
97
1
3013
454
52
148
699
3700
700

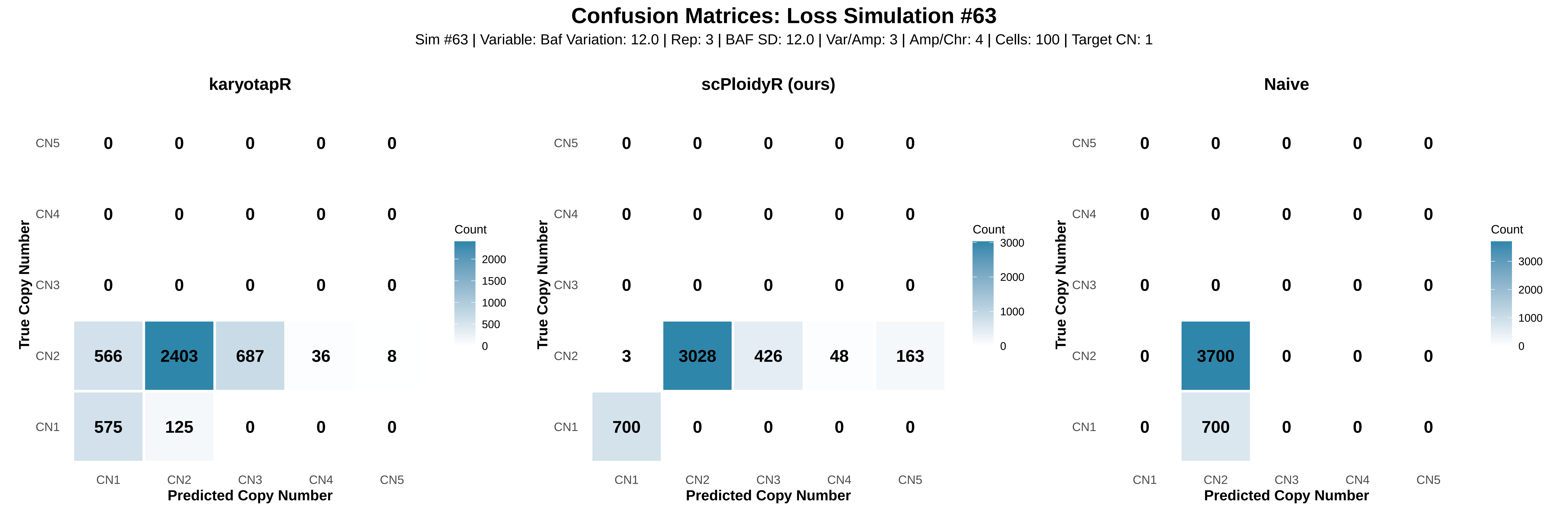
Confusion Matrices: Loss Simulation #63
Sim #63 | Variable: Baf Variation: 12.0 | Rep: 3 | BAF SD: 12.0 | Var/Amp: 3 | Amp/Chr: 4 | Cells: 100 | Target CN: 1
karyotapR
scPloidyR (ours)
Naive
True Copy Number
Predicted Copy Number
CN1
CN2
CN3
CN4
CN5
Count
566
2403
687
36
8
575
125
3
3028
426
48
163
700
3700
0
500
1000
1500
2000
3000

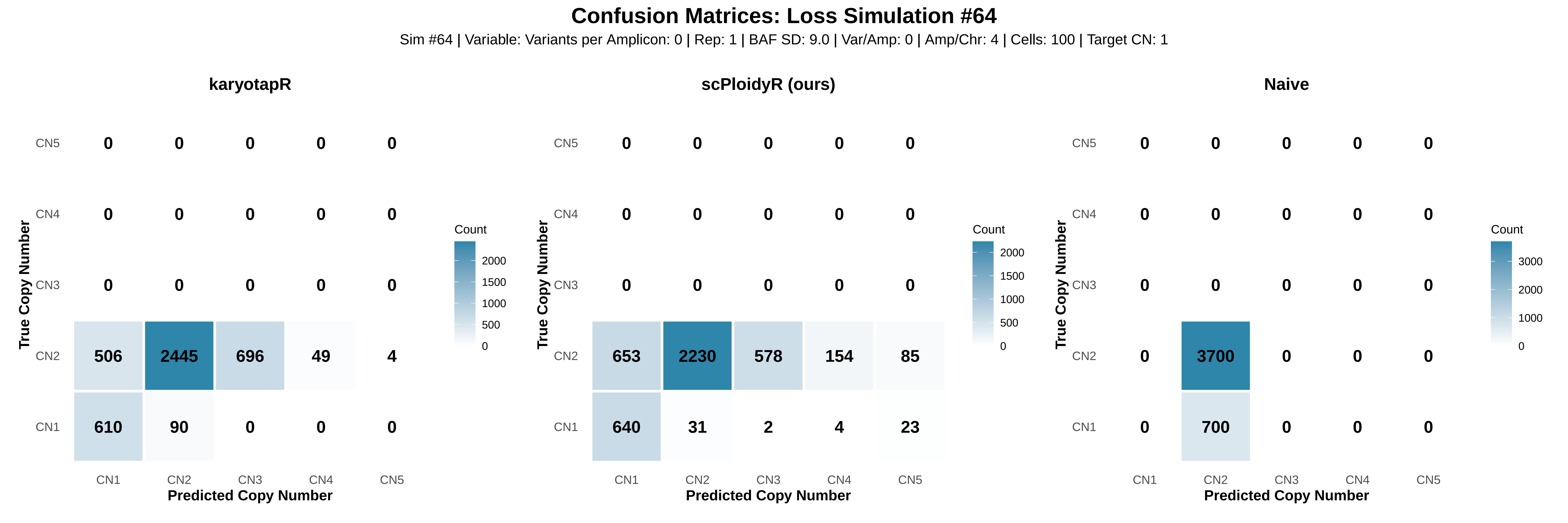

Confusion Matrices: Loss Simulation #64
Sim #64 | Variable: Variants per Amplicon: 0 | Rep: 1 | BAF SD: 9.0 | Var/Amp: 0 | Amp/Chr: 4 | Cells: 100 | Target CN: 1
karyotapR
scPloidyR (ours)
Naive
True Copy Number
Predicted Copy Number
Count
CN1
CN2
CN3
CN4
CN5
506
2445
696
49
4
610
90
653
2230
578
154
85
640
31
2
4
23
3700
700
0
500
1000
1500
2000
3000

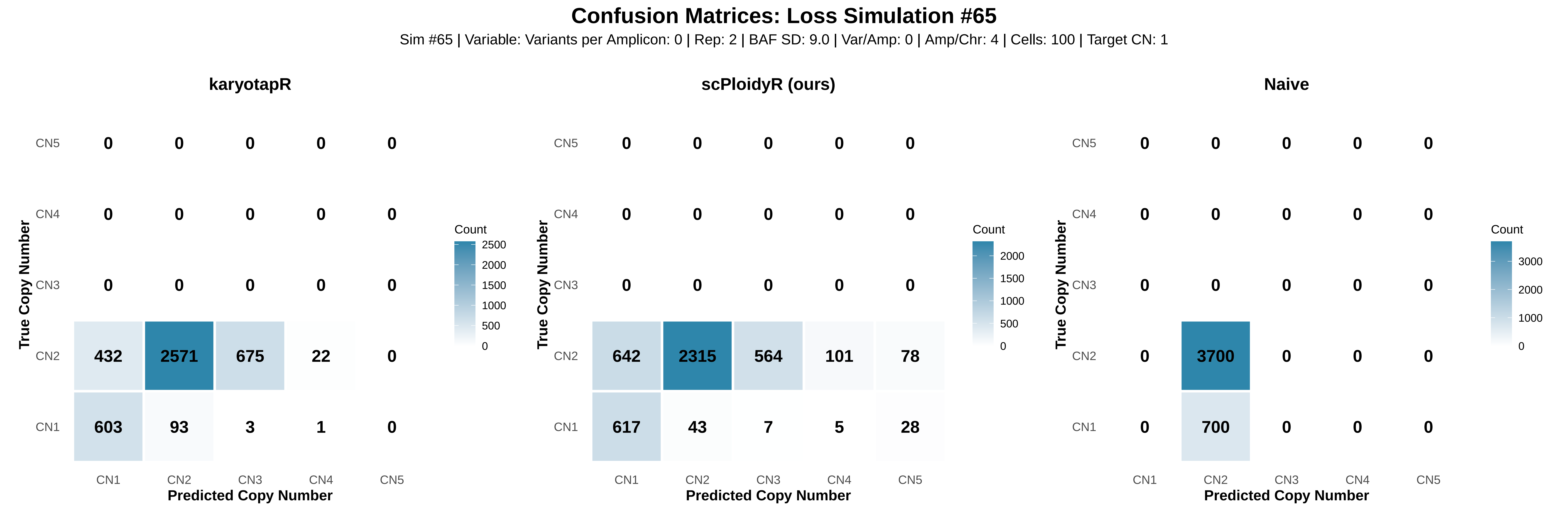

Confusion Matrices: Loss Simulation #65
Sim #65 | Variable: Variants per Amplicon: 0 | Rep: 2 | BAF SD: 9.0 | Var/Amp: 0 | Amp/Chr: 4 | Cells: 100 | Target CN: 1
karyotapR
scPloidyR (ours)
Naive
True Copy Number
Predicted Copy Number
Count
CN1
CN2
CN3
CN4
CN5
432
2571
675
22
603
93
3
1
642
2315
564
101
78
617
43
7
5
28
3700
700
2500
2000
1500
1000
500
3000
0

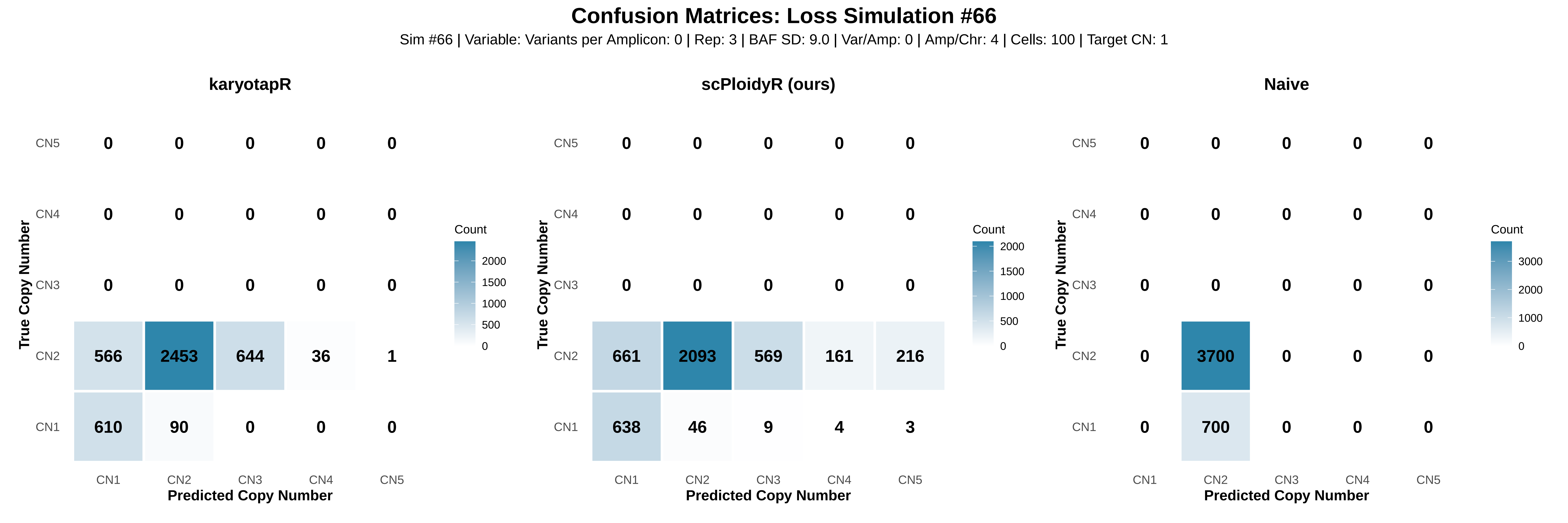

Confusion Matrices: Loss Simulation #66
Sim #66 | Variable: Variants per Amplicon: 0 | Rep: 3 | BAF SD: 9.0 | Var/Amp: 0 | Amp/Chr: 4 | Cells: 100 | Target CN: 1
karyotapR
scPloidyR (ours)
Naive
True Copy Number
Predicted Copy Number
CN1
CN2
CN3
CN4
CN5
Count
566
2453
644
36
1
610
90
661
2093
569
161
216
638
46
9
4
3
3700
700

Confusion Matrices: Loss Simulation #67

Sim #67 | Variable: Variants per Amplicon: 1 | Rep: 1 | BAF SD: 9.0 | Var/Amp: 1 | Amp/Chr: 4 | Cells: 100 | Target CN: 1

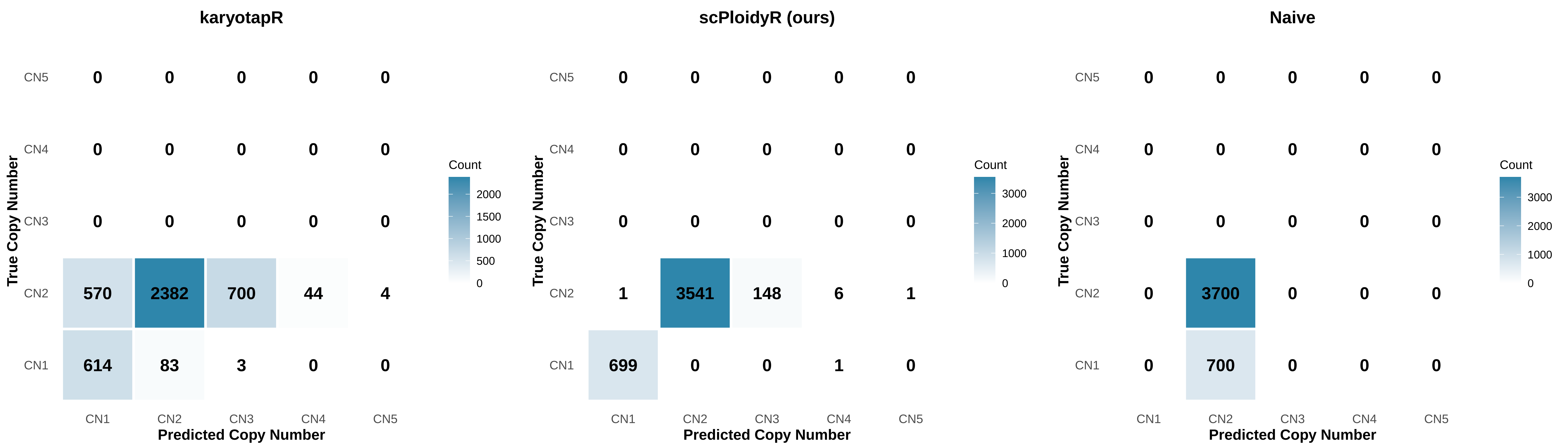

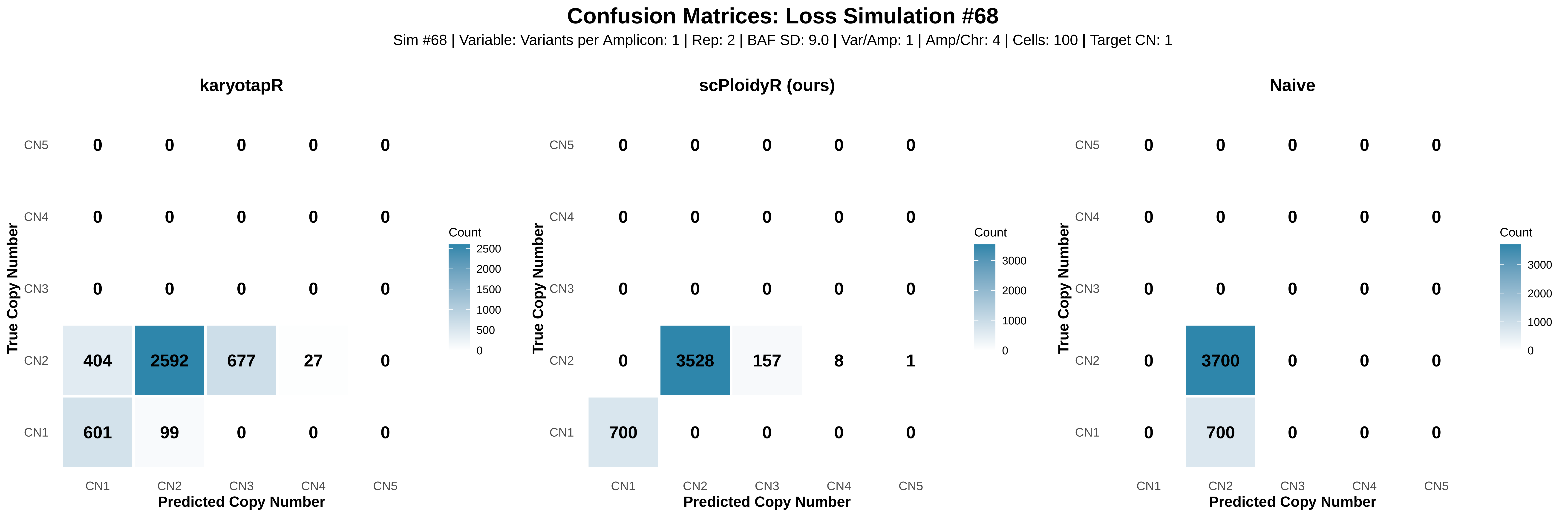
Confusion Matrices: Loss Simulation #68
Sim #68 | Variable: Variants per Amplicon: 1 | Rep: 2 | BAF SD: 9.0 | Var/Amp: 1 | Amp/Chr: 4 | Cells: 100 | Target CN: 1
karyotapR
CN5 0 0 0 0 0
CN4 0 0 0 0 0
CN3 0 0 0 0 0
CN2 404 2592 677 27 0
CN1 601 99 0 0 0
CN1 CN2 CN3 CN4 CN5
True Copy Number
Predicted Copy Number
Count
2500
2000
1500
1000
500
0
scPloidyR (ours)
CN5 0 0 0 0 0
CN4 0 0 0 0 0
CN3 0 0 0 0 0
CN2 0 3528 157 8 1
CN1 700 0 0 0 0
CN1 CN2 CN3 CN4 CN5
True Copy Number
Predicted Copy Number
Count
3000
2000
1000
0
Naive
CN5 0 0 0 0 0
CN4 0 0 0 0 0
CN3 0 0 0 0 0
CN2 0 3700 0 0 0
CN1 0 700 0 0 0
CN1 CN2 CN3 CN4 CN5
True Copy Number
Predicted Copy Number
Count
3000
2000
1000
0

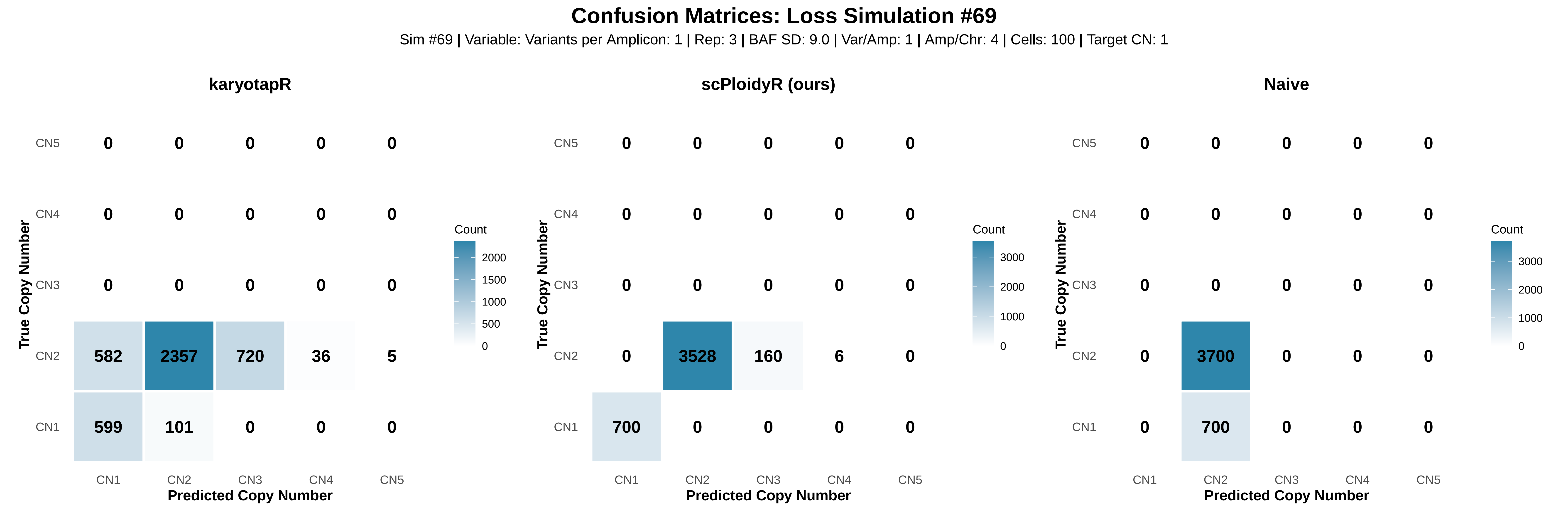

Confusion Matrices: Loss Simulation #69
Sim #69 | Variable: Variants per Amplicon: 1 | Rep: 3 | BAF SD: 9.0 | Var/Amp: 1 | Amp/Chr: 4 | Cells: 100 | Target CN: 1
karyotapR
scPloidyR (ours)
Naive
True Copy Number
Predicted Copy Number
CN1
CN2
CN3
CN4
CN5
Count
582
2357
720
36
5
599
101
3528
160
6
700
3700

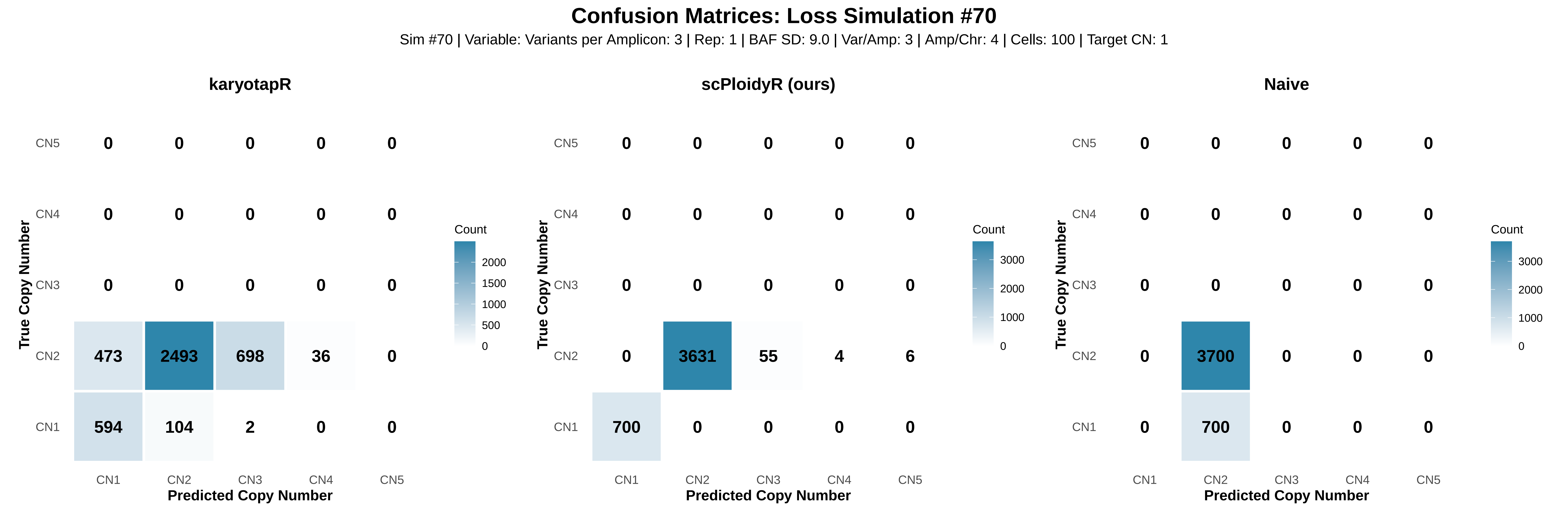

Confusion Matrices: Loss Simulation #70
Sim #70 | Variable: Variants per Amplicon: 3 | Rep: 1 | BAF SD: 9.0 | Var/Amp: 3 | Amp/Chr: 4 | Cells: 100 | Target CN: 1
karyotapR
scPloidyR (ours)
Naive
True Copy Number
Predicted Copy Number
Count
CN1
CN2
CN3
CN4
CN5
473
2493
698
36
594
104
2
3631
55
4
6
700
3700

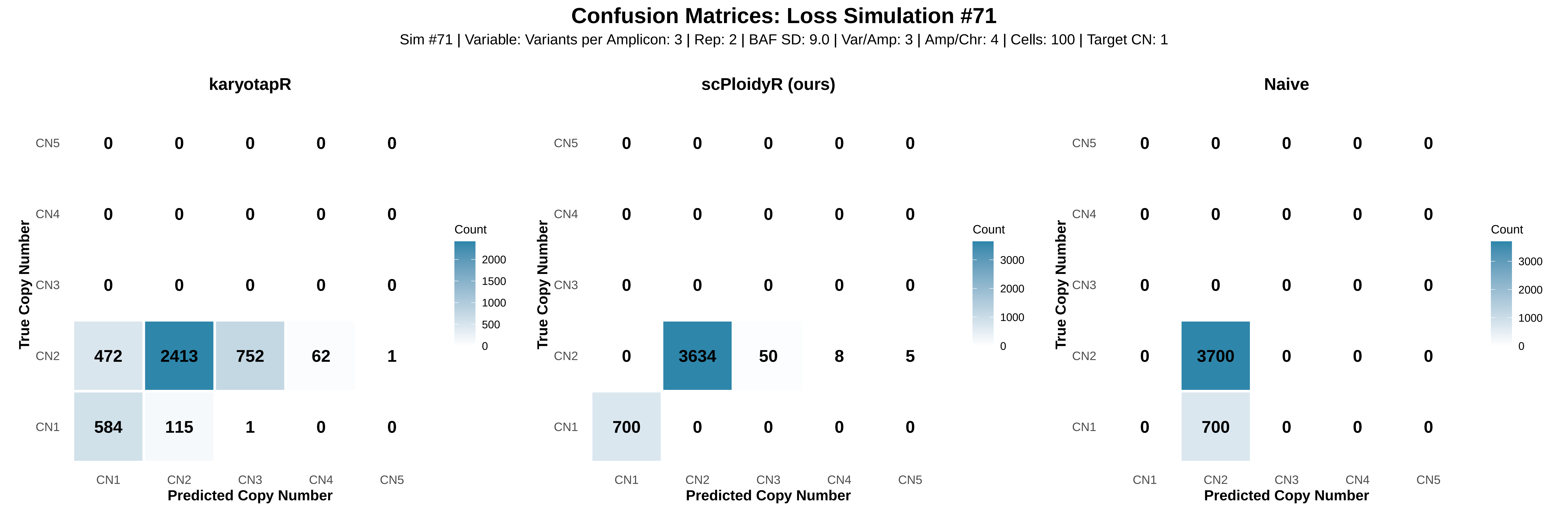
Confusion Matrices: Loss Simulation #71
Sim #71 | Variable: Variants per Amplicon: 3 | Rep: 2 | BAF SD: 9.0 | Var/Amp: 3 | Amp/Chr: 4 | Cells: 100 | Target CN: 1
karyotapR
scPloidyR (ours)
Naive
True Copy Number
Predicted Copy Number
CN1
CN2
CN3
CN4
CN5
Count
472
2413
752
62
1
584
115
3634
50
8
5
700
3700

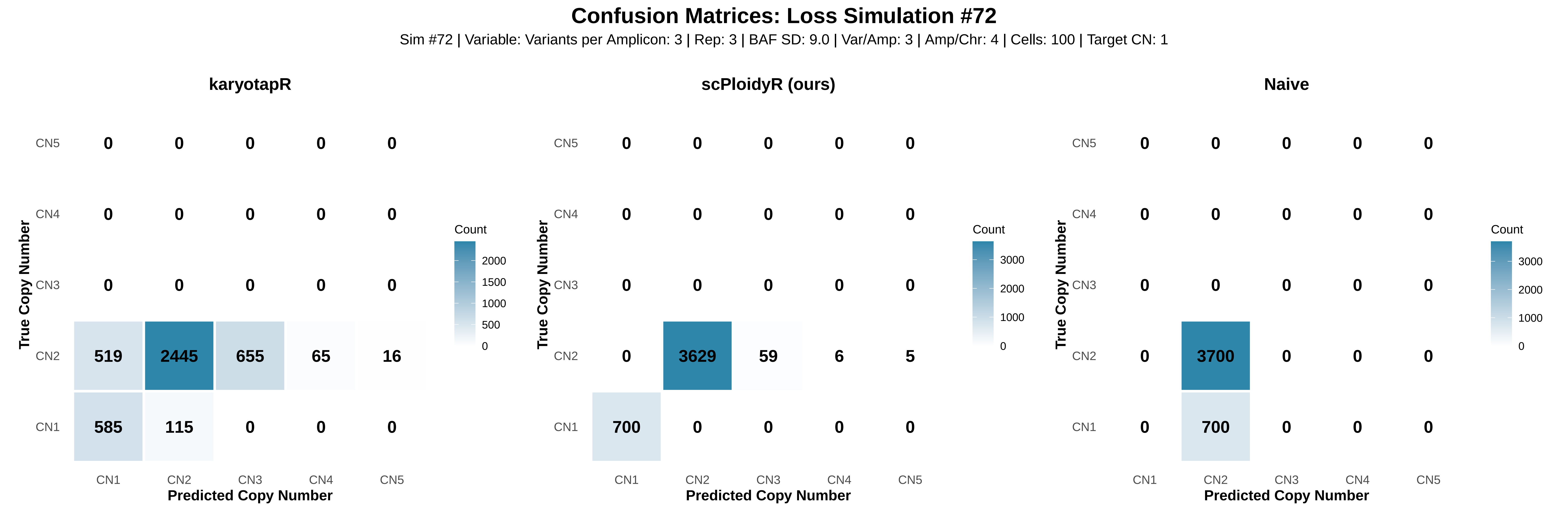

Confusion Matrices: Loss Simulation #72
Sim #72 | Variable: Variants per Amplicon: 3 | Rep: 3 | BAF SD: 9.0 | Var/Amp: 3 | Amp/Chr: 4 | Cells: 100 | Target CN: 1
karyotapR
scPloidyR (ours)
Naive
True Copy Number
Predicted Copy Number
CN1
CN2
CN3
CN4
CN5
Count
519
2445
655
65
16
585
115
3629
59
6
5
700
3700

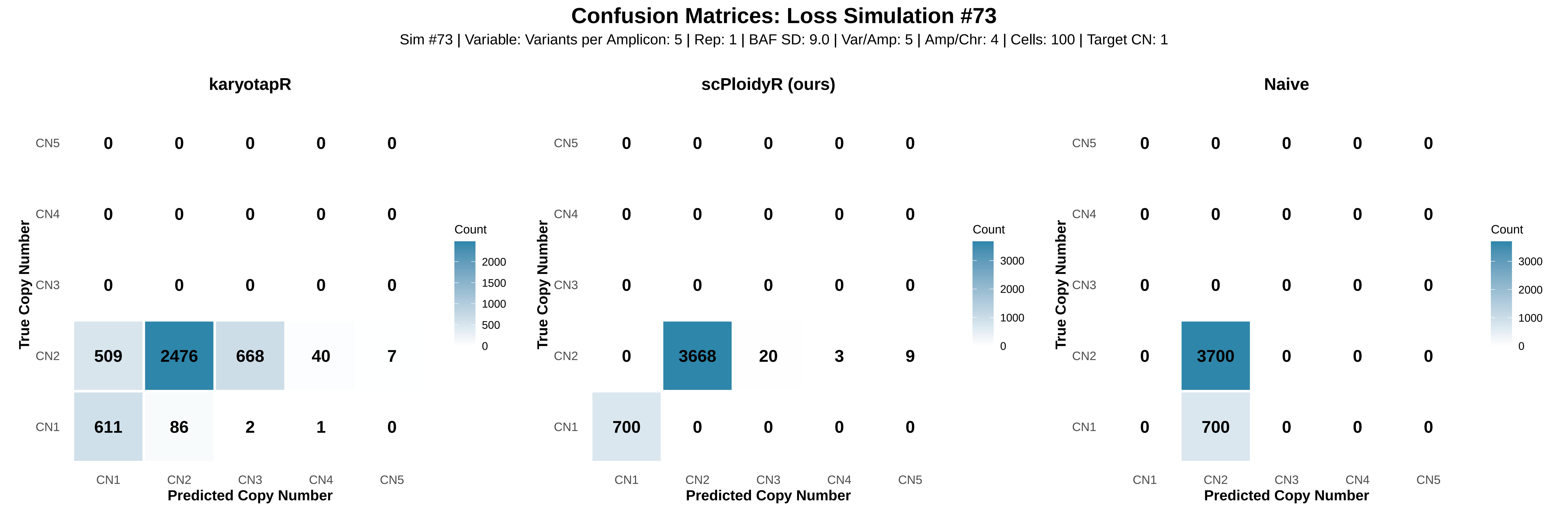
Confusion Matrices: Loss Simulation #73
Sim #73 | Variable: Variants per Amplicon: 5 | Rep: 1 | BAF SD: 9.0 | Var/Amp: 5 | Amp/Chr: 4 | Cells: 100 | Target CN: 1
karyotapR
scPloidyR (ours)
Naive
True Copy Number
Predicted Copy Number
CN1
CN2
CN3
CN4
CN5
Count
509
2476
668
40
7
611
86
2
1
3668
20
3
9
700
3700

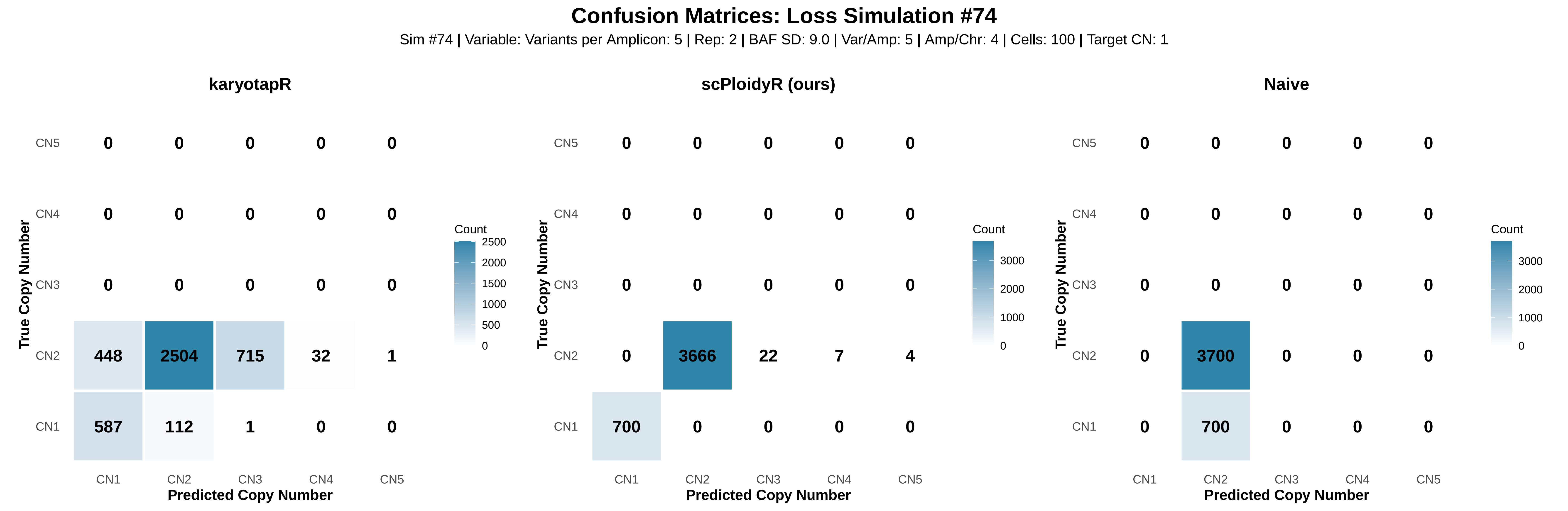

Confusion Matrices: Loss Simulation #74
Sim #74 | Variable: Variants per Amplicon: 5 | Rep: 2 | BAF SD: 9.0 | Var/Amp: 5 | Amp/Chr: 4 | Cells: 100 | Target CN: 1
karyotapR
scPloidyR (ours)
Naive
True Copy Number
Predicted Copy Number
Count
CN1
CN2
CN3
CN4
CN5
448
2504
715
32
1
587
112
1
3666
22
7
4
700
3700
700

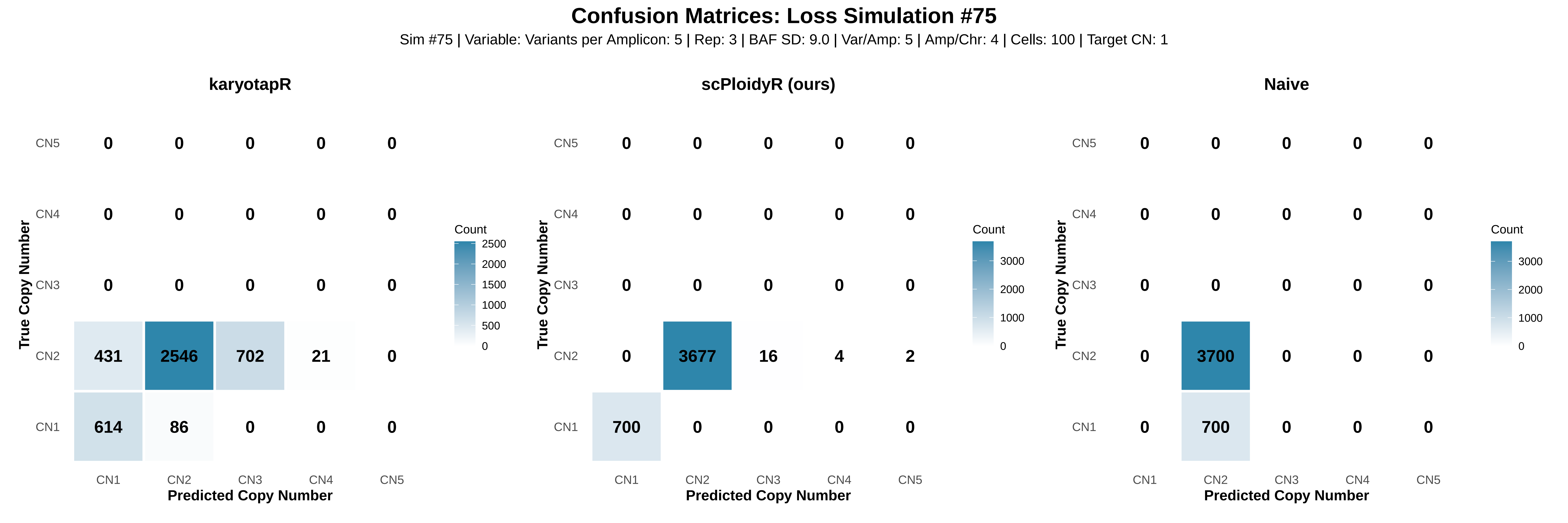
Confusion Matrices: Loss Simulation #75
Sim #75 | Variable: Variants per Amplicon: 5 | Rep: 3 | BAF SD: 9.0 | Var/Amp: 5 | Amp/Chr: 4 | Cells: 100 | Target CN: 1
karyotapR
scPloidyR (ours)
Naive
True Copy Number
Predicted Copy Number
CN1
CN2
CN3
CN4
CN5
Count
431
2546
702
21
614
86
3677
16
4
2
700
3700
0
500
1000
1500
2000
2500
3000

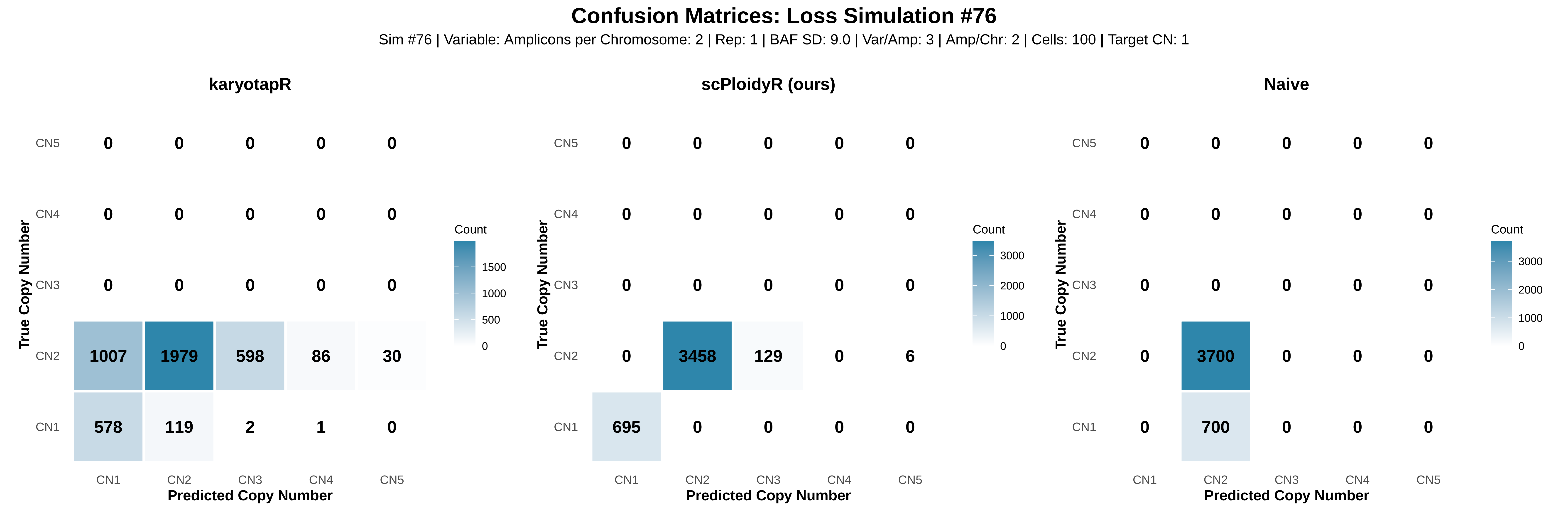
Confusion Matrices: Loss Simulation #76
Sim #76 | Variable: Amplicons per Chromosome: 2 | Rep: 1 | BAF SD: 9.0 | Var/Amp: 3 | Amp/Chr: 2 | Cells: 100 | Target CN: 1
karyotapR
scPloidyR (ours)
Naive
True Copy Number
Predicted Copy Number
Count
CN1
CN2
CN3
CN4
CN5
karyotapR: CN5: 0 0 0 0 0; CN4: 0 0 0 0 0; CN3: 0 0 0 0 0; CN2: 1007 1979 598 86 30; CN1: 578 119 2 1 0
scPloidyR (ours): CN5: 0 0 0 0 0; CN4: 0 0 0 0 0; CN3: 0 0 0 0 0; CN2: 0 3458 129 0 6; CN1: 695 0 0 0 0
Naive: CN5: 0 0 0 0 0; CN4: 0 0 0 0 0; CN3: 0 0 0 0 0; CN2: 0 3700 0 0 0; CN1: 0 700 0 0 0
0
500
1000
1500
2000
3000

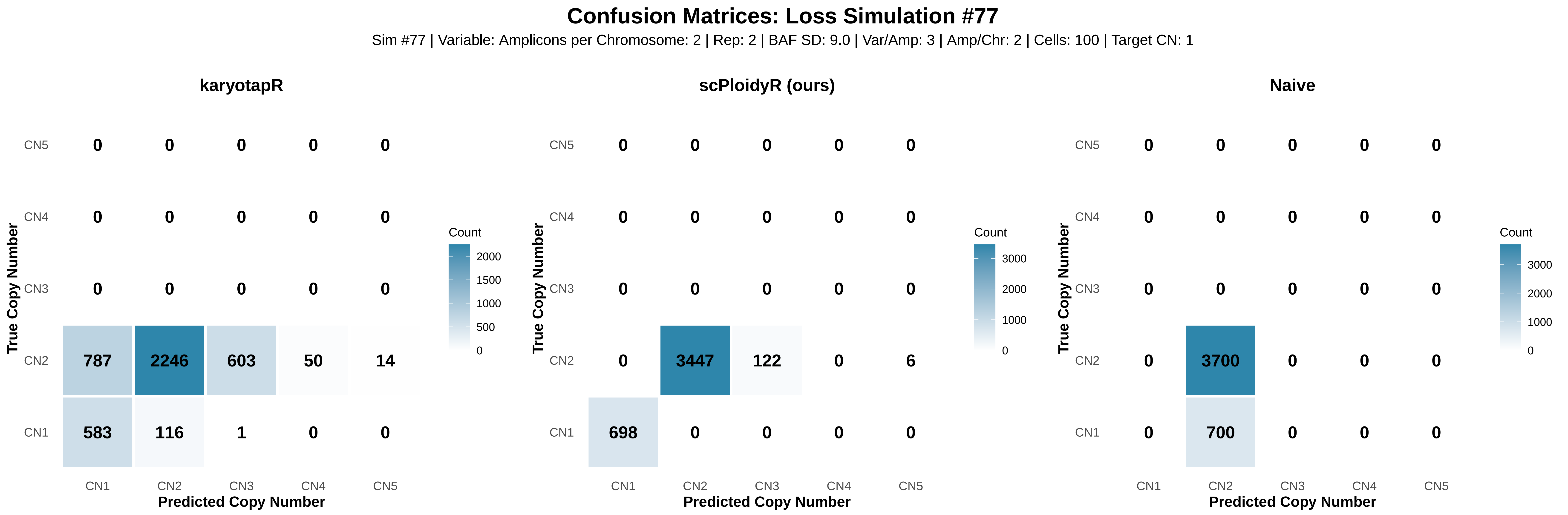

Confusion Matrices: Loss Simulation #77
Sim #77 | Variable: Amplicons per Chromosome: 2 | Rep: 2 | BAF SD: 9.0 | Var/Amp: 3 | Amp/Chr: 2 | Cells: 100 | Target CN: 1
karyotapR
scPloidyR (ours)
Naive
True Copy Number
Predicted Copy Number
Count
CN1
CN2
CN3
CN4
CN5
787
2246
603
50
14
583
116
1
3447
122
6
698
3700
700

Confusion Matrices: Loss Simulation #78

Sim #78 | Variable: Amplicons per Chromosome: 2 | Rep: 3 | BAF SD: 9.0 | Var/Amp: 3 | Amp/Chr: 2 | Cells: 100 | Target CN: 1

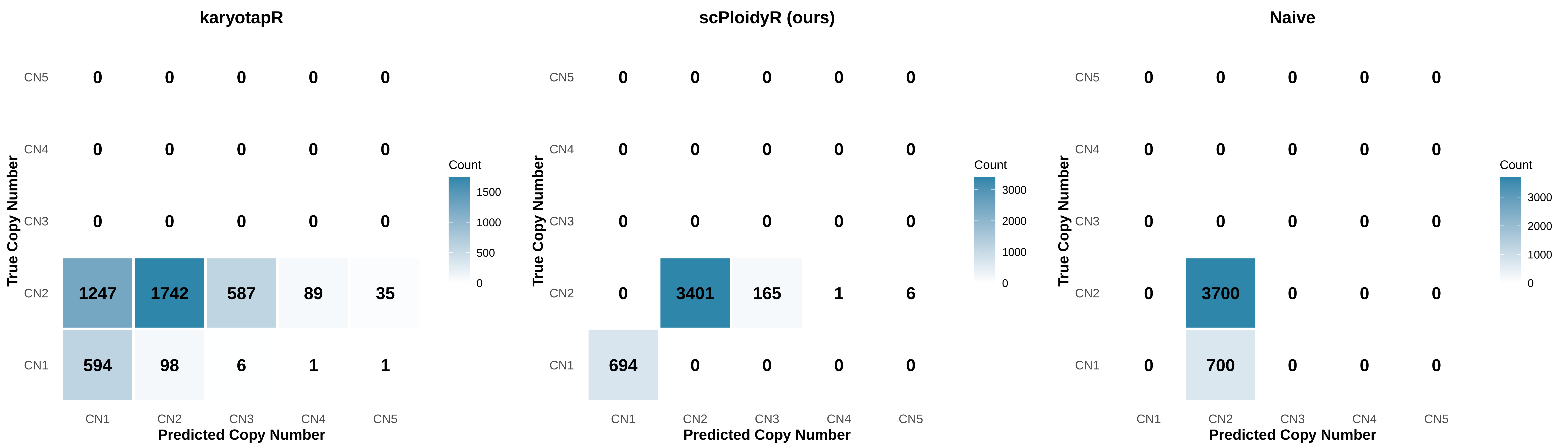

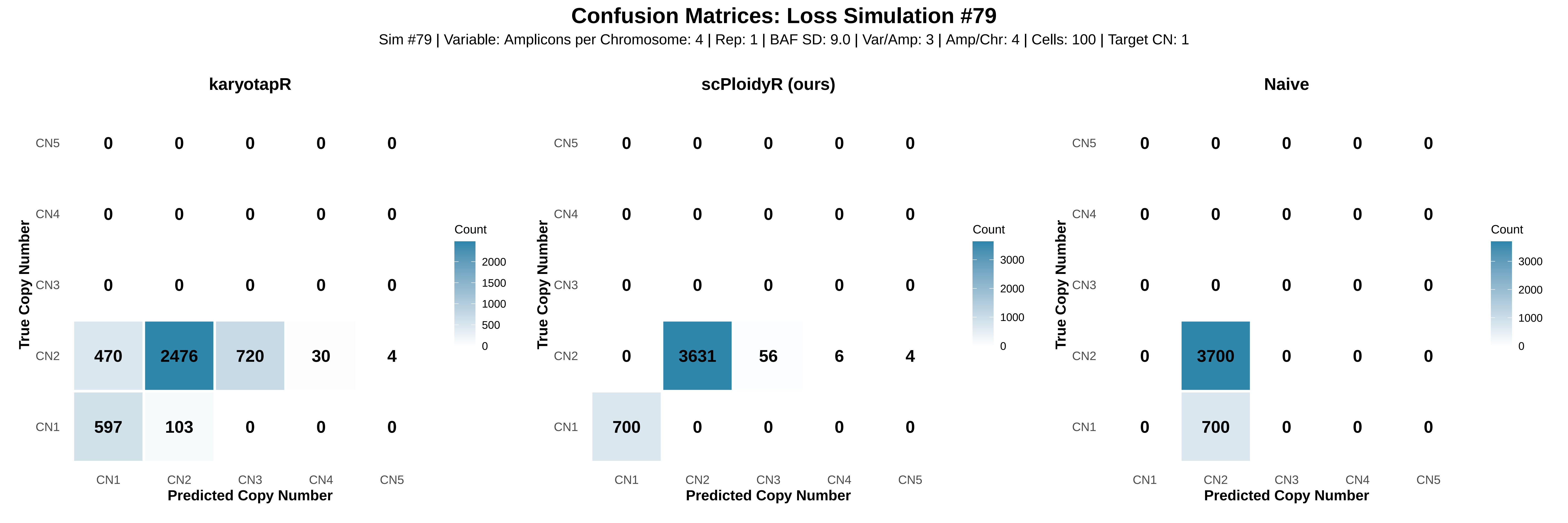
Confusion Matrices: Loss Simulation #79
Sim #79 | Variable: Amplicons per Chromosome: 4 | Rep: 1 | BAF SD: 9.0 | Var/Amp: 3 | Amp/Chr: 4 | Cells: 100 | Target CN: 1
karyotapR
scPloidyR (ours)
Naive
True Copy Number
Predicted Copy Number
Count
CN1
CN2
CN3
CN4
CN5
470
2476
720
30
4
597
103
3631
56
6
700
3700
0
500
1000
1500
2000
3000

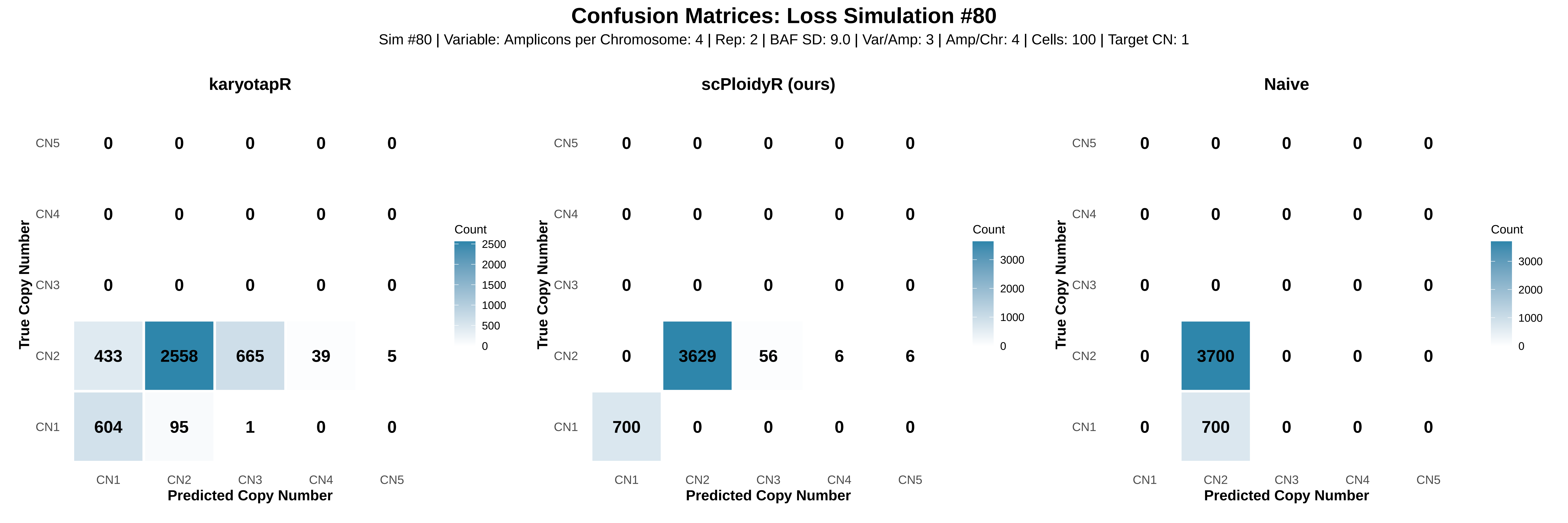
Confusion Matrices: Loss Simulation #80
Sim #80 | Variable: Amplicons per Chromosome: 4 | Rep: 2 | BAF SD: 9.0 | Var/Amp: 3 | Amp/Chr: 4 | Cells: 100 | Target CN: 1
karyotapR
scPloidyR (ours)
Naive
True Copy Number
Predicted Copy Number
Count
CN1
CN2
CN3
CN4
CN5
433
2558
665
39
5
604
95
1
3629
56
6
700
3700
2500
2000
1500
1000
500
3000
0

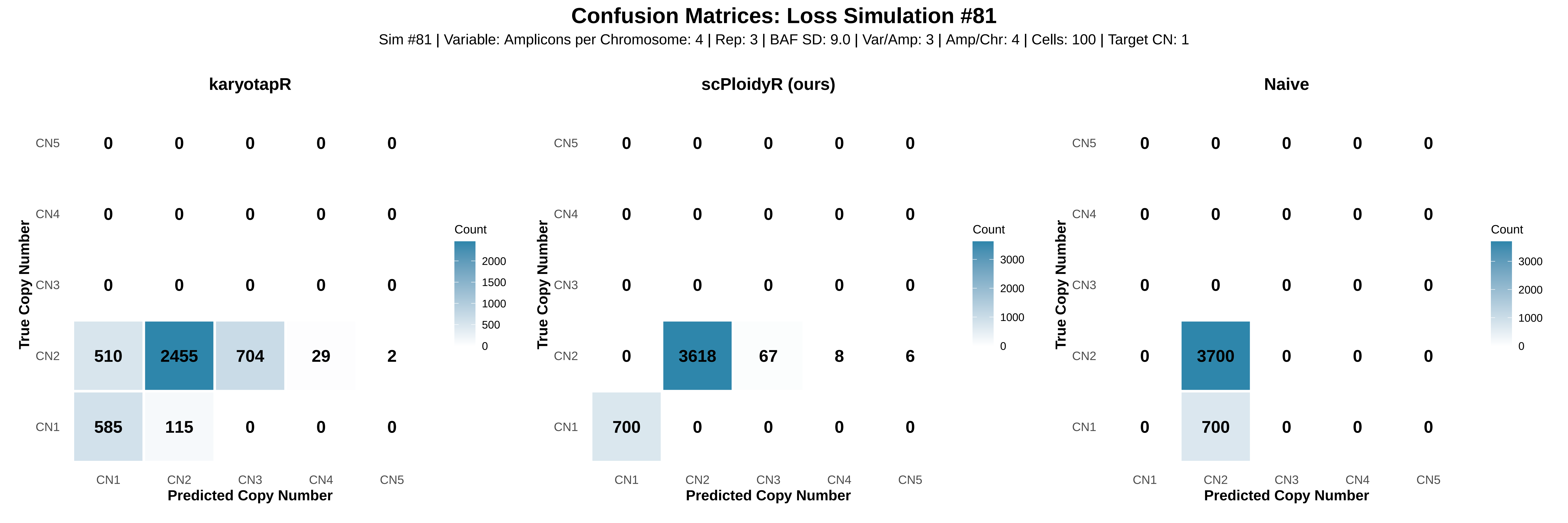
Confusion Matrices: Loss Simulation #81
Sim #81 | Variable: Amplicons per Chromosome: 4 | Rep: 3 | BAF SD: 9.0 | Var/Amp: 3 | Amp/Chr: 4 | Cells: 100 | Target CN: 1
karyotapR
scPloidyR (ours)
Naive
True Copy Number
Predicted Copy Number
CN1
CN2
CN3
CN4
CN5
Count
0
500
1000
1500
2000
3000
510
2455
704
29
2
585
115
3618
67
8
6
700
3700

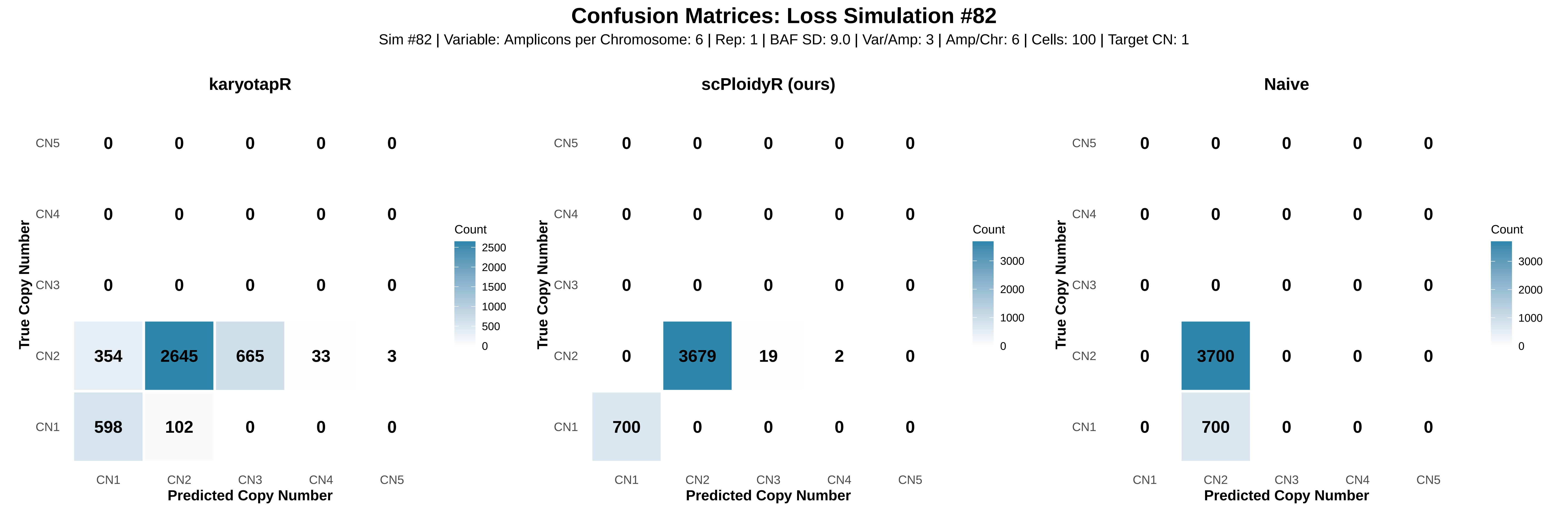

Confusion Matrices: Loss Simulation #82
Sim #82 | Variable: Amplicons per Chromosome: 6 | Rep: 1 | BAF SD: 9.0 | Var/Amp: 3 | Amp/Chr: 6 | Cells: 100 | Target CN: 1
karyotapR
scPloidyR (ours)
Naive
True Copy Number
Predicted Copy Number
Count
CN1
CN2
CN3
CN4
CN5
354
2645
665
33
3
598
102
3679
19
2
700
3700

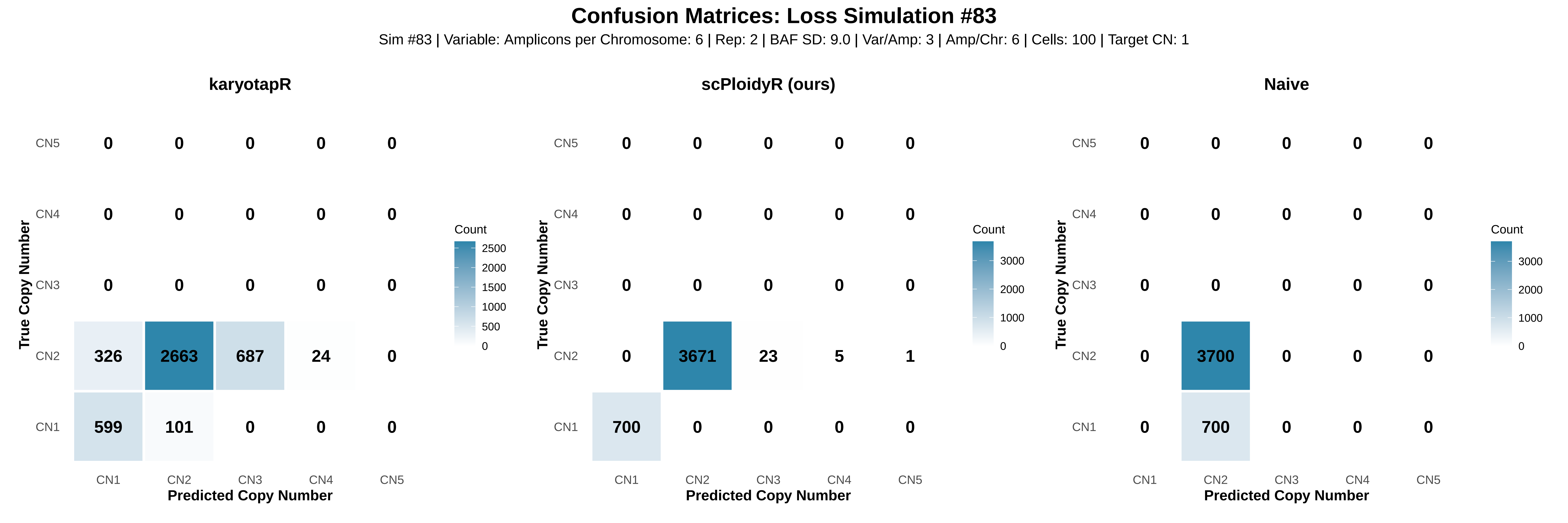

Confusion Matrices: Loss Simulation #83
Sim #83 | Variable: Amplicons per Chromosome: 6 | Rep: 2 | BAF SD: 9.0 | Var/Amp: 3 | Amp/Chr: 6 | Cells: 100 | Target CN: 1
karyotapR
scPloidyR (ours)
Naive
True Copy Number
Predicted Copy Number
CN1
CN2
CN3
CN4
CN5
Count
326
2663
687
24
599
101
3671
23
5
1
700
3700
0
500
1000
1500
2000
2500
3000

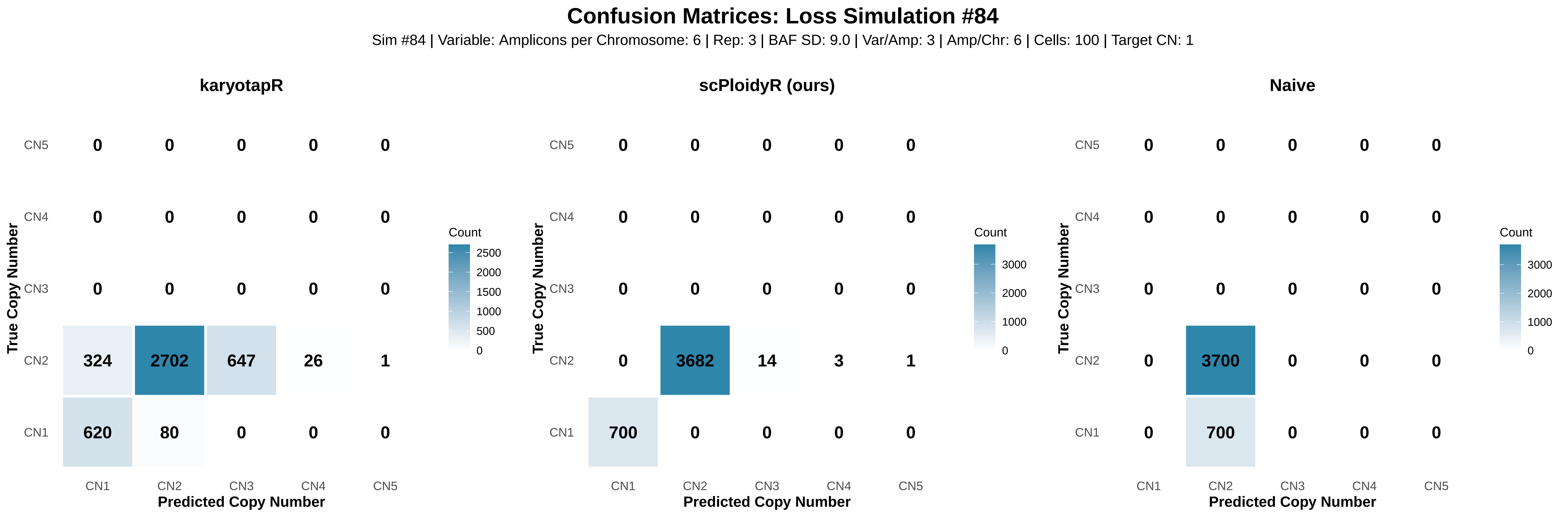

Confusion Matrices: Loss Simulation #84
Sim #84 | Variable: Amplicons per Chromosome: 6 | Rep: 3 | BAF SD: 9.0 | Var/Amp: 3 | Amp/Chr: 6 | Cells: 100 | Target CN: 1
karyotapR
scPloidyR (ours)
Naive
True Copy Number
Predicted Copy Number
CN1
CN2
CN3
CN4
CN5
Count
2500
2000
1500
1000
500
0
3000
324
2702
647
26
1
620
80
3682
14
3
700
3700

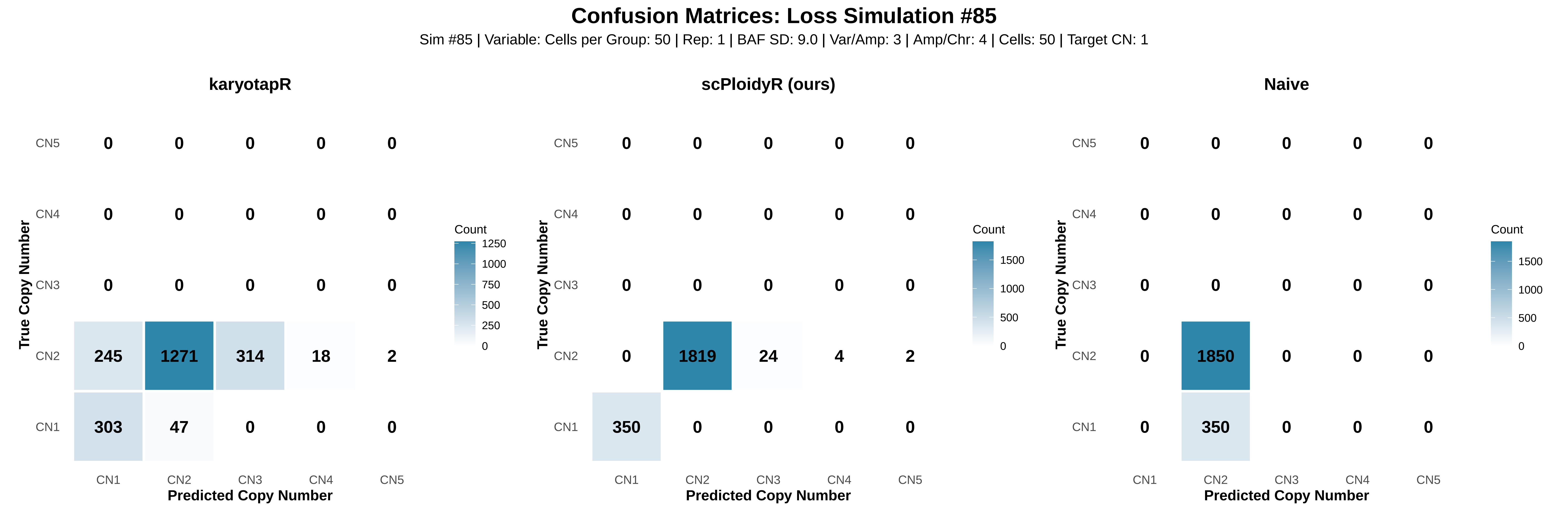
Confusion Matrices: Loss Simulation #85
Sim #85 | Variable: Cells per Group: 50 | Rep: 1 | BAF SD: 9.0 | Var/Amp: 3 | Amp/Chr: 4 | Cells: 50 | Target CN: 1
karyotapR
scPloidyR (ours)
Naive
True Copy Number
Predicted Copy Number
Count
CN5 0 0 0 0 0
CN4 0 0 0 0 0
CN3 0 0 0 0 0
CN2 245 1271 314 18 2
CN1 303 47 0 0 0
CN1 CN2 CN3 CN4 CN5
1250 1000 750 500 250 0
CN5 0 0 0 0 0
CN4 0 0 0 0 0
CN3 0 0 0 0 0
CN2 0 1819 24 4 2
CN1 350 0 0 0 0
CN1 CN2 CN3 CN4 CN5
1500 1000 500 0
CN5 0 0 0 0 0
CN4 0 0 0 0 0
CN3 0 0 0 0 0
CN2 0 1850 0 0 0
CN1 0 350 0 0 0
CN1 CN2 CN3 CN4 CN5
1500 1000 500 0

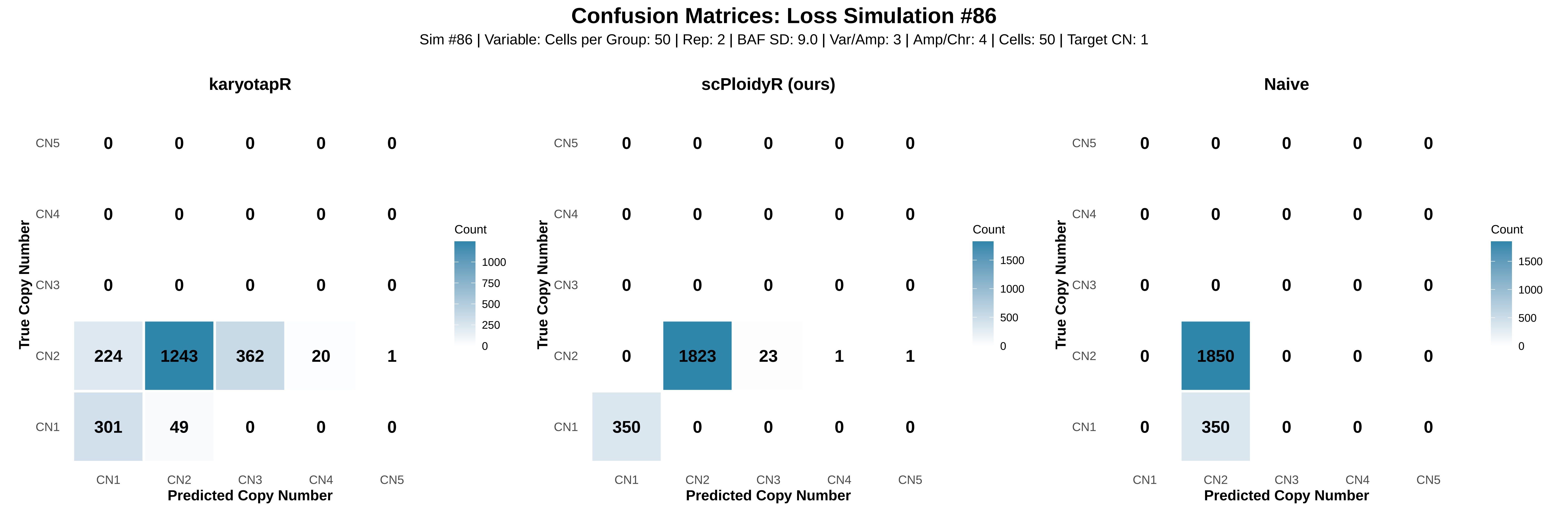

Confusion Matrices: Loss Simulation #86
Sim #86 | Variable: Cells per Group: 50 | Rep: 2 | BAF SD: 9.0 | Var/Amp: 3 | Amp/Chr: 4 | Cells: 50 | Target CN: 1
karyotapR
scPloidyR (ours)
Naive
True Copy Number
Predicted Copy Number
Count
CN1
CN2
CN3
CN4
CN5
224
1243
362
20
1
301
49
1823
23
350
1850
0
250
500
750
1000
1500

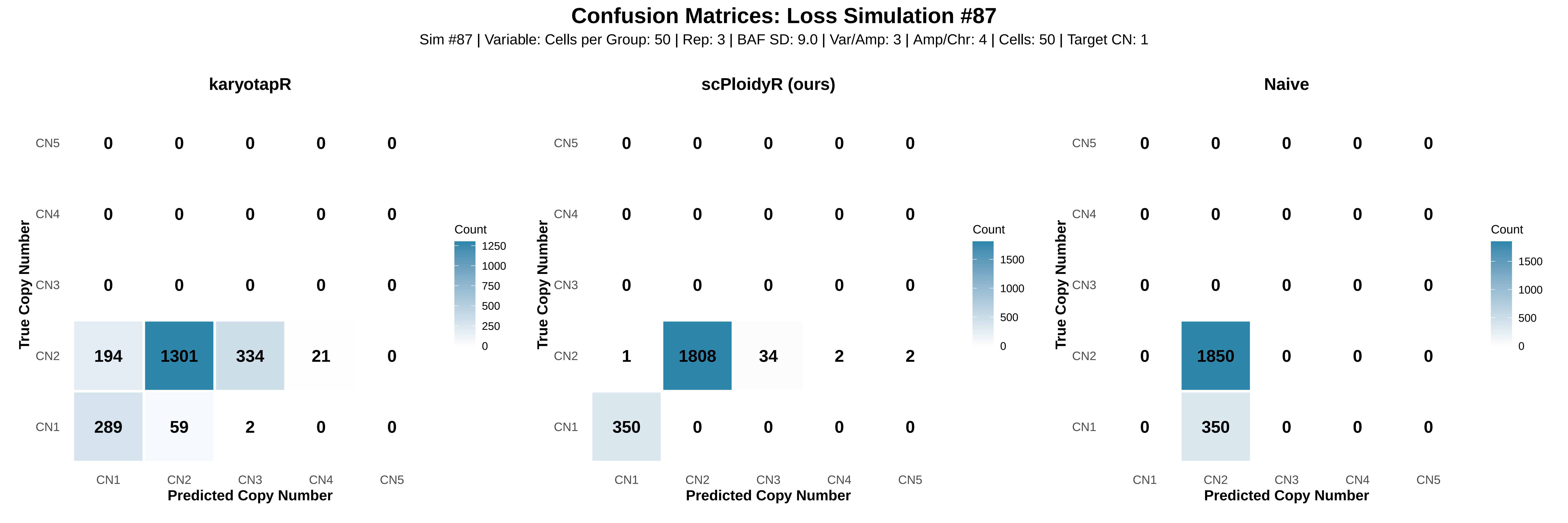
Confusion Matrices: Loss Simulation #87
Sim #87 | Variable: Cells per Group: 50 | Rep: 3 | BAF SD: 9.0 | Var/Amp: 3 | Amp/Chr: 4 | Cells: 50 | Target CN: 1
karyotapR
scPloidyR (ours)
Naive
True Copy Number
Predicted Copy Number
CN1
CN2
CN3
CN4
CN5
Count
194
1301
334
21
289
59
2
1
1808
34
350
1850
0
250
500
750
1000
1250
1500

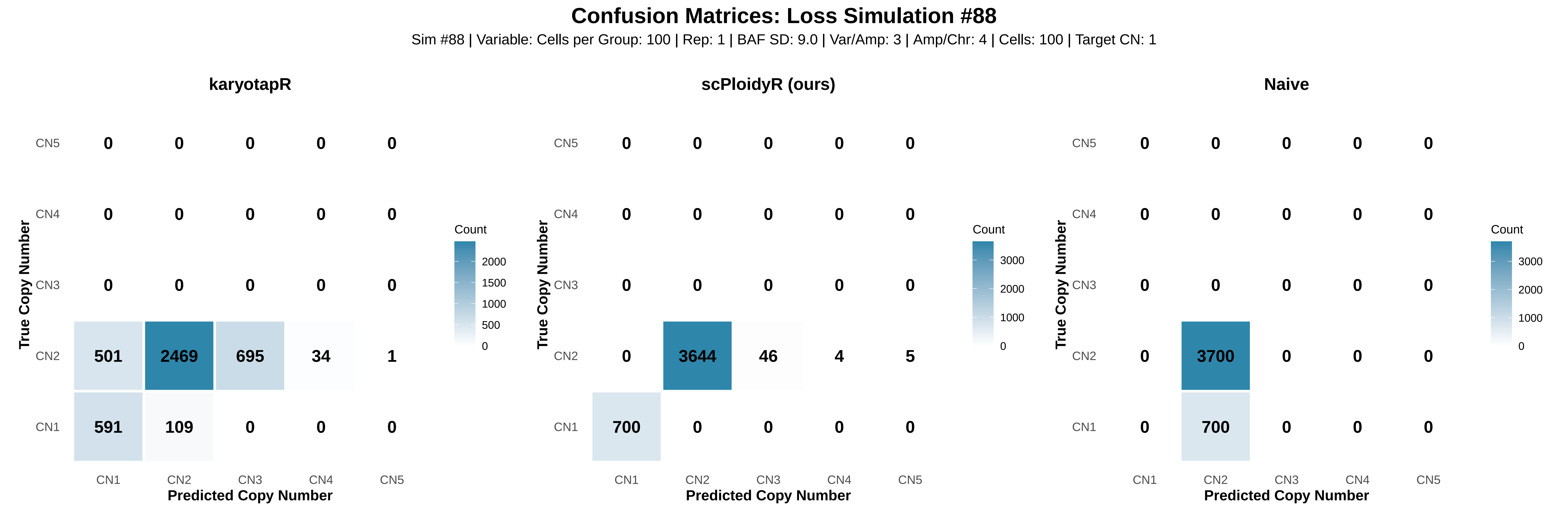
Confusion Matrices: Loss Simulation #88
Sim #88 | Variable: Cells per Group: 100 | Rep: 1 | BAF SD: 9.0 | Var/Amp: 3 | Amp/Chr: 4 | Cells: 100 | Target CN: 1
karyotapR
CN5 0 0 0 0 0
CN4 0 0 0 0 0
CN3 0 0 0 0 0
CN2 501 2469 695 34 1
CN1 591 109 0 0 0
CN1 CN2 CN3 CN4 CN5
True Copy Number
Predicted Copy Number
Count
2000
1500
1000
500
0
scPloidyR (ours)
CN5 0 0 0 0 0
CN4 0 0 0 0 0
CN3 0 0 0 0 0
CN2 0 3644 46 4 5
CN1 700 0 0 0 0
CN1 CN2 CN3 CN4 CN5
True Copy Number
Predicted Copy Number
Count
3000
2000
1000
0
Naive
CN5 0 0 0 0 0
CN4 0 0 0 0 0
CN3 0 0 0 0 0
CN2 0 3700 0 0 0
CN1 0 700 0 0 0
CN1 CN2 CN3 CN4 CN5
True Copy Number
Predicted Copy Number
Count
3000
2000
1000
0

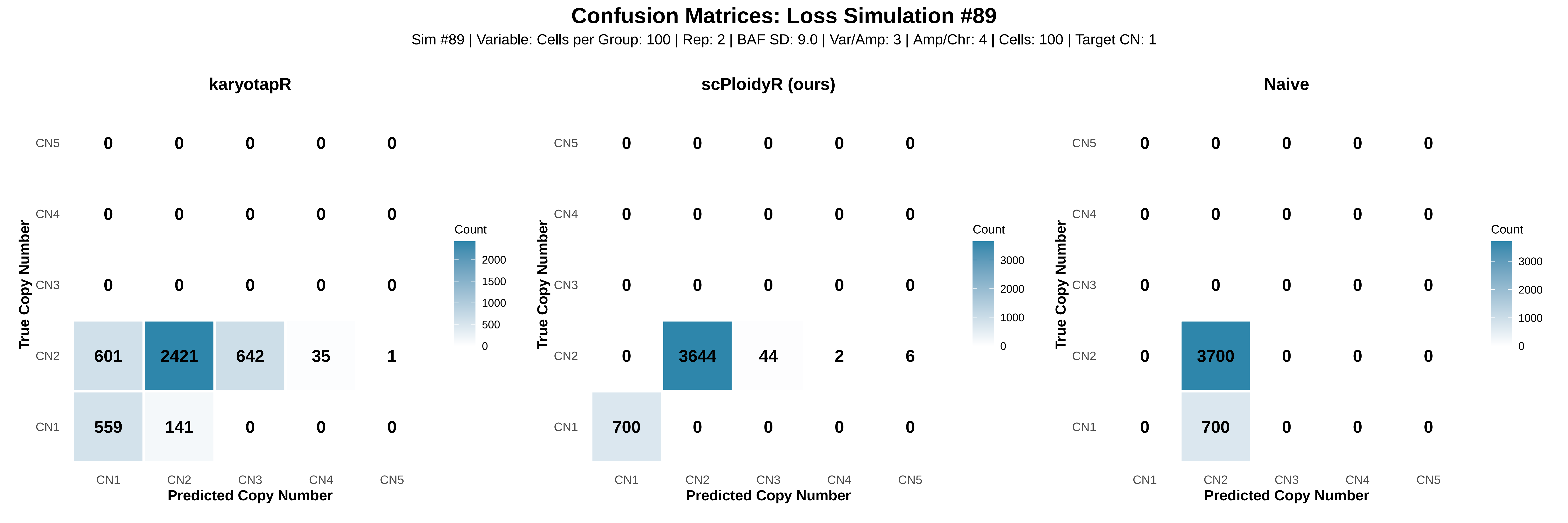

Confusion Matrices: Loss Simulation #89
Sim #89 | Variable: Cells per Group: 100 | Rep: 2 | BAF SD: 9.0 | Var/Amp: 3 | Amp/Chr: 4 | Cells: 100 | Target CN: 1
karyotapR
scPloidyR (ours)
Naive
True Copy Number
Predicted Copy Number
CN1
CN2
CN3
CN4
CN5
Count
601
2421
642
35
1
559
141
3644
44
2
6
700
3700

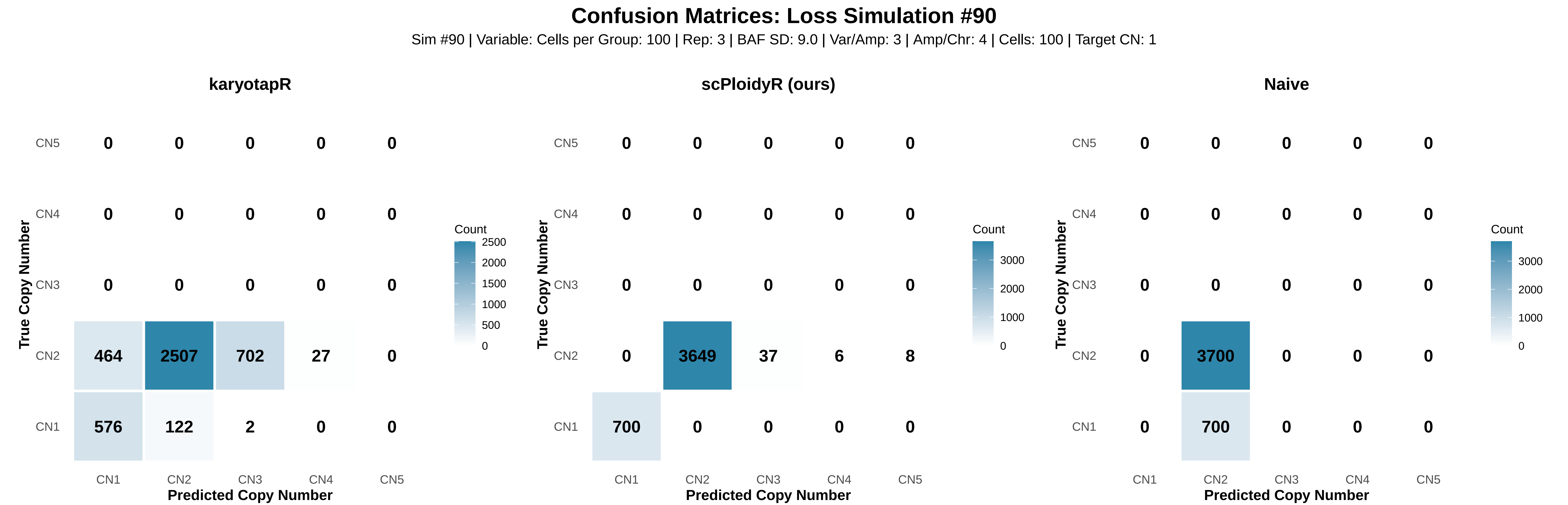

Confusion Matrices: Loss Simulation #90
Sim #90 | Variable: Cells per Group: 100 | Rep: 3 | BAF SD: 9.0 | Var/Amp: 3 | Amp/Chr: 4 | Cells: 100 | Target CN: 1
karyotapR
scPloidyR (ours)
Naive
True Copy Number
Predicted Copy Number
Count
CN1
CN2
CN3
CN4
CN5
464
2507
702
27
576
122
2
3649
37
6
8
700
3700
2500
2000
1500
1000
500
3000
0

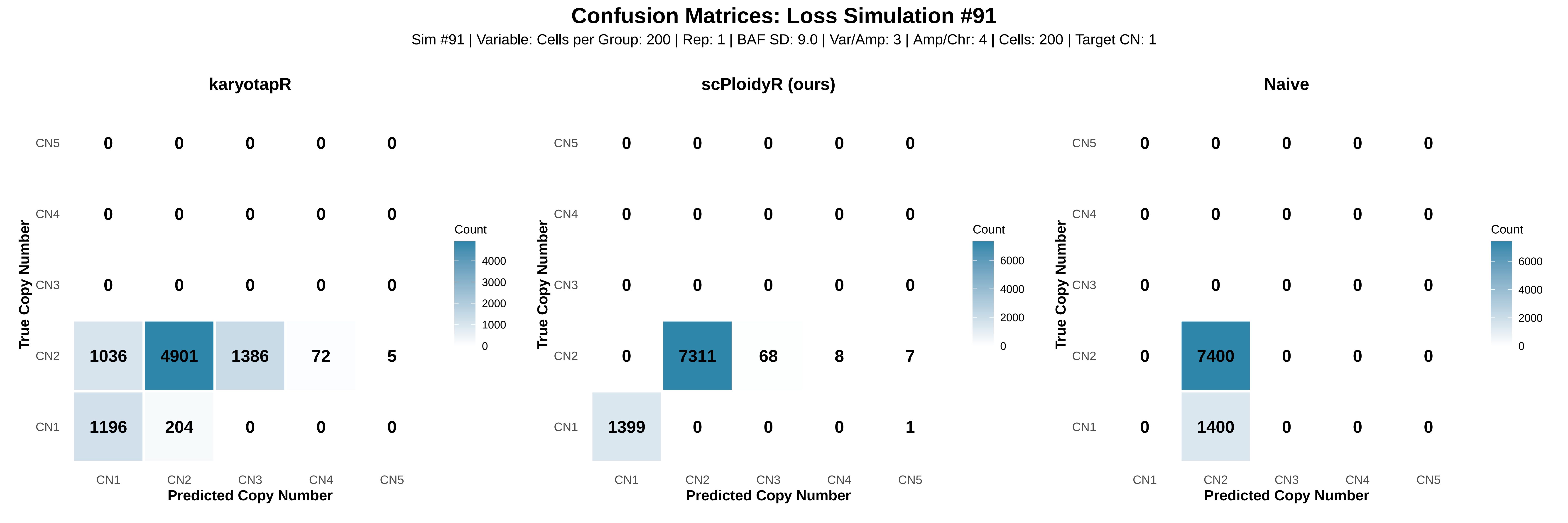

Confusion Matrices: Loss Simulation #91
Sim #91 | Variable: Cells per Group: 200 | Rep: 1 | BAF SD: 9.0 | Var/Amp: 3 | Amp/Chr: 4 | Cells: 200 | Target CN: 1
karyotapR
scPloidyR (ours)
Naive
True Copy Number
Predicted Copy Number
CN1
CN2
CN3
CN4
CN5
Count
1036
4901
1386
72
5
1196
204
0
7311
68
8
7
1399
1
7400
1400
0
1000
2000
3000
4000
6000

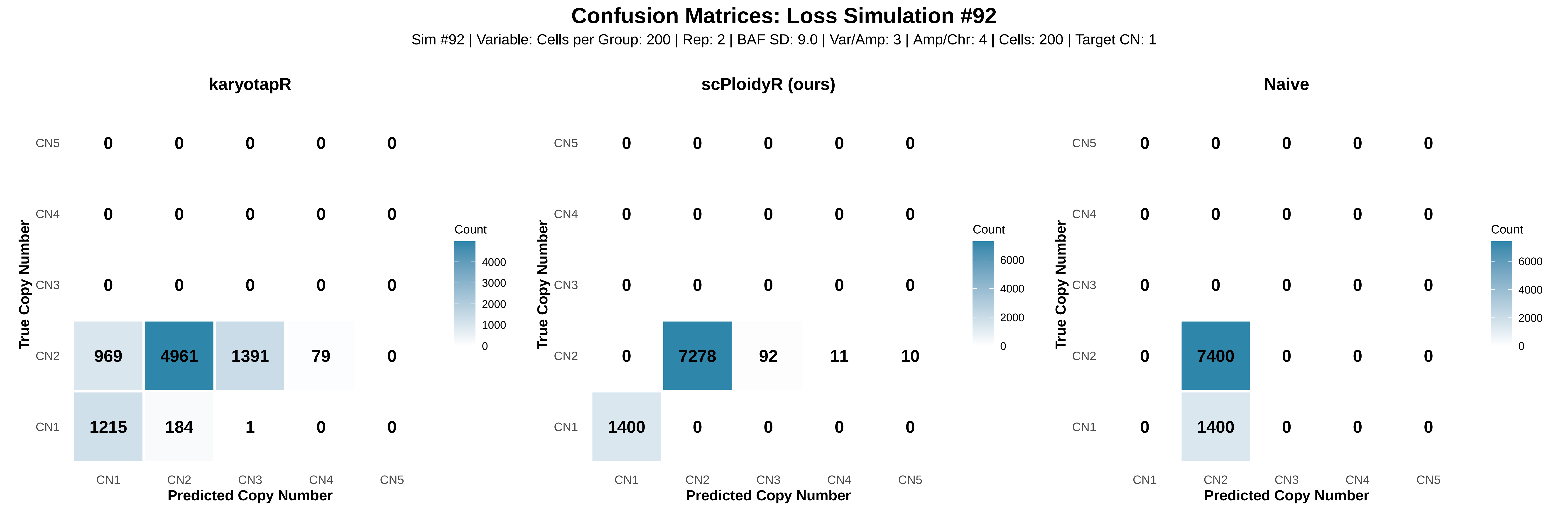
Confusion Matrices: Loss Simulation #92
Sim #92 | Variable: Cells per Group: 200 | Rep: 2 | BAF SD: 9.0 | Var/Amp: 3 | Amp/Chr: 4 | Cells: 200 | Target CN: 1
karyotapR
scPloidyR (ours)
Naive
True Copy Number
Predicted Copy Number
CN1
CN2
CN3
CN4
CN5
Count
0
1000
2000
3000
4000
6000
969
4961
1391
79
1215
184
1
7278
92
11
10
1400
7400

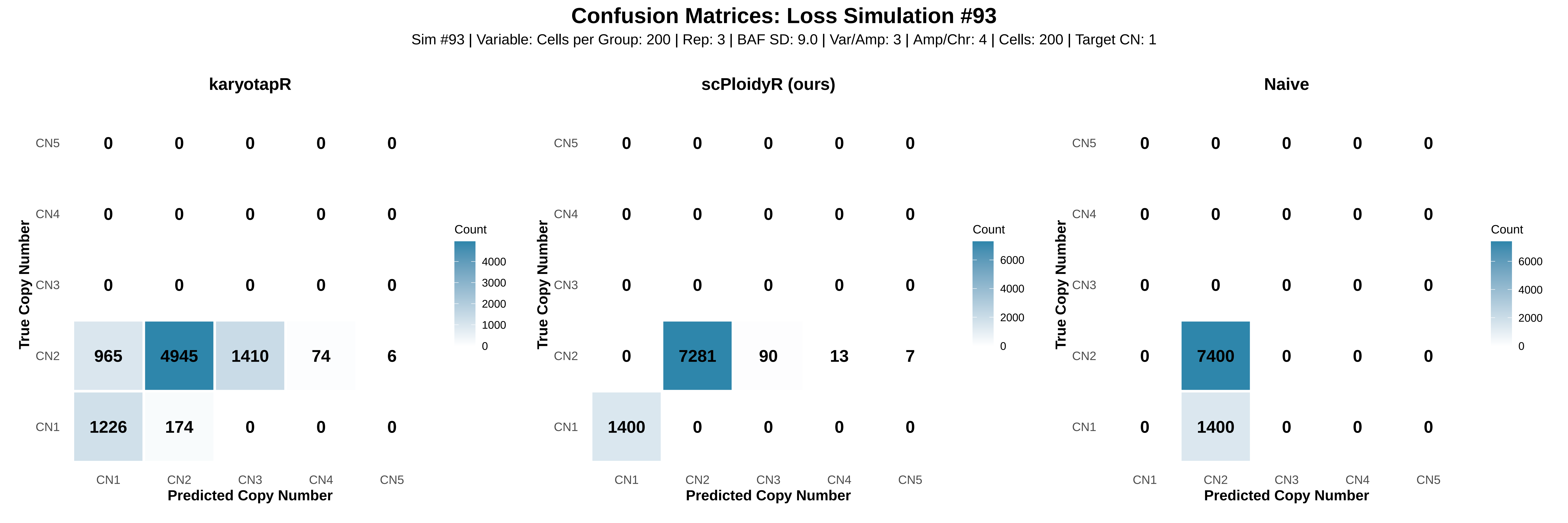

Confusion Matrices: Loss Simulation #93
Sim #93 | Variable: Cells per Group: 200 | Rep: 3 | BAF SD: 9.0 | Var/Amp: 3 | Amp/Chr: 4 | Cells: 200 | Target CN: 1
karyotapR
scPloidyR (ours)
Naive
True Copy Number
Predicted Copy Number
Count
CN1
CN2
CN3
CN4
CN5
0
0
0
0
0
0
0
0
0
0
0
0
0
0
0
965
4945
1410
74
6
1226
174
0
0
0
7281
90
13
7
1400
7400
4000
3000
2000
1000
6000

Confusion Matrices: Loss Simulation #94

Sim #94 | Variable: Heterozygosity Rate: 0.0% | Rep: 1 | BAF SD: 9.0 | Var/Amp: 3 | Amp/Chr: 4 | Cells: 100 | Target CN: 1

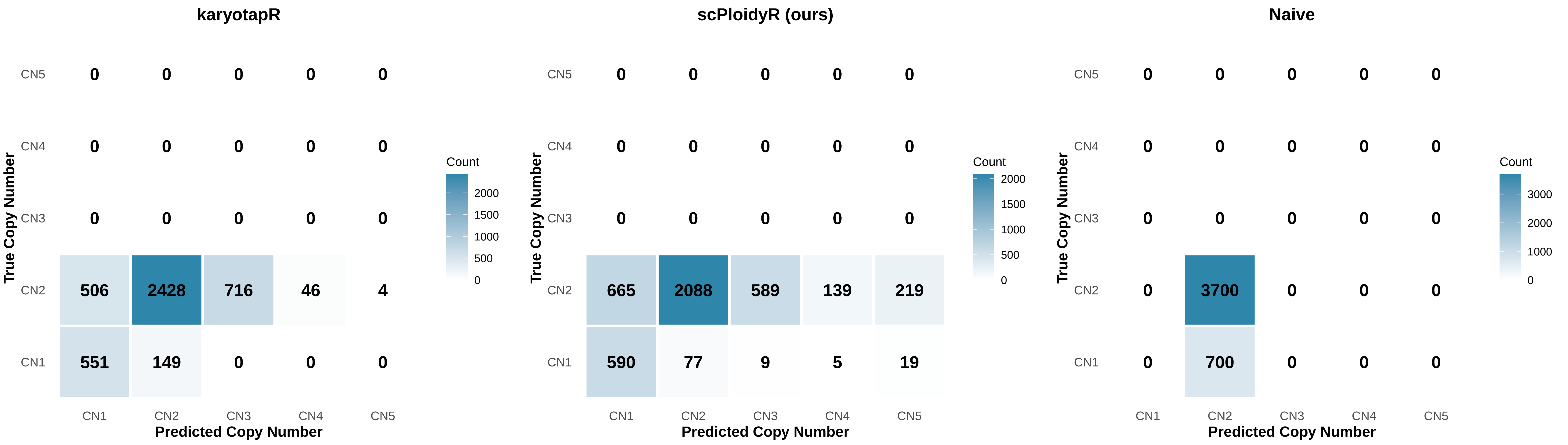

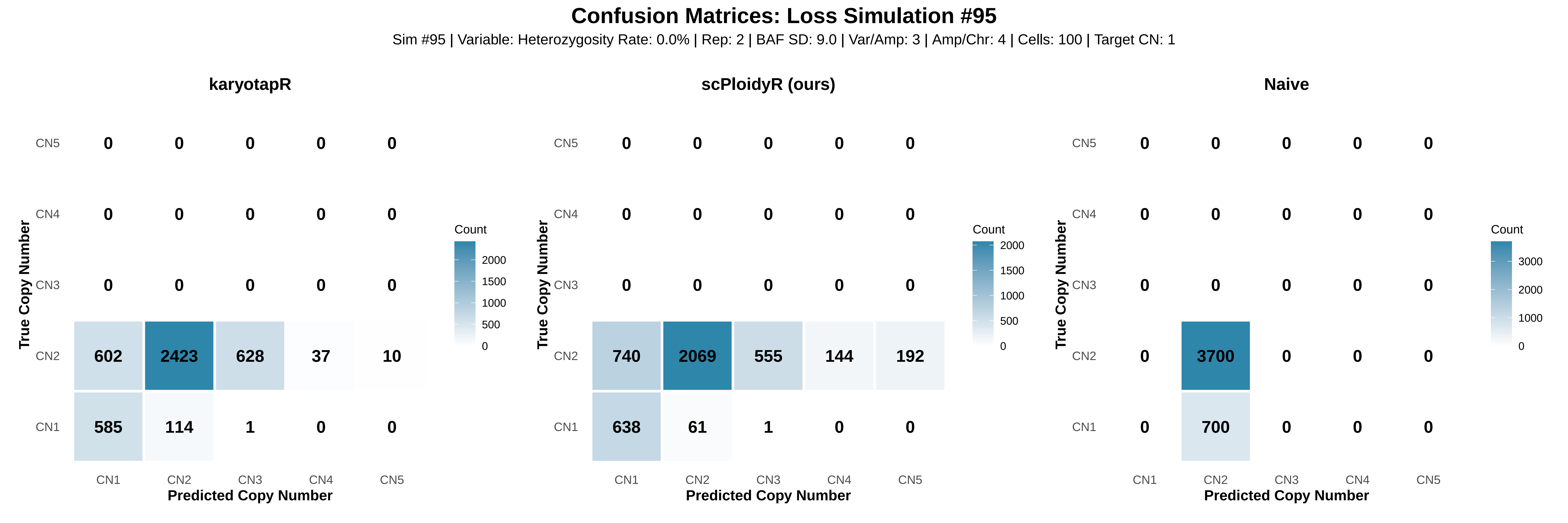

Confusion Matrices: Loss Simulation #95
Sim #95 | Variable: Heterozygosity Rate: 0.0% | Rep: 2 | BAF SD: 9.0 | Var/Amp: 3 | Amp/Chr: 4 | Cells: 100 | Target CN: 1
karyotapR
scPloidyR (ours)
Naive
True Copy Number
Predicted Copy Number
Count
CN1
CN2
CN3
CN4
CN5
602
2423
628
37
10
585
114
1
740
2069
555
144
192
638
61
3700
700

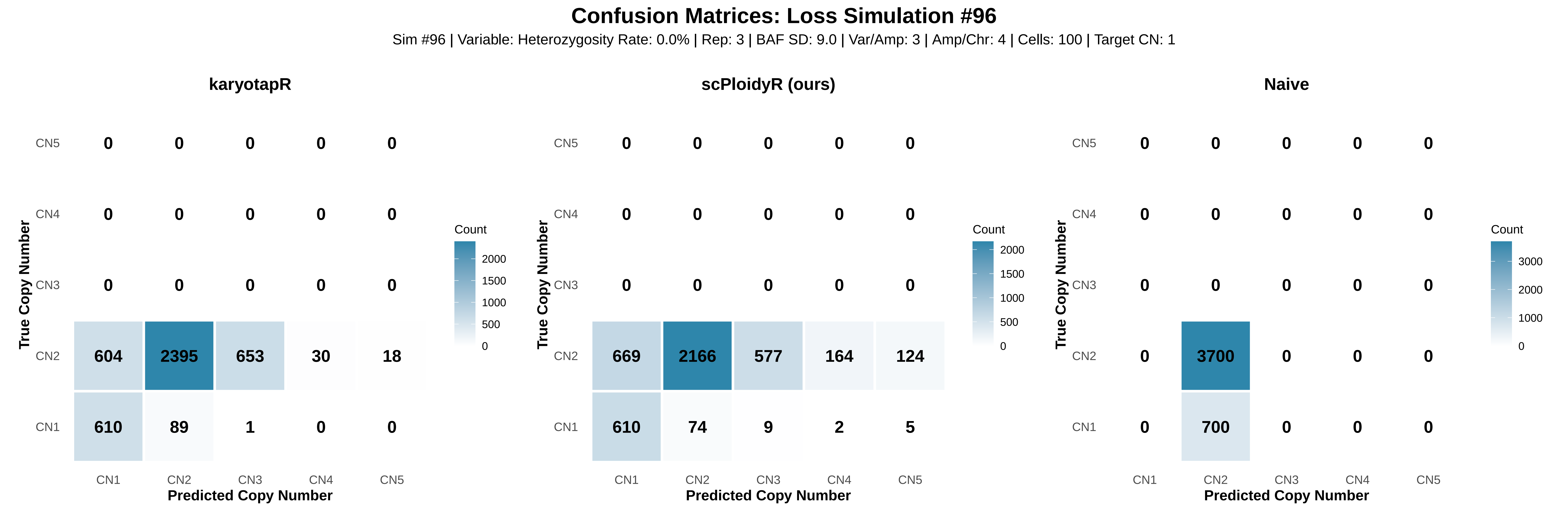
Confusion Matrices: Loss Simulation #96
Sim #96 | Variable: Heterozygosity Rate: 0.0% | Rep: 3 | BAF SD: 9.0 | Var/Amp: 3 | Amp/Chr: 4 | Cells: 100 | Target CN: 1
karyotapR
scPloidyR (ours)
Naive
True Copy Number
Predicted Copy Number
Count
CN1
CN2
CN3
CN4
CN5
604
2395
653
30
18
610
89
1
669
2166
577
164
124
74
9
2
5
3700
700
0
500
1000
1500
2000
3000

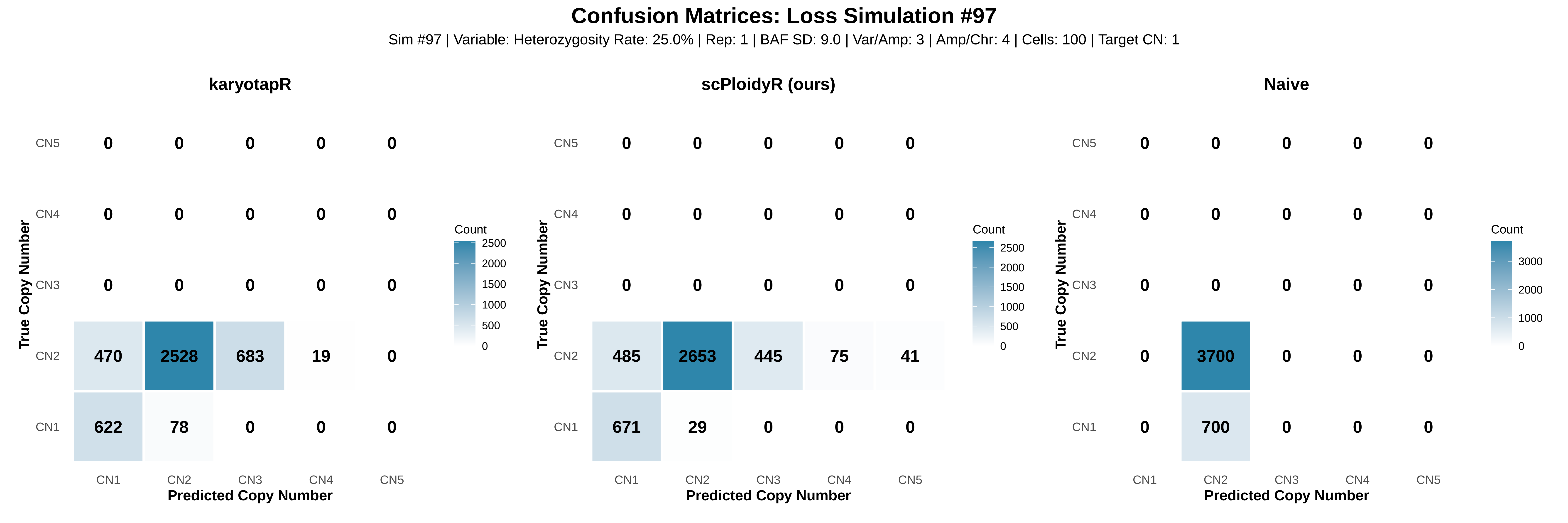
Confusion Matrices: Loss Simulation #97
Sim #97 | Variable: Heterozygosity Rate: 25.0% | Rep: 1 | BAF SD: 9.0 | Var/Amp: 3 | Amp/Chr: 4 | Cells: 100 | Target CN: 1
karyotapR
scPloidyR (ours)
Naive
True Copy Number
Predicted Copy Number
Count
CN1
CN2
CN3
CN4
CN5
470
2528
683
19
622
78
485
2653
445
75
41
671
29
3700
700

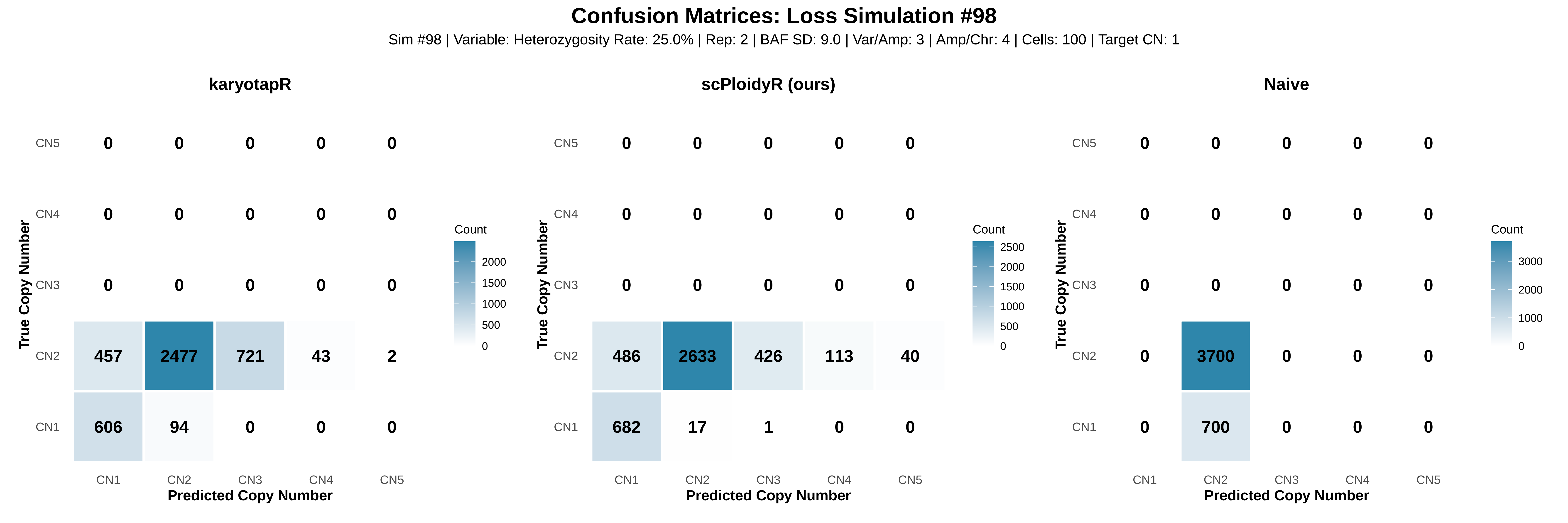
Confusion Matrices: Loss Simulation #98
Sim #98 | Variable: Heterozygosity Rate: 25.0% | Rep: 2 | BAF SD: 9.0 | Var/Amp: 3 | Amp/Chr: 4 | Cells: 100 | Target CN: 1
karyotapR
scPloidyR (ours)
Naive
True Copy Number
Predicted Copy Number
Count
CN1
CN2
CN3
CN4
CN5
457
2477
721
43
2
606
94
486
2633
426
113
40
682
17
1
3700
700
0
500
1000
1500
2000
2500
3000

Confusion Matrices: Loss Simulation #99

Sim #99 | Variable: Heterozygosity Rate: 25.0% | Rep: 3 | BAF SD: 9.0 | Var/Amp: 3 | Amp/Chr: 4 | Cells: 100 | Target CN: 1

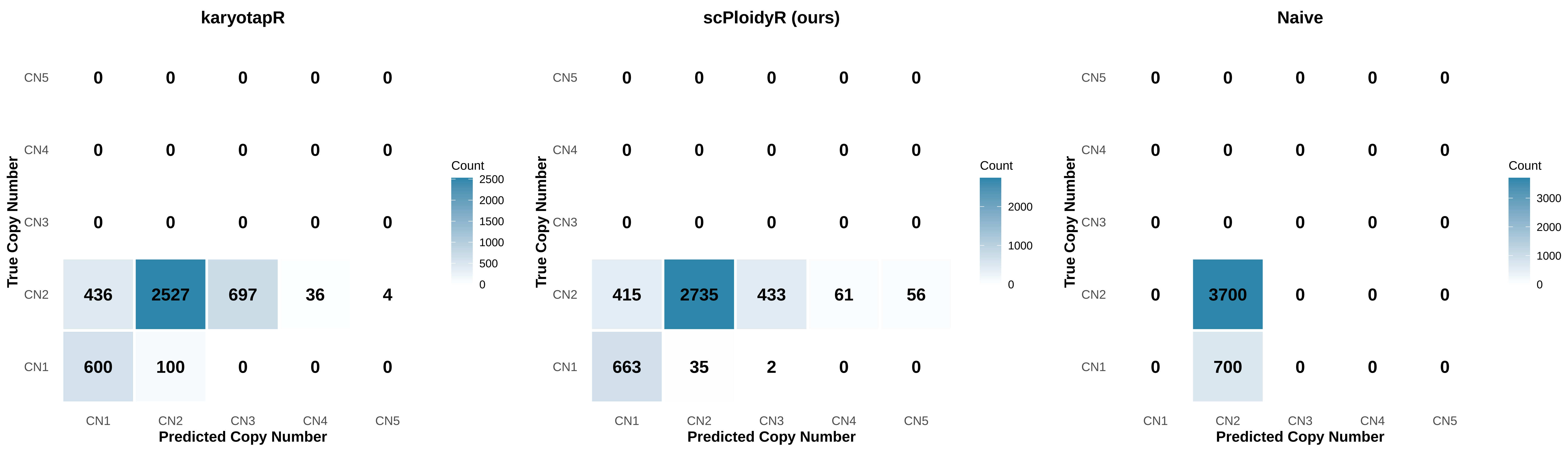

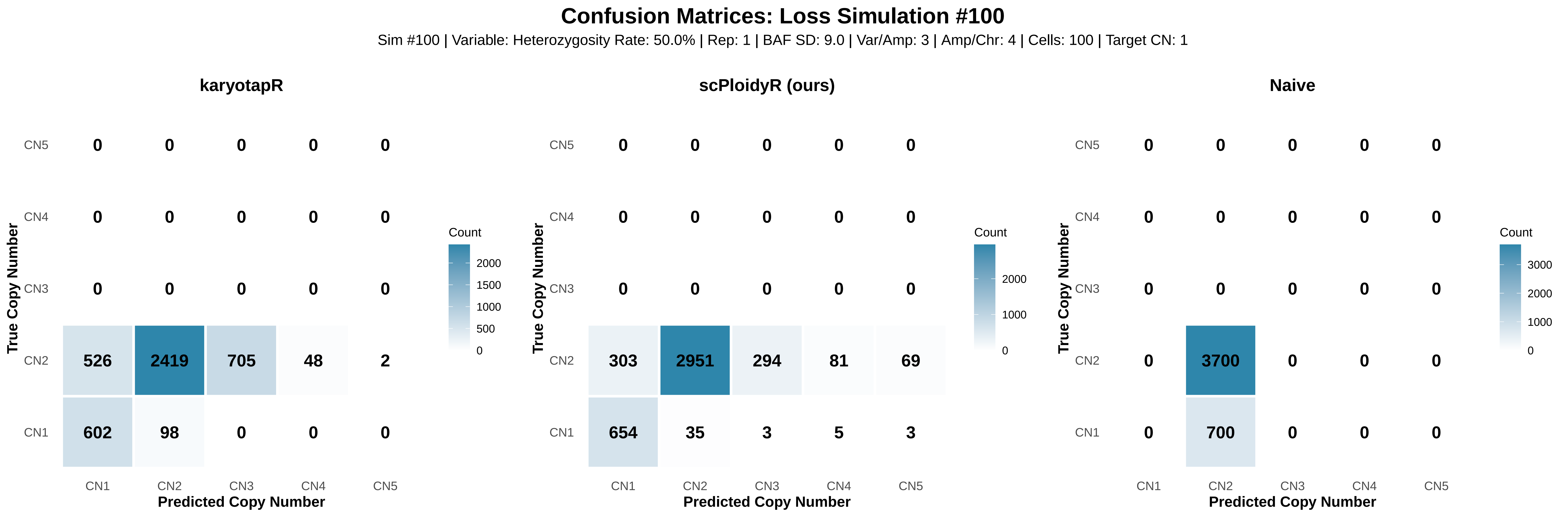
Confusion Matrices: Loss Simulation #100
Sim #100 | Variable: Heterozygosity Rate: 50.0% | Rep: 1 | BAF SD: 9.0 | Var/Amp: 3 | Amp/Chr: 4 | Cells: 100 | Target CN: 1
karyotapR
scPloidyR (ours)
Naive
True Copy Number
Predicted Copy Number
CN1
CN2
CN3
CN4
CN5
Count
karyotapR: CN5: 0 0 0 0 0; CN4: 0 0 0 0 0; CN3: 0 0 0 0 0; CN2: 526 2419 705 48 2; CN1: 602 98 0 0 0
2000
1500
1000
500
0
scPloidyR (ours): CN5: 0 0 0 0 0; CN4: 0 0 0 0 0; CN3: 0 0 0 0 0; CN2: 303 2951 294 81 69; CN1: 654 35 3 5 3
2000
1000
0
Naive: CN5: 0 0 0 0 0; CN4: 0 0 0 0 0; CN3: 0 0 0 0 0; CN2: 0 3700 0 0 0; CN1: 0 700 0 0 0
3000
2000
1000
0

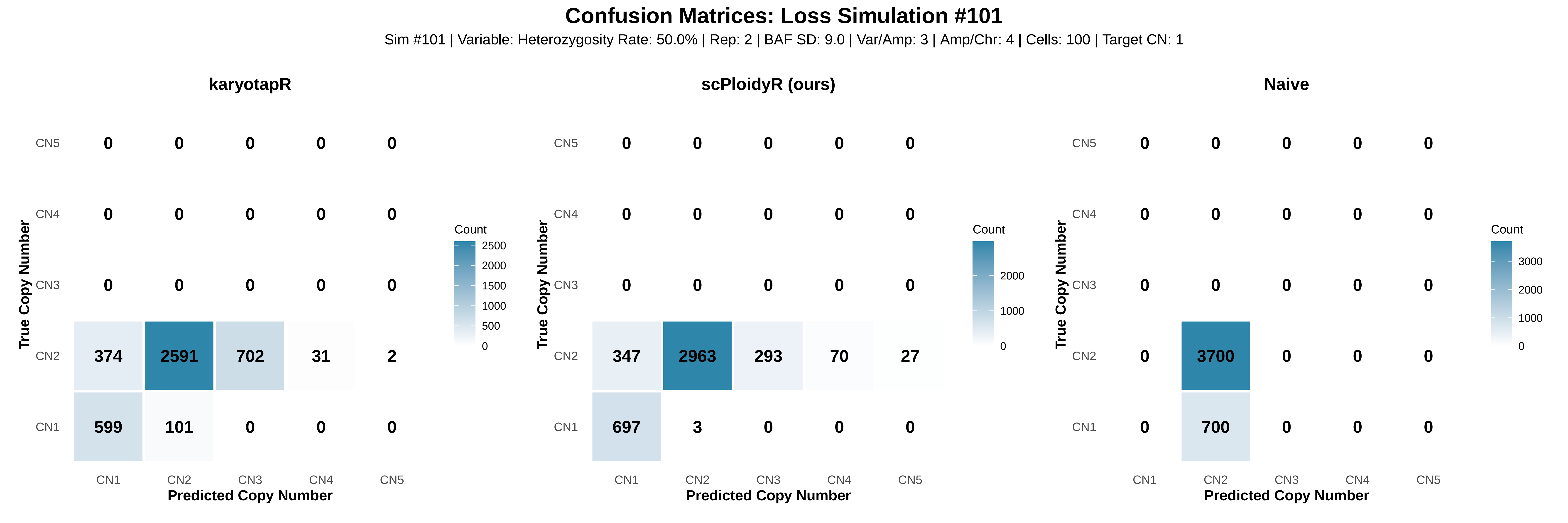

Confusion Matrices: Loss Simulation #101
Sim #101 | Variable: Heterozygosity Rate: 50.0% | Rep: 2 | BAF SD: 9.0 | Var/Amp: 3 | Amp/Chr: 4 | Cells: 100 | Target CN: 1
karyotapR
scPloidyR (ours)
Naive
True Copy Number
Predicted Copy Number
CN1
CN2
CN3
CN4
CN5
Count
karyotapR: CN2 row: 374, 2591, 702, 31, 2; CN1 row: 599, 101, 0, 0, 0; CN3–CN5 rows: 0
scPloidyR (ours): CN2 row: 347, 2963, 293, 70, 27; CN1 row: 697, 3, 0, 0, 0; CN3–CN5 rows: 0
Naive: CN2 row: 0, 3700, 0, 0, 0; CN1 row: 0, 700, 0, 0, 0; CN3–CN5 rows: 0
2500
2000
1500
1000
500
0
3000

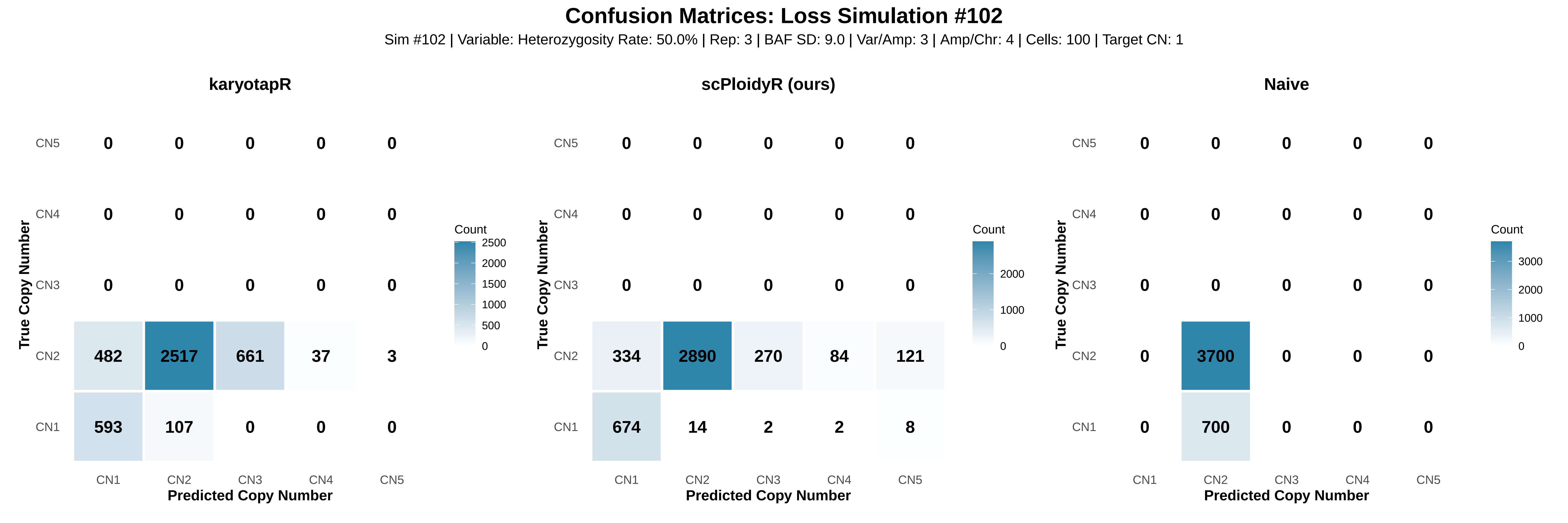
Confusion Matrices: Loss Simulation #102
Sim #102 | Variable: Heterozygosity Rate: 50.0% | Rep: 3 | BAF SD: 9.0 | Var/Amp: 3 | Amp/Chr: 4 | Cells: 100 | Target CN: 1
karyotapR
scPloidyR (ours)
Naive
True Copy Number
Predicted Copy Number
CN1
CN2
CN3
CN4
CN5
Count
2500
2000
1500
1000
500
0
3000
karyotapR: CN5 row: 0 0 0 0 0; CN4 row: 0 0 0 0 0; CN3 row: 0 0 0 0 0; CN2 row: 482 2517 661 37 3; CN1 row: 593 107 0 0 0
scPloidyR (ours): CN5 row: 0 0 0 0 0; CN4 row: 0 0 0 0 0; CN3 row: 0 0 0 0 0; CN2 row: 334 2890 270 84 121; CN1 row: 674 14 2 2 8
Naive: CN5 row: 0 0 0 0 0; CN4 row: 0 0 0 0 0; CN3 row: 0 0 0 0 0; CN2 row: 0 3700 0 0 0; CN1 row: 0 700 0 0 0

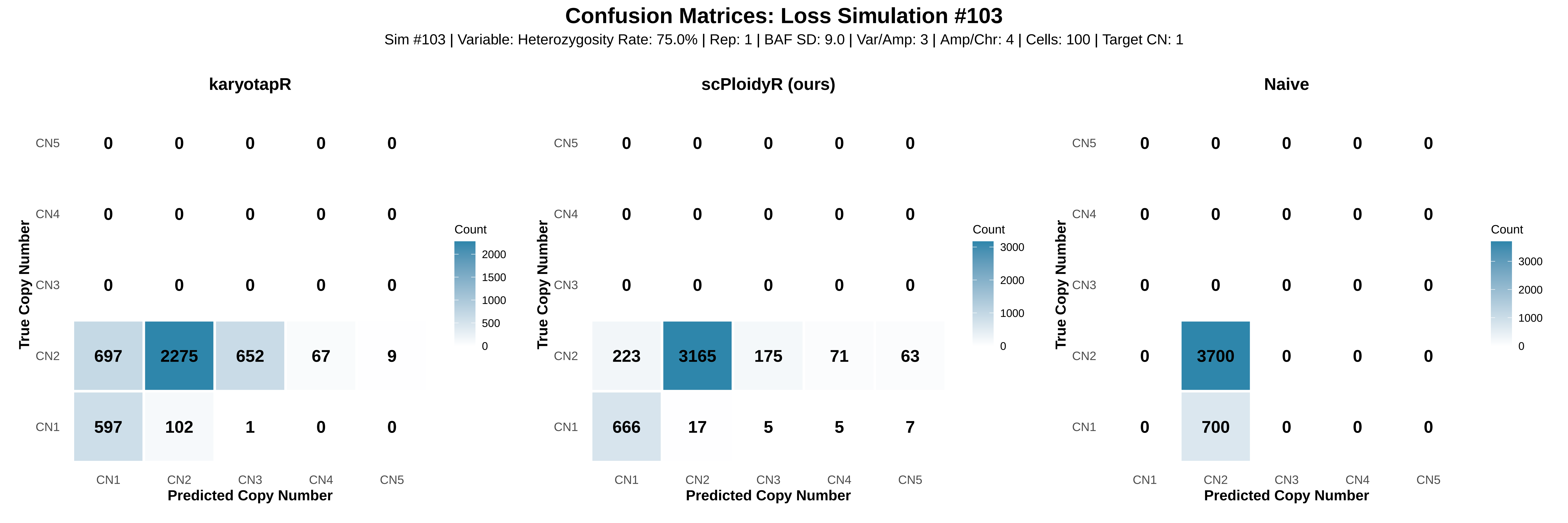
Confusion Matrices: Loss Simulation #103
Sim #103 | Variable: Heterozygosity Rate: 75.0% | Rep: 1 | BAF SD: 9.0 | Var/Amp: 3 | Amp/Chr: 4 | Cells: 100 | Target CN: 1
karyotapR
scPloidyR (ours)
Naive
True Copy Number
Predicted Copy Number
CN1
CN2
CN3
CN4
CN5
Count
karyotapR: CN5: 0 0 0 0 0; CN4: 0 0 0 0 0; CN3: 0 0 0 0 0; CN2: 697 2275 652 67 9; CN1: 597 102 1 0 0
scPloidyR (ours): CN5: 0 0 0 0 0; CN4: 0 0 0 0 0; CN3: 0 0 0 0 0; CN2: 223 3165 175 71 63; CN1: 666 17 5 5 7
Naive: CN5: 0 0 0 0 0; CN4: 0 0 0 0 0; CN3: 0 0 0 0 0; CN2: 0 3700 0 0 0; CN1: 0 700 0 0 0
0
500
1000
1500
2000
3000

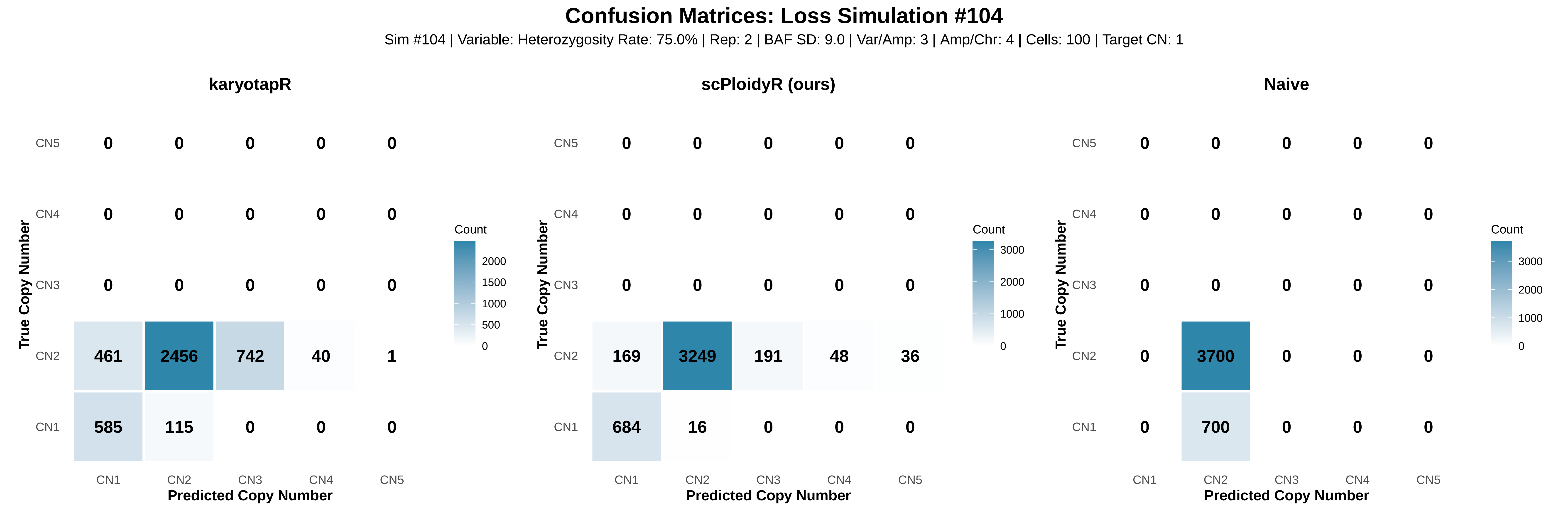
Confusion Matrices: Loss Simulation #104
Sim #104 | Variable: Heterozygosity Rate: 75.0% | Rep: 2 | BAF SD: 9.0 | Var/Amp: 3 | Amp/Chr: 4 | Cells: 100 | Target CN: 1
karyotapR
scPloidyR (ours)
Naive
True Copy Number
Predicted Copy Number
CN1
CN2
CN3
CN4
CN5
Count
0
500
1000
1500
2000
3000
461
2456
742
40
1
585
115
169
3249
191
48
36
684
16
3700
700

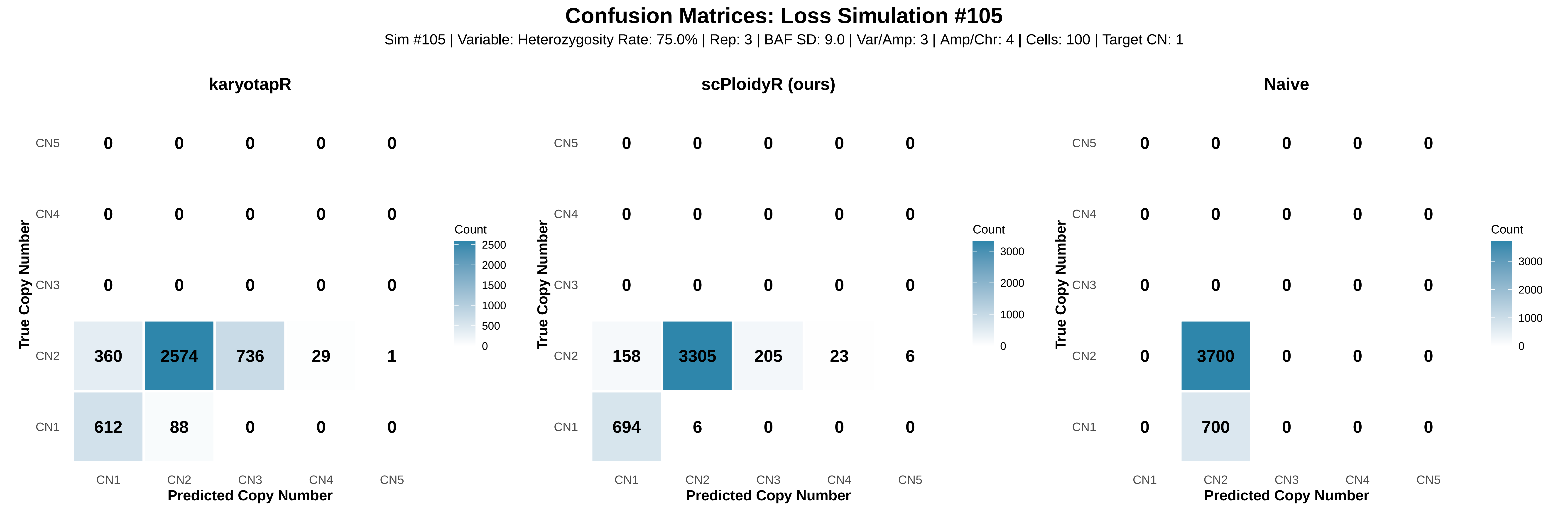
Confusion Matrices: Loss Simulation #105
Sim #105 | Variable: Heterozygosity Rate: 75.0% | Rep: 3 | BAF SD: 9.0 | Var/Amp: 3 | Amp/Chr: 4 | Cells: 100 | Target CN: 1
karyotapR
scPloidyR (ours)
Naive
True Copy Number
Predicted Copy Number
CN1
CN2
CN3
CN4
CN5
Count
karyotapR: CN2 row: 360, 2574, 736, 29, 1; CN1 row: 612, 88, 0, 0, 0; CN3–CN5 rows: 0
scPloidyR (ours): CN2 row: 158, 3305, 205, 23, 6; CN1 row: 694, 6, 0, 0, 0; CN3–CN5 rows: 0
Naive: CN2 row: 0, 3700, 0, 0, 0; CN1 row: 0, 700, 0, 0, 0; CN3–CN5 rows: 0
2500
2000
1500
1000
500
0
3000

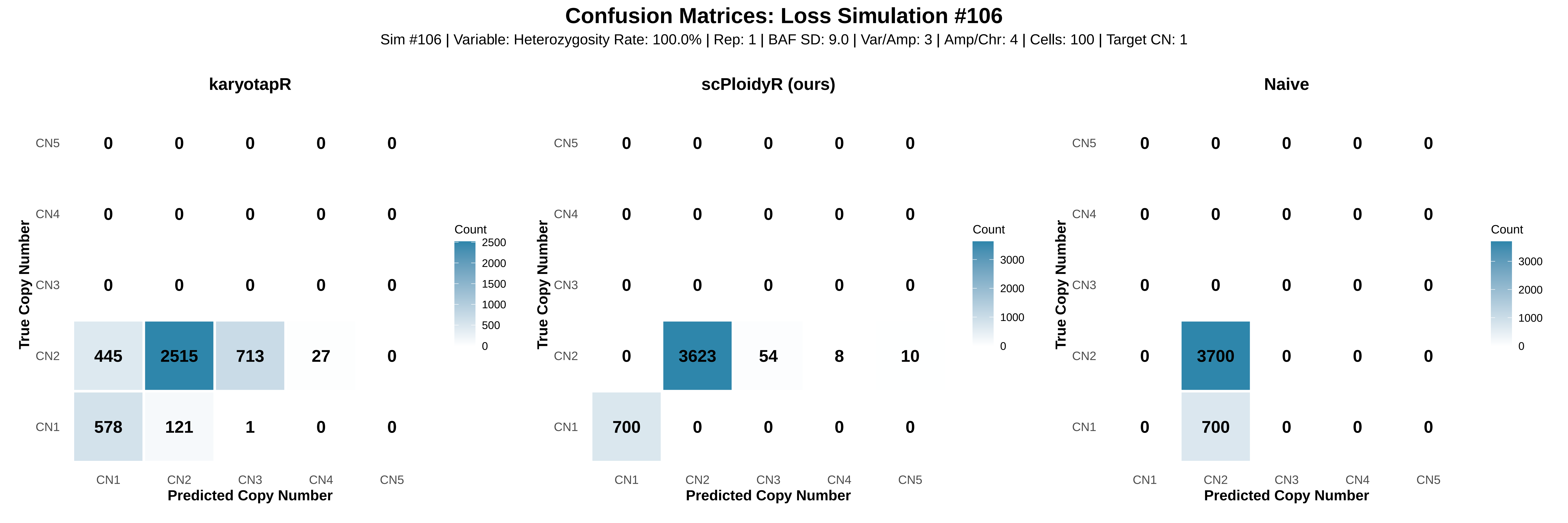
Confusion Matrices: Loss Simulation #106
Sim #106 | Variable: Heterozygosity Rate: 100.0% | Rep: 1 | BAF SD: 9.0 | Var/Amp: 3 | Amp/Chr: 4 | Cells: 100 | Target CN: 1
karyotapR
scPloidyR (ours)
Naive
True Copy Number
Predicted Copy Number
Count
CN1
CN2
CN3
CN4
CN5

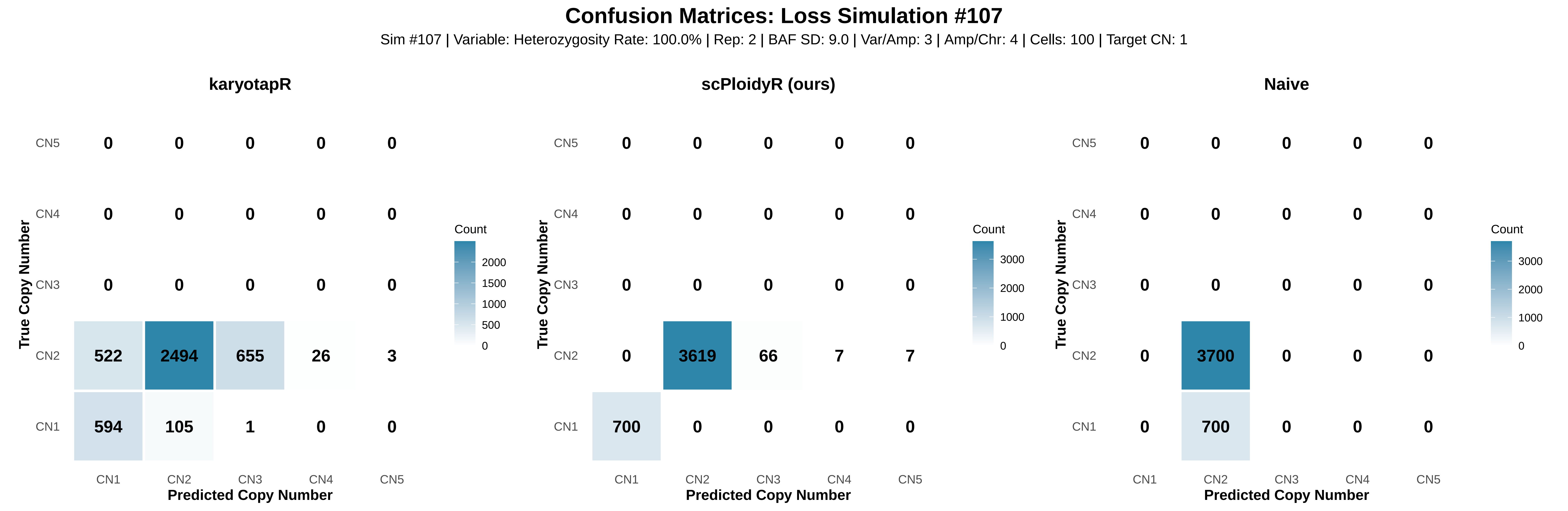
Confusion Matrices: Loss Simulation #107
Sim #107 | Variable: Heterozygosity Rate: 100.0% | Rep: 2 | BAF SD: 9.0 | Var/Amp: 3 | Amp/Chr: 4 | Cells: 100 | Target CN: 1
karyotapR
scPloidyR (ours)
Naive
True Copy Number
Predicted Copy Number
CN1
CN2
CN3
CN4
CN5
Count
522
2494
655
26
3
594
105
1
3619
66
7
7
700
3700
700

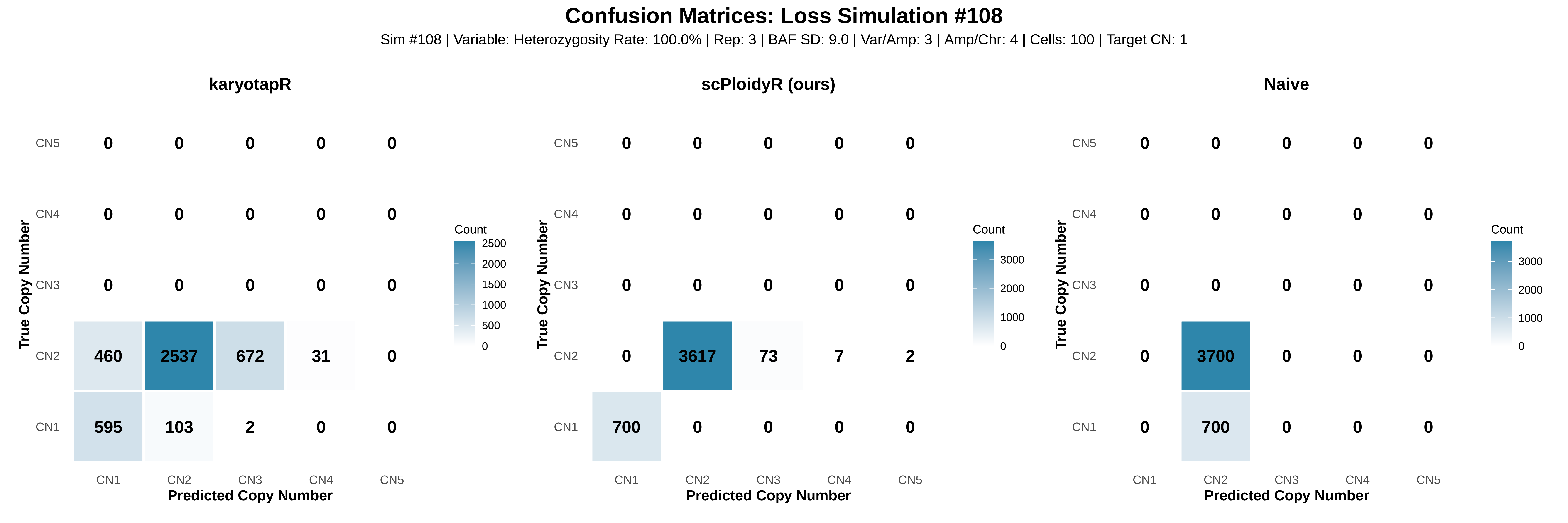
Confusion Matrices: Loss Simulation #108
Sim #108 | Variable: Heterozygosity Rate: 100.0% | Rep: 3 | BAF SD: 9.0 | Var/Amp: 3 | Amp/Chr: 4 | Cells: 100 | Target CN: 1
karyotapR
scPloidyR (ours)
Naive
True Copy Number
Predicted Copy Number
CN1
CN2
CN3
CN4
CN5
Count
2500
2000
1500
1000
500
0
3000
2000
1000
0
460
2537
672
31
595
103
2
3617
73
7
2
700
3700
700
