# Supplementary material for: Integrative Modeling of Read Depth and B-Allele Frequency Improves Single-Cell Copy Number Calling from Targeted DNA Sequencing Panels
Source: Comput Struct Biotechnol J. 2026 Aug 21;35(1):0203. doi: 10.34133/csbj.0203 (PMC13494159; doi:10.34133/csbj.0203)
Supplement: Supplementary file 1 [file csbj.0203.f1.zip › revision_S8_implementation_and_parameters.pdf]

Supplementary File S8. scPloidyR implementation: inputs, outputs, and default parameters

Integrative modeling of read depth and B-allele frequency improves single-cell copy number calling from targeted DNA sequencing panels

This supplementary file documents the required inputs, the preprocessing path, the output format, and the default parameters of scPloidyR, in response to Reviewer #1's Comment 1.9.

Required inputs and preprocessing

scPloidyR does **not** consume the raw Mission Bio Tapestri HDF5 (`.h5`) output directly. Instead, its single exported entry point, `call_copy_number()`, operates on preprocessed per-cell matrices. The standard preprocessing pipeline is performed with the karyotapR package: `createTapestriExperiment()` reads the Mission Bio `.h5` into a `TapestriExperiment` object, `calcNormCounts()` applies the Mission Bio "mb" normalization scheme (Eqs. 1–3 of the main text), and cell populations and reference (diploid) cells are identified by allele-frequency clustering (PCA, UMAP, DBSCAN). B-allele frequencies are extracted from the `alleleFrequency` assay and mapped to amplicons by genomic coordinate. This preprocessing is identical to the standard karyotapR workflow, so scPloidyR slots directly into existing Tapestri analysis pipelines.

`call_copy_number()` requires five inputs:

Input	Type	Description
`depth_matrix`	numeric matrix (amplicons × cells)	mb-normalized amplicon read depths
`baf_data`	list (per cell) of per-amplicon minor-allele-frequency vectors, or a matrix	allelic signal at panel variants
`amplicon_chr_labels`	vector (length = amplicons)	chromosome label of each amplicon
`reference_cell_indices`	integer vector	columns of `depth_matrix` that are reference (diploid) cells
`reference_cn`	scalar, per-chromosome, or per-amplicon	assumed copy number of reference cells (default 2; encodes known aneuploidy such as `c("10" = 3)`)

Output format

The primary output of `call_copy_number()` is `cn_calls_amplicon`, an integer matrix of copy number calls at **per-amplicon, per-cell** resolution (amplicons × cells) — the native resolution of the targeted panel. The call also returns the learned per-chromosome model parameters (state-dependent depth means, depth variance, BAF standard deviation, transition probability, log-likelihood), per-chromosome convergence flags, and timing.

Coarser resolutions are obtained by aggregation:

- **Per segment (chromosome or chromosome arm), per cell:** amplicon-level calls are aggregated to a segment-level matrix using the mode across amplicons within each segment (`aggregate_copy_number()`; Eq. 22 of the main text).
- **Per clone (cell population), per segment:** per-cell segment calls are grouped by their cluster/cell-population assignment to produce clone-level karyotype profiles.

Thus scPloidyR reports copy number per amplicon and per cell natively, and per segment and per clone after aggregation.

Default parameters

Parameters whose default is "learned from reference cells" are initialized from the reference (diploid) population and then re-estimated by Baum–Welch expectation-maximization (EM). All defaults are defined in the package source (`scPloidyR/R/constants.R`) and the `call_copy_number()` signature.

- **Copy number states** (`states`, K): default 1–5 (range 0–5 supported) — hidden CN states modeled by the HMM.
- **Transition probability, initial** (ϵ, `eps_init`): 1×10^{-3} — initial off-diagonal transition probability; re-estimated by EM.
- **Depth variance, initial** (σ_d^2): learned from reference cells — variance of normalized depth across amplicons; re-estimated by EM.
- **BAF (MAF) standard deviation, initial** (σ_b, `sigma_maf_init`): learned, bounded to $[0.05, 0.15]$, or 0.08 if no heterozygous variants — initial truncated-normal SD for MAF emissions; re-estimated by EM.
- **Heterozygosity threshold**: 0.30 — MAF cutoff classifying a reference variant as heterozygous vs. homozygous.
- **Low-heterozygosity threshold**: 0.25 — mean het rate below which depth-anchoring regularization is applied.
- **Depth-anchor prior weight** (w): 10 — weight pulling μ_k toward $k/2$ under low heterozygosity.
- **EM convergence tolerance** (`tol`): 1×10^{-5} — maximum parameter change to declare convergence.
- **Maximum EM iterations** (`max_iter`): 50 — iteration cap if tolerance is not reached.
- **Reference copy number** (`reference_cn`): 2 — assumed CN of reference cells (scalar / per-chromosome / per-amplicon).
- **Near-zero baseline filter**: 0.05 — amplicons with a reference diploid baseline at or below this value are removed in preprocessing.
- **CPU cores** (`ncores`): `detectCores() - 1` — per-cell parallelism (the application used 8 cores).

The Mission Bio mb-normalization preprocessing thresholds (good-barcode cutoff at 10% of the 11th-largest total count, and the +0.05 and +1 stabilizing constants) are karyotapR defaults and are given in Eqs. 1–3 of the main text.
