# Supplementary material for: Integrative Modeling of Read Depth and B-Allele Frequency Improves Single-Cell Copy Number Calling from Targeted DNA Sequencing Panels
Source: Comput Struct Biotechnol J. 2026 Aug 21;35(1):0203. doi: 10.34133/csbj.0203 (PMC13494159; doi:10.34133/csbj.0203)
Supplement: Supplementary file 1 [file csbj.0203.f1.zip › revision_S9_ablation_analysis.pdf]

Supplementary File S9: Ablation analysis of the scPloidyR emission and transition components

Purpose

This supplementary file accompanies our response to Reviewer 1, Comment 1.3. To isolate the separate contributions of (i) integrating B-allele frequency (BAF) with read depth and (ii) the hidden Markov model (HMM) transition structure that enforces spatial smoothing, we benchmarked four variants of the scPloidyR model against the depth-only karyotapR Gaussian mixture model (GMM) and the naive diploid predictor on the simulation study 1 design.

Variant definitions

The four HMM variants differ only in which terms of the per-amplicon emission (Equation 7) are retained and whether the transition matrix (Equation 6) provides smoothing. They are produced by toggling three flags on the single production code path (`call_copy_number()`), so the comparison reflects the model alone and not differences in preprocessing, reference learning, or aggregation:

- **scPloidyR (full)** — depth emission + BAF emission + HMM smoothing. This is the published method.
- **Depth-only HMM** — depth emission + HMM smoothing, BAF emission removed (`use_baf = FALSE`). Isolates the contribution of BAF integration.
- **BAF-only HMM** — BAF emission + HMM smoothing, depth emission removed (`use_depth = FALSE`). Isolates the contribution of the depth channel.
- **Joint non-HMM** — depth emission + BAF emission, transition matrix held uniform so decoding reduces to independent per-amplicon maximum-a-posteriori classification (`use_smoothing = FALSE`). Isolates the contribution of HMM smoothing.

Benchmark and metrics

All variants were evaluated on the simulation study 1 benchmark (six balanced cell populations spanning copy number states 1–5, 22 chromosomes with 4 amplicons per chromosome, 3 variants per amplicon, BAF noise sd = 9, 100% heterozygosity; ten independent replicates with parameter jitter). On each replicate, all six methods were scored on the identical simulated data. We report the same class-balanced and per-class metrics used for simulation study 1: macro-F1, balanced accuracy, altered-versus-diploid alteration F1, and per-class sensitivity for CN 1–5. The full model and the GMM reproduce the simulation study 1 values (full-model macro-F1 0.477 and alteration F1 0.903; GMM 0.273 and 0.381) to within replicate jitter, confirming the variants sit on the same benchmark.

Results

Table S9. Mean performance across ten replicates on the simulation study 1 benchmark. Bold metrics are class-balanced; CN 1–5 columns are per-class sensitivity.

Method	Macro-F1	Balanced acc.	Alteration F1	CN1	CN2	CN3	CN4	CN5
scPloidyR (full)	0.477	0.525	0.903	1.000	0.983	0.382	0.208	0.052
Depth-only HMM	0.101	0.163	0.280	0.119	0.162	0.429	0.083	0.023
BAF-only HMM	0.479	0.527	0.907	1.000	0.985	0.389	0.206	0.055
Joint non-HMM	0.320	0.410	0.541	0.963	0.617	0.217	0.126	0.127
karyotapR (GMM)	0.273	0.310	0.381	0.162	0.520	0.310	0.171	0.387
Naive (diploid)	0.176	0.200	0.000	0.000	1.000	0.000	0.000	0.000

Interpretation

BAF integration is the dominant contributor. Removing the BAF emission (depth-only HMM) collapses macro-F1 from 0.477 to 0.101, balanced accuracy from 0.525 to 0.163, and alteration F1 from 0.903 to 0.280. Under the comprehensive design's realistic overdispersion (per-amplicon depth coefficient of variation about 0.55), the depth channel alone cannot separate five copy number states with only four amplicons per chromosome, and the per-chromosome HMM is prone to mean ("mu") collapse, which is why depth-only HMM even falls below the depth-only GMM (0.273); the GMM is more stable in this regime because it pools depth across cells. The allelic channel therefore supplies most of the multi-class discriminative signal on this benchmark.

HMM smoothing contributes a substantial, separable increment. Removing the transition structure (joint non-HMM) drops macro-F1 from 0.477 to 0.320, balanced accuracy from 0.525 to 0.410, and alteration F1 from 0.903 to 0.541. The effect is concentrated in the diploid background: diploid (CN 2) sensitivity falls from 0.983 to 0.617, and although the per-amplicon decode retains high alteration sensitivity (0.898), its alteration F1 is much lower (0.541), indicating many false-positive alteration calls in diploid regions. Smoothing thus acts mainly to suppress isolated misfires and protect specificity, consistent with the high diploid-region specificity reported in the event-size analysis (Figure 7).

The depth channel's contribution is regime-dependent. On this benchmark, where BAF is rich (three variants per amplicon, full heterozygosity, low BAF noise), BAF-only is comparable to the full model (macro-F1 0.479 vs 0.477) because intermediate minor-allele-frequency values (for example 1/3 at CN 3 and 1/4 at CN 4) already separate most states from the diploid balanced genotype. Depth becomes essential in the opposite regime — when allelic information is sparse or noisy — which is precisely where simulation study 2 quantifies its value: with zero variants per amplicon the model has only depth to rely on (gain accuracy 0.556), and adding a single heterozygous variant raises gain accuracy to 0.897. The joint model is designed to be robust across both regimes: it matches the allele-driven performance when BAF is informative and falls back to depth when BAF is absent, without requiring the user to choose a channel in advance.

Taken together, the ablation isolates the two contributions the reviewer asked about: BAF integration accounts for the large gain over the depth-only GMM (macro-F1 0.477 vs 0.273; alteration F1 0.903 vs 0.381), HMM smoothing adds a further class-balanced and specificity gain over a per-amplicon decode (macro-F1 0.477 vs 0.320), and the two channels are complementary across the conditions spanned by simulation study 2.

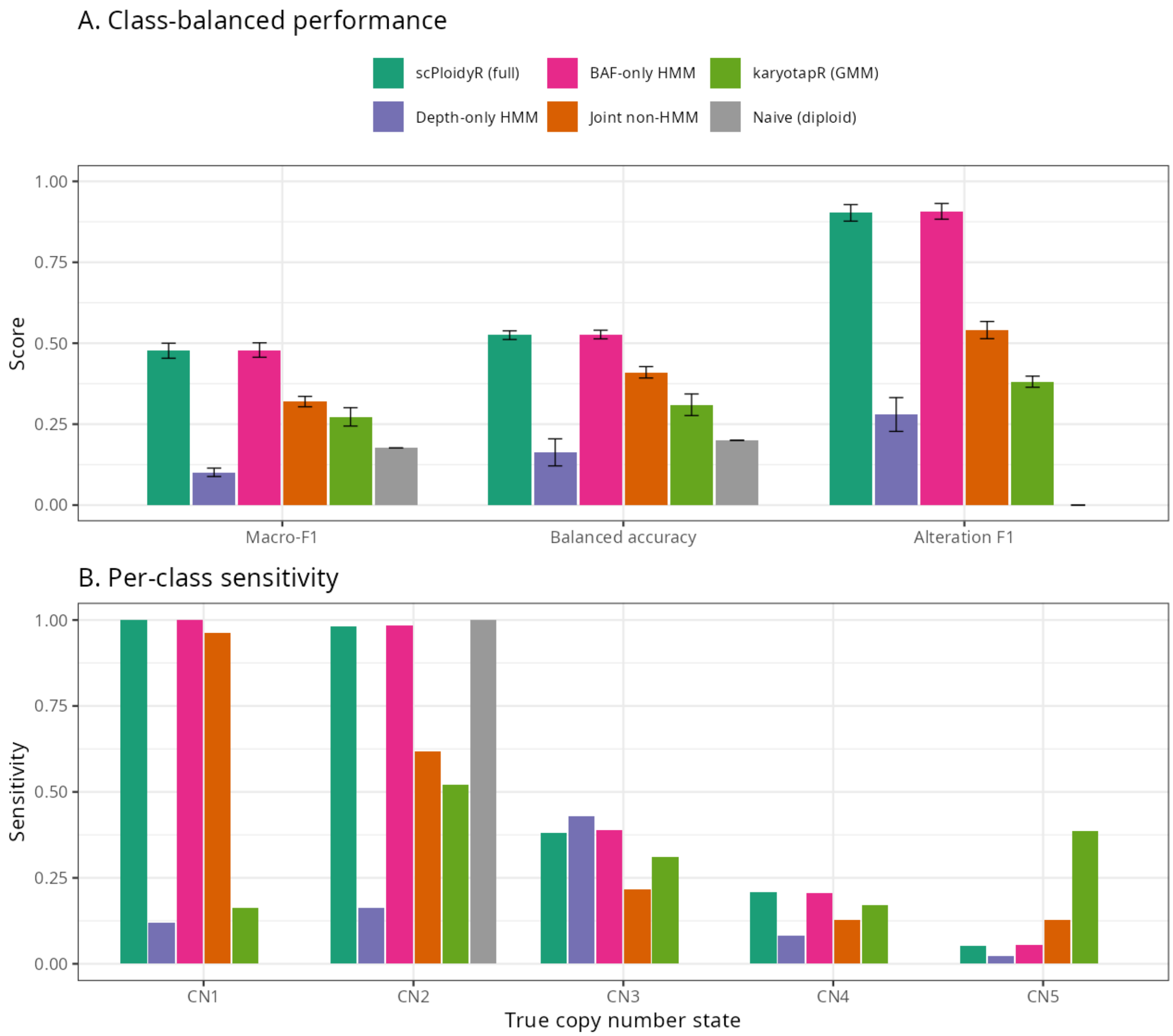

Figure 1: Ablation analysis on the simulation study 1 benchmark. (A) Class-balanced metrics (macro-F1, balanced accuracy, alteration F1; mean over ten replicates, error bars show standard deviation) for the four HMM variants, the depth-only karyotapR GMM, and the naive diploid predictor. (B) Per-class sensitivity for copy number states 1–5.
